# Studies of the Synthesis of Fused Isoxazoline/Isoquinolinones and Evaluation of the Antifungal Activity of Isoxazole-like Benzamide and Isoquinolinone Hybrids

**DOI:** 10.3390/molecules30030589

**Published:** 2025-01-27

**Authors:** Konstantinos A. Ouzounthanasis, Jasmina Glamočlija, Ana Ćirić, Alexandros E. Koumbis

**Affiliations:** 1 Laboratory of Organic Chemistry, Department of Chemistry, Aristotle University of Thessaloniki, 54124 Thessaloniki, Greece; kouzounth@chem.auth.gr; 2 Department of Plant Physiology, Institute for Biological Research “Siniša Stanković”—National Institute of Republic of Serbia, University of Belgrade, 11060 Belgrade, Serbia; jasna@ibiss.bg.ac.rs (J.G.); rancic@ibiss.bg.ac.rs (A.Ć.)

**Keywords:** isoxazole, isoxazoline, isoxazolidine, isoquinolinone, benzamide, 1,3-dipolar cycloaddition, nitrile oxide, Beckmann rearrangement, oxidation, antifungal activity

## Abstract

Isoxazole derivatives (isoxazoles, isoxazolines, and isoxazolidines) are present in the structure of several natural products and/or pharmaceutically interesting compounds. In this work, a synthetic study for the preparation of fused isoxazoline/isoquinolinone hybrids is presented. The initial approach involving the sequential 1,3-dipolar cycloaddition of nitrile oxides to indenone (to obtain the isoxazoline ring) and a Beckmann rearrangement (to construct the isoquinolinone lactam system) was complicated by the formation of fragmentation products during the latter. Therefore, the desired hybrids were successfully reached by applying DDQ-mediated oxidation of the respective isoxazolidines. Based on the results, key observations were made regarding the mechanism of the Beckmann reaction. Moreover, selected isoxazole benzamides and fused isoxazoline/isoxazolidine isoquinolinones were in vitro evaluated against a series of fungi strains (including a 2D checkerboard assay with ketoconazole), revealing that some of these compounds exhibit promising antifungal activity.

## 1. Introduction

**Scheme 1:**
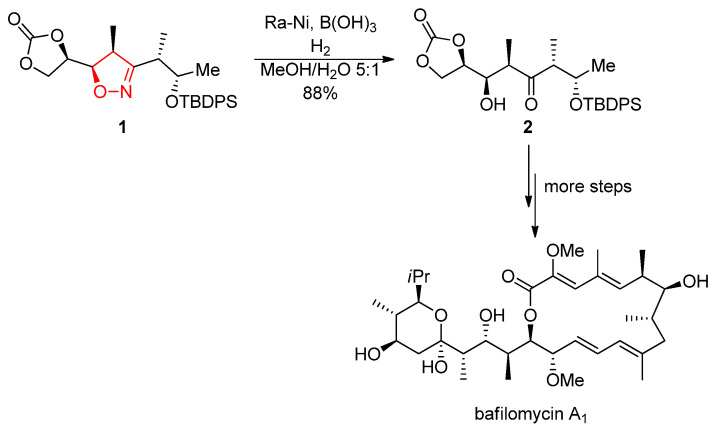
An example of using a key intermediate isoxazoline for the total synthesis of a natural product [4].

**Scheme 2:**
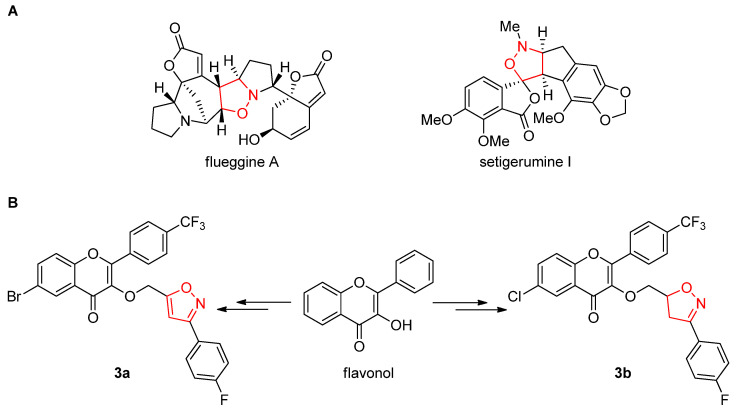
(**A**) Examples of naturally occurring isoxazolidine compounds [9,10]; (**B**) examples of medicinally designed isoxazole and isoxazoline derivatives [12].

Isoxazoles, isoxazolines, and isoxazolidines constitute families of five-membered heterocycles with diverse applications [1,2,3]. In synthetic organic chemistry, those heterocycles may serve as valuable building blocks for constructing more complex compounds upon further functionalization (e.g., Scheme 1, [4]). Additionally, numerous biologically active molecules and pharmaceutical compounds incorporate these moieties as integral structural features [5,6,7]. It is worth noting that even though the isoxazolidine ring scaffold has been found in natural products [8,9,10] (Scheme 2A), isoxazoles and isoxazolines have been widely employed in medicinal chemistry as tools of synthetic modifications of natural products to enhance various properties (pharmacokinetics, activity, selectivity, etc.), thereby optimizing drug candidates [11,12] (e.g., Scheme 2B). In particular, a significant number of isoxazole-like compounds have been found to exhibit antifungal activity [7,13,14,15,16].

**Scheme 3:**
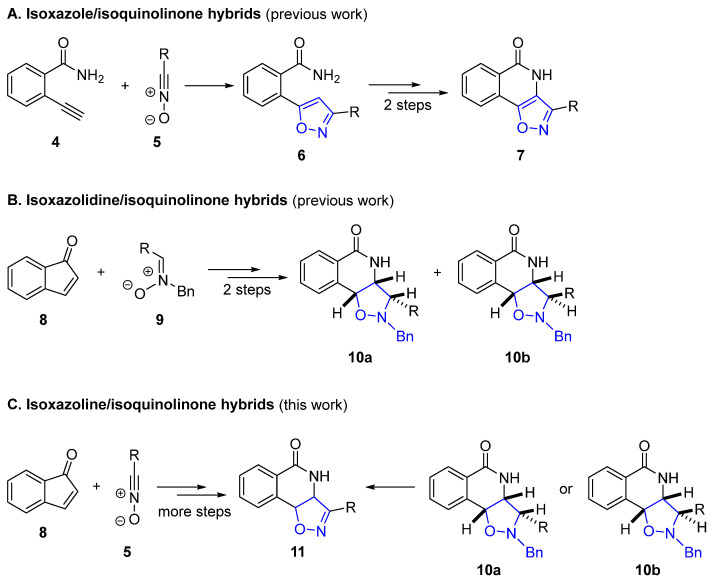
Strategies for the synthesis of isoquinolinone hybrids **7**, **10a**,**b**, and **11**.

In our previous work [17], we reported a couple of concise synthetic strategies for accessing novel isoxazole (**7**) and isoxazolidine (**10a**,**b**) hybrid compounds (Scheme 3A,B) as the result of merging these five-membered heterocycles with the privileged scaffold of isoquinolinone [18] and several new isoxazole benzamide precursors. Those hybrids represent the first reported derivatives of isoquinolinone with the isoxazole and isoxazolidine ring fused at C3 and C4 of the isoquinolinone template. To our knowledge, references regarding fused isoquinolinones with five-membered heterocycles at the aforementioned positions are exceptionally rare, despite the potential of such compounds to show alluring activity against a wide range of biological targets [19]. However, establishing a dependable methodology for the facile preparation of the corresponding isoxazoline/isoquinolinone fused hybrids (**11**) proved to be a rather challenging task (Scheme 3C).

Fungal infections trigger about 1.7 million deaths per year worldwide, mostly in immunocompromised individuals with two or more pathological conditions [20]. With the widespread use of antifungal drugs, the prevalence of resistance to each drug has also increased. Therefore, new drugs need to be developed that are more effective and have fewer side effects, as both fungi and mammals are eukaryotes and, therefore, have many common drug targets. Thanks to advances in medical technology and research, new drugs can now be developed that are more effective against fungal infections. In addition, new antifungal strategies, innovative delivery methods, and synergistic approaches are needed.

Most fungal infections are caused by Candida, Cryptococcus, and Aspergillus species, which kill more than one million people every year [21]. The species responsible for Aspergillus infections mainly include *Aspergillus fumigatus*, *Aspergillus flavus*, *Aspergillus terreus*, and *Aspergillus niger* [22]. Although *Candida albicans* is often found as part of the healthy human microbiome, it is one of the most common fungal pathogens in humans. Recurrent infections, antimicotic resistance, and a mortality rate of up to 54% for candidiasis indicate that this species poses a serious risk to human health and a significant economic burden to our society [23]. Several Penicillium species, such as *P. chrysogenum*, are known to be both allergens and pathogens. Penicillium spores can be detected in the air throughout the year, but they can also show seasonal fluctuations. They can cause a variety of infections, especially in people with a weakened immune system due to an existing disease [24]. The treatment of choice for invasive diseases recently expanded to first-line treatments, and azole resistance represents an emerging problem, with primary resistance observed in several fungal species.

Herein, we report our efforts to develop a new synthetic strategy for accessing hybrid compounds **11** (Scheme 3C), ultimately leading to a complete collection of isoquinolinone fused hybrids with the isoxazole family of five-membered heterocycles, making these compounds readily available for extensive biological screening. Additionally, we present the in vitro antifungal activity evaluation of selected compounds of isoxazoline and isoxazolidine isoquinolinone and isoxazole benzamide libraries (prepared in this work or as previously described by us [17]) against Aspergillus, Penicillium, and Candida species, as well as the evaluation of the vitro combination of ketoconazole with the compounds that showed the best antifungal activity.

## 2. Results and Discussion

### 2.1. Retrosynthetic Plans for the Targeted Hybrids

**Scheme 4:**
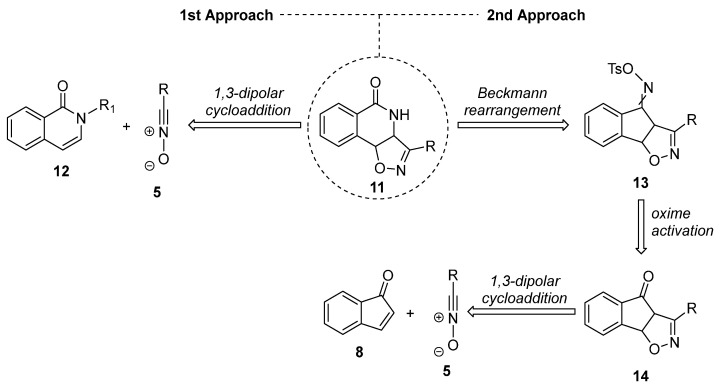
The initial retrosynthetic strategies towards isoxazoline/isoquinolinone hybrids.

As a more direct approach to obtaining the desired hybrid compounds, we envisioned the 1,3-dipolar cycloaddition of *N*-substituted isoquinolinone **12** with various nitrile oxides. An alternative route for preparing the target compounds could involve a Beckmann rearrangement of tosyl oxime **13**. The latter can be prepared from ketone **14**, which are the adducts of an alkene–nitrile oxide 1,3-dipolar cycloaddition reaction to indenone (**8**) (Scheme 4). In the next section, we thoroughly discuss the challenges encountered during the process, our attempts to overcome them, and the successful synthesis of some isoxazoline/isoquinolinone hybrids.

### 2.2. Attempted Synthesis Directly from Isoquinolinone

**Scheme 5:**
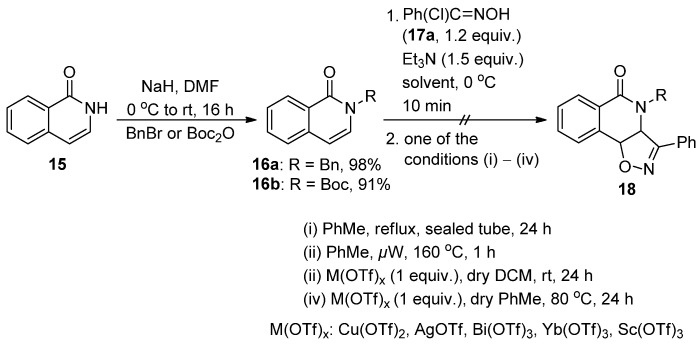
Attempted 1,3-dipolar cycloadditions to *N*-substituted isoquinolinones.

In our initial synthetic approach, isoquinolinone **15** was treated with NaH in DMF and an appropriate electrophile, affording the respective *N*-substituted isoquinolinones **16a** [25] and **16b** in excellent yields (Scheme 5). The preparation of **16** was deemed necessary to disrupt the aromaticity of isoquinolinone in an attempt to enable it to participate as the dipolarophile partner in the subsequent 1,3-cycloaddition reaction. Hydroxamoyl chloride precursor **17a** [17] was used for the in situ preparation of the model nitrile oxide to investigate the various conditions of the desired cycloaddition reaction. Both **16a** and **16b** were refluxed in toluene for 24 h without any sign of the desired product forming. Similarly, conducting the reaction under microwave irradiation at 160 °C also failed to afford isoxazoline cycloadducts (Scheme 5). Additionally, Lewis acids were employed as additives to facilitate the reaction. Triflate salts of various metals [M(OTf)_x_] were used for this purpose; however, none of the catalysts employed yielded the desired product (Scheme 5). In all cases, isoquinolinone **16a** was recovered intact, whereas the deprotection of *N*-Boc starting material **16b** was observed with all metal triflates, except Sc(OTf)_3_. There are several examples of *N*-vinyl amides in the literature participating in 1,3-dipolar cycloaddition reactions without any difficulties [26,27]; hence, we assume that the nitrogen atom in combination with the bicyclic structure of isoquinolinone is the main issue preventing the substrate from acting as a dipolarophile.

**Scheme 6:**
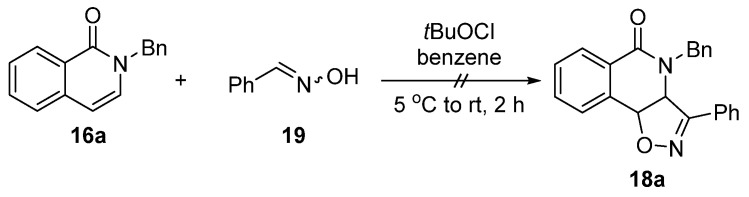
Testing cycloaddition with an in situ generated chloroxime.

In a final attempt, the reaction of *N*-benzyl isoquinolinone **16a** with benzaldoxime (**19**) in the presence of *t*BuOCl in benzene [28] resulted in the degradation of the starting materials (Scheme 6).

Conclusively, despite the fact that isoquinolinone was ‘trapped’ in its non-aromatic tautomeric form, it failed to act as a dipolarophile in a 1,3-dipolar cycloaddition under both conventional heating and microwave irradiation, even under Lewis acid catalysis conditions. At this point, it was clear that this approach for more direct access to isoxazoline/isoquinolinone hybrid compounds was not feasible.

### 2.3. Exploring the Synthesis of Isoxazoline/Isoquinolinone Hybrids via Beckmann Rearrangement

Based on our assumption that the main issue lies in the presence of the nitrogen atom already existing in the structure of the substrate, the cycloaddition reaction could be conducted on a different bicyclic starting material. Subsequently, the nitrogen atom could be introduced through a rearrangement reaction. This general strategy had been successfully exploited by us [17] for the preparation of isoxazolidine adducts. Thus, indenone (**8**) could serve as the new dipolarophile, and after proper modifications, a Beckmann rearrangement could introduce the amide functional group. Due to its instability, indenone (**8**) was synthesized in situ from 3-bromoindan-1-one (**20**) prior to the cycloaddition reaction [17], ensuring the integrity of the methodology. Moreover, the required hydroxamoyl chlorides were prepared from the corresponding aldehydes using a known general approach [17].

The cycloaddition reaction between **8** and the nitrile oxide derived from benzaldehyde has already been presented in our previous work [17]. Following a similar logic, the previously prepared hydroxamoyl chloride **17** and bromide **20** were treated with Et_3_N in DCM (Table 1). The reaction proceeded smoothly in all cases, affording regioselectively the major cycloadduct **14** in very good yields. In most instances, the minor cycloadduct **21** was also isolated, albeit in meager yields. The predominance of cycloadduct **14** is not surprising since indenone as an α,β-unsaturated ketone should favor the approach of the negatively charged oxygen atom of the 1,3-dipole to the less electron-rich β-carbon.

**Scheme 7:**
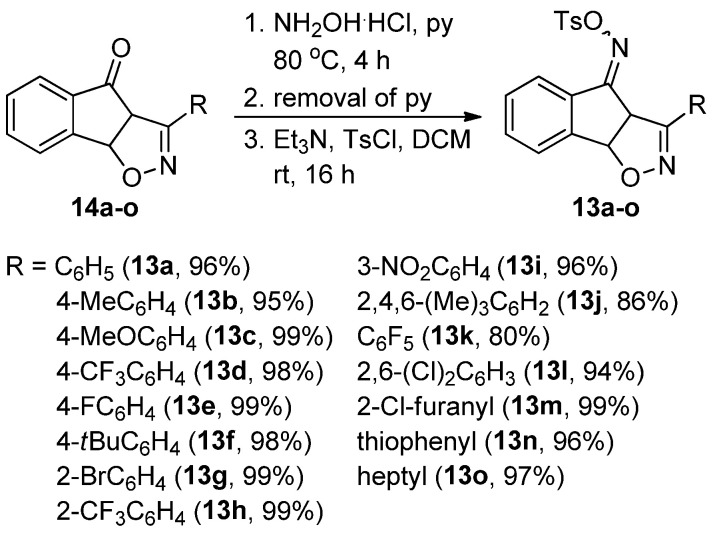
One-pot protocol for the preparation of tosyl oximes **13**.

To avoid the more conventional methods for the rearrangement involving direct application of harsh conditions, such as strong inorganic acids (e.g., H_2_SO_4_ and PPA) and high temperatures that are unsuitable for sensitive substrates [29], we decided to convert ketone **14** into its more activated tosyl oxime analog [30]. This transformation was expected to facilitate the subsequent Beckmann rearrangement, allowing it to proceed under milder conditions. Initially, ketone **14a** was treated with hydroxylamine hydrochloride in pyridine at 80 °C. Upon completion of the oxime formation, TsCl was added directly to the reaction mixture, and the desired tosyl oxime **13a** was isolated in 70%. However, this one-pot procedure was deemed only moderately efficient, prompting a slight modification. In the improved protocol, after the completion of the oxime formation, pyridine was removed in vacuo, and the reaction mixture was redissolved in DCM. Et_3_N and TsCl were subsequently added, leading to the isolation of the desired tosyl oxime in an excellent yield (Scheme 7). This protocol was then applied to the rest of the ketone derivative **14**, furnishing tosyl oxime **13** in high yields without any significant issues. Notably, tosyl oximes **13** were crystallized from methanol and collected pure enough via simple filtration, eliminating any further need for chromatographic purification.

**Scheme 8:**
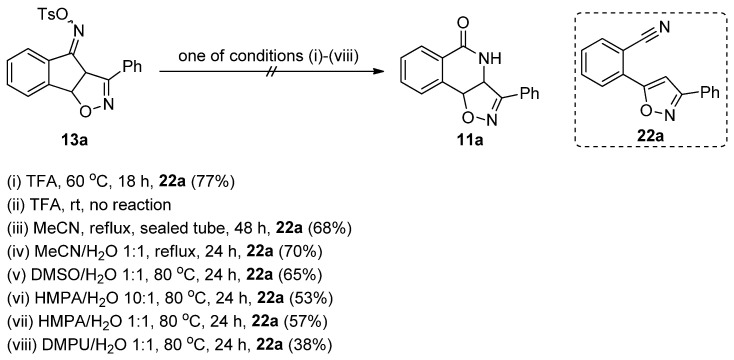
Beckmann fragmentation instead of rearrangement of tosyl oxime **13a**.

**Scheme 9:**
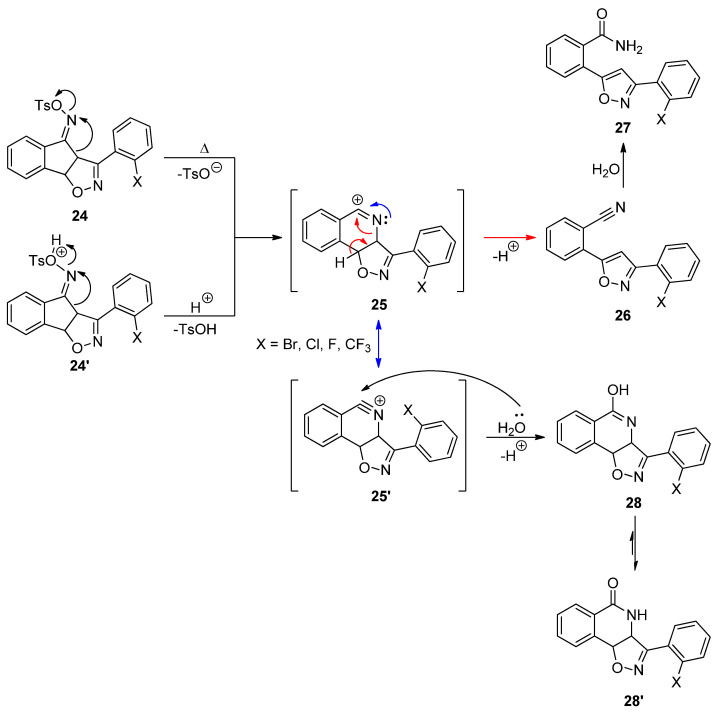
A possible mechanistic explanation for the Beckmann reaction products.

The next step in this methodology was the crucial Beckmann rearrangement. Tosyl oxime **13a** was selected as the model compound and treated with trifluoroacetic acid (TFA) at 60 °C. A single product was isolated; however, it was not the desired isoxazoline/isoquinolinone hybrid. Instead, the compound was identified as nitrile **22a**, the product of a Beckmann fragmentation reaction rather than the intended rearrangement. Although nitriles are known Beckmann rearrangement side products [31], in this case, the reaction exclusively yielded the nitrile without any trace of the expected amide product (Scheme 8). The formation of the aromatic isoxazole ring is likely responsible for this outcome, strongly driving the reaction toward the benzonitrile derivative. It seems that the carbocation intermediate (shown in Scheme 9), formed during the Beckmann rearrangement mechanism, has an extremely short lifetime, making it unable to be quenched, even by water. Nonetheless, the fact that the *sp*^3^ carbon group was the one migrating to the nitrogen atom is worth mentioning. At this point, a small-scale investigation was conducted to explore whether it was possible to increase the lifetime of this carbocation and achieve the isolation of the desired amides, even in low yields. The TFA-mediated transformation was attempted again at room temperature but failed to give any reaction. Subsequently, we excluded the acid from the reaction conditions and applied heat to **13a** in an appropriate solvent. Once more, the isolated product was nitrile **22a**, although the starting material required notably more time to be consumed. Next, we introduced water into the solvent system and applied heat, aiming to increase the likelihood of the carbocation intermediate being trapped by a water molecule. However, this approach also proved unsuccessful. As a final attempt, we employed solvents known to stabilize generated carbocations and potentially extend their lifetime [32,33], but the obtained product was nitrile **22a** in every case (Scheme 8).

Despite the unsuccessful Beckmann rearrangement attempts, we decided to apply the TFA-mediated conditions to the previously prepared tosyl oxime derivatives (**13**). Additionally, the reaction scale was increased using nearly 1 g of starting material. As expected under these conditions, the formation of the nitrile was predominant in almost all entries (Table 2). Interestingly, however, the formation of additional products was observed. Primary benzamides **23** was isolated in most cases, albeit in low but notable yields ranging from 6% to 32%. It appeared reasonable to speculate that these benzamides were formed via hydrolysis of the corresponding nitriles as a post-Beckmann fragmentation transformation. Indeed, the hydrolysis of **22a** to **23a** was verified by conducting control experiments at 60 °C for 18 h (Table 3), which showed that nitrile **22a** is slowly hydrolyzed in the presence of *p*-TsOH (entry 3). Notably, such behavior was not observed in the smaller-scale model reaction of **13a**. To our surprise, we detected for the first time the formation of the desired isoxazoline/isoquinolinone hybrid compounds (Beckmann amides) in the reactions of four of the tosyl oximes tested.

The four tosyl oximes that underwent the rearrangement pathway, even in low yield, share a structural similarity that might explain this unexpected result, contrary to our previous observations. These derivatives bear an aromatic ring with an appropriate substituent at the *ortho* position (Scheme 9). This combination provides an intrinsic stabilization to the carbocation intermediate formed during the Beckmann rearrangement (**25′**), extending its lifetime just enough for a water molecule to attack it, ultimately leading to the formation of the amide product. That being said, this synthetic approach allows access to only certain isoxazoline/isoquinolinone hybrids (albeit in low yields), largely determined by the substrate’s structural features.

**Scheme 10:**
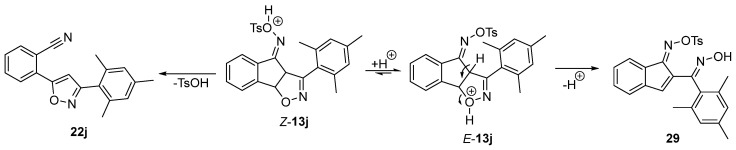
A reasonable explanation for the formation of compound **29**.

Lastly, a unique behavior was observed with mesityl derivative **13j**. The respective nitrile product (**22j**) was isolated in a meager 12% yield, while a new, unknown compound was found to be the major product (65%). This was identified as oxime **29** (Scheme 10). The Beckmann rearrangement (or fragmentation) requires the migrating group to be in an antiperiplanar position relative to the leaving group [34,35]. In this case, the position of the nitrogen atom in the isolated products suggests that the *Z*-tosyl oxime isomer is the one that undergoes the reaction. While *E* to *Z* isomerization of tosyl oximes can take place under the reaction conditions, it seems that for derivative *E*-**13j**, an interaction between the tosyl and mesityl groups favors the formation of the latter, which undergoes elimination towards **29**.

### 2.4. Isoxazoline/Isoquinolinone Hybrids via Oxidation of the Corresponding Isoxazolidines

**Scheme 11:**
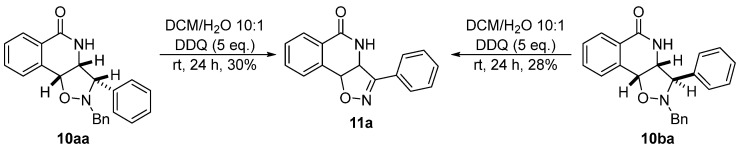
DDQ-mediated synthesis of isoxazoline **11a** from the corresponding isoxazolidines.

Even though we successfully accessed some isoxazoline/isoquinolinone hybrids, the outcome was not ideal. From our previous work [17], isomeric isoxazolidines **10aa** and **10ba** were already available in our lab. As a last resort, we envisioned that an oxidative removal of the benzyl group could afford the corresponding desired isoxazoline derivatives. Both isomers were treated separately with DDQ in a solvent system of DCM/H_2_O 10:1 at room temperature [36], and indeed, the same target isoxazoline **11a** was isolated (Scheme 11). Moreover, no reaction occurred when DCM was replaced with MeCN, benzene, or 1,4-dioxane, whereas CHCl_3_ gave similar results. Gently heating the reaction mixture to 40 °C (DCM or CHCl_3_) showed no spectacular difference as well. However, replacing the biphasial solvent system with wet DCM increased the productivity of the transformation (Table 4, entries 1 and 2). Interestingly, the starting material has not been consumed, even after prolonged reaction times and, practically, the ratio of product/starting material remained unchanged after 24 h.

The same protocol was then tested using selected isoxazolidine precursors as starting materials, confirming the generality of its efficiency (Table 4). Although the yields were modest (41–75%, with the remaining starting material fully recovered), this method enables the preparation of various isoxazoline/isoquinolinone hybrid compounds, potentially expands the substrate scope, and offers a feasible pathway for accessing these hybrid structures.

### 2.5. Evaluation of Antifungal Activity

**Figure 1:**
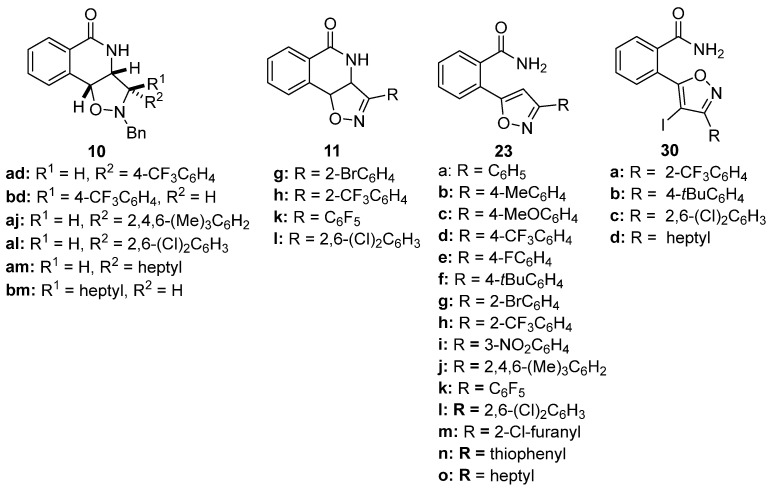
Compounds used in the antifungal assay.

The synthesized compounds tested are categorized into four groups according to their structure, i.e., isoxazole benzamide **23**, 4-iodoisoxazole benzamide **30**, isoxazoline isoquinolinone **11,** and isoxazolidine isoquinolinone **10** (Figure 1). Those heterocyclic derivatives were evaluated for their antifungal activity against six fungal species using the microdilution method, and the results are presented in Table 5 and Table 6.

According to the obtained results, isoxazole benzamide **23**, which showed activity at all, had MIC values ranging between 0.04 and 0.24 mg/mL and MFC values ranging between 0.08 and 0.32 mg/mL. These values are higher than the ones for the commercially available reference drug ketoconazole (MIC 0.0004–0.05 mg/mL and MFC 0.0008–0.10 mg/mL) with a deviation for the species *A. niger*, where the MIC/MFC values of the mycotic were 0.10 and 0.20 mg/mL, respectively (Table 5). The best activity was observed for compound **23j**, with MIC in the range of 0.04–0.16 mg/mL and MFC in the range of 0.08–0.24 mg/mL. Moreover, different species showed varying sensitivity towards the compounds tested. Thus, *P. funiculosum* and *A. niger* exhibited sensitivity to all the tested compounds, whereas yeast *C. albicans* was resistant to seven out of fifteen of them. Compared to ketoconazole activity, most compounds were found to be superior against *A. niger*, and some were equally good against *P. chrysogenum*. 4-Iodoisoxazole benzamide **30** was active against the six fungal species, with MIC values ranging between 0.02 and 0.16 mg/mL and MFC values ranging between 0.04 and 0.32 mg/mL (Table 5). The activity of those derivatives was higher against *A. niger* and, actually, much higher than that of ketoconazole, while the results against *P. chrysogenum* are also interesting.

Isoxazoline isoquinolinone hybrid **11** was active against the six fungal species as well. Their MIC values ranged between 0.01 and 0.16 mg/mL and their MFC values ranged between 0.02 and 0.32 mg/mL (Table 6). More specifically, among those hybrids, compound **11l** showed excellent activity against *A. niger*. Analogously, isoxazolidine isoquinolinone hybrid **10** acted on all tested micromycetes and yeasts, with MIC ranging from 0.005 to 0.16 mg/mL and MFC ranging from 0.01 to 0.32 mg/mL, showing better results for *A. niger* (Table 6). The most potent compound was **10bm**, exhibiting 10–100-fold higher activity than ketoconazole. Additionally, derivative **10al** showed excellent activity, while micromycete *A. niger* was also quite sensitive to hybrids **10aj** and **10am**.

Comparing the different libraries tested, it is obvious that collectively, isoxazolidine/isoquinolinone hybrids **10** exhibits higher activity. It is also worth noting that for each category, the best results were observed for compounds bearing relatively bulky substituents on the isoxazole-like scaffold (e.g., compounds **23j**, **30b**, **11l**, **10aj,** and **10al**). Exceptions were heptyl derivatives **10am** and **10bm**. More interestingly, comparing those two isomers, **10bm** (*anti*) was generally more active than **10am** (*syn*).

**Figure 2:**
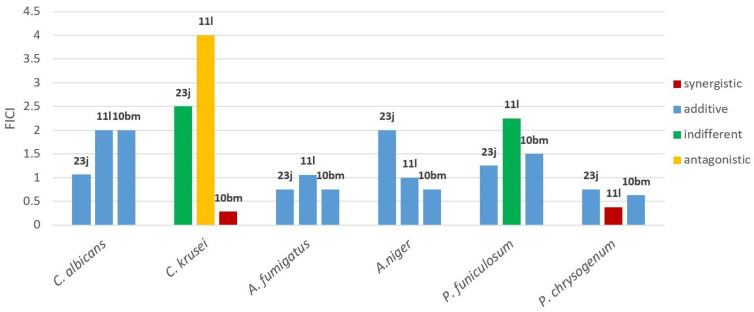
The fractional inhibitory concentration index (FICI) values of compounds **23j**, **11l**, and **10bm** against selected fungal strains.

Based on the above results, compounds **23j**, **11l**, and **10bm** were selected for further evaluation since they showed, representatively for each category, the best inhibitory effect on the most tested fungi. The activity of those compounds was in vitro examined against the six selected fungal strains using a two-dimensional checkerboard with two-fold dilution in order to determine the interactions (synergism, additive effect, indifference, or antagonism) between them and the antifungal agent (ketoconazole) in combinations. The results of those experiments are presented in Table 7, and a summarized presentation is given in Figure 2. The combination of compound **23j** with ketoconazole showed additive effects on all strains, except it had an indifferent effect on *C. krusei*. Regarding the experiments with compound **11l** and ketoconazole, synergistic effects existed for one micromycete *P. chrysogenum*, with an FICI value of 0.375 mg/mL, and additive effects were observed for *A. fumigatus* and *A. niger*, whereas the effect was indifferent for *P. funiculosum* and antagonistic for *C. krusei*. Finally, the combination experiments of compound **10bm** with ketoconazole showed synergistic effects for *C. krusei* (FICI 0.28) and additive effects for the rest of the strains. Notably, the three compounds exhibited completely different effects on *C. krusei*, while the compounds were generally found to act additively in most of the cases. Moreover, the synergistic effects observed in the two combination experiments do not correspond to strains for which the specific compounds showed the highest activity (Table 7).

**Table 7:** In vitro screening of compounds **23j**, **11l**, and **10bm** in combination with ketoconazole against selected fungal strains using checkerboard methodology (mg/mL).

Compound	Strain	MIC1° (Compound)	MIC2° (K ^1^)	FIC1°/FIC2° (Compound/K)	FICI
**23j**	*C. albicans*	0.08	0.0125	0.005/0.0125	1.06
	*C. krusei*	0.16	0.0004	0.08/0.0008	2.50
	*A. fumigatus*	0.08	0.025	0.04/0.00625	0.75
	*A.niger*	0.04	0.10	0.04/0.10	2.00
	*P. funiculosum*	0.04	0.0125	0.04/0.00315	1.25
	*P. chrysogenum*	0.08	0.05	0.02/0.025	0.75
**11l**	*C. albicans*	0.16	0.0125	0.16/0.0125	2
	*C. krusei*	0.16	0.0004	0.32/0.0008	4
	*A. fumigatus*	0.16	0.025	0.01/0.025	1.06
	*A.niger*	0.01	0.10	0.005/0.05	1
	*P. funiculosum*	0.04	0.0125	0.08/0.00315	2.25
	*P. chrysogenum*	0.16	0.05	0.01/0.0125	0.375
**10bm**	*C. albicans*	0.16	0.0125	0.16/0.0125	2
	*C. krusei*	0.16	0.0004	0.01/0.0002	0.28
	*A. fumigatus*	0.16	0.025	0.08/0.00625	0.75
	*A.niger*	0.005	0.10	0.0025/0.025	0.75
	*P. funiculosum*	0.04	0.0125	0.04/0.00625	1.50
	*P. chrysogenum*	0.16	0.05	0.02/0.025	0.625

^1^ K = ketoconazole.

## 3. Experimental Section

### 3.1. General Information

All reactions requiring anhydrous conditions were conducted under an inert (argon) atmosphere using an oven (160 °C) or flame-died (under vacuum) glassware. Anhydrous DMF was prepared by drying overnight over barium oxide (BaO), followed by decantation of the drying agent and distillation under reduced pressure. Anhydrous toluene was distilled over sodium/benzophenone. Anhydrous DCM was distilled over calcium hydride (CaH_2_). All anhydrous solvents were stored in pre-dried Schlenk Kjeldahl storage flasks, containing activated molecular sieves (4 Å), and they were allowed to stand for at least 24 h. All reactions requiring high temperatures were conducted in silicon oil baths. Flash column chromatography was performed by employing silica gel 60 [(40–63 μm, Merck, KGaA (Darmstadt, Germany)]. TLC plates (0.25 mm silica gel 60 F_254_) used for reaction monitoring were purchased from Merck KgaA (Darmstadt, Germany) and were visualized through exposure to ultraviolet light (UV) and/or exposure to an acidic solution of *p*-anisaldehyde or a solution of ninhydrin stain, followed by heating with a heat gun (400 °C). All commercially available reagents and solvents were purchased from Fluorochem Ltd. (Dublin, Ireland), Sigma-Aldrich (MilliporeSigma, St. Louis, MO, USA) & Merck KGaA (Darmstadt, Germany), Fischer Scientific GmbH (Scwerte, Germany), and TCI Chemicals (Tokyo, Japan) and used without further purification. Molecular sieves (3 Å and 4 Å) were activated by being heated with a propane torch in a round-bottom flask under a high vacuum for 1–2 min, and the procedure was repeated 2–3 times. Celite^®^ 545 was purchased from Fluorochem Ltd. (Dublin, Ireland). ^1^H, ^13^C, ^19^F, and 2D NMR spectra were recorded with an Agilent-500/54 spectrometer. All NMR spectra were recorded at 25 °C. Proton chemical shifts are reported in parts per million (*δ* scale) and are calibrated relative to a residual non-deuterated solvent as an internal reference (CDCl_3_: *δ* 7.26, DMSO-*d*_6_: *δ* 2.5 ppm). Carbon chemical shifts are reported in parts per million (*δ* scale) and are referenced from the central peak of the carbon resonance of the solvent (CDCl_3_: *δ* 77.00, DMSO-*d*_6_: *δ* 40.00 ppm). Infrared (IR) data were recorded in a scan range from 400 to 4000 cm^−1^ on a Thermo Scientific Nicolet 6700 FT-IR spectrometer equipped with a diamond attenuated total reflection (ATR) stage. HRMS data were acquired using an Agilent 6540 HRMS-QTOF model equipped with a Dual AJS ESI-MS system or with a Q-TOF (Time of Flight Mass Spectrometry) Maxis Impact (Bruker Daltonics, Bremen, Germany) with an ESI source and U-HPLC Thermo Dionex UltiMate 3000 RSLC (ThermoFisher Scientific, Dreieich, Germany) pump and autosampler. Melting points were determined on an A.KRÜSS Optronic Melting Point Meters KSP1N model apparatus. Reactions under microwave irradiation were carried out using a Biotage Initiator+ microwave synthesizer.

### 3.2. General Procedure for the Synthesis of N-Substituted Isoquinolinones ***16***

To a pre-dried round-bottom flask containing NaH (90%, 101 mg, 3.78 mmoles, 1.1 equiv.), anhydrous DMF (3 mL) was added under an argon atmosphere. The reaction flask was then placed in an ice bath, and isoquinolin-1-one (**15**, 0.5 g, 3.44 mmoles, 1 equiv.) was dropwise added as a DMF solution (2 mL) over a period of 10 min. After stirring at 0 °C for 15 min., benzyl bromide (0.49 mL, 4.13 mmoles, 1.2 equiv.) or Boc_2_O (0.95 mL, 4.13 mmoles, 1.2 equiv.) was dropwise added, and the reaction mixture was stirred at room temperature for 16 h. The resulting mixture was diluted with EtOAc (10 mL), quenched with H_2_O (15 mL), and transferred into a separatory funnel. The organic layer was separated, and the aqueous layer was extracted with EtOAc (2 × 10 mL). The organic layers were combined, washed with saturated brine (30 mL), and dried over anhydrous Na_2_SO_4_. The solvent was removed in vacuo, and the crude mixture was purified through flash column chromatography (silica gel, *n*-hexane/EtOAc 10:1 to 5:1 *v*/*v*) to afford compounds **16**.

### 3.3. General Procedure for the Preparation of Hydroxamoyl Chlorides ***17***

Hydroxamoyl chlorides **17** were prepared from the corresponding aldehydes following a previously reported synthetic approach [17,37] and were used without further purification.

### 3.4. Synthesis of 3-Bromo-2,3-dihydro-1H-inden-1-one ***20***

Bromide **20** was prepared following a slightly modified procedure previously reported [17,38].

### 3.5. General Procedure for the Preparation of Isoxazoline Adducts ***14*** and ***21***

To a round-bottom flask containing bromide **20** (1.05 g, 5 mmoles, 1 equiv.), DCM (80 mL, 16 mL/mmole) was added, and the orange solution was placed in an ice bath. Et_3_N (0.84 mL, 6 mmoles, 1.2 equiv.) was dropwise added over a period of 10 min, and the bright yellow solution was stirred for another 10 min at 0 °C. The corresponding hydroxamoyl chloride **17** (6 mmoles, 1.2 equiv.) was then added to the reaction mixture as a DCM solution (120 mL, 20 mL/mmole) via a pressure-equalizing dropping funnel over a period of 1 h, closely maintaining the temperature at between 0 and 5 °C. Upon completion of the addition, the yellowish mixture was stirred for another 1 h and was gradually allowed to reach room temperature. The reaction mixture was then transferred into a separatory funnel containing semi-saturated brine (250 mL), the organic layer was separated, and the aqueous layer was extracted with DCM (2 × 200 mL). The organic layers were combined, washed with saturated brine (600 mL), and dried over anhydrous Na_2_SO_4_. The solvent was removed in vacuo, and the crude mixture was purified through flash column chromatography (silica gel, *n*-hexane/EtOAc 20:1 to 1:1 *v*/*v*) to afford major adduct **14** and, in most cases, also minor adduct **21**. All characterization and spectroscopic data for compounds **14** and **21** are given in the Supplementary Materials.

### 3.6. General Procedure for the One-Pot Synthesis of Tosyl Oxime ***13***

To a round-bottom flask containing adduct **14** (4 mmoles, 1 equiv.), pyridine (20 mL, 5 mL/mmole) and NH_2_OH·HCl (12 mmoles, 3 equiv.) were sequentially added at room temperature. Then, the colorless solution was stirred for 4 h at 80 °C. Upon consumption of the starting ketone, as indicated by TLC, pyridine was removed in vacuo, and the resulting mixture was redissolved with the addition of anhydrous DCM (20 mL, 5 mL/mmole) under an Ar atmosphere at room temperature. Anhydrous Et_3_N (1.67 mL, 12 mmoles, 3 equiv.) was added, and the reaction mixture was stirred for 15 min at that temperature, followed by the addition of TsCl (1.14 g, 6 mmoles, 1.5 equiv.) as a single portion. After stirring for 16 h at room temperature, the reaction mixture was diluted with DCM (20 mL), quenched through the addition of saturated aq. NH_4_Cl solution (50 mL), and transferred into a separatory funnel. The organic layer was separated, and the aqueous layer was extracted with DCM (2 × 40 mL). The organic layers were combined, washed with aq. HCl solution (2M, 100 mL) and saturated brine (100 mL), and dried over anhydrous Na_2_SO_4_. The solvent was removed in vacuo, and MeOH (10 to 15 mL) was added to the crude mixture to completely dissolve it. After 2–3 min, the corresponding tosyl oxime **13** crystallized as white crystals and was collected via simple filtration. All characterization and spectroscopic data for compound **13** are given in the Supplementary Materials.

### 3.7. General Procedure for the Beckmann Rearrangement/Fragmentation

To a round-bottom flask containing tosyl oxime **13** (3 mmoles, 1 equiv.), TFA (15 mL, 5 mL/mmole) was added at room temperature. The resulting solution was stirred for 18 h at 60 °C and was then allowed to cool to room temperature. The reaction mixture was diluted with DCM (20 mL), transferred into an Erlenmeyer flask, and placed in an ice bath. A saturated aq. Na_2_CO_3_ solution (50 mL) was added portionwise under vigorous stirring, and after the effervescence subsided, the mixture was transferred into a separatory funnel. The organic layer was separated, and the aqueous one was extracted with DCM (2 × 30 mL). The organic layers were combined, washed with saturated aq. Na_2_CO_3_ solution (50 mL) and saturated brine (50 mL), and dried over anhydrous Na_2_SO_4_. The solvent was removed in vacuo, and the crude mixture was purified through flash column chromatography (silica gel, *n*-hexane/EtOAc 15:1 to 1:2 *v*/*v*) to give nitrile **22**, benzamide **23,** and in some cases, isoxazoline/isoquinolinone hybrid **11**. Oxime **29** was also isolated from the reaction mixture of **13j**. All characterization and spectroscopic data for compounds **11**, **22**, **23**, and **29** are given in the Supplementary Materials.

### 3.8. Synthesis of Compounds ***23***, ***30***, and ***10***

The preparation of compounds **23**, **30,** and **10** was previously reported by us [17].

### 3.9. General Procedure for the Hydrolysis of Nitrile ***22a***

To a round-bottom flask containing the nitrile **22a** (1 mmoles, 1 equiv.), the reagents shown in Table 3 were added at room temperature (5 mL/mmole of 96% H_2_SO_4_ or TFA were used). The resulting solution was stirred for 18 h at 60 °C and was then allowed to cool to room temperature. The reaction mixture was diluted with DCM (10 mL), transferred into an Erlenmeyer flask, and placed in an ice bath. A saturated aq. Na_2_CO_3_ solution (20 mL) was added portionwise under vigorous stirring, and after effervescence subsided, the mixture was transferred into a separatory funnel. The organic layer was separated, and the aqueous one was extracted with DCM (2 × 10 mL). The organic layers were combined, washed with saturated aq. Na_2_CO_3_ solution (20 mL) and saturated brine (20 mL), and dried over anhydrous Na_2_SO_4_. The solvent was removed in vacuo, and the crude mixture was purified through flash column chromatography (silica gel, *n*-hexane/EtOAc 15:1 to 1:2 *v*/*v*) to give benzamide **23a** (Table 3, entries 1 and 3), whereas the starting material was fully recovered for entry 2.

### 3.10. General Procedure for the DDQ-Mediated Synthesis of Isoxazoline Isoquinolinone Hybrid ***11***

Wet DCM was prepared by adding equal volumes (1:1 *v*/*v*) of DCM and H_2_O into a separatory funnel. The funnel was sealed and shaken vigorously for at least 5 min, after which the two layers were left to separate. The organic layer was then collected and used as such. To a round bottom flask containing isoxazolidine isoquinolinone **10a** or **10b** (0.05 mmoles, 1 equiv.), wet DCM (100 mL/mmole) was added at room temperature. Subsequently, DDQ (57 mg, 0.25 mmoles, 5 equiv.) was added in a single portion, and the red-brownish mixture was stirred for 24 h at the same temperature. The reaction mixture was then diluted with DCM (5 mL), quenched through the addition of H_2_O (10 mL), and transferred into a separatory funnel. The organic layer was separated, and the aqueous one was extracted with DCM (2 × 10 mL). The organic layers were combined, washed with saturated aq. Na_2_CO_3_ solution (30 mL) and saturated brine (30 mL), and dried over anhydrous Na_2_SO_4_. The solvent was removed in vacuo, and the crude mixture was purified through flash column chromatography (silica gel, *n*-hexane/EtOAc 6:1 to 1:1 *v*/*v*) to give isoxazoline isoquinolinone hybrid **11**. All characterization and spectroscopic data for compound **11** are given in the Supplementary Materials.

### 3.11. In Vitro Antifungal Assay

The antifungal activity of compounds was determined by the modified microdilution method [39,40]. The following fungal strains were used: *Aspergillus fumigatus* (clinical isolate), *Aspergillus niger* (ATCC 6275), *Penicillium funiculosum* (ATCC 36839), *Penicillium chrysogenum* (2761 isolate from Lomonosov Moscow State University), *Candida albicans* (ATCC 10231), and *Candida krusei* (ATCC 14243). All the tested microorganisms are deposited at the Mycological Laboratory, Department of Plant Physiology, Institute for Biological Research ‘Siniša Stankovic’—National Institute of Republic of Serbia, University of Belgrade, Serbia. Prior to the antifungal assay, fungi were cultured for 14 days on solid malt agar at 25 °C, after which the spore inoculum was prepared. Spores were washed using sterile saline and 0.1% Tween 80 (*v*/*v*), and the suspension was adjusted to a concentration of approximately 1.0 × 10^5^ CFU in a final volume of 100 µL per well by microscopic enumeration with a cell-counting hemotocytometer (Neubauer chamber; Paul Marienfeld, Frimley, UK). The inocula were stored at 4 °C until further use. The obtained results were presented as minimal inhibitory (MIC) and minimal fungicidal concentrations (MFCs) needed to effectively retard fungal growth. Commercially available antifungal agent ketoconazole (K1003, Sigma, Deisenhofen, Germany) was used as a positive control. Five percent DMSO was used as a negative control. Experiments were repeated twice.

### 3.12. In Vitro 2D Checkerboard Assay

The interactions (synergism, additive effect, indifference, or antagonism) between the selected compounds (compounds that showed the best inhibitory effect on the tested fungi) and the antifungal agent (ketoconazole) in combinations on the growth of a panel of micromycetes were analyzed using a two-dimensional checkerboard with two-fold dilution. The MICs obtained were used as a starting point for further tests. For this test, 96-well microtiter plates containing malt broth growth medium combined with the tested compound and antimicrobial agent at concentrations ranging from 1/8 MIC to 2 MIC [41] in a checkerboard pattern were used. An untreated inoculum served as a control. The plates were incubated at 25 °C for 72 h.

The fractional inhibitory concentration index (FICI) was determined according to the following formula: FICI = (FIC1°/MIC1°) + (FIC2°/MIC2°).

FIC1° and FIC2° are the MIC values of the tested compounds and antibiotics in combination, while MIC1° and MIC2° are the MIC values of the individual activity of the selected agents. The results were interpreted as follows: FICI 0.5 = synergistic effect, FICI > 0.5 = additive effect, FICI > 2 = indifferent effect, and FICI > 4 = antagonistic effect. Experiments were repeated twice [42].

## 4. Conclusions

Herein, we presented our investigation of the synthesis of novel isoxazoline isoquinolinone hybrids. According to our original plan, those fused heterocyclic compounds could be prepared via a 1,3-dipolar cycloaddition of indenone with nitrile oxides and a subsequent Beckmann rearrangement of the tosyl oximes of the corresponding adducts. The cycloaddition with nitrile oxides occurred uneventfully and was found to be regioselective. The major adducts were transformed into the required tosyl oximes, and the Beckmann rearrangement wasthen attempted. However, the applied conditions failed to give the desired isoquinolinones, favoring instead the formation of the fragmentation products (isoxazole benzonitriles) and the related hydrolysis products (isoxazole benzamides). A mechanistic description of this outcome is also given. An alternative successful approach was adopted involving the preparation of fused isoxazoline isoquinolinone hybrids via DDQ-mediated oxidation of the corresponding isoxazolidine isoquinolinones. The synthesis of isoxazoline derivatives completes the diverse libraries of isoxazole-like benzamides and isoquinolinones that we had previously described. Representative compounds of those libraries (i.e., isoxazole and 4-iodoisoxazole benzamides, as well as isoxazoline and isoxazolidine isoquinolinones) were evaluated for their antifungal potential. In some cases, depending on the strain used and the compounds employed, very interesting activity was observed. Further examination of this activity using the 2D checkerboard methodology revealed that some compounds exhibit significant additive and synergistic effects when combined with ketoconazole. The overall synthetic schemes represent versatile methodologies for the expansion of those libraries in the future, which could clarify their antifungal behavior and determine new biological targets.

## Figures and Tables

**Table 1:** Synthesis of isoxazoline adducts **14** and **21** from various nitrile oxides.

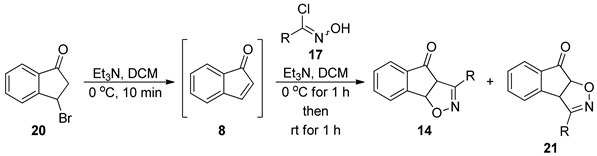				
**Entry**	**R =**	**14** **(Major Cycloadduct)**	**21** **(Minor Cycloadduct)**	**Yield ^1^** **(Comb.)**
1	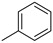	88% (**14a**)	6% (**21a**)	94%
2	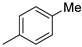	82% (**14b**)	7% (**21b**)	89%
3	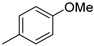	86% (**14c**)	5% (**21c**)	91%
4	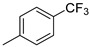	85% (**14d**)	4% (**21d**)	89%
5	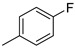	88% (**14e**)	5% (**21e**)	93%
6	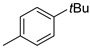	83% (**14f**)	6% (**21f**)	89%
7	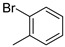	84% (**14g**)	6% (**21g**)	90%
8	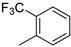	87% (**14h**)	3% (**21h**)	90%
9	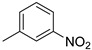	89% (**14i**)	3% (**21i**)	92%
10	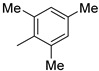	87% (**14j**)	3% (**21j**)	90%
11	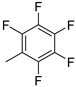	80% (**14k**)	-	80%
12	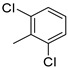	89% (**14l**)	4% (**21l**)	93%
13	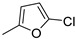	70% (**14m**)	-	70%
14	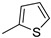	64% (**14n**)	-	64%
15	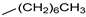	92% (**14o**)	-	92%

^1^ Yield after purification by flash column chromatography based on the amount of **20** used.

**Table 2:** Expanding the Beckmann protocol to a wider panel of tosyl oximes.

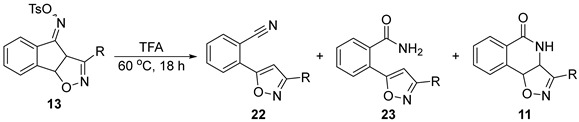					
**Entry**	**R =**	**22**	**23**	**11**	**Yield ^1^** **(Comb.)**
1	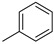	80% (**22a**)	10% (**23a**)	-	80%
2	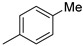	76% (**22b**)	9% (**23b**)	-	85%
3	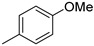	75% (**22c**)	13% (**23c**)	-	88%
4	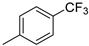	72% (**22d**)	15% (**23d**)	-	87%
5	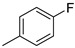	81% (**22e**)	8% (**23e**)	-	89%
6	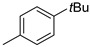	85% (**22f**)	6% (**23f**)	-	91%
7	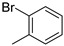	57% (**22g**)	12% (**23g**)	10% (**11g**)	79%
8	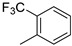	59% (**22h**)	22% (**23h**)	11% (**11h**)	92%
9	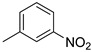	86% (**22i**)	-	-	86%
10	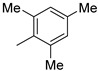	12% (**22j**)	-	-	77% ^2^
11	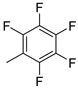	54% (**22k**)	29% (**23k**)	13% (**11k**)	96%
12	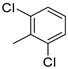	53% (**22l**)	32% (**23l**)	6% (**11l**)	91%
13	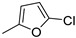	71% (**22m**)	12% (**23m**)	-	83%
14	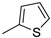	70% (**22n**)	9% (**23n**)	-	79%
15	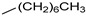	82% (**22o**)	9% (**23o**)	-	91%

^1^ Yield after purification by flash column chromatography; ^2^ the main product was **29** (yield 65%), as shown in Scheme 10.

**Table 3:** Control experiments for the hydrolysis of nitrile **22a**.

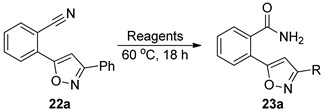		
**Entry**	**Reagents**	**Yield ^1^**
1	96% H_2_SO_4_	97%
2	TFA, H_2_O (1 equiv.)	0%
3	TFA, *p*-TsOH (1 equiv.), H_2_O (1 equiv.)	9%

^1^ Yield after purification by flash column chromatography.

**Table 4:** Synthesis of isoxazoline/isoquinolinone hybrids via oxidation.

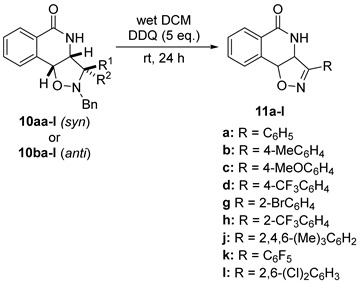					
**Entry**	**SM ^1^**	**R^1^ =**	**R^2^ =**	**Product ^2^**	**BRSM** **Yield ^3^**
1	**10aa**	H	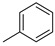	45% (**11a**)	97%
2	**10ba**	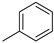	H	48% (**11a**)	96%
3	**10ab**	H	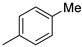	44% (**11b**)	94%
4	**10bb**	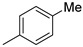	H	47% (**11b**)	92%
5	**10ac**	H	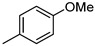	53% (**11c**)	91%
6	**10bc**	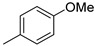	H	50% (**11c**)	95%
7	**10ad**	H	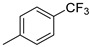	42% (**11d**)	90%
8	**10bd**	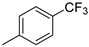	H	41% (**11d**)	89%
9	**10ag**	H	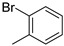	48% (**11g**)	93%
10	**10bg**	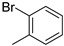	H	51% (**11g**)	97%
11	**10ah**	H	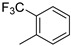	48% (**11h**)	93%
12	**10bh**	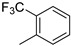	H	45% (**11h**)	95%
13	**10aj**	H	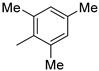	75% (**11j**)	93%
14	**10ak**	H	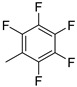	46% (**11k**)	96%
15	**10bk**	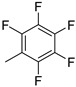	H	48% (**11k**)	93%
16	**10al**	H	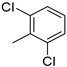	49% (**11l**)	95%
17	**10bl**	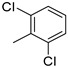	H	49% (**11l**)	92%

^1^ SM = starting material; ^2^ yield after purification by flash column chromatography; ^3^ BRSM yield is the yield based on recovered starting material.

**Table 5:** Antifungal activity of isoxazole benzamides **23** and **30** (MIC/MFC in mg/mL).

Compound ^1^		*C. albicans*	*C. krusei*	*A. fumigatus*	*A. niger*	*P. funiculosum*	*P. chrysogenum*
**23a**	MIC	0.08	0.16	0.24	0.16	0.08	0.16
	MFC	0.16	0.24	0.32	0.24	0.16	0.24
**23b**	MIC	-	-	0.16	0.08	0.04	0.08
	MFC	-	-	0.24	0.16	0.08	0.16
**23c**	MIC	-	0.16	0.16	0.08	0.04	0.16
	MFC	-	0.24	0.24	0.16	0.08	0.24
**23d**	MIC	-	0.08	0.24	0.04	0.04	0.08
	MFC	-	0.16	0.32	0.08	0.08	0.16
**23e**	MIC	-	0.16	0.16	0.16	0.16	-
	MFC	-	0.24	0.24	0.24	0.32	-
**23f**	MIC	-	0.16	0.16	0.04	0.04	0.16
	MFC	-	0.24	0.24	0.16	0.08	0.24
**23g**	MIC	-	0.16	0.16	0.08	0.16	0.16
	MFC		0.24	0.24	0.16	0.32	0.24
**23h**	MIC	0.16	0.16	0.16	0.16	0.08	0.16
	MFC	0.24	0.24	-	0.32	0.16	0.24
**23i**	MIC	0.16	0.16	0.16	0.16	0.16	-
	MFC	0.24	0.24	0.24	0.32	0.24	-
**23j**	MIC	0.08	0.16	0.08	0.04	0.04	0.08
	MFC	0.16	0.24	0.24	0.16	0.08	0.16
**23k**	MIC	0.24	-	0.16	0.16	0.04	0.08
	MFC	0.32	-	0.24	0.24	0.08	0.16
**23l**	MIC	0.08	0.08	0.24	0.04	0.16	0.16
	MFC	-	0.16	0.32	0.08	0.24	0.24
**23m**	MIC	0.08	0.16	0.08	0.08	0.04	0.04
	MFC	0.16	0.24	0.16	0.16	0.08	0.08
**23n**	MIC	0.16	-	0.16	0.16	0.04	0.16
	MFC	0.24	-	0.24	0.24	0.16	0.24
**23o**	MIC	-	0.24	0.24	0.08	0.08	0.16
	MFC	-	0.32	0.32	0.16	0.16	0.24
**30a**	MIC	0.16	0.08	0.12	0.04	0.16	0.08
	MFC	0.32	0.16	0.16	0.08	0.32	0.16
**30b**	MIC	0.16	0.16	0.16	0.02	0.08	0.16
	MFC	0.32	0.32	0.24	0.04	0.16	0.32
**30c**	MIC	0.16	0.16	0.16	0.04	0.08	0.08
	MFC	0.32	0.32	0.32	0.08	0.16	0.16
**30d**	MIC	0.16	0.08	0.16	0.04	0.16	0.16
	MFC	0.32	0.24	0.24	0.08	0.24	0.32
K ^2^	MIC	0.0125	0.0004	0.025	0.10	0.0125	0.05
	MFC	0.025	0.0008	0.05	0.20	0.025	0.10

^1^ Means no activity was observed; ^2^ K = ketoconazole.

**Table 6:** Antifungal activity of selected isoxazoline isoquinolinones and isoxazolidine isoquinolinones (MIC/MFC in mg/mL).

Compound ^1^		*C. albicans*	*C. krusei*	*A. fumigatus*	*A. niger*	*P. funiculosum*	*P. chrysogenum*
**11g**	MIC	0.08	0.16	0.16	0.08	0.16	0.16
	MFC	0.16	0.32	0.24	0.16	0.32	0.24
**11h**	MIC	0.16	0.16	0.16	0.16	0.16	0.16
	MFC	0.32	0.32	0.32	0.32	0.32	0.32
**11k**	MIC	0.16	0.08	0.08	0.04	0.04	0.08
	MFC	0.32	0.16	0.16	0.08	0.08	0.16
**11l**	MIC	0.16	0.16	0.16	0.01	0.04	0.16
	MFC	0.32	0.32	0.32	0.02	0.08	0.32
**10ad**	MIC	0.08	0.16	0.16	0.08	0.16	0.16
	MFC	0.16	0.32	0.32	0.16	0.32	0.24
**10bd**	MIC	0.16	0.16	0.16	0.04	0.16	0.08
	MFC	0.32	0.32	0.32	0.08	0.24	0.16
**10aj**	MIC	0.16	0.16	0.16	0.02	0.16	0.04
	MFC	0.32	0.32	0.32	0.04	0.24	0.08
**10al**	MIC	0.16	0.08	0.16	0.01	0.16	0.16
	MFC	0.32	0.24	0.32	0.02	0.32	0.32
**10am**	MIC	0.16	0.16	0.12	0.02	0.08	0.16
	MFC	0.32	0.32	0.24	0.04	0.16	0.32
**10bm**	MIC	0.16	0.16	0.16	0.005	0.04	0.16
	MFC	0.32	0.32	0.32	0.01	0.08	0.32
K ^2^	MIC	0.0125	0.0004	0.025	0.10	0.0125	0.05
	MFC	0.025	0.0008	0.05	0.20	0.025	0.10

^1^ Means no activity was observed; ^2^ K = ketoconazole.

## Data Availability

All data supporting the findings of this study are available within the paper and within its Supplementary Materials published online.

## Supplementary Materials

The following supporting information can be downloaded at https://www.mdpi.com/1420-3049/30/3/589/s1. It contains data for compounds **11**, **13**, **14**, **16**, **21**–**23**, and **29** and copies of ^1^H, ^13^C, ^19^F, and 2D NMR spectra of all new compounds.
