# Supplementary material for: Studies of the Synthesis of Fused Isoxazoline/Isoquinolinones and Evaluation of the Antifungal Activity of Isoxazole-like Benzamide and Isoquinolinone Hybrids
Source: Molecules. 2025 Jan 27;30(3):589. doi: 10.3390/molecules30030589 (PMC12128697; doi:10.3390/molecules30030589)
Supplement: Supplementary file 1 [file molecules-30-00589-s001.zip › molecules-3424562-supplementary.pdf]

Studies towards the synthesis of fused isoxazoline/isoquinolinones and evaluation of the antifungal activity of isoxazole-like benzamide and isoquinolinone hybrids

Konstantinos A. Ouzounthanasis [1], Jasmina Glamočlija [2], Ana Ćirić [2] and Alexandros E. Koumbis [1,*]

[1]Laboratory of Organic Chemistry, Department of Chemistry, Aristotle University of Thessaloniki, 54124 Thessaloniki, Greece
[2]Department of Plant Physiology, Institute for Biological Research "Siniša Stanković" - National Institute of Republic of Serbia, University of Belgrade, 11060 Belgrade, Serbia

*akoumbis@chem.auth.gr

SUPPORTING INFORMATION

Table of Contents

1. Data for compounds **16**…..……………………………………………………………………S2
2. Data for compounds **14** and **21**…………………………………………………………………S2
3. Data for compounds **13**………………………………………………….……………………S9
4. Data for compounds **22**….…………………………………………….………………….………S14
5. Data for compounds **23**……………………..…………………….………………………...S17
6. Data for compound **29**……………………………………………………………….………S18
7. Data for compounds **11**…………………………………………………………………………S18
8. Copies of ^{1}H, ^{13}C, ^{19}F and 2D NMR Spectra.……………..……………………………………S21

Data for compounds **16**

2-Benzylisoquinolin-1(2*H*)-one (**16a**)

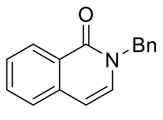

Yield: 98%; white solid; $\boldsymbol{R}_\mathbf{f}$ = 0.31 (*n*-hexane/EtOAc 10:1 *v*/*v*); **^{1}H NMR** (500 MHz, $CDCl_3$, 25 °C): δ = 8.47 (d, *J* = 8.1 Hz, 1H), 7.64 (td, *J* = 7.3, 1.1 Hz, 1H), 7.52 – 7.48 (m, 2H), 7.34 – 7.27 (m, 5H), 7.09 (d, *J* = 7.3 Hz, 1H), 6.49 (d, *J* = 7.4 Hz, 1H), 5.23 (s, 2H) ppm. All spectroscopic data were in accordance with those reported in the literature [1].

1-Oxoisoquinoline-2(1*H*)-carboxylate (**16b**)

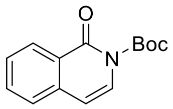

Yield: 91%; yellowish oil; $\boldsymbol{R}_\mathbf{f}$ = 0.44 (*n*-hexane/EtOAc 10:1 *v*/*v*); **^{1}H NMR** (500 MHz, $CDCl_3$, 25 °C): δ = 8.41 (dd, *J* = 7.9, 1.1 Hz, 1H), 7.63 (td, *J* = 7.5, 1.3 Hz, 1H), 7.50 (d, *J* = 7.9 Hz, 1H), 7.48 – 7.42 (m, 2H), 6.42 (d, *J* = 7.9 Hz, 1H), 1.65 (s, 9H). ppm; **^{13}C NMR** (125 MHz, $CDCl_3$, 25 °C): δ = 161.0, 151.1, 136.3, 133.3, 128.7, 127.4, 127.29, 127.26, 125.9, 106.7, 85.5, 27.8 ppm; **FT-IR** (neat): ν = 3062, 2946, 1732, 1647, 1620, 1600, 1456, 1365, 782, 689 cm^{-1}; **HRMS** (ESI), *m*/*z*: $[M + Na]^+$ calcd for $C_{14}H_{15}NNaO_3^+$ 268.0944; found 268.0946.

Data for compounds **14** and **21**

3-Phenyl-3a,8b-dihydro-4*H*-indeno[2,1-*d*]isoxazol-4-one (**14a**)

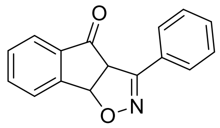

Yield: 88%; white solid; $\boldsymbol{R}_\mathbf{f}$ = 0.31 (*n*-hexane/EtOAc 4:1 *v*/*v*); **^{1}H NMR** (500 MHz, $CDCl_3$, 25 °C): δ = 8.03 – 7.96 (m, 2H), 7.82 (d, *J* = 7.7 Hz, 1H), 7.79 – 7.73 (m, 2H), 7.55 (t, *J* = 7.4 Hz, 1H), 7.46 – 7.41 (m, 3H), 6.31 (d, *J* = 8.3 Hz, 1H), 4.75 (d, *J* = 8.3 Hz, 1H) ppm. All spectroscopic data were in accordance with those reported in the literature [2].

3-Phenyl-3a,8a-dihydro-8*H*-indeno[1,2-*d*]isoxazol-8-one (**21a**)

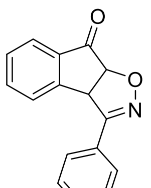

Yield: 6%; white solid; $\boldsymbol{R}_\mathbf{f}$ = 0.18 (*n*-hexane/EtOAc 4:1 *v*/*v*); **^{1}H NMR** (500 MHz, $CDCl_3$, 25 °C): δ = 7.85 (d, *J* = 7.7 Hz, 1H), 7.83 – 7.79 (m, 2H), 7.53 (td, *J* = 7.5, 1.3 Hz, 1H), 7.50 – 7.47 (m, 3H), 7.43 (t, *J* = 7.9 Hz, 2H), 5.42 (d, *J* = 8.6 Hz, 1H), 5.33 (d, *J* = 8.6 Hz, 1H) ppm. All spectroscopic data were in accordance with those reported in the literature [2].

1 Yang, C.; Zhang, G.; Tang, S.; Pan, Y.; Shao, H.; Jiao, W. Dess–Martin Periodinane-Mediated Oxidative Coupling Reaction of Isoquinoline with Benzyl Bromide. *Molecules* **2023**, *28*, 923.

2 Ouzounthanasis, K. A.; Rizos, S. R.; Koumbis, A. E. A Convenient Synthesis of Novel Isoxazolidine and Isoxazole Isoquinolinones Fused Hybrids. *Molecules* **2024**, *29*, 91.

3-(*p*-Tolyl)-3a,8b-dihydro-4*H*-indeno[2,1-*d*]isoxazol-4-one (**14b**)

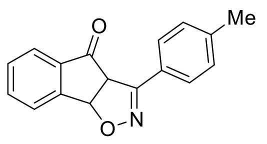

Yield: 82%; white solid; **m.p.** 147–148 °C; ***R*f** = 0.54 (*n*-hexane/EtOAc 2:1 *v/v*); **^{1}H NMR** (500 MHz, $CDCl_3$, 25 °C): *δ* = 7.87 (d, *J* = 8.2 Hz, 2H), 7.81 (d, *J* = 7.7 Hz, 1H), 7.78 – 7.72 (m, 2H), 7.54 (t, *J* = 7.4 Hz, 1H), 7.24 (d, *J* = 8.0 Hz, 2H), 6.29 (d, *J* = 8.2 Hz, 1H), 4.74 (d, *J* = 8.3 Hz, 1H), 2.38 (s, 3H) ppm; **^{13}C NMR** (125 MHz, $CDCl_3$, 25 °C): *δ* = 197.4, 152.5, 150.7, 140.7, 136.2, 134.4, 130.5, 129.3, 127.8, 126.9, 125.1, 124.2, 82.7, 60.6, 21.5 ppm; **FT-IR** (neat): ν = 2945, 1722, 1602, 1334, 1269, 902, 839, 762 cm^{-1}; **HRMS** (ESI), *m*/*z*: $[M + Na]^+$ calcd for $C_{17}H_{13}NNaO_2^+$ 286.0838; found 286.0841.

3-(*p*-Tolyl)-3a,8a-dihydro-8*H*-indeno[1,2-*d*]isoxazol-8-one (**21b**)

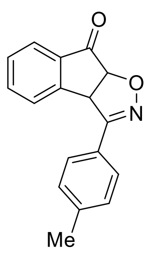

Yield: 7%; white solid; **m.p.** 189–190 °C; ***R*f** = 0.46 (*n*-hexane/EtOAc 2:1 *v/v*); **^{1}H NMR** (500 MHz, $CDCl_3$, 25 °C): *δ* = 7.83 (d, *J* = 7.2 Hz, 1H), 7.69 (d, *J* = 8.0 Hz, 2H), 7.52 (t, *J* = 7.5 Hz, 1H), 7.45 – 7.39 (m, 2H), 7.28 (d, *J* = 8.0 Hz, 2H), 5.39 (d, *J* = 8.6 Hz, 1H), 5.30 (d, *J* = 8.6 Hz, 1H), 2.41 (s, 3H) ppm; **^{13}C NMR** (125 MHz, $CDCl_3$, 25 °C): *δ* = 198.9, 156.9, 150.4, 140.9, 136.0, 134.3, 129.7, 129.2, 127.5, 126.2, 125.6, 125.0, 84.5, 53.1, 21.4 ppm; **FT-IR** (neat): ν = 3034, 2965, 1717, 1583, 1468, 1339 cm^{-1}; **HRMS** (ESI), *m*/*z*: $[M + Na]^+$ calcd for $C_{17}H_{13}NNaO_2^+$ 286.0838; found 286.0836.

3-(4-Methoxyphenyl)-3a,8b-dihydro-4*H*-indeno[2,1-*d*]isoxazol-4-one (**14c**)

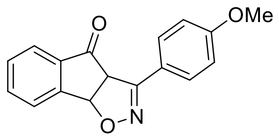

Yield: 86%; white solid; **m.p.** 164–165 °C; ***R*f** = 0.47 (*n*-hexane/EtOAc 2:1 *v/v*); **^{1}H NMR** (500 MHz, $CDCl_3$, 25 °C): *δ* = 7.95 – 7.92 (m, 2H), 7.80 (d, *J* = 7.6 Hz, 1H), 7.77 – 7.72 (m, 2H), 7.53 (t, *J* = 7.4 Hz, 1H), 6.96 – 6.93 (m, 2H), 6.26 (d, *J* = 8.3 Hz, 1H), 4.71 (d, *J* = 8.3 Hz, 1H), 3.84 (s, 3H) ppm; **^{13}C NMR** (125 MHz, $CDCl_3$, 25 °C): *δ* = 197.5, 161.2, 152.1, 150.8, 136.2, 134.4, 130.5, 129.5, 126.9, 124.2, 120.5, 114.0, 82.6, 60.8, 55.3 ppm; **FT-IR** (neat): ν = 3046, 2967, 2836, 1720, 1679, 1510, 1246, 1178, 848, 766 cm^{-1}; **HRMS** (ESI), *m*/*z*: $[M + Na]^+$ calcd for $C_{17}H_{13}NNaO_3^+$ 302.0788; found 302.0792.

3-(4-Methoxyphenyl)-3a,8a-dihydro-8*H*-indeno[1,2-*d*]isoxazol-8-one (**21c**)

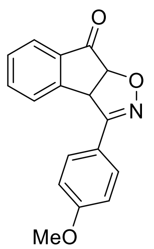

Yield: 5%; white solid; **m.p.** 139–140 °C; ***R*f** = 0.24 (*n*-hexane/EtOAc 2:1 *v/v*); **^{1}H NMR** (500 MHz, $CDCl_3$, 25 °C): *δ* = 7.84 (d, *J* = 7.0 Hz, 1H), 7.77 – 7.74 (m, 2H), 7.53 (td, *J* = 7.3, 1.3 Hz, 1H), 7.43 (t, *J* = 7.4 Hz, 2H), 7.02 – 6.98 (m, 2H), 5.37 (d, *J* = 8.5 Hz, 1H), 5.30 (d, *J* = 8.5 Hz, 1H), 3.87 (s, 3H) ppm; **^{13}C NMR** (125 MHz, $CDCl_3$, 25 °C): *δ* = 198.8, 161.4, 156.5, 150.5, 136.0, 134.4, 129.2, 129.1, 126.2, 125.7, 120.3, 114.4, 84.5, 55.4, 53.3 ppm; **FT-IR** (neat): ν = 3047, 2964, 2842, 1724, 1608, 1515, 1249, 896, 830 cm^{-1}; **HRMS** (ESI), *m*/*z*: $[M + Na]^+$ calcd for $C_{17}H_{13}NNaO_3^+$ 302.0788; found 302.0793.

3-(4-(Trifluoromethyl)phenyl)-3a,8b-dihydro-4*H*-indeno[2,1-*d*]isoxazol-4-one (**14d**)

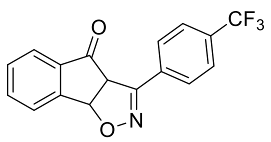

Yield: 85%; white solid; **m.p.** 155–156 °C; R_f = 0.50 (*n*-hexane/EtOAc 2:1 *v/v*); **^{1}H NMR** (500 MHz, $CDCl_3$, 25 °C): δ = 8.12 (d, *J* = 8.1 Hz, 2H), 7.84 – 7.74 (m, 3H), 7.68 (d, *J* = 8.2 Hz, 2H), 7.56 (t, *J* = 7.4 Hz, 1H), 6.36 (d, *J* = 8.3 Hz, 1H), 4.74 (d, *J* = 8.4 Hz, 1H) ppm; **^{13}C NMR** (125 MHz, $CDCl_3$, 25 °C): δ = 197.0, 151.6, 150.4, 136.5, 134.3, 131.9 (q, *J* = 32.6 Hz, 1C), 131.4 (d, *J* = 1.5 Hz, 1C), 130.8, 128.2, 127.0, 125.5 (q, *J* = 3.9 Hz, 1C), 124.4, 123.8 (q, *J* = 272.3 Hz, 1C), 83.5, 60.1 ppm; **^{19}F NMR** (470 MHz, $CDCl_3$, 25 °C): δ = -63.00 (s, 3F) ppm; **FT-IR** (neat): ν = 3083, 2951, 1716, 1603, 1324, 1166, 1106, 1070, 856, 761 cm^{-1}; **HRMS** (ESI), *m*/*z*: $[M + Na]^+$ calcd for $C_{17}H_{10}F_3NNaO_2^+$ 340.0556; found 340.0551.

3-(4-(Trifluoromethyl)phenyl)-3a,8a-dihydro-8*H*-indeno[1,2-*d*]isoxazol-8-one (**21d**)

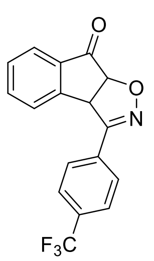

Yield: 4%; white solid; **m.p.** 180–181 °C; R_f = 0.40 (*n*-hexane/EtOAc 2:1 *v/v*); **^{1}H NMR** (500 MHz, $CDCl_3$, 25 °C): δ = 7.94 (d, *J* = 8.1 Hz, 2H), 7.86 (d, *J* = 7.6 Hz, 1H), 7.74 (d, *J* = 8.2 Hz, 2H), 7.56 (td, *J* = 7.5, 1.3 Hz, 1H), 7.46 (t, *J* = 7.5 Hz, 1H), 7.40 (d, *J* = 7.8 Hz, 1H), 5.45 (d, *J* = 8.7 Hz, 1H), 5.40 (d, *J* = 8.7 Hz, 1H) ppm; **^{13}C NMR** (125 MHz, $CDCl_3$, 25 °C): δ = 198.3, 156.0, 149.9, 136.3, 134.4, 132.3 (q, *J* = 32.9 Hz, 1C), 131.6 (d, *J* = 1.5 Hz, 1C), 129.5, 127.8, 126.1, 126.0 (q, *J* = 3.8 Hz, 1C), 125.9, 123.6 (q, *J* = 272.4 Hz, 1C), 85.2, 52.7 ppm; **^{19}F NMR** (470 MHz, $CDCl_3$, 25 °C): δ = -62.93 (s, 3F) ppm; **FT-IR** (neat): ν = 2964, 1720, 1470, 1326, 1113, 1071, 884, 770 cm^{-1}; **HRMS** (ESI), *m*/*z*: $[M + Na]^+$ calcd for $C_{17}H_{10}F_3NNaO_2^+$ 340.0556; found 340.0550.

3-(4-Fluorophenyl)-3a,8b-dihydro-4*H*-indeno[2,1-*d*]isoxazol-4-one (**14e**)

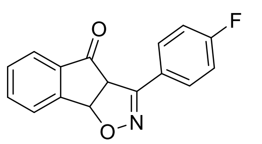

Yield: 88%; white solid; **m.p.** 150–151 °C; R_f = 0.51 (*n*-hexane/EtOAc 2:1 *v/v*); **^{1}H NMR** (500 MHz, $CDCl_3$, 25 °C): δ = 8.02 – 7.97 (m, 2H), 7.82 – 7.73 (m, 3H), 7.55 (t, *J* = 7.4 Hz, 1H), 7.14 – 7.08 (m, 2H), 6.30 (d, *J* = 8.3 Hz, 1H), 4.70 (d, *J* = 8.3 Hz, 1H) ppm; **^{13}C NMR** (125 MHz, $CDCl_3$, 25 °C): δ = 197.2, 163.9 (d, *J* = 251.3 Hz, 1C), 151.6 (d, *J* = 0.9 Hz, 1C), 150.6, 136.3, 134.3, 130.6, 130.0 (d, *J* = 8.6 Hz, 1C), 126.9, 124.3, 124.2, 115.7 (d, *J* = 21.9 Hz, 1C), 83.0, 60.5 ppm; **^{19}F NMR** (470 MHz, $CDCl_3$, 25 °C): δ = -109.39 – -109.45 (m, 1F) ppm; **FT-IR** (neat): ν = 3092, 1717, 1600, 1510, 1228, 911, 841, 759 cm^{-1}; **HRMS** (ESI), *m*/*z*: $[M + Na]^+$ calcd for $C_{16}H_{10}FNNaO_2^+$ 290.0588; found 290.0591.

3-(4-Fluorophenyl)-3a,8a-dihydro-8*H*-indeno[1,2-*d*]isoxazol-8-one (**21e**)

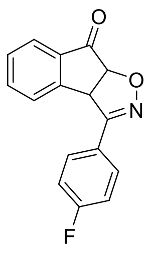

Yield: 5%; white solid; **m.p.** 163–164 °C; R_f = 0.40 (*n*-hexane/EtOAc 2:1 *v/v*); **^{1}H NMR** (500 MHz, $CDCl_3$, 25 °C): δ = 7.85 (d, *J* = 7.6 Hz, 1H), 7.83 – 7.78 (m, 2H), 7.55 (td, *J* = 7.5, 1.3 Hz, 1H), 7.45 (t, *J* = 7.4 Hz, 1H), 7.39 (dd, *J* = 7.8, 0.9 Hz, 1H), 7.21 – 7.15 (m, 2H), 5.39 (d, *J* = 8.6 Hz, 1H), 5.34 (d, *J* = 8.6 Hz, 1H) ppm; **^{13}C NMR** (125 MHz, $CDCl_3$, 25 °C): δ = 198.6, 164.0 (d, *J* = 251.8 Hz, 1C), 156.0, 150.1, 136.1, 134.4, 129.5 (d, *J* = 8.6 Hz, 1C), 129.4, 126.1, 125.8, 124.2 (d, *J* = 3.4 Hz, 1C), 116.2 (d, *J* = 22.4 Hz, 1C), 84.8, 53.1 ppm; **^{19}F NMR** (470 MHz, $CDCl_3$, 25 °C): δ = -108.80 – -108.87 (m, 1F) ppm; **FT-IR** (neat): ν = 3092, 2929, 1728, 1602, 1512, 1337, 1229, 881, 840, 771 cm^{-1}; **HRMS** (ESI), *m*/*z*: $[M + Na]^+$ calcd for $C_{16}H_{10}FNNaO_2^+$ 290.0588; found 290.0590

3-(4-(*tert*-Butyl)phenyl)-3a,8b-dihydro-4*H*-indeno[2,1-*d*]isoxazol-4-one (**14f**)

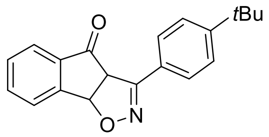

Yield: 83%; white solid; **m.p.** 130–131 °C; R_f = 0.51 (*n*-hexane/EtOAc 3:1 *v/v*); **^{1}H NMR** (500 MHz, $CDCl_3$, 25 °C): δ = 7.93 (d, *J* = 8.5 Hz, 2H), 7.81 (d, *J* = 7.7 Hz, 1H), 7.77 – 7.72 (m, 2H), 7.53 (t, *J* = 7.4 Hz, 1H), 7.46 (d, *J* = 8.5 Hz, 2H), 6.28 (d, *J* = 8.3 Hz, 1H), 4.74 (d, *J* = 8.3 Hz, 1H), 1.34 (s, 9H) ppm; **^{13}C NMR** (125 MHz, $CDCl_3$, 25 °C): δ = 197.4, 153.7, 152.4, 150.7, 136.2, 134.5, 130.5, 127.7, 126.9, 125.5, 125.1, 124.2, 82.76, 60.7, 34.8, 31.1 ppm; **FT-IR** (neat): ν = 3055, 2963, 1709, 1599, 1369, 1271, 906, 766 cm^{-1}; **HRMS** (ESI), *m*/*z*: $[M + Na]^+$ calcd for $C_{20}H_{19}NNaO_2^+$ 328.1308; found 328.1313.

3-(4-(*tert*-Butyl)phenyl)-3a,8a-dihydro-8*H*-indeno[1,2-*d*]isoxazol-8-one (**21f**)

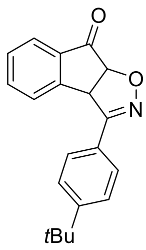

Yield: 6%; white solid; m.p. 195–196 °C; $\boldsymbol{R_f}$ = 0.37 (*n*-hexane/EtOAc 3:1 *v/v*); **^{1}H NMR** (500 MHz, $CDCl_3$, 25 °C): δ = 7.84 (d, *J* = 7.6 Hz, 1H), 7.75 (d, *J* = 8.5 Hz, 2H), 7.55 (td, *J* = 7.6, 1.2 Hz, 1H), 7.50 (d, *J* = 8.4 Hz, 2H), 7.48 – 7.41 (m, 2H), 5.40 (d, *J* = 8.6 Hz, 1H), 5.31 (d, *J* = 8.6 Hz, 1H), 1.36 (s, 9H) ppm; **^{13}C NMR** (125 MHz, $CDCl_3$, 25 °C): δ = 198.8, 156.7, 154.0, 150.4, 136.1, 134.4, 129.2, 127.3, 126.4, 125.9, 125.6, 125.0, 84.6, 53.1, 34.9, 31.1 ppm; **FT-IR** (neat): ν = 3043, 2955, 1721, 1599, 1468, 1342, 883, 763 cm^{-1}; **HRMS** (ESI), *m*/*z*: $[M + Na]^+$ calcd for $C_{20}H_{19}NNaO_2^+$ 328.1308; found 328.1314.

3-(2-Bromophenyl)-3a,8b-dihydro-4*H*-indeno[2,1-*d*]isoxazol-4-one (**14g**)

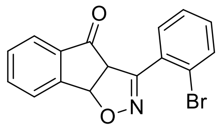

Yield: 84%; white solid; **m.p.** 130–131 °C; R_f = 0.46 (*n*-hexane/EtOAc 2:1 *v/v*); **^{1}H NMR** (500 MHz, $CDCl_3$, 25 °C): δ = 7.81 (d, *J* = 7.6 Hz, 1H), 7.78 – 7.73 (m, 2H), 7.67 – 7.64 (m, 1H), 7.55 (td, *J* = 7.5, 1.1 Hz, 1H), 7.34 – 7.26 (m, 3H), 6.31 (d, *J* = 8.3 Hz, 1H), 5.07 (d, *J* = 8.3 Hz, 1H) ppm; **^{13}C NMR** (125 MHz, $CDCl_3$, 25 °C): δ = 196.8, 153.7, 150.8, 136.2, 134.4, 133.4, 131.5, 131.2, 130.6, 129.2, 127.4, 127.0, 124.1, 122.3, 82.5, 62.0 ppm; **FT-IR** (neat): ν = 2962, 1721, 1463, 1261, 1199, 878, 761 cm^{-1}; **HRMS** (ESI), *m*/*z*: $[M + Na]^+$ calcd for $C_{16}H_{10}BrNNaO_2^+$ 349.9787; found 349.9780

3-(2-Bromophenyl)-3a,8a-dihydro-8*H*-indeno[1,2-*d*]isoxazol-8-one (**21g**)

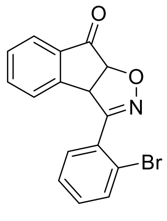

Yield: 6%; white solid; **m.p.** 120–122 °C; $\boldsymbol{R_f}$ = 0.43 (*n*-hexane/EtOAc 2:1 *v/v*); **^{1}H NMR** (500 MHz, $CDCl_3$, 25 °C): δ = 7.85 (d, *J* = 7.6 Hz, 1H), 7.70 (d, *J* = 7.9 Hz, 1H), 7.49 (t, *J* = 7.4 Hz, 1H), 7.43 (t, *J* = 7.4 Hz, 1H), 7.35 – 7.27 (m, 2H), 7.16 (dd, *J* = 7.5, 1.8 Hz, 1H), 7.02 (d, *J* = 7.6 Hz, 1H), 5.81 (d, *J* = 8.8 Hz, 1H), 5.41 (d, *J* = 8.8 Hz, 1H) ppm; **^{13}C NMR** (125 MHz, $CDCl_3$, 25 °C): δ = 199.6, 157.7, 150.2, 136.1, 134.4, 133.5, 132.2, 131.5, 129.2, 129.1, 127.6, 125.9, 125.6, 121.8, 84.4, 54.4 ppm; **FT-IR** (neat): ν = 3094, 2980, 1714, 1585, 1467, 1326, 876, 754 cm^{-1}; **HRMS** (ESI), *m*/*z*: $[M + Na]^+$ calcd for $C_{16}H_{10}BrNNaO_2^+$ 349.9787; found 349.9781.

3-(2-(Trifluoromethyl)phenyl)-3a,8b-dihydro-4*H*-indeno[2,1-*d*]isoxazol-4-one (**14h**)

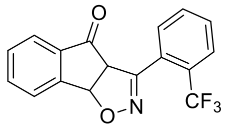

Yield: 87%; white solid; **m.p.** 164–165 °C; $\boldsymbol{R_f}$ = 0.46 (*n*-hexane/EtOAc 2:1 *v/v*); **^{1}H NMR** (500 MHz, $CDCl_3$, 25 °C): *δ* = 7.84 – 7.76 (m, 4H), 7.61 – 7.55 (m, 3H), 7.33 (d, *J* = 7.1 Hz, 1H), 6.32 (d, *J* = 8.3 Hz, 1H), 4.68 (d, *J* = 8.3 Hz, 1H) ppm; **^{13}C NMR** (125 MHz, $CDCl_3$, 25 °C): *δ* = 196.8, 152.0, 150.9, 136.4, 134.4, 131.8, 131.1, 130.7, 130.0, 129.2 (q, *J* = 31.2 Hz, 1C), 127.1, 126.8 (q, *J* = 5.1 Hz, 1C), 126.6 (q, *J* = 1.8 Hz, 1C), 124.1, 123.6 (q, *J* = 273.6 Hz, 1C), 82.6, 63.8 (q, *J* = 2.0 Hz, 1C) ppm; **^{19}F NMR** (470 MHz, $CDCl_3$, 25 °C): δ = -58.67 (s, 3F) ppm; **FT-IR** (neat): ν = 3040, 1718, 1603, 1313, 1273, 1184, 1119, 874, 768 cm^{-1}; **HRMS** (ESI), *m*/*z*: $[M + Na]^+$ calcd for $C_{17}H_{10}F_3NNaO_2^+$ 340.0556; found 340.0562.

3-(2-(Trifluoromethyl)phenyl)-3a,8a-dihydro-8*H*-indeno[1,2-*d*]isoxazol-8-one (**21h**)

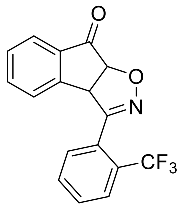

Yield: 3%; white solid; **m.p.** 135–136 °C; $\boldsymbol{R_f}$ = 0.40 (*n*-hexane/EtOAc 2:1 *v/v*); **^{1}H NMR** (500 MHz, $CDCl_3$, 25 °C): *δ* = 7.88 (d, *J* = 7.0 Hz, 1H), 7.82 (d, *J* = 7.8 Hz, 1H), 7.61 (t, *J* = 7.7 Hz, 1H), 7.54 (t, *J* = 7.5 Hz, 1H), 7.51 – 7.44 (m, 2H), 7.15 (d, *J* = 7.6 Hz, 1H), 6.90 (d, *J* = 7.3 Hz, 1H), 5.42 (d, *J* = 8.8 Hz, 1H), 5.35 (d, *J* = 8.8 Hz, 1H) ppm; **^{13}C NMR** (125 MHz, $CDCl_3$, 25 °C): *δ* = 199.5, 156.3, 149.9, 136.0, 134.5, 132.1, 131.9, 130.3, 129.5, 128.9 (q, *J* = 31.2 Hz, 1C), 126.8 (q, *J* = 5.1 Hz, 1C), 126.4 (q, *J* = 1.7 Hz, 1C), 125.9, 125.8, 123.8 (q, *J* = 273.4 Hz, 1C), 84.5, 56.3 (q, *J* = 1.6 Hz, 1C) ppm; **^{19}F NMR** (470 MHz, $CDCl_3$, 25 °C): δ = -58.52 (s, 3F) ppm; **FT-IR** (neat): ν = 2971, 1716, 1313, 1180, 1110, 877, 771 cm^{-1}; **HRMS** (ESI), *m*/*z*: $[M + Na]^+$ calcd for $C_{17}H_{10}F_3NNaO_2^+$ 340.0556; found 340.0555.

3-(3-Nnitrophenyl)-3a,8b-dihydro-4*H*-indeno[2,1-*d*]isoxazol-4-one (**14i**)

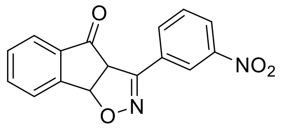

Yield: 89%; white solid; m.p. 158–159 °C; $\boldsymbol{R_f}$ = 0.37 (*n*-hexane/EtOAc 2:1 *v/v*); **^{1}H NMR** (500 MHz, $CDCl_3$, 25 °C): *δ* = 78.88 (t, *J* = 2.0 Hz, 1H), 8.33 (d, *J* = 7.8 Hz, 1H), 8.26 (dd, *J* = 8.3, 1.4 Hz, 1H), 7.85 – 7.75 (m, 3H), 7.64 – 7.56 (m, 2H), 6.40 (d, *J* = 8.4 Hz, 1H), 4.78 (d, *J* = 8.4 Hz, 1H) ppm; **^{13}C NMR** (125 MHz, $CDCl_3$, 25 °C): *δ* = 196.9, 151.0, 150.4, 148.4, 136.6, 134.2, 133.4, 130.9, 129.9, 129.6, 127.1, 124.7, 124.5, 122.9, 83.8, 60.0 ppm; **FT-IR** (neat): ν = 3096, 1728, 1524, 1340, 1278, 928, 764 cm^{-1}; **HRMS** (ESI), *m*/*z*: $[M + K]^+$ calcd for $C_{16}H_{10}KN_2O_4^+$ 333.0272; found 333.0280.

3-(3-Nitrophenyl)-3a,8a-dihydro-8*H*-indeno[1,2-*d*]isoxazol-8-one (**21i**)

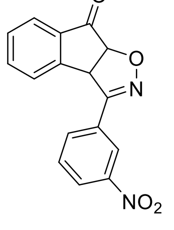

Yield: 3%; white solid; **m.p.** 169–170 °C; $\boldsymbol{R_f}$ = 0.26 (*n*-hexane/EtOAc 2:1 *v/v*); **^{1}H NMR** (500 MHz, $CDCl_3$, 25 °C): *δ* = 8.66 (s, 1H), 8.35 – 8.31 (m, 1H), 8.18 (dd, *J* = 7.8, 1.3 Hz, 1H), 7.87 (d, *J* = 7.7 Hz, 1H), 7.68 (t, *J* = 8.0 Hz, 1H), 7.58 (t, *J* = 7.5 Hz, 1H), 7.47 (t, *J* = 7.5 Hz, 1H), 7.43 (d, *J* = 7.8 Hz, 1H), 5.50 (d, *J* = 8.8 Hz, 1H), 5.45 (d, *J* = 8.7 Hz, 1H) ppm; **^{13}C NMR** (125 MHz, $CDCl_3$, 25 °C): *δ* = 198.1, 155.3, 149.5, 148.4, 136.4, 134.4, 133.2, 130.2, 129.9, 129.6, 126.1, 126.0, 125.0, 122.2, 85.4, 52.6 ppm; **FT-IR** (neat): ν = 3094, 1728, 1531, 1352, 899, 739 cm^{-1}; **HRMS** (ESI), *m*/*z*: $[M + Na]^+$ calcd for $C_{16}H_{10}N_2NaO_4^+$ 317.0533; found 317.0536.

3-Mesityl-3a,8b-dihydro-4*H*-indeno[2,1-*d*]isoxazol-4-one (**14j**)

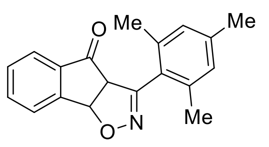

Yield: 87%; white solid; **m.p.** 215–216 °C; $\boldsymbol{R_f}$ = 0.43 (*n*-hexane/DCM 1:2 *v/v*); **^{1}H NMR** (500 MHz, $CDCl_3$, 25 °C): δ = 7.85 (d, *J* = 7.6 Hz, 1H), 7.79 (td, *J* = 8.1, 7.6, 1.4 Hz, 2H), 7.58 (t, *J* = 7.4 Hz, 1H), 6.88 (s, 2H), 6.26 (d, *J* = 8.1 Hz, 1H), 4.52 (d, *J* = 8.0 Hz, 1H), 2.29 (s, 3H), 2.06 (br s, 6H) ppm; **^{13}C NMR** (125 MHz, $CDCl_3$, 25 °C): δ = 197.0, 153.5, 151.3, 139.2, 136.8, 136.2, 134.7, 130.6, 128.7, 127.1, 123.9, 123.7, 81.4, 64.3, 21.1, 19.8 ppm; **FT-IR** (neat): ν = 2945, 2918, 1723, 1595, 1260, 1195, 858, 768 cm^{-1}; **HRMS** (ESI), *m*/*z*: [M + Na]$^+$ calcd for $C_{19}H_{17}NNaO_2^+$ 314.1151; found 314.1159.

3-Mesityl-3a,8a-dihydro-8*H*-indeno[1,2-*d*]isoxazol-8-one (**21j**)

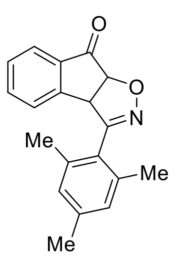

Yield: 3%; white solid; **m.p.** 179–180 °C; $\boldsymbol{R_f}$ = 0.24 (*n*-hexane/DCM 1:2 *v/v*); **^{1}H NMR** (500 MHz, $CDCl_3$, 25 °C): δ = 7.83 (d, *J* = 7.4 Hz, 1H), 7.51 – 7.43 (m, 2H), 6.86 (d, *J* = 7.4 Hz, 1H), 6.75 (s, 1H), 5.35 (d, *J* = 8.8 Hz, 1H), 5.16 (d, *J* = 8.7 Hz, 1H), 2.38 (s, 3H), 2.28 (s, 3H), 1.39 (s, 3H) ppm; **^{13}C NMR** (125 MHz, $CDCl_3$, 25 °C): δ = 200.4, 156.9, 150.1, 139.2, 137.8, 136.0, 135.9, 134.4, 129.2, 128.6, 128.5, 125.9, 125.3, 123.5, 83.2, 56.6, 21.1, 19.8, 19.0 ppm; **FT-IR** (neat): ν = 3065, 2970, 2918, 1716, 1605, 1465, 841, 765 cm^{-1}; **HRMS** (ESI), *m*/*z*: [M + Na]$^+$ calcd for $C_{19}H_{17}NNaO_2^+$ 314.1151; found 314.1157.

3-(Perfluorophenyl)-3a,8b-dihydro-4*H*-indeno[2,1-*d*]isoxazol-4-one (**14k**)

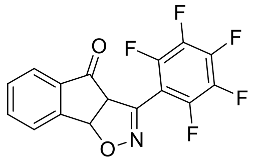

Yield: 80%; white solid; **m.p.** 127–128 °C; $\boldsymbol{R_f}$ = 0.37 (*n*-hexane/EtOAc 3:1 *v/v*); **^{1}H NMR** (500 MHz, $CDCl_3$, 25 °C): δ = 7.85 – 7.76 (m, 3H), 7.60 (td, *J* = 8.0, 1.4 Hz, 1H), 6.37 (d, *J* = 8.5 Hz, 1H), 4.76 (d, *J* = 8.6 Hz, 1H) ppm; **^{13}C NMR** (125 MHz, $CDCl_3$, 25 °C): δ = 196.3, 150.4, 143.1, 136.7, 134.1, 131.0, 127.2, 124.5, 82.9, 61.2 ppm; **^{19}F NMR** (470 MHz, $CDCl_3$, 25 °C): δ = -136.64 (dt, *J* = 20.4, 5.6 Hz, 2F), -150.00 (tt, *J* = 21.1, 3.3 Hz, 1F), -160.42 – -160.56 (m, 2F) ppm; **FT-IR** (neat): ν = 2984, 1717, 1525, 1490, 1337, 1261, 1072, 985, 928, 792, 761 cm^{-1}; **HRMS** (ESI), *m*/*z*: [M + Na]$^+$ calcd for $C_{16}H_6F_5NNaO_2^+$ 362.0211; found 362.0212.

3-(2,6-Dichlorophenyl)-3a,8b-dihydro-4*H*-indeno[2,1-*d*]isoxazol-4-one (**14l**)

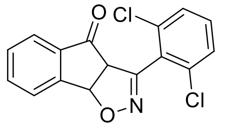

Yield: 89%; white solid; **m.p.** 215–217 °C; $\boldsymbol{R_f}$ = 0.48 (*n*-hexane/EtOAc 2:1 *v/v*); **^{1}H NMR** (500 MHz, $CDCl_3$, 25 °C): δ = 7.85 (d, *J* = 7.6 Hz, 1H), 7.79 (t, *J* = 7.4 Hz, 3H), 7.58 (t, *J* = 7.4 Hz, 1H), 7.40 – 7.31 (m, 3H), 6.35 (d, *J* = 8.2 Hz, 1H), 4.71 (d, *J* = 8.2 Hz, 1H) ppm; **^{13}C NMR** (125 MHz, $CDCl_3$, 25 °C): δ = 196.8, 150.9, 150.2, 136.2, 134.7, 131.5, 130.7, 128.2 (br, 2C), 127.1, 126.8, 124.0, 82.5, 62.9 ppm; **FT-IR** (neat): ν = 3077, 1727, 1603, 1558, 1430, 1260, 1188, 871, 781, 768 cm^{-1}; **HRMS** (ESI), *m*/*z*: [M + Na]$^+$ calcd for $C_{16}H_9Cl_2NNaO_2^+$ 339.9903; found 339.9909.

3-(2,6-Dichlorophenyl)-3a,8a-dihydro-8*H*-indeno[1,2-*d*]isoxazol-8-one (**21l**)

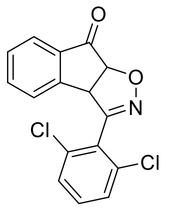

Yield: 4%; white solid; **m.p.** 184–185 °C; R_f = 0.40 (*n*-hexane/EtOAc 2:1 *v/v*); **^{1}H NMR** (500 MHz, $CDCl_3$, 25 °C): δ = 7.85 (d, *J* = 7.6 Hz, 1H), 7.53 (td, *J* = 7.5, 1.3 Hz, 1H), 7.48 – 7.43 (m, 2H), 7.34 (t, *J* = 8.0 Hz, 1H), 7.28 (br s, 1H), 6.94 (d, *J* = 7.6 Hz, 1H), 5.45 (d, *J* = 8.9 Hz, 1H), 5.42 (d, *J* = 8.8 Hz, 1H) ppm; **^{13}C NMR** (125 MHz, $CDCl_3$, 25 °C): δ = 199.7, 153.6, 149.6, 136.3, 134.6, 131.6, 129.4, 128.4, 128.1, 126.5, 125.7, 125.6, 84.1, 55.3 ppm; **FT-IR** (neat): ν = 3092, 2982, 1715, 1609, 1434, 1294, 1194, 865, 773 cm^{-1}; **HRMS** (ESI), *m/z*: $[M + Na]^+$ calcd for $C_{16}H_9Cl_2NNaO_2^+$ 339.9903; found 339.9900.

3-(5-Chlorofuran-2-yl)-3a,8b-dihydro-4*H*-indeno[2,1-*d*]isoxazol-4-one (**14m**)

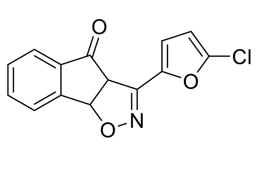

Yield: 70%; white solid; **m.p.** 185–186 °C; R_f = 0.40 (*n*-hexane/EtOAc 2:1 *v/v*); **^{1}H NMR** (500 MHz, $CDCl_3$, 25 °C): δ = 7.83 – 7.75 (m, 3H), 7.56 (td, *J* = 7.4, 1.3 Hz, 1H), 7.18 (d, *J* = 3.5 Hz, 1H), 6.31 (d, *J* = 3.5 Hz, 1H), 6.28 (d, *J* = 8.2 Hz, 1H), 4.50 (d, *J* = 8.3 Hz, 1H) ppm; **^{13}C NMR** (125 MHz, $CDCl_3$, 25 °C): δ = 196.6, 150.5, 143.5, 142.7, 140.0, 136.5, 134.1, 130.8, 127.1, 124.2, 117.4, 108.6, 82.7, 60.3 ppm; **FT-IR** (neat): ν = 3111, 2978, 1732, 1604, 1486, 1198, 1022, 897, 764 cm^{-1}; **HRMS** (ESI), *m/z*: $[M + Na]^+$ calcd for $C_{14}H_8ClNNaO_3^+$ 296.0085; found 296.0081.

3-(Thiophen-2-yl)-3a,8b-dihydro-4*H*-indeno[2,1-*d*]isoxazol-4-one (**14n**)

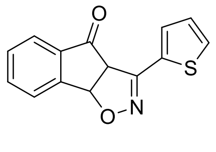

Yield: 64%; white solid; **m.p.** 160–161 °C; R_f = 0.33 (*n*-hexane/DCM 1:1 *v/v*); **^{1}H NMR** (500 MHz, $CDCl_3$, 25 °C): δ = 7.87 (dd, *J* = 3.7, 1.1 Hz, 1H), 7.82 – 7.75 (m, 3H), 7.56 (t, *J* = 7.4 Hz, 1H), 7.38 (dd, *J* = 5.0, 1.1 Hz, 1H), 7.12 (dd, *J* = 5.1, 3.7 Hz, 1H), 6.30 (d, *J* = 8.2 Hz, 1H), 4.64 (d, *J* = 8.2 Hz, 1H) 7.83 – 7.75 (m, 3H), 7.56 (td, *J* = 7.4, 1.3 Hz, 1H), 7.18 (d, *J* = 3.5 Hz, 1H), 6.31 (d, *J* = 3.5 Hz, 1H), 6.28 (d, *J* = 8.2 Hz, 1H), 4.50 (d, *J* = 8.3 Hz, 1H) ppm; **^{13}C NMR** (125 MHz, $CDCl_3$, 25 °C): δ = 196.9, 150.7, 148.4, 136.4, 134.3, 131.3, 130.7, 130.3, 128.7, 127.5, 127.0, 124.2, 83.1, 61.3 ppm; **FT-IR** (neat): ν = 3113, 1717, 1601, 1435, 1265, 933, 905, 773, 713 cm^{-1}; **HRMS** (ESI), *m/z*: $[M + Na]^+$ calcd for $C_{14}H_9NNaO_2S^+$ 278.0246; found 278.0249.

3-Heptyl-3a,8b-dihydro-4*H*-indeno[2,1-*d*]isoxazol-4-one (**14o**)

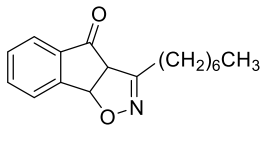

Yield: 92%; white solid; **m.p.** 56–57 °C; R_f = 0.57 (*n*-hexane/EtOAc 3:1 *v/v*); **^{1}H NMR** (500 MHz, $CDCl_3$, 25 °C): δ = 7.78 – 7.72 (m, 3H), 7.54 (td, *J* = 7.9, 7.3, 1.8 Hz, 1H), 6.07 (d, *J* = 8.1 Hz, 1H), 4.21 (d, *J* = 8.1 Hz, 1H), 2.52 – 2.45 (m, 1H), 2.41 – 2.34 (m, 1H), 1.79 – 1.69 (m, 1H), 1.65 – 1.56 (m, 1H), 1.38 – 1.23 (m, 8H), 0.87 (t, *J* = 6.8 Hz, 3H) ppm; **^{13}C NMR** (125 MHz, $CDCl_3$, 25 °C): δ = 198.0, 154.8, 151.6, 136.2, 134.3, 130.4, 127.1, 123.9, 80.9, 62.9, 31.6, 29.1, 28.9, 26.3, 25.8, 22.6, 14.0 ppm; **FT-IR** (neat): ν = 2934, 2848, 1716, 1602, 1470, 1270, 880, 764 cm^{-1}; **HRMS** (ESI), *m/z*: $[M + Na]^+$ calcd for $C_{17}H_{21}NNaO_2^+$ 294.1465; found 294.1460.

Data for compounds **13**

3-Phenyl-3a,8b-dihydro-4*H*-indeno[2,1-*d*]isoxazol-4-one *O*-tosyl oximes (**13a**)

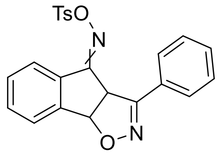

Yield: 96%; white solid (ratio *E*/*Z* 4:1); $\boldsymbol{R_f}$ = 0.31 (*n*-hexane/EtOAc 2:1 *v/v*); **^{1}H NMR** (500 MHz, $CDCl_3$, 25 °C): δ = 8.29 (d, *J* = 8.0 Hz, 1H), 7.76 – 7.68 (m, 8H), 7.67 – 7.63 (m, 3H), 7.61 – 7.50 (m, 3H), 7.48 – 7.37 (m, 3H), 7.32 – 7.17 (m, 8H), 6.19 (d, *J* = 8.6, 1H), 6.18 (d, *J* = 8.2, 1H), 5.51 (d, *J* = 8.1 Hz, 1H), 5.07 (d, *J* = 8.7 Hz, 1H), 2.42 (s, 3H), 2.40 (s, 3H) ppm; **^{13}C NMR** (125 MHz, $CDCl_3$, 25 °C): δ = 162.7, 162.4, 154.1, 154.0, 146.5, 145.2, 145.0, 134.3, 133.5, 132.1, 132.0, 131.9, 130.7, 130.3, 130.14, 130.1, 130.0, 129.6, 129.5, 128.8, 128.6, 128.4, 128.3, 128.27, 128.2, 127.9, 127.7, 126.5, 126.2, 122.8, 85.3, 84.7, 55.4, 55.1, 21.6 (2C) ppm.

3-(*p*-Tolyl)-3a,8b-dihydro-4*H*-indeno[2,1-*d*]isoxazol-4-one *O*-tosyl oximes (**13b**)

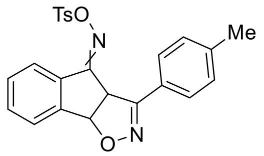

Yield: 95%; white solid (ratio *E*/*Z* 1.5:1); $\boldsymbol{R_f}$ = 0.37 (*n*-hexane/EtOAc 3:1 *v/v*); **^{1}H NMR** (500 MHz, $CDCl_3$, 25 °C): δ = 8.27 (d, *J* = 7.9 Hz, 1H), 7.75 (d, *J* = 8.4 Hz, 2H), 7.68 (d, *J* = 7.7 Hz, 2H), 7.65 – 7.61 (m, 4H), 7.59 – 7.53 (m, 3H), 7.50 (td, *J* = 7.6, 1.3 Hz, 2H), 7.40 (td, *J* = 7.6, 1.1 Hz, 1H), 7.25 – 7.19 (m, 6H), 7.08 (d, *J* = 8.0 Hz, 3H), 6.16 (d, *J* = 8.6 Hz, 1H), 6.15 (d, *J* = 8.1 Hz, 1H), 5.48 (d, *J* = 8.0 Hz, 1H), 5.04 (d, *J* = 8.6 Hz, 1H), 2.43 (s, 3H), 2.41 (s, 3H), 2.40 (s, 3H), 2.39 (s, 3H) ppm; **^{13}C NMR** (125 MHz, $CDCl_3$, 25 °C): δ = 162.8, 162.5, 154.0, 153.9, 146.5, 145.3, 145.2, 144.9, 140.3, 140.1, 134.2, 133.5, 132.1, 132.06, 132.0, 130.6, 130.3, 130.1, 130.0, 129.5, 129.4, 129.0, 128.96, 128.8, 128.7, 128.2, 127.8, 126.5, 126.1, 125.2, 124.9, 122.7, 85.1, 84.5, 55.4, 55.2, 21.7, 21.6, 21.5, 21.4 ppm.

3-(4-Methoxyphenyl)-3a,8b-dihydro-4*H*-indeno[2,1-*d*]isoxazol-4-one *O*-tosyl oximes (**13c**)

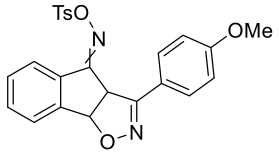

Yield: 99%; white solid (ratio *E*/*Z* 3:1); $\boldsymbol{R_f}$ = 0.43 (*n*-hexane/EtOAc 3:1 *v/v*); **^{1}H NMR** (500 MHz, $CDCl_3$, 25 °C): δ = 8.28 (d, *J* = 8.0 Hz, 1H), 7.74 (d, *J* = 8.4 Hz, 2H), 7.71 – 7.54 (m, 10H), 7.50 (td, *J* = 7.6, 1.3 Hz, 2H), 7.41 (td, *J* = 7.6, 1.1 Hz, 1H), 7.25 (d, *J* = 7.5 Hz, 2H), 7.21 (d, *J* = 8.0 Hz, 2H), 6.91 (d, *J* = 8.9 Hz, 2H), 6.79 (d, *J* = 8.9 Hz, 2H), 6.14 (d, *J* = 8.7 Hz, 1H), 6.13 (d, *J* = 8.1 Hz, H), 5.46 (d, *J* = 8.0 Hz, 1H), 5.02 (d, *J* = 8.6 Hz, 1H), 3.86 (s, 6H), 2.42 (s, 3H), 2.41 (s, 3H) ppm; **^{13}C NMR** (125 MHz, $CDCl_3$, 25 °C): δ = 162.9, 162.5, 161.1, 161.0, 153.7, 153.6, 146.6, 145.3, 145.0, 134.2, 133.5, 132.1, 132.08, 132.0, 130.6, 130.3, 130.1, 130.0, 129.8, 129.5, 129.49, 128.8, 128.7, 126.5, 126.1, 122.7, 120.4, 120.2, 113.8, 113.7, 85.1, 84.4, 55.4, 55.38, 55.37, 55.3, 21.65, 21.62 ppm.

3-(4-(Trifluoromethyl)phenyl)-3a,8b-dihydro-4*H*-indeno[2,1-*d*]isoxazol-4-one *O*-tosyl oximes (**13d**)

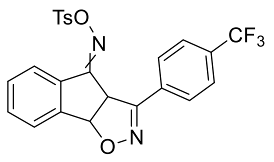

Yield: 98%; white solid (ratio *E*/*Z* 3:1); ***R*f** = 0.43 (*n*-hexane/EtOAc 2:1 *v/v*); **^{1}H NMR** (500 MHz, $CDCl_3$, 25 °C): *δ* = 8.32 (d, *J* = 8.0 Hz, 1H), 7.84 (d, *J* = 8.2 Hz, 3H), 7.80 – 7.75 (m, 4H), 7.73 – 7.64 (m, 4H), 7.64 – 7.53 (m, 4H), 7.50 – 7.43 (m, 4H), 6.25 (t, *J* = 7.9 Hz, 2H), 5.52 (d, *J* = 8.1 Hz, 1H), 5.07 (d, *J* = 8.7 Hz, 1H), 2.43 (s, 3H), 2.41 (s, 3H) ppm; **^{13}C NMR** (125 MHz, $CDCl_3$, 25 °C): *δ* = 162.4, 162.1, 152.99, 152.96, 146.2, 145.55, 145.51, 145.0, 134.5, 133.7, 132.0, 131.8 (q, *J* = 31.9 Hz, 1C), 131.19, 131.18, 130.9, 130.6, 130.2, 130.0, 129.6, 129.5, 128.82, 128.77, 128.74, 128.1, 126.6, 126.2, 125.3 (q, *J* = 3.7 Hz, 1C), 125.1 (q, *J* = 3.8 Hz, 1C), 123.8 (q, *J* = 272.3 Hz, 1C), 122.9, 85.8, 85.2, 55.1, 54.8, 21.7, 21.5 ppm; **^{19}F NMR** (470 MHz, $CDCl_3$, 25 °C): δ = -62.82 (s, 3F), -62.87 (s, 3F) ppm.

3-(4-Fluorophenyl)-3a,8b-dihydro-4*H*-indeno[2,1-*d*]isoxazol-4-one *O*-tosyl oximes (**13e**)

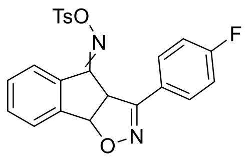

Yield: 99%; white solid (ratio *E*/*Z* 2.5:1); ***R*f** = 0.24(*n*-hexane/EtOAc 3:1 *v/v*); **^{1}H NMR** (500 MHz, $CDCl_3$, 25 °C): *δ* = 8.30 (d, *J* = 7.9 Hz, 1H), 7.74 (d, *J* = 8.4 Hz, 2H), 7.71 – 7.55 (m, 11H), 7.54 – 7.50 (m, 2H), 7.42 (td, *J* = 7.6, 1.2 Hz, 1H), 7.28 – 7.22 (m, 4H), 7.08 (t, *J* = 8.7 Hz, 1H), 6.90 (t, *J* = 8.7 Hz, 2H), 6.18 (d, *J* = 8.6 Hz, 1H), 6.17 (d, *J* = 7.9 Hz, 1H), 5.46 (d, *J* = 8.1 Hz, 1H), 5.01 (d, *J* = 8.6 Hz, 1H), 2.44 (s, 3H), 2.41 (s, 3H) ppm; **^{13}C NMR** (125 MHz, $CDCl_3$, 25 °C): *δ* = 163.8 (d, *J* = 250.5 Hz, 1C), 163.6 (d, *J* = 250.8 Hz, 1C), 162.6, 162.1, 153.1, 146.4, 145.3 (d, *J* = 34.5 Hz, 1C), 145.29, 134.4, 133.6, 132.0, 131.9 (d, *J* = 18.2 Hz, 1C), 130.7, 130.4, 130.3, 130.1, 130.0, 129.9 (d, *J* = 8.5 Hz, 1C), 129.6, 129.5, 128.8, 128.6, 126.5, 126.1, 124.2 (d, *J* = 3.4 Hz, 1C), 123.8 (d, *J* = 3.3 Hz, 1C), 122.8, 115.4 (d, *J* = 21.9 Hz, 1C), 115.3 (d, *J* = 21.9 Hz, 1C), 85.4, 84.7, 55.3, 55.1, 21.6, 21.5 ppm; **^{19}F NMR** (470 MHz, $CDCl_3$, 25 °C): δ = -109.69 (tt, *J* = 8.6, 5.2 Hz, 1F), -109.94 (tt, *J* = 8.7, 5.5 Hz, 1F) ppm.

3-(4-(*tert*-Butyl)phenyl)-3a,8b-dihydro-4*H*-indeno[2,1-*d*]isoxazol-4-one *O*-tosyl oximes (**13f**)

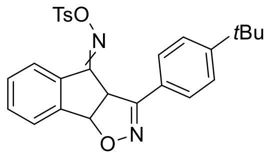

Yield: 98%; white solid (ratio *E*/*Z* 4:1); ***R*f** = 0.25 (*n*-hexane/EtOAc 3:1 *v/v*); **^{1}H NMR** (500 MHz, $CDCl_3$, 25 °C): *δ* = 8.25 (d, *J* = 7.9 Hz, 1H), 7.79 (d, *J* = 8.3 Hz, 3H), 7.75 (d, *J* = 8.5 Hz, 3H), 7.67 (d, *J* = 7.7 Hz, 2H), 7.64 – 7.59 (m, 3H), 7.55 (dd, *J* = 8.0, 5.9 Hz, 2H), 7.51 – 7.44 (m, 2H), 7.39 (t, *J* = 7.5 Hz, 1H), 7.34 (d, *J* = 8.5 Hz, 3H), 7.24 – 7.20 (m, 4H), 6.16 (d, *J* = 8.5 Hz, 2H), 5.51 (d, *J* = 8.1 Hz, 1H), 5.05 (d, *J* = 8.7 Hz, 1H), 2.42 (s, 3H), 2.39 (s, 3H), 1.37 (s, 9H), 1.36 (s, 9H) ppm; **^{13}C NMR** (125 MHz, $CDCl_3$, 25 °C): *δ* = 162.8, 162.7, 153.9, 153.7, 153.4, 153.3, 146.5, 145.2, 145.1, 144.9, 134.2, 133.4, 132.1, 132.0, 131.98, 130.5, 130.2, 130.0, 129.90, 129.5, 129.4, 128.8, 128.7, 128.0, 127.7, 126.4, 126.0, 125.24, 125.19, 125.0, 122.7, 85.2, 84.5, 55.30, 55.28, 34.76, 34.75, 31.15, 31.12, 21.7, 21.6 ppm.

3-(2-Bromophenyl)-3a,8b-dihydro-4*H*-indeno[2,1-*d*]isoxazol-4-one *O*-tosyl oximes (**13g**)

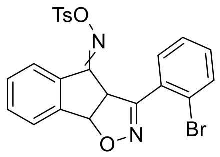

Yield: 99%; white solid (ratio *E*/*Z* 1.7:1); ***R*f** = 0.41 (*n*-hexane/EtOAc 2:1 *v/v*); **^{1}H NMR** (500 MHz, $CDCl_3$, 25 °C): δ = 8.33 (d, *J* = 7.9 Hz, 1H), 7.71 (d, *J* = 7.6 Hz, 1H), 7.69 – 7.64 (m, 3H), 7.63 – 7.57 (m, 3H), 7.56 – 7.50 (m, 3H), 7.46 (t, *J* = 7.6 Hz, 1H), 7.35 (td, *J* = 7.7, 1.8 Hz, 1H), 7.31 – 7.18 (m, 9H), 7.14 (dd, *J* = 7.2, 2.2 Hz, 1H), 7.10 (dd, *J* = 7.5, 1.7 Hz, 1H), 6.21 (d, *J* = 8.4 Hz, 2H), 5.75 (d, *J* = 8.4 Hz, 1H), 5.37 (d, *J* = 8.7 Hz, 1H), 2.46 (s, 3H), 2.42 (s, 3H) ppm; **^{13}C NMR** (125 MHz, $CDCl_3$, 25 °C): δ = 162.7, 161.6, 155.5, 153.6, 146.7, 145.8, 145.1, 144.8, 134.3, 133.7, 133.04, 133.01, 131.95, 131.88, 131.7, 131.1, 130.9, 130.77, 130.76, 130.5, 130.15, 130.14, 130.0, 129.44, 129.36, 129.1, 128.9, 128.8, 127.22, 127.21, 126.7, 126.5, 123.1, 123.0, 122.3, 84.8, 84.3, 56.8, 56.7, 21.71, 21.66 ppm.

3-(2-(Trifluoromethyl)phenyl)-3a,8b-dihydro-4*H*-indeno[2,1-*d*]isoxazol-4-one *O*-tosyl oximes (**13h**)

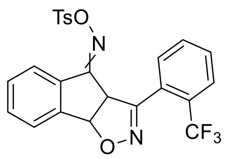

Yield: 99%; white solid (ratio *E*/*Z* 1.7:1); ***R*f** = 0.28 (*n*-hexane/EtOAc 2:1 *v/v*); **^{1}H NMR** (500 MHz, $CDCl_3$, 25 °C): δ = 8.34 (d, *J* = 7.9 Hz, 1H), 7.75 – 7.43 (m, 19H), 7.29 (d, *J* = 7.5 Hz, 1H), 7.25 (d, *J* = 8.3 Hz, H), 7.20 (d, *J* = 7.6 Hz, 1H), 7.16 (d, *J* = 8.1 Hz, 1H), 6.21 (d, *J* = 8.6 Hz, 1H), 6.20 (d, *J* = 8.4 Hz, 1H), 5.42 (d, *J* = 8.4 Hz, 1H), 4.95 (d, *J* = 8.6 Hz, 1H), 2.45 (s, 3H), 2.42 (s, 3H) ppm; **^{13}C NMR** (125 MHz, $CDCl_3$, 25 °C): δ = 162.6, 161.4, 153.7, 151.5, 146.7, 145.8, 145.2, 144.8, 134.4, 133.8, 131.9, 131.8, 131.7, 131.63, 131.60, 131.4, 130.8, 130.6, 130.5, 130.07, 130.01, 129.8, 129.5, 129.39, 129.38, 129.2 (q, *J* = 31.1 Hz, 1C), 129.1 (q, *J* = 31.4 Hz, 1C), 128.7, 127.1 (q, *J* = 2.3 Hz, 1C), 126.73, 126.67 (q, *J* = 4.9 Hz, 1C), 126.5, 126.3 (q, *J* = 5.0 Hz, 1C), 123.5 (q, *J* = 273.8 Hz, 1C), 123.4 (q, *J* = 273.7 Hz, 1C), 122.9, 84.8, 84.2, 58.3 (q, *J* = 2.6 Hz, 2C), 21.7, 21.6 ppm; **^{19}F NMR** (470 MHz, $CDCl_3$, 25 °C): δ = -58.74 (s, 3F), -58.82 (s, 3F) ppm.

3-(3-Nitrophenyl)-3a,8b-dihydro-4*H*-indeno[2,1-*d*]isoxazol-4-one *O*-tosyl oximes (**13i**)

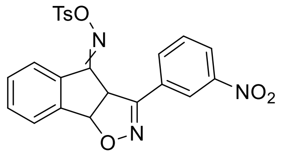

Yield: 96%; white solid (ratio *E*/*Z* 1.6:1); ***R*f** = 0.28 (*n*-hexane/EtOAc 2:1 *v/v*); **^{1}H NMR** (500 MHz, $CDCl_3$, 25 °C): δ = 8.56 (t, *J* = 2.0 Hz, 1H), 8.43 (t, *J* = 2.0 Hz, 1H), 8.34 (d, *J* = 8.0 Hz, 1H), 8.30 (dd, *J* = 7.9, 1.8 Hz, 1H), 8.22 (dd, *J* = 8.2, 1.4 Hz, 1H), 8.08 (d, *J* = 7.8 Hz, 1H), 8.03 (d, *J* = 7.8 Hz, 1H), 7.77 – 7.59 (m, 10H), 7.55 (t, *J* = 7.2 Hz, 1H), 7.47 (q, *J* = 7.5, 6.9 Hz, 2H), 7.27 – 7.21 (m, 4H), 6.28 (d, *J* = 8.8 Hz, 1H), 6.27 (d, *J* = 8.2 Hz, 1H), 5.51 (d, *J* = 8.3 Hz, 1H), 5.09 (d, *J* = 8.8 Hz, 1H), 2.41 (s, 3H), 2.40 (s, 3H) ppm; **^{13}C NMR** (125 MHz, $CDCl_3$, 25 °C): δ = 162.4, 161.9, 152.7, 152.2, 148.05, 148.00, 146.1, 145.6, 145.3, 144.9, 134.6, 134.1, 133.8, 133.5, 131.9, 131.8, 131.6, 131.0, 130.7, 130.2, 130.1, 130.0, 129.61, 129.56, 129.5, 129.4, 129.3, 128.7, 128.6, 126.6, 126.3, 124.6, 124.5, 123.4, 123.0, 122.9, 86.2, 85.4, 55.1, 54.5, 21.64, 21.61 ppm.

3-Mesityl-3a,8b-dihydro-4*H*-indeno[2,1-*d*]isoxazol-4-one *O*-tosyl oximes (**13j**)

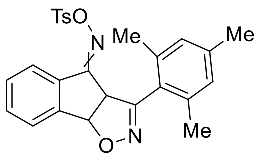

Yield: 86%; white solid (ratio *E*/*Z* 3:1); $\boldsymbol{R_f}$ = 0.33 (*n*-hexane/EtOAc 3:1 *v/v*); **^{1}H NMR** (500 MHz, $CDCl_3$, 25 °C): δ = 8.34 (d, *J* = 7.9 Hz, 1H), 7.75 – 7.65 (m, 2H), 7.63 – 7.58 (m, 2H), 7.57 – 7.52 (m, 1H), 7.44 (t, *J* = 7.5 Hz, 2H), 7.38 (d, *J* = 8.3 Hz, 3H), 7.23 (d, *J* = 8.1 Hz, 4H), 7.19 (d, *J* = 8.1 Hz, 1H), 6.97 (s, 2H), 6.69 (s, 2H), 6.15 (d, *J* = 8.2 Hz, 2H), 5.44 (d, *J* = 8.0 Hz, 1H), 4.81 (d, *J* = 8.5 Hz, 1H), 2.48 (s, 3H), 2.44 (s, 3H), 2.42 (s, 6H), 2.35 (s, 3H), 2.31 (s, 3H), 1.57 (s, 6H) ppm; **^{13}C NMR** (125 MHz, $CDCl_3$, 25 °C): δ = 162.9, 161.8, 155.1, 153.4, 147.2, 146.2, 144.9, 144.7, 138.8, 138.5, 137.4, 137.0, 134.3, 133.6, 132.0, 131.9, 131.8, 130.7, 130.4, 130.2, 129.9, 129.3, 129.2, 128.8, 128.7, 128.5, 128.4, 126.7, 126.5, 124.5, 123.7, 122.6, 83.7, 83.1, 58.7, 57.8, 21.8, 21.7, 21.21, 21.18, 19.7 (2C), 19.3 (2C) ppm.

3-(Perfluorophenyl)-3a,8b-dihydro-4*H*-indeno[2,1-*d*]isoxazol-4-one *O*-tosyl oximes (**13k**)

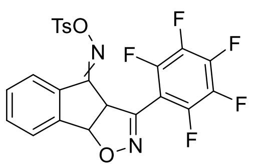

Yield: 80%; white solid (ratio *E*/*Z* 5:1); $\boldsymbol{R_f}$ = 0.28 (*n*-hexane/EtOAc 3:1 *v/v*); **^{1}H NMR** (500 MHz, $CDCl_3$, 25 °C): δ = 8.36 (d, *J* = 7.9 Hz, 1H), 7.76 – 7.70 (m, 4H), 7.70 – 7.61 (m, 3H), 7.58 (ddd, *J* = 8.3, 7.0, 1.6 Hz, 1H), 7.50 (td, *J* = 7.5, 1.3 Hz, 2H), 7.33 (d, *J* = 8.1 Hz, 4H), 7.31 (d, *J* = 8.3 Hz, 1H), 6.27 (d, *J* = 8.7 Hz, 1H), 6.26 (d, *J* = 8.9 Hz, 1H), 5.41 (d, *J* = 8.7 Hz, 1H), 5.05 (d, *J* = 8.9 Hz, 1H), 2.48 (s, 3H), 2.44 (s, 3H) ppm; **^{13}C NMR** (125 MHz, $CDCl_3$, 25 °C): δ = 162.6, 161.1, 146.1, 145.8, 145.6, 145.1, 142.2, 134.7, 134.1, 131.8, 131.6, 131.5, 131.2, 130.9, 130.3, 129.8, 129.7, 129.4, 128.7, 128.6, 126.9, 126.6, 123.3, 85.6, 84.6, 56.1 (2C), 21.7, 21.5 ppm; **^{19}F NMR** (470 MHz, $CDCl_3$, 25 °C): δ = 136.51 – -136.64 (m, 2Fa), -138.64 (d, *J* = 21.4 Hz, 2Fb), -150.17 (t, *J* = 21.0 Hz, 1Fa), -150.50 (t, *J* = 20.9 Hz, 1Fb), -160.57 (td, *J* = 22.1, 8.0 Hz, 2Fb), -160.66 – -160.82 (m, 2Fa) ppm.

3-(2,6-Dichlorophenyl)-3a,8b-dihydro-4*H*-indeno[2,1-*d*]isoxazol-4-one *O*-tosyl oximes (**13l**)

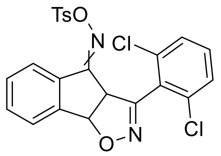

Yield: 94%; white solid (ratio *E*/*Z* 4.5:1); $\boldsymbol{R_f}$ = 0.43 (*n*-hexane/EtOAc 2:1 *v/v*); **^{1}H NMR** (500 MHz, $CDCl_3$, 25 °C): δ = 8.36 (d, *J* = 8.0 Hz, 1H), 7.74 – 7.58 (m, 9H), 7.55 (td, *J* = 7.7, 1.3 Hz, 1H), 7.50 – 7.42 (m, 3H), 7.34 (t, *J* = 8.1 Hz, 2H), 7.29 (d, *J* = 8.0 Hz, 3H), 7.24 – 7.17 (m, 2H), 6.97 (d, *J* = 8.1 Hz, 1H), 6.25 (d, *J* = 8.6 Hz, 1H), 6.22 (d, *J* = 8.4 Hz, 1H), 5.55 (d, *J* = 8.4 Hz, 1H), 4.98 (d, *J* = 8.6 Hz, 1H), 2.47 (s, 3H), 2.43 (s, 3H) ppm; **^{13}C NMR** (125 MHz, $CDCl_3$, 25 °C): δ = 163.1, 161.2, 151.8, 149.9, 146.6, 145.8, 145.2, 144.7, 135.4, 135.3, 134.3, 133.7, 132.0, 131.99, 131.3, 130.8, 130.7, 130.6, 130.0, 129.5, 129.4, 128.9, 128.8, 128.0, 127.4, 126.7, 126.5, 123.0, 84.7, 84.0, 57.5, 56.5, 21.7, 21.6 ppm.

3-(5-Chlorofuran-2-yl)-3a,8b-dihydro-4*H*-indeno[2,1-*d*]isoxazol-4-one *O*-tosyl oximes (**13m**)

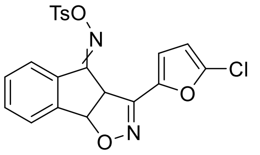

Yield: 99%; white solid (ratio *E*/*Z* 1.2:1); $\boldsymbol{R_f}$ = 0.26 (*n*-hexane/EtOAc 2:1 *v/v*); **¹H NMR** (500 MHz, $CDCl_3$, 25 °C): *δ* = 8.30 (d, *J* = 7.9 Hz, 1H), 7.90 (dd, *J* = 11.9, 8.4 Hz, 2H), 7.69 – 7.60 (m, 5H), 7.58 – 7.50 (m, 2H), 7.43 (t, *J* = 7.5 Hz, 1H), 7.35 (dd, *J* = 8.0, 5.6 Hz, 5H), 7.00 (d, *J* = 3.5 Hz, 1H), 6.73 (d, *J* = 3.5 Hz, 1H), 6.26 (d, *J* = 3.6 Hz, 1H), 6.14 (d, *J* = 8.7 Hz, 1H), 6.12 (d, *J* = 7.8 Hz, 1H), 6.05 (d, *J* = 3.5 Hz, 1H), 5.27 (d, *J* = 7.8 Hz, 1H), 4.79 (d, *J* = 8.6 Hz, 1H), 2.47 (s, 3H), 2.44 (s,3H) ppm; **¹³C NMR** (125 MHz, $CDCl_3$, 25 °C): *δ* = 162.5, 161.7, 146.2, 145.7, 145.4, 144.9, 144.8, 144.5, 142.5, 142.3, 139.59, 139.56, 134.5, 133.6, 132.1, 131.9, 131.8, 130.8, 130.5, 129.9, 129.8, 129.72, 129.70, 129.0, 128.9, 126.6, 126.1, 122.7, 116.8, 115.8, 108.4, 108.2, 85.4, 84.4, 55.0, 53.7, 21.7 (2C) ppm.

3-(Thiophen-2-yl)-3a,8b-dihydro-4*H*-indeno[2,1-*d*]isoxazol-4-one *O*-tosyl oximes (**13n**)

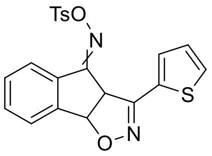

Yield: 96%; white solid (ratio *E*/*Z* 1:1); $\boldsymbol{R_f}$ = 0.27 (*n*-hexane/EtOAc 2:1 *v/v*); **¹H NMR** (500 MHz, $CDCl_3$, 25 °C): *δ* = 8.30 (d, *J* = 7.9 Hz, 1H), 7.87 (dd, *J* = 8.3, 6.1 Hz, 4H), 7.69 (dd, *J* = 7.7, 1.1 Hz, 1H), 7.66 – 7.60 (m, 4H), 7.56 (td, *J* = 7.5, 1.2 Hz, 1H), 7.52 (td, *J* = 7.6, 1.3 Hz, 1H), 7.45 (dd, *J* = 3.7, 1.1 Hz, 1H), 7.42 (t, *J* = 7.7 Hz, 1H), 7.36 – 7.30 (m, 6H), 7.06 (dd, *J* = 5.1, 3.8 Hz, 1H), 6.83 (dd, *J* = 5.1, 3.7 Hz, 1H), 6.17 (d, *J* = 8.5 Hz, 1H), 6.14 (d, *J* = 7.7 Hz, 1H), 5.39 (d, *J* = 7.7 Hz, 1H), 4.93 (d, *J* = 8.5 Hz, 1H), 2.46 (s, 3H), 2.44 (s, 3H) ppm; **¹³C NMR** (125 MHz, $CDCl_3$, 25 °C): *δ* = 162.8, 162.0, 149.7, 149.5, 146.4, 145.6, 145.3, 144.7, 134.4, 133.5, 132.1, 132.0, 131.9, 130.7, 130.5, 130.4, 130.2, 129.93, 129.92, 129.9, 129.7, 129.69, 129.67, 129.0, 128.9, 128.5, 128.3, 127.5, 127.1, 126.5, 126.1, 122.7, 85.6, 84.8, 56.0, 54.8, 21.71, 21.70 ppm.

3-Heptyl-3a,8b-dihydro-4*H*-indeno[2,1-*d*]isoxazol-4-one *O*-tosyl oximes (**13o**)

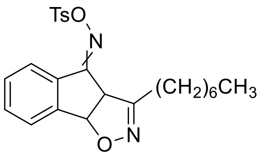

Yield: 97%; yellowish oil (ratio *E*/*Z* 2.5:1); $\boldsymbol{R_f}$ = 0.43 (*n*-hexane/EtOAc 3:1 *v/v*); **¹H NMR** (500 MHz, $CDCl_3$, 25 °C): *δ* = 8.32 (d, *J* = 7.9 Hz, 1H), 7.95 (d, *J* = 8.3 Hz, 2H), 7.93 (d, *J* = 8.5 Hz, 1H), 7.67 – 7.61 (m, 2H), 7.61 – 7.53 (m, 3H), 7.51 (ddd, *J* = 8.3, 6.1, 2.4 Hz, 1H), 7.42 (td, *J* = 7.5, 1.3 Hz, 2H), 7.37 (t, *J* = 8.6 Hz, 4H), 5.95 (d, *J* = 8.4 Hz, 1H), 5.93 (d, *J* = 8.1 Hz, 1H), 4.97 (d, *J* = 8.2 Hz, 1H), 4.46 (d, *J* = 8.5 Hz, 1H), 2.46 (s, 6H), 2.31 (t, *J* = 7.6 Hz, 4H), 2.20 – 2.12 (m, 1H), 2.05 – 1.97 (m, 1H), 1.75 – 1.63 (m, 2H), 1.61 – 1.48 (m, 2H), 1.47 – 1.37 (m, 1H), 1.35 – 1.22 (m, 12H), 1.21 – 1.15 (m, 1H), 0.89 (q, *J* = 7.0 Hz, 6H) ppm; **¹³C NMR** (125 MHz, $CDCl_3$, 25 °C): *δ* = 163.9, 162.1, 156.0, 154.8, 147.5, 146.3, 145.5, 145.3, 134.3, 133.6, 132.2, 132.0, 131.5, 130.5, 130.2, 129.9, 129.8, 129.7, 129.6, 128.98, 128.95, 126.7, 126.3, 122.7, 83.3, 82.5, 57.1, 56.7, 31.7, 31.6, 29.2, 29.1, 28.94, 28.88, 27.4, 26.0, 25.8, 25.6, 22.6, 22.5, 21.7, 21.6, 14.04, 14.03 ppm.

Data for compounds **22**

2-(3-Phenylisoxazol-5-yl)benzonitrile (**22a**)

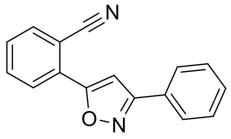

Yield: 80%; white solid; $\boldsymbol{R_f}$ = 0.53 (*n*-hexane/EtOAc 3:1 *v/v*); **^{1}H NMR** (500 MHz, $CDCl_3$, 25 °C): δ = 8.14 (d, *J* = 8.0 Hz, 1H), 7.93 – 7.87 (m, 2H), 7.83 (d, *J* = 7.8 Hz, 1H), 7.76 (td, *J* = 7.8, 1.4 Hz, 1H), 7.56 (t, *J* = 7.7 Hz, 1H), 7.52 – 7.48 (m, 4H) ppm. All spectroscopic data were in accordance with those reported in the literature [2].

2-(3-(*p*-Tolyl)isoxazol-5-yl)benzonitrile (**22b**)

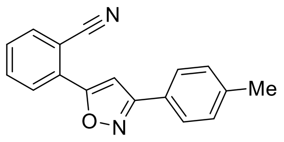

Yield: 76%; white solid; **m.p.** 142–143 °C; $\boldsymbol{R_f}$ = 0.51 (*n*-hexane/EtOAc 3:1 *v/v*); **^{1}H NMR** (500 MHz, $CDCl_3$, 25 °C): δ = 8.12 (dd, *J* = 8.1, 1.1 Hz, 1H), 7.82 (dd, *J* = 7.8, 1.3 Hz, 1H), 7.79 (d, *J* = 8.1 Hz, 2H), 7.75 (td, *J* = 7.8, 1.4 Hz, 1H), 7.55 (td, *J* = 7.7, 1.2 Hz, 1H), 7.45 (s, 1H), 7.30 (d, *J* = 7.9 Hz, 2H), 2.42 (s, 3H) ppm; **^{13}C NMR** (125 MHz, $CDCl_3$, 25 °C): δ = 165.5, 163.3, 140.5, 134.2, 133.3, 129.9, 129.8, 129.7, 127.7, 126.8, 125.6, 118.0, 109.0, 101.6, 21.4 ppm; **FT-IR** (neat): ν = 3042, 2222, 1567, 1438, 1385, 1181, 948, 801, 768 cm^{-1}; **HRMS** (ESI), *m*/*z*: $[M + Na]^+$ calcd for $C_{17}H_{12}N_2NaO^+$ 283.0842; found 283.0845.

2-(3-(4-Methoxyphenyl)isoxazol-5-yl)benzonitrile (**22c**)

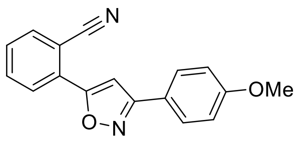

Yield: 75%; white solid; **m.p.** 149–150 °C; $\boldsymbol{R_f}$ = 0.49 (*n*-hexane/EtOAc 3:1 *v/v*); **^{1}H NMR** (500 MHz, $CDCl_3$, 25 °C): δ = 8.10 (dd, *J* = 8.1, 1.2 Hz, 1H), 7.85 – 7.79 (m, 3H), 7.74 (td, *J* = 7.7, 1.3 Hz, 1H), 7.54 (td, *J* = 7.7, 1.2 Hz, 1H), 7.41 (s, 1H), 7.00 (d, *J* = 8.8 Hz, 2H), 3.86 (s, 3H) ppm; **^{13}C NMR** (125 MHz, $CDCl_3$, 25 °C): δ = 165.4, 162.9, 161.2, 134.2, 133.3, 129.9, 129.8, 128.3, 127.7, 121.0, 118.0, 114.3, 109.0, 101.4, 55.3 ppm; **FT-IR** (neat): ν = 3035, 2955, 2226, 1612, 1493, 1435, 1307, 1257, 1177, 1019, 801, 768 cm^{-1}; **HRMS** (ESI), *m*/*z*: $[M + Na]^+$ calcd for $C_{17}H_{12}N_2NaO_2^+$ 299.0791; found 299.0791.

2-(3-(4-(Trifluoromethyl)phenyl)isoxazol-5-yl)benzonitrile (**22d**)

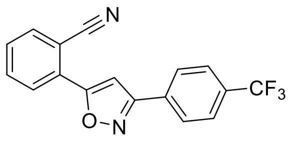

Yield: 72%; white solid; **m.p.** 144–145 °C; $\boldsymbol{R_f}$ = 0.40 (*n*-hexane/EtOAc 3:1 *v/v*); **^{1}H NMR** (500 MHz, $CDCl_3$, 25 °C): δ = 8.14 (dd, *J* = 8.0, 1.2 Hz, 1H), 8.02 (d, *J* = 8.1 Hz, 2H), 7.84 (dd, *J* = 7.8, 1.4 Hz, 1H), 7.80 – 7.74 (m, 3H), 7.59 (td, *J* = 7.7, 1.2 Hz, 1H), 7.51 (s, 1H) ppm; **^{13}C NMR** (125 MHz, $CDCl_3$, 25 °C): δ = 166.3, 162.2, 134.3, 133.4, 132.1 (q, *J* = 32.7 Hz, 1C), 131.9 (d, *J* = 1.6 Hz, 1C), 130.3, 129.4, 127.8, 127.3, 126.0 (q, *J* = 3.8 Hz, 1C), 123.8 (q, *J* = 272.5 Hz, 1C), 117.9, 109.2, 101.6 ppm; **^{19}F NMR** (470 MHz, $CDCl_3$, 25 °C): δ = -62.90 (s, 3F) ppm; **FT-IR** (neat): ν = 3071, 2228, 1442, 1327, 1161, 1110, 1066, 850, 765 cm^{-1}; **HRMS** (ESI), *m*/*z*: $[M + Na]^+$ calcd for $C_{17}H_9F_3N_2NaO^+$ 337.0559; found 337.0561.

2-(3-(4-Fluorophenyl)isoxazol-5-yl)benzonitrile (**22e**)

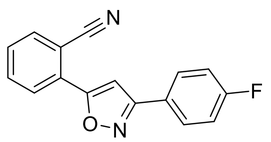

Yield: 81%; white solid; **m.p.** 153–154 °C; ***R*$_f$** = 0.34 (*n*-hexane/EtOAc 4:1 *v/v*); **^{1}H NMR** (500 MHz, $CDCl_3$, 25 °C): *δ* = 8.11 (dd, *J* = 8.1, 1.2 Hz, 1H), 7.90 – 7.85 (m, 2H), 7.81 (d, *J* = 7.8 Hz, 1H), 7.75 (td, *J* = 7.8, 1.4 Hz, 1H), 7.56 (td, *J* = 7.6, 1.2 Hz, 1H), 7.42 (s, 1H), 7.20 – 7.15 (m, 2H) ppm; **^{13}C NMR** (125 MHz, $CDCl_3$, 25 °C): *δ* = 165.8, 163.9 (d, *J* = 250.7 Hz, 1C), 162.4, 134.2, 133.3, 130.1, 129.6, 128.9 (d, *J* = 8.5 Hz, 1C), 127.7, 124.7 (d, *J* = 3.3 Hz, 1C), 117.9, 116.1 (d, *J* = 21.9 Hz, 1C), 109.0, 101.5 ppm; **^{19}F NMR** (470 MHz, $CDCl_3$, 25 °C): δ = -109.92 – -109.98 (m, 1F) ppm; **FT-IR** (neat): v = 3078, 2224, 1607, 1527, 1439, 1230, 837, 764 cm^{-1}; **HRMS** (ESI), *m*/*z*: [M + Na]$^+$ calcd for $C_{16}H_9FN_2NaO^+$ 287.0591; found 287.0587.

2-(3-(4-(*tert*-Butyl)phenyl)isoxazol-5-yl)benzonitrile (**22f**)

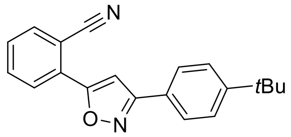

Yield: 85%; white solid; **m.p.** 115–116 °C; ***R*$_f$** = 0.40 (*n*-hexane/EtOAc 4:1 *v/v*); **^{1}H NMR** (500 MHz, $CDCl_3$, 25 °C): *δ* = 8.12 (dd, *J* = 8.0, 1.1 Hz, 1H), 7.85 – 7.80 (m, 3H), 7.75 (td, *J* = 7.8, 1.4 Hz, 1H), 7.57 – 7.50 (m, 3H), 7.46 (s, 1H), 1.37 (s, 9H) ppm; **^{13}C NMR** (125 MHz, $CDCl_3$, 25 °C): *δ* = 165.5, 163.2, 153.6, 134.2, 133.3, 129.9, 129.8, 127.7, 126.7, 125.9, 125.6, 117.9, 109.0, 101.6, 34.8, 31.2 ppm; **FT-IR** (neat): v = 2961, 2224, 1582, 1492, 1436, 1122, 948, 807, 764 cm^{-1}; **HRMS** (ESI), *m*/*z*: [M + Na]$^+$ calcd for $C_{20}H_{18}N_2NaO^+$ 325.1311; found 325.1312.

2-(3-(2-Bromophenyl)isoxazol-5-yl)benzonitrile (**22g**)

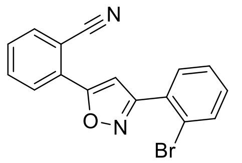

Yield: 57%; white solid; **m.p.** 100–101 °C; ***R*$_f$** = 0.51 (*n*-hexane/EtOAc 3:1 *v/v*); **^{1}H NMR** (500 MHz, $CDCl_3$, 25 °C): *δ* = 8.13 (d, *J* = 8.0 Hz, 1H), 7.83 (d, *J* = 7.8 Hz, 1H), 7.77 (td, *J* = 7.8, 1.4 Hz, 1H), 7.74 – 7.69 (m, 2H), 7.60 (s, 1H), 7.57 (td, *J* = 7.7, 1.2 Hz, 1H), 7.44 (td, *J* = 7.5, 1.2 Hz, 1H), 7.35 (td, *J* = 7.7, 1.8 Hz, 1H) ppm; **^{13}C NMR** (125 MHz, $CDCl_3$, 25 °C): *δ* = 165.0, 163.3, 134.2, 133.7, 133.3, 131.3, 131.2, 130.1, 129.9, 129.7, 127.8, 127.6, 122.3, 117.8, 109.3, 105.1 ppm; **FT-IR** (neat): v = 2981, 2223, 1442, 1396, 1024, 946, 776, 760 cm^{-1}; **HRMS** (ESI), *m*/*z*: [M + Na]$^+$ calcd for $C_{16}H_9BrN_2NaO^+$ 346.9790; found 346.9795.

2-(3-(2-(Trifluoromethyl)phenyl)isoxazol-5-yl)benzonitrile (**22h**)

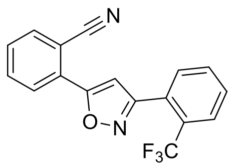

Yield: 59%; white solid; **m.p.** 98–99 °C; ***R*$_f$** = 0.51 (*n*-hexane/EtOAc 3:1 *v/v*); **^{1}H NMR** (500 MHz, $CDCl_3$, 25 °C): *δ* = 8.15 (dd, *J* = 8.1, 1.1 Hz, 1H), 7.83 (td, *J* = 6.7, 6.0, 1.3 Hz, 2H), 7.77 (td, *J* = 7.8, 1.4 Hz, 1H), 7.71 – 7.61 (m, 3H), 7.57 (td, *J* = 7.7, 1.2 Hz, 1H), 7.37 (s, 1H) ppm; **^{13}C NMR** (125 MHz, $CDCl_3$, 25 °C): *δ* = 165.3, 162.3, 134.2, 133.3, 132.0, 131.8, 130.2, 130.0, 129.5, 129.0 (q, *J* = 31.2 Hz, 1C), 127.9, 127.7 (q, *J* = 2.0 Hz, 1C), 126.6 (q, *J* = 5.3 Hz, 1C), 123.6 (q, *J* = 273.7 Hz, 1C), 117.7, 109.3, 105.0 (q, *J* = 3.0 Hz, 1C) ppm; **^{19}F NMR** (470 MHz, $CDCl_3$, 25 °C): δ = -58.06 (s, 3F) ppm; **FT-IR** (neat): v = 3130, 2232, 1578, 1441, 1392, 1312, 1137, 1035, 780, 770 cm^{-1}; **HRMS** (ESI), *m*/*z*: [M + K]$^+$ calcd for $C_{17}H_9F_3KN_2O^+$ 353.0299; found 353.0231.

2-(3-(3-Nitrophenyl)isoxazol-5-yl)benzonitrile (**22i**)

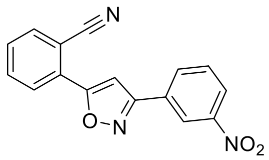

Yield: 86%; white solid; **m.p.** 210–211 °C; $\boldsymbol{R_f}$ = 0.37 (*n*-hexane/EtOAc 3:1 *v/v*); **^{1}H NMR** (500 MHz, $CDCl_3$, 25 °C): δ = 8.74 (t, *J* = 2.0 Hz, 1H), 8.35 (ddd, *J* = 8.2, 2.3, 1.1 Hz, 1H), 8.23 (dt, *J* = 7.8, 1.4 Hz, 1H), 8.15 (d, *J* = 8.0 Hz, 1H), 7.86 (dd, *J* = 7.7, 1.4 Hz, 1H), 7.80 (td, *J* = 7.8, 1.3 Hz, 1H), 7.71 (t, *J* = 8.0 Hz, 1H), 7.61 (td, *J* = 7.7, 1.0 Hz, 1H), 7.53 (s, 1H) ppm; **^{13}C NMR** (125 MHz, $CDCl_3$, 25 °C): δ = 166.8, 161.6, 148.7, 134.3, 133.5, 132.7, 130.5, 130.3, 130.1, 129.3, 127.9, 124.9, 121.9, 117.8, 109.3, 101.5 ppm; **FT-IR** (neat): v = 3089, 2224, 1540, 1461, 1347, 952, 808, 765, 736 cm^{-1}; **HRMS** (ESI), *m*/*z*: $[M + Na]^+$ calcd for $C_{16}H_9N_3NaO_3^+$ 314.0536; found 314.0540.

2-(3-Mesitylisoxazol-5-yl)benzonitrile (**22j**)

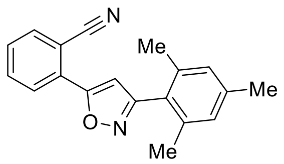

Yield: 12%; white solid; **m.p.** 135–136 °C; $\boldsymbol{R_f}$ = 0.42 (*n*-hexane/EtOAc 3:1 *v/v*); **^{1}H NMR** (500 MHz, $CDCl_3$, 25 °C): δ = 8.17 (dd, *J* = 8.0, 1.2 Hz, 1H), 7.82 (dd, *J* = 7.7, 1.3 Hz, 1H), 7.78 (td, *J* = 7.8, 1.4 Hz, 1H), 7.56 (td, *J* = 7.7, 1.2 Hz, 1H), 7.16 (s, 1H), 6.97 (s, 2H), 2.34 (s, 3H), 2.21 (s, 6H) ppm; **^{13}C NMR** (125 MHz, $CDCl_3$, 25 °C): δ = 165.1, 163.2, 139.1, 137.2, 134.2, 133.3, 129.9, 128.5, 127.8, 125.5, 117.9, 109.2, 105.4, 21.1, 20.3 ppm; **FT-IR** (neat): v = 3144, 2925, 2224, 1609, 1581, 1452, 1377, 916, 841, 774 cm^{-1}; **HRMS** (ESI), *m*/*z*: $[M + Na]^+$ calcd for $C_{19}H_{16}N_2NaO^+$ 311.1155; found 311.1149.

2-(3-(Perfluorophenyl)isoxazol-5-yl)benzonitrile (**22k**)

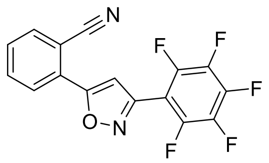

Yield: 54%; white solid; **m.p.** 144–145 °C; $\boldsymbol{R_f}$ = 0.57 (*n*-hexane/EtOAc 3:1 *v/v*); **^{1}H NMR** (500 MHz, $CDCl_3$, 25 °C): δ = 8.14 (dd, *J* = 8.1, 1.2 Hz, 1H), 7.85 (dd, *J* = 7.8, 1.3 Hz, 1H), 7.80 (td, *J* = 7.8, 1.4 Hz, 1H), 7.61 (td, *J* = 7.7, 1.2 Hz, 1H), 7.48 (t, *J* = 1.6 Hz, 1H) ppm; **^{13}C NMR** (125 MHz, $CDCl_3$, 25 °C): δ = 166.3, 152.5, 134.3, 133.5, 130.6, 128.9, 127.9, 117.6, 109.4, 104.5 (t, *J* = 3.6 Hz, 1C) ppm; **^{19}F NMR** (470 MHz, $CDCl_3$, 25 °C): δ = -137.79 – -137.92 (m, 2F), -150.41 (tt, *J* = 20.9, 3.0 Hz, 1F), -160.46 – -160.60 (m, 2F) ppm; **FT-IR** (neat): v = 3142, 3078, 2231, 1506, 1487, 1383, 1093, 999, 988, 778 cm^{-1}; **HRMS** (ESI), *m*/*z*: $[M + Na]^+$ calcd for $C_{16}H_5F_5N_2NaO^+$ 359.0214; found 359.0210.

2-(3-(2,6-Dichlorophenyl)isoxazol-5-yl)benzonitrile (**22l**)

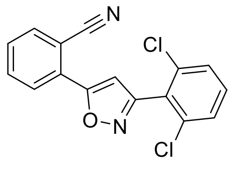

Yield: 53%; white solid; **m.p.** 189–190 °C; $\boldsymbol{R_f}$ = 0.38 (*n*-hexane/EtOAc 3:1 *v/v*); **^{1}H NMR** (500 MHz, $CDCl_3$, 25 °C): δ = 8.17 (dd, *J* = 8.0, 1.2 Hz, 1H), 7.82 (dd, *J* = 7.7, 1.3 Hz, 1H), 7.78 (td, *J* = 8.0, 0.6 Hz, 1H), 7.83 (dd, *J* = 7.8, 0.7 Hz, 1H), 7.78 (td, *J* = 7.8, 1.4 Hz, 1H), 7.58 (td, *J* = 7.7, 1.2 Hz, 1H), 7.46 (d, *J* = 1.0 Hz, 1H), 7.44 (d, *J* = 0.6 Hz, 1H), 7.37 (dd, *J* = 8.8, 7.3 Hz, 1H), 7.31 (s, 1H) ppm; **^{13}C NMR** (125 MHz, $CDCl_3$, 25 °C): δ = 165.5, 159.8, 135.5, 134.2, 133.4, 131.3, 130.2, 129.5, 128.3, 127.81, 127.78, 117.8, 109.2, 105.3 ppm; **FT-IR** (neat): v = 3128, 3085, 2228, 1561, 1435, 1380, 1193, 789, 766 cm^{-1}; **HRMS** (ESI), *m*/*z*: $[M + Na]^+$ calcd for $C_{16}H_8Cl_2N_2NaO^+$ 336.9906; found 336.9909.

2-(3-(5-Chlorofuran-2-yl)isoxazol-5-yl)benzonitrile (**22m**)

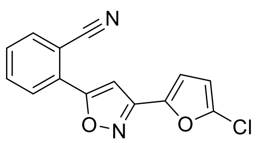

Yield: 71%; white solid; **m.p.** 154–155 °C; ***R*$_f$** = 0.48 (*n*-hexane/EtOAc 3:1 *v/v*); **^{1}H NMR** (500 MHz, $CDCl_3$, 25 °C): δ = 8.10 (d, *J* = 8.0 Hz, 1H), 7.82 (dd, *J* = 7.8, 1.3 Hz, 1H), 7.76 (td, *J* = 7.8, 1.3 Hz, 1H), 7.57 (td, *J* = 7.7, 1.2 Hz, 1H), 7.36 (s, 1H), 6.97 (d, *J* = 3.5 Hz, 1H), 6.34 (d, *J* = 3.5 Hz, 1H) ppm; **^{13}C NMR** (125 MHz, $CDCl_3$, 25 °C): δ = 165.7, 154.9, 143.1, 138.8, 134.2, 133.4, 130.3, 129.2, 127.9, 117.7, 112.8, 109.2, 108.6, 100.8 ppm; **FT-IR** (neat): ν = 3135, 3116, 2225, 1613, 1513, 1432, 1386, 1211, 1020, 797, 768 cm^{-1}; **HRMS** (ESI), *m*/*z*: $[M + K]^+$ calcd for $C_{14}H_7ClKN_2O_2^+$ 308.9828; found 308.9831.

2-(3-(Thiophen-2-yl)isoxazol-5-yl)benzonitrile (**22n**)

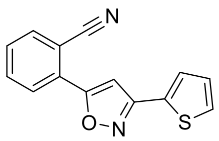

Yield: 70%; white solid; **m.p.** 140–141 °C; ***R*$_f$** = 0.44 (*n*-hexane/EtOAc 3:1 *v/v*); **^{1}H NMR** (500 MHz, $CDCl_3$, 25 °C): δ = 8.10 (d, *J* = 8.0 Hz, 1H), 7.82 (dd, *J* = 7.7, 1.4 Hz, 1H), 7.75 (td, *J* = 7.8, 1.4 Hz, 1H), 7.59 – 7.54 (m, 2H), 7.46 (dd, *J* = 5.1, 1.2 Hz, 1H), 7.39 (s, 1H), 7.15 (dd, *J* = 5.1, 3.6 Hz, 1H) ppm; **^{13}C NMR** (125 MHz, $CDCl_3$, 25 °C): δ = 165.6, 158.6, 134.2, 133.4, 130.2, 130.1, 129.5, 128.04, 128.0, 127.8, 127.7, 117.8, 109.1, 101.6 ppm; **FT-IR** (neat): ν = 3111, 2981, 2229, 1602, 1578, 1429, 1391, 1230, 771, 714 cm^{-1}; **HRMS** (ESI), *m*/*z*: $[M + H]^+$ calcd for $C_{14}H_9N_2OS^+$ 253.0430; found 253.0428.

2-(3-Heptylisoxazol-5-yl)benzonitrile (**22o**)

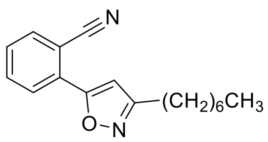

Yield: 82%; white solid; **m.p.** 36–37 °C; ***R*$_f$** = 0.60 (*n*-hexane/EtOAc 3:1 *v/v*); **^{1}H NMR** (500 MHz, $CDCl_3$, 25 °C): δ = 8.06 (d, *J* = 8.1 Hz, 1H), 7.78 (dd, *J* = 7.8, 1.3 Hz, 1H), 7.72 (td, *J* = 7.8, 1.3 Hz, 1H), 7.52 (td, *J* = 7.7, 1.2 Hz, 1H), 7.02 (s, 1H), 2.74 (t, 7.4 Hz, 2H), 1.72 (p, *J* = 7.6 Hz, 2H), 1.43 – 1.24 (m, 8H), 0.88 (t, *J* = 6.8 Hz, 3H) ppm; **^{13}C NMR** (125 MHz, $CDCl_3$, 25 °C): δ = 165.1, 164.8, 134.1, 133.3, 130.0, 129.7, 127.7, 118.0, 108.9, 103.4, 31.6, 29.1, 28.9, 28.2, 26.1, 22.6, 14.0 ppm; **FT-IR** (neat): ν = 3105, 2219, 1541, 1470, 1342, 951, 808, 768, 736 cm^{-1}; **HRMS** (ESI), *m*/*z*: $[M + Na]^+$ calcd for $C_{17}H_{20}N_2NaO^+$ 291.1468; found 291.1460.

Data for compounds **23**

Compounds **23** were isolated in 6-29% yield, as given in Table 2. All spectroscopic data for those compounds were in accordance with those reported in the literature [2].

Data for compound **29**

(*Z*)-2-((*E*)-(Hydroxyimino)(mesityl)methyl)-1*H*-inden-1-one *O*-tosyl oxime (**29**)

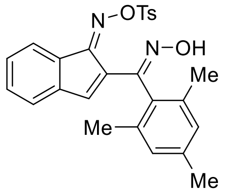

Yield: 65%; yellow solid; **m.p.** >165 °C (decomp.); ***R*$_f$** = 0.28 (*n*-hexane/EtOAc 2:1 *v/v*); **^{1}H NMR** (500 MHz, $CDCl_3$, 25 °C): δ = 8.39 (br s, 1H), 8.16 (dd, *J* = 7.5, 1.1 Hz, 1H), 7.93 (s, 1H), 7.82 (d, *J* = 8.4 Hz, 2H), 7.44 (td, *J* = 7.5, 1.2 Hz, 1H), 7.39 (td, *J* = 7.6, 1.3 Hz, 1H), 7.31 (d, *J* = 7.2 Hz, 1H), 7.14 (d, *J* = 8.1 Hz, 2H), 6.94 (s, 2H), 2.36 (s, 3H), 2.32 (s, 3H), 2.11 (s, 6H) ppm; **^{13}C NMR** (125 MHz, $CDCl_3$, 25 °C): δ = 160.0, 159.1, 148.6, 146.1, 140.5, 137.0, 135.0, 133.3, 131.4, 131.0, 130.9, 130.4, 130.0, 129.97, 128.81, 128.79, 127.8, 124.3, 21.6, 21.0, 18.3 ppm; **FT-IR** (neat): ν = 3355, 2920, 1669, 1593, 1573, 1504, 1374, 1190, 1175, 904, 729 cm^{-1}; **HRMS** (ESI), *m*/*z*: [M + H]$^+$ calcd for $C_{26}H_{25}N_2O_4S^+$ 461.1530; found 461.1533.

Data for compounds **11**

The yields given for compounds **11** correspond to those obtained via oxidation of *syn*/*anti* isomers **10a**/**10b**.

3-Phenyl-3a,9b-dihydroisoxazolo[4,5-*c*]isoquinolin-5(4*H*)-one (**11a**)

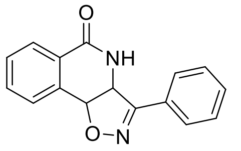

Yields: 45/48%; white solid; **m.p.** 260–261 °C; ***R*$_f$** = 0.43 (*n*-hexane/EtOAc 1:1 *v/v*); **^{1}H NMR** (500 MHz, DMSO-*d*$_6$, 25 °C): δ = 8.56 (br s, 1H), 8.01 (d, *J* = 7.7 Hz, 1H), 7.81 (dd, *J* = 7.6, 2.0 Hz, 2H), 7.73 – 7.67 (m, 2H), 7.60 (td, *J* = 7.4, 1.4 Hz, 1H), 7.54 – 7.47 (m, 3H), 5.74 (d, *J* = 8.1 Hz, 1H), 5.71 (dd, *J* = 8.2, 2.2 Hz, 1H) ppm; **^{13}C NMR** (125 MHz, DMSO-*d*$_6$, 25 °C): δ = 161.9, 159.1, 133.2, 132.8, 131.1, 130.3, 130.1, 129.4, 128.11, 128.09, 127.9, 127.6, 76.8, 58.6 ppm; **FT-IR** (neat): ν = 3181, 2923, 1669, 1583, 1407, 1330, 903, 761, 691 cm^{-1}; **HRMS** (ESI), *m*/*z*: [M + Na]$^+$ calcd for $C_{16}H_{12}N_2NaO_2^+$ 287.0791; found 287.0792.

3-(*p*-Tolyl)-3a,9b-dihydroisoxazolo[4,5-*c*]isoquinolin-5(4*H*)-one (**11b**)

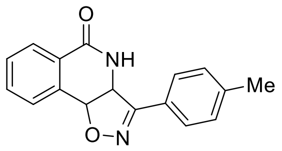

Yields: 44/47%; white solid; **m.p.** >270 °C (decomp.); ***R*$_f$** = 0.54 (*n*-hexane/EtOAc 1:1 *v/v*); **^{1}H NMR** (500 MHz, DMSO-*d*$_6$, 25 °C): δ = 8.52 (br s, 1H), 8.00 (d, *J* = 7.7 Hz, 1H), 7.73 – 7.66 (m, 4H), 7.60 (t, *J* = 7.5 Hz, 1H), 7.30 (d, *J* = 7.8 Hz, 2H), 5.70 (d, *J* = 8.2 Hz, 1H), 5.67 (d, *J* = 8.7 Hz, 1H), 2.37 (s, 3H) ppm; **^{13}C NMR** (125 MHz, DMSO-*d*$_6$, 25 °C): δ = 161.9, 159.0, 140.9, 133.2, 132.92, 130.3, 130.0, 129.96, 128.1, 128.06, 127.6, 125.1, 76.6, 58.7, 21.5 ppm; **FT-IR** (neat): ν = 3177, 2918, 1667, 1604, 1580, 1410, 902, 762 cm^{-1}; **HRMS** (ESI), *m*/*z*: [M + Na]$^+$ calcd for $C_{17}H_{14}N_2NaO_2^+$ 301.0947; found 301.0952.

3-(4-Methoxyphenyl)-3a,9b-dihydroisoxazolo[4,5-*c*]isoquinolin-5(4*H*)-one (**11c**)

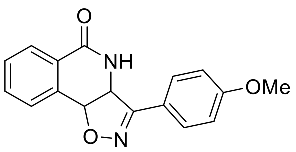

Yields: 53/50%; white solid; **m.p.** >270 °C (decomp.); $\boldsymbol{R_f}$ = 0.44 (*n*-hexane/EtOAc 1:2 *v/v*); **^{1}H NMR** (500 MHz, DMSO-d_6, 25 °C): δ = 8.52 (br s, 1H), 8.00 (d, J = 7.7 Hz, 1H), 7.75 (d, J = 8.3 Hz, 2H), 7.72 – 7.66 (m, 2H), 7.59 (t, J = 7.4 Hz, 1H), 7.04 (d, J = 8.3 Hz, 2H), 5.67 (d, J = 8.5 Hz, 1H), 5.66 (d, J = 8.4 Hz, 1H), 3.82 (s, 3H) ppm; **^{13}C NMR** (125 MHz, DMSO-d_6, 25 °C): δ = 161.9, 161.5, 158.6, 133.2, 133.0, 130.2, 130.1, 129.8, 128.1, 127.6, 120.2, 114.8, 76.5, 58.8, 55.9 ppm; **FT-IR** (neat): ν = 3190, 1659, 1579, 1406, 1342, 905, 793 cm^{-1}; **HRMS** (ESI), *m*/*z*: [M + Na]$^+$ calcd for $C_{17}H_{14}N_2NaO_3^+$ 317.0897; found 317.0891.

3-(4-(Trifluoromethyl)phenyl)-3a,9b-dihydroisoxazolo[4,5-*c*]isoquinolin-5(4*H*)-one (**11d**)

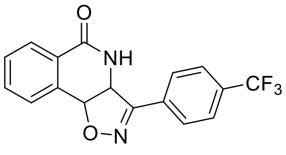

Yields: 42/41%; white solid; **m.p.** >270 °C (decomp.); $\boldsymbol{R_f}$ = 0.37 (*n*-hexane/EtOAc 1:1 *v/v*); **^{1}H NMR** (500 MHz, DMSO-d_6, 25 °C): δ = 8.63 (br s, 1H), 8.07 – 7.99 (m, 3H), 7.86 (d, J = 8.0 Hz, 2H), 7.75 – 7.68 (m, 2H), 7.62 (td, J = 7.6, 1.2 Hz 1H), 5.80 (d, J = 8.3 Hz, 1H), 5.77 (d, J = 8.3 Hz, 1H) ppm; **^{13}C NMR** (125 MHz, DMSO-d_6, 25 °C): δ = 161.8, 158.5, 133.3, 132.3, 131.9, 130.8 (q, J = 32.0 Hz, 1C), 130.5, 130.2, 129.0, 128.1, 127.7, 126.2 (q, J = 4.8 Hz, 1C), 124.5 (q, J = 272.2 Hz, 1C), 77.6, 58.3 ppm; **^{19}F NMR** (470 MHz, DMSO-d_6, 25 °C): δ = -62.92 (s, 3F) ppm; **FT-IR** (neat): ν = 3175, 2931, 1660, 1604, 1410, 1135, 981, 789 cm^{-1}; **HRMS** (ESI), *m*/*z*: [M + Na]$^+$ calcd for $C_{17}H_{11}F_3N_2NaO_2^+$ 355.0665; found 355.0671.

3-(2-Bromophenyl)-3a,9b-dihydroisoxazolo[4,5-*c*]isoquinolin-5(4*H*)-one (**11g**)

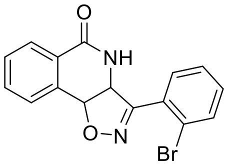

Yields: 48/51%; white solid; **m.p.** 199–200 °C; $\boldsymbol{R_f}$ = 0.32 (*n*-hexane/EtOAc 2:1 *v/v*); **^{1}H NMR** (500 MHz, DMSO-d_6, 25 °C): δ = 8.49 (d, J = 3.0 Hz, 1H), 8.01 (dd, J = 7.8, 1.2 Hz, 1H), 7.77 (dd, J = 7.9, 1.2 Hz, 1H), 7.73 – 7.66 (m, 2H), 7.58 (td, J = 7.0, 1.9 Hz, 1H), 7.53 – 7.43 (m, 3H), 5.91 (d, J = 9.0 Hz, 1H), 5.73 (dd, J = 9.0, 3.0 Hz, 1H) ppm; **^{13}C NMR** (125 MHz, DMSO-d_6, 25 °C): δ = 161.8, 159.2, 133.6, 133.5, 133.2, 132.4, 132.3, 130.0, 129.7, 129.4, 128.3, 127.9, 127.4, 122.6, 75.9, 61.2 ppm; **FT-IR** (neat): ν = 3174, 3067, 1664, 1585, 1405, 1311, 752 cm^{-1}; **HRMS** (ESI), *m*/*z*: [M + Na]$^+$ calcd for $C_{16}H_{11}BrN_2NaO_2^+$ 364.9896; found 364.9901.

3-(2-(Trifluoromethyl)phenyl)-3a,9b-dihydroisoxazolo[4,5-*c*]isoquinolin-5(4*H*)-one (**11h**)

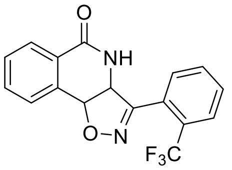

Yields: 48/45%; white solid; **m.p.** >255 °C (decomp.); $\boldsymbol{R_f}$ = 0.6 (*n*-hexane/EtOAc 1:1 *v/v*); **^{1}H NMR** (500 MHz, DMSO-d_6, 25 °C): δ = 8.52 (d, J = 3.3 Hz, 1H), 8.00 (dd, J = 7.8, 1.4 Hz, 1H), 7.88 (dd, J = 7.9, 1.2 Hz, 1H), 7.82 (td, J = 7.6, 1.3 Hz, 1H), 7.76 – 7.64 (m, 4H), 7.57 (td, J = 7.4, 1.6 Hz, 1H), 5.97 (d, J = 9.4 Hz, 1H), 5.67 (dd, J = 9.4, 3.3 Hz, 1H) ppm; **^{13}C NMR** (125 MHz, DMSO-d_6, 25 °C): δ = 161.8, 157.7, 134.0, 133.2, 133.0, 131.9, 130.9, 129.9, 129.4, 128.6 (q, J = 30.5 Hz, 1C), 127.8, 127.3, 127.2 (q, J = 5.2 Hz, 1C), 126.7 (q, J = 2.3 Hz, 1C), 124.1 (q, J = 273.9 Hz, 1C), 75.4, 62.3 ppm; **^{19}F NMR** (470 MHz, DMSO-d_6, 25 °C): δ = -57.12 (s, 3F) ppm; **FT-IR** (neat): ν = 3172, 3045, 1662, 1581, 1312, 1133, 1113, 770 cm^{-1}; **HRMS** (ESI), *m*/*z*: [M + Na]$^+$ calcd for $C_{17}H_{11}F_3N_2NaO_2^+$ 355.0665; found 355.0669.

3-Mesityl-3a,9b-dihydroisoxazolo[4,5-*c*]isoquinolin-5(4*H*)-one (**11j**)

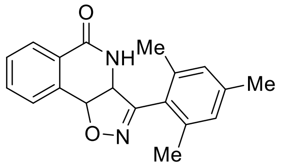

Yields: 75/-%; white solid; **m.p.** >270 °C (decomp.); $\boldsymbol{R_f}$ = 0.39 (*n*-hexane/EtOAc 2:1 *v/v*); **^{1}H NMR** (500 MHz, $CDCl_3$, 25 °C): δ = 8.12 (d, *J* = 7.8 Hz, 1H), 7.66 (d, *J* = 3.8 Hz, 2H), 7.55 (dt, *J* = 8.4, 4.1 Hz, 1H), 6.90 (s, 2H), 6.39 (br s, 1H), 5.76 (d, *J* = 9.2 Hz, 1H), 5.31 (dd, *J* = 9.3, 2.4 Hz, 1H), 2.29 (s, 3H), 2.19 (s, 6H) ppm; **^{13}C NMR** (125 MHz, $CDCl_3$, 25 °C): δ = 162.3, 157.4, 139.8, 137.4, 133.2, 132.7, 129.8, 129.2, 128.9, 128.0, 126.8, 123.0, 75.1, 62.5, 21.1, 20.0 ppm; **FT-IR** (neat): ν = 3180, 2956, 1665, 1602, 1233, 771 cm^{-1}; **HRMS** (ESI), *m*/*z*: [M + Na]$^+$ calcd for $C_{19}H_{18}N_2NaO_2^+$ 329.1260; found 329.1261.

3-(Perfluorophenyl)-3a,9b-dihydroisoxazolo[4,5-*c*]isoquinolin-5(4*H*)-one (**11k**)

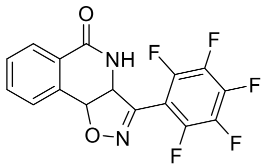

Yields: 48/49%; white solid; **m.p.** >260 °C (decomp.); $\boldsymbol{R_f}$ = 0.60 (*n*-hexane/EtOAc 1:1 *v/v*); **^{1}H NMR** (500 MHz, DMSO-d_6, 25 °C): δ = 8.53 (br s, 1H), 8.04 (d, *J* = 7.6 Hz, 1H), 7.75 – 7.70 (m, 2H), 7.66 – 7.59 (m, 1H), 5.91 (d, *J* = 8.8 Hz, 1H), 5.62 (d, *J* = 8.8 Hz, 1H) ppm; **^{13}C NMR** (125 MHz, DMSO-d_6, 25 °C): δ = 161.5, 133.4, 131.8, 130.6, 130.4, 127.7, 77.2, 60.1 ppm; **^{19}F NMR** (470 MHz, DMSO-d_6, 25 °C): δ = -137.67 (d, *J* = 18.6 Hz, 2F), -151.61 (t, *J* = 22.3 Hz, 1F), -161.93 (td, *J* = 21.9, 5.6 Hz, 2F) ppm; **FT-IR** (neat): ν = 3194, 2938, 1675, 1525, 1486, 1402, 1297, 977, 786, 754 cm^{-1}; **HRMS** (ESI), *m*/*z*: [M + Na]$^+$ calcd for $C_{16}H_7F_5N_2NaO_2^+$ 377.0320; found 377.0314.

3-(2,6-Dichlorophenyl)-3a,9b-dihydroisoxazolo[4,5-*c*]isoquinolin-5(4*H*)-one (**11l**)

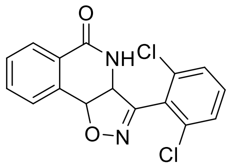

Yields: 49/49%; white solid; **m.p.** >260 °C (decomp.); $\boldsymbol{R_f}$ = 0.28 (*n*-hexane/EtOAc 1:1 *v/v*); **^{1}H NMR** (500 MHz, DMSO-d_6, 25 °C): δ = 8.50 (d, *J* = 3.6 Hz, 1H), 7.99 (dd, *J* = 7.8, 1.3 Hz, 1H), 7.72 – 7.54 (m, 6H), 6.09 (d, *J* = 9.9 Hz, 1H), 5.60 (dd, *J* = 9.9, 3.7 Hz, 1H) ppm; **^{13}C NMR** (125 MHz, DMSO-d_6, 25 °C): δ = 161.8, 156.0, 135.2, 134.2, 133.1, 133.0, 129.8, 129.2, 129.0, 127.9, 127.1, 126.8, 75.1, 61.5 ppm; **FT-IR** (neat): ν = 3189, 1668, 1428, 1407, 1304, 1189, 782 cm^{-1}; **HRMS** (ESI), *m*/*z*: [M + Na]$^+$ calcd for $C_{16}H_{10}Cl_2N_2NaO_2^+$ 355.0012; found 355.0010.

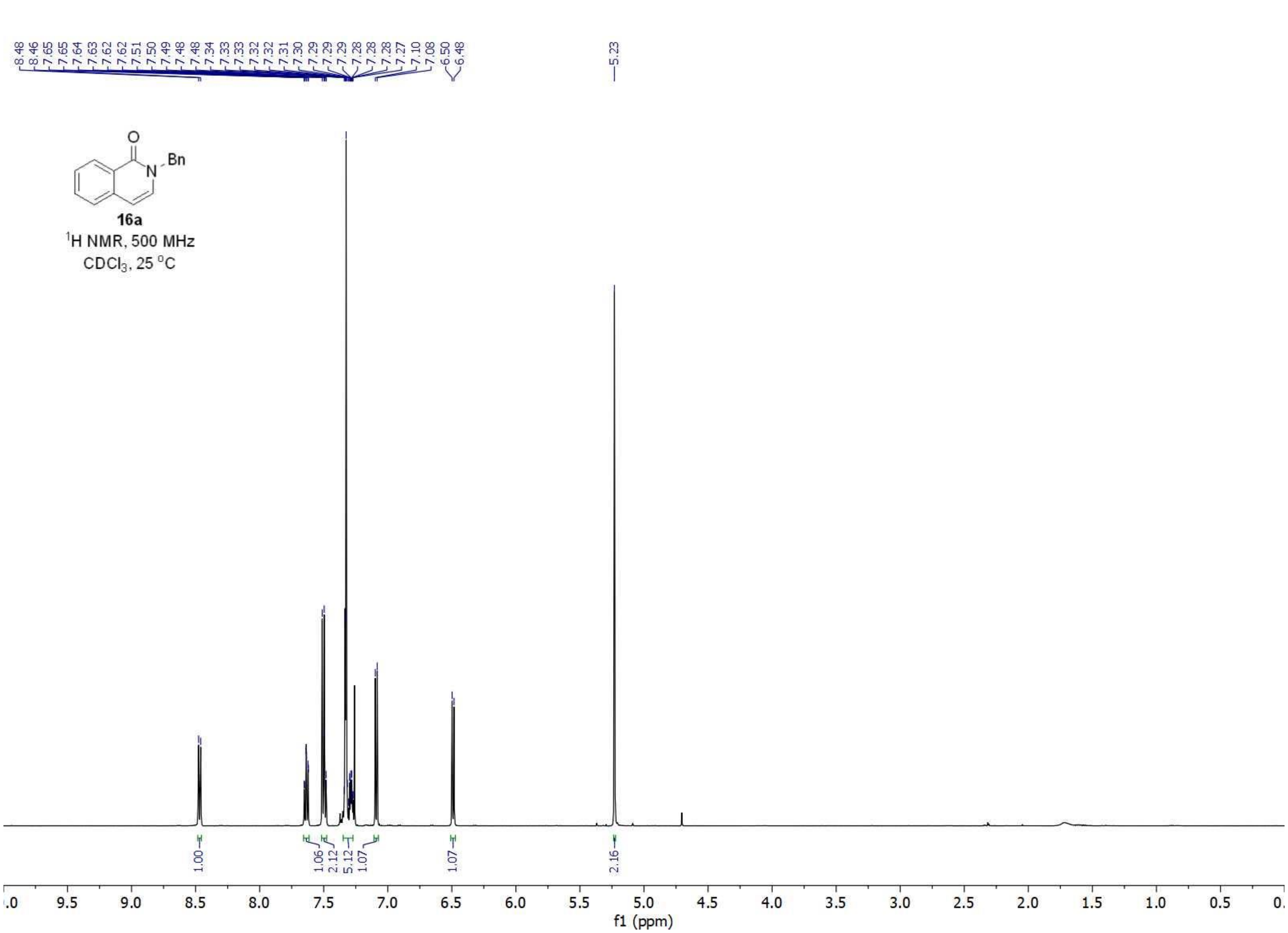
8.48 8.46 7.65 7.65 7.64 7.63 7.62 7.62 7.51 7.50 7.49 7.48 7.48 7.34 7.33 7.33 7.32 7.32 7.31 7.30 7.29 7.29 7.29 7.28 7.28 7.28 7.27 7.10 7.08 6.50 6.48
5.23
O
N
Bn
16a
^{1}H NMR, 500 MHz
$CDCl_3$, 25 °C
1.00
1.06
2.12
5.12
1.07
1.07
2.16
9.5 9.0 8.5 8.0 7.5 7.0 6.5 6.0 5.5 5.0 4.5 4.0 3.5 3.0 2.5 2.0 1.5 1.0 0.5
f1 (ppm)

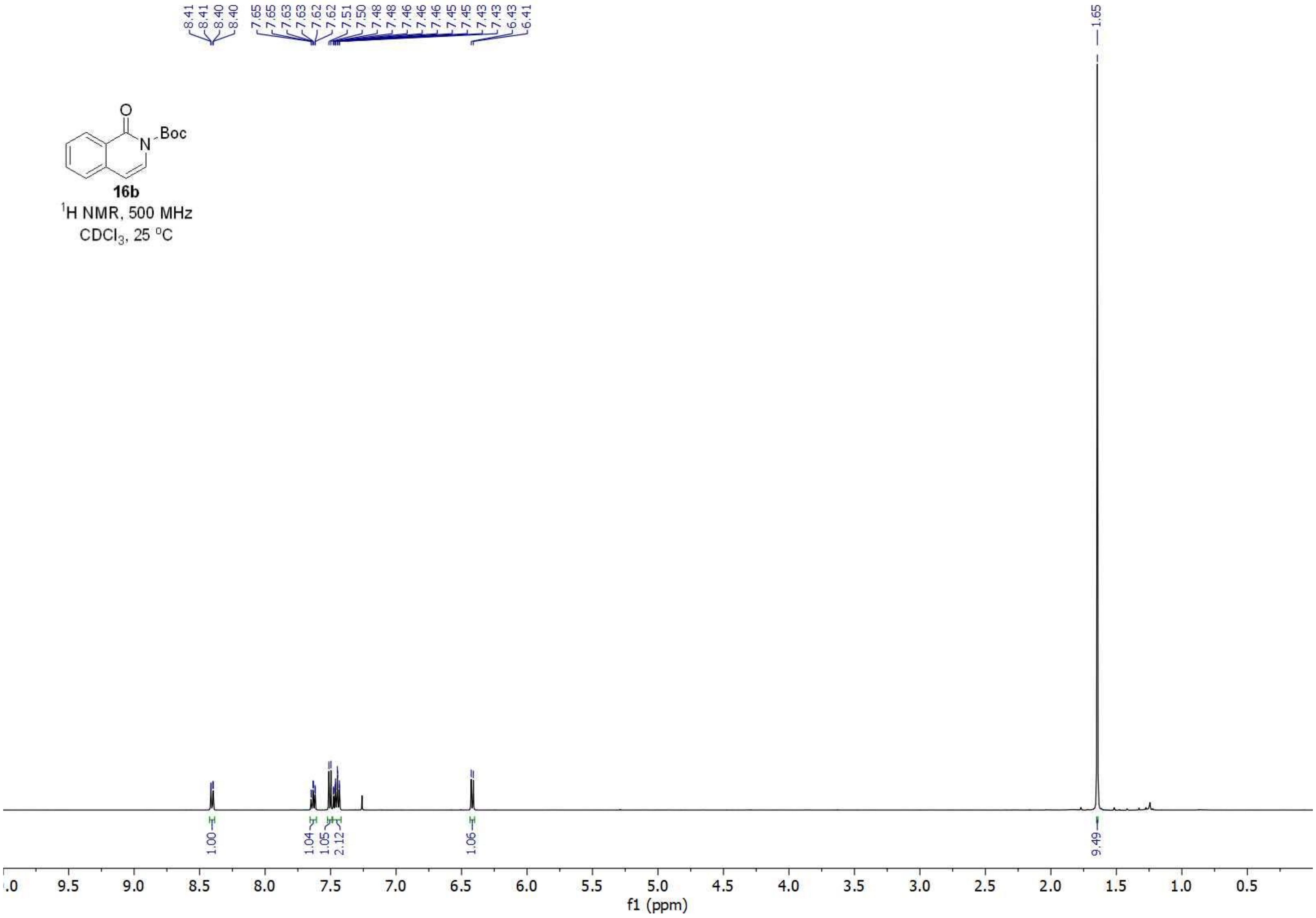
8.41
8.41
8.40
8.40
7.65
7.65
7.63
7.63
7.62
7.62
7.51
7.50
7.48
7.48
7.46
7.46
7.45
7.45
7.43
7.43
6.43
6.41
1.65
O
N
Boc
16b
¹H NMR, 500 MHz
CDCl3, 25 °C
1.00
1.04
1.05
2.12
1.06
9.49
9.5
9.0
8.5
8.0
7.5
7.0
6.5
6.0
5.5
5.0
4.5
4.0
3.5
3.0
2.5
2.0
1.5
1.0
0.5
f1 (ppm)

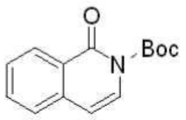

16b
^{13}C NMR, 125 MHz
$CDCl_3$, 25 °C

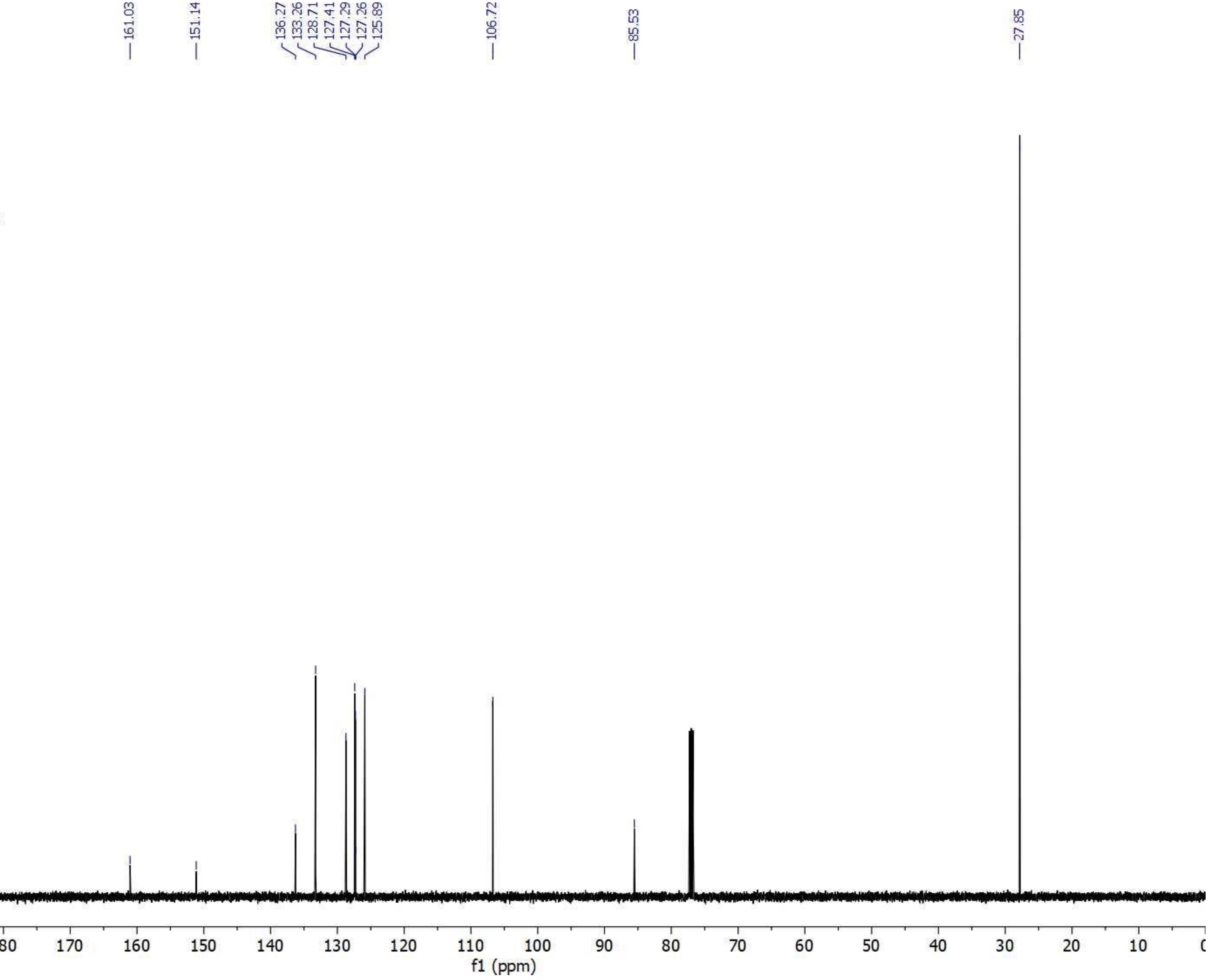

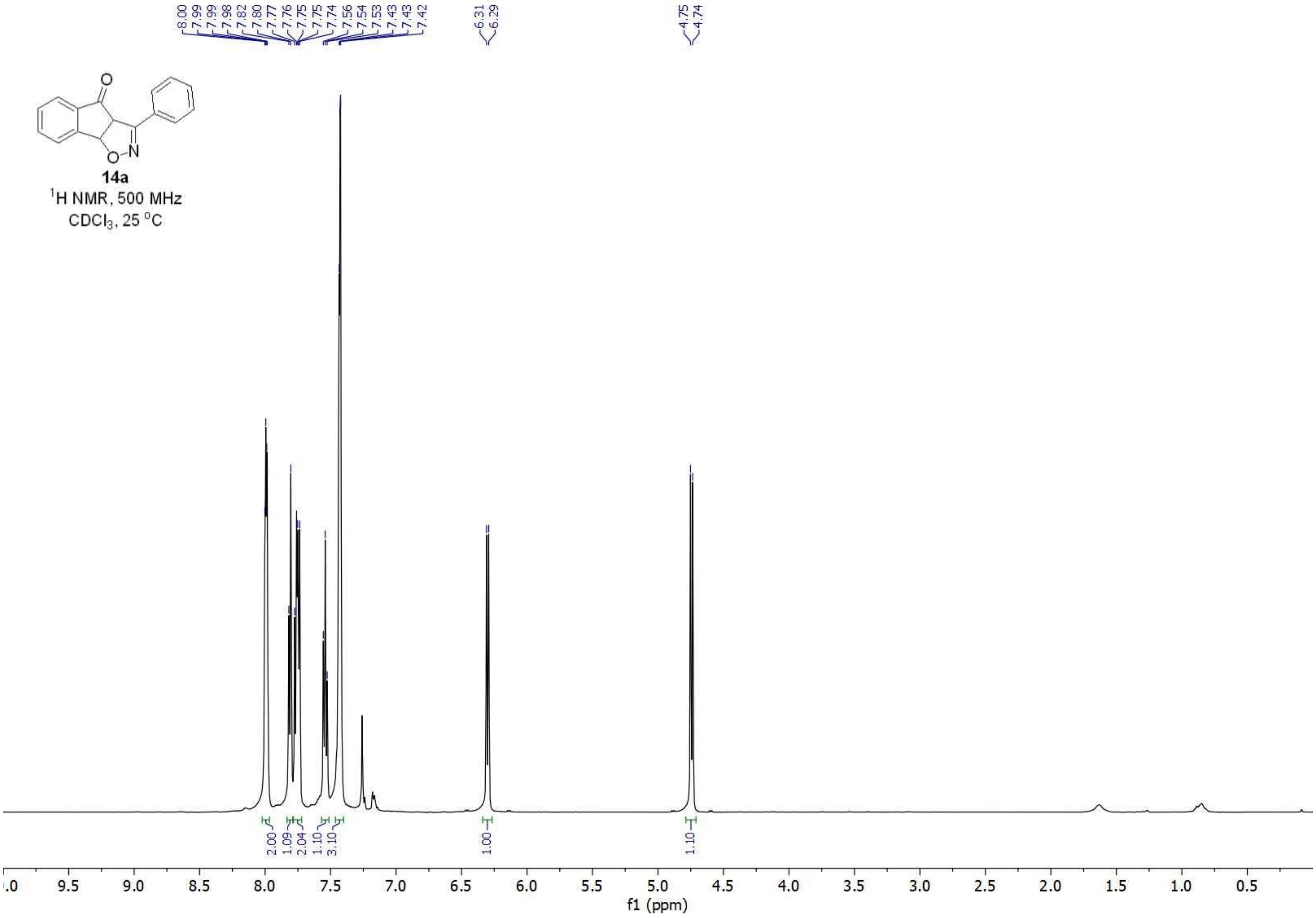

8.00
7.99
7.99
7.98
7.82
7.80
7.77
7.76
7.75
7.75
7.74
7.56
7.54
7.53
7.43
7.43
7.42
6.31
6.29
4.75
4.74
14a
^{1}H NMR, 500 MHz
CDCl$_3$, 25 ^{o}C
2.00
1.09
2.04
1.10
3.10
1.00
1.10
f1 (ppm)

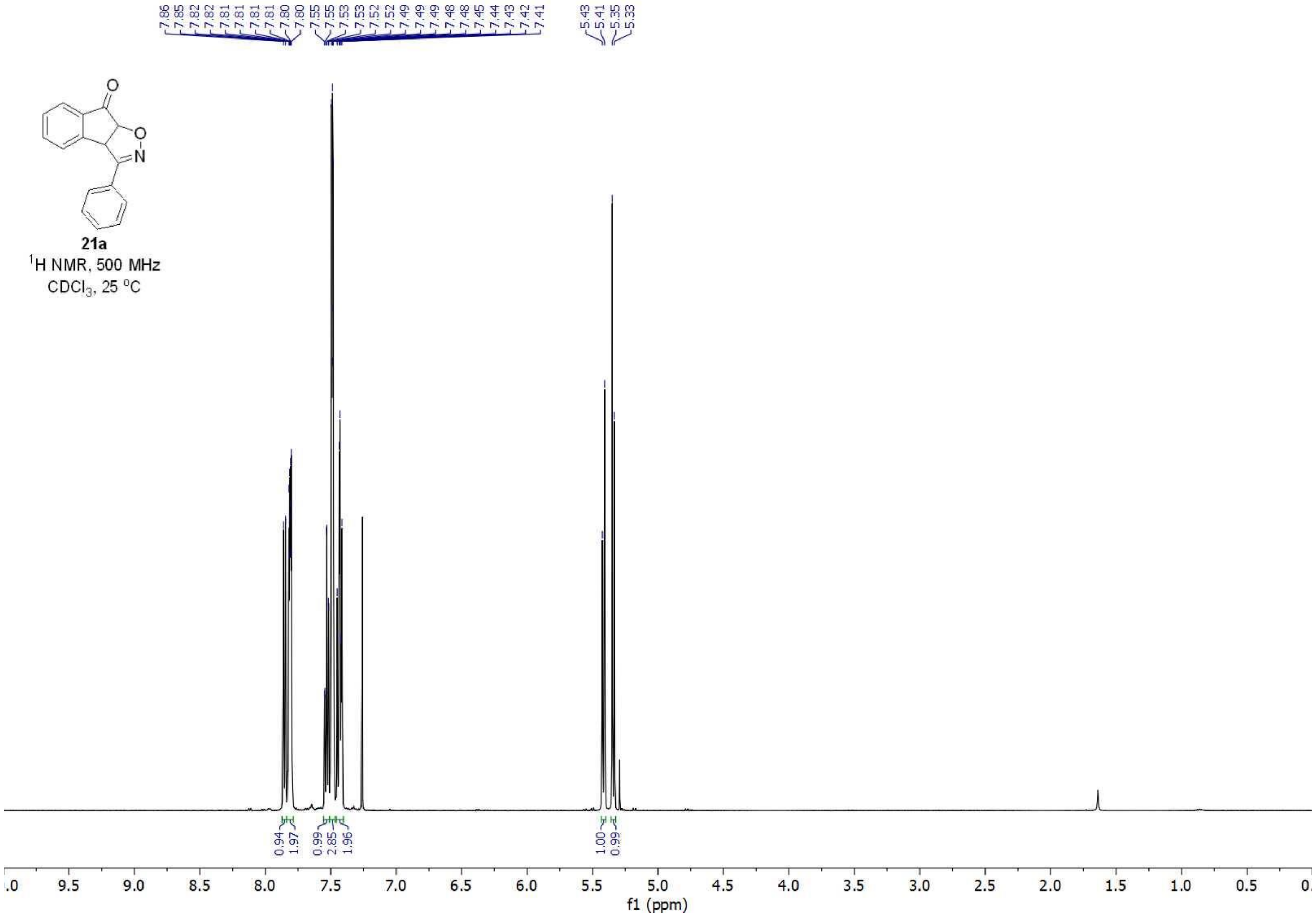
7.86 7.85 7.82 7.81 7.81 7.81 7.80 7.80 7.55 7.55 7.53 7.53 7.52 7.52 7.49 7.49 7.48 7.48 7.45 7.44 7.43 7.42 7.41
5.43 5.41 5.35 5.33
O
O
N
21a
^{1}H NMR, 500 MHz
$CDCl_3$, 25 ^oC
0.94 1.97 0.99 2.85 1.96
1.00 0.99
.0 9.5 9.0 8.5 8.0 7.5 7.0 6.5 6.0 5.5 5.0 4.5 4.0 3.5 3.0 2.5 2.0 1.5 1.0 0.5 0.
f1 (ppm)

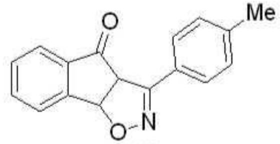

14b

^{1}H NMR, 500 MHz

$CDCl_3$, 25 °C

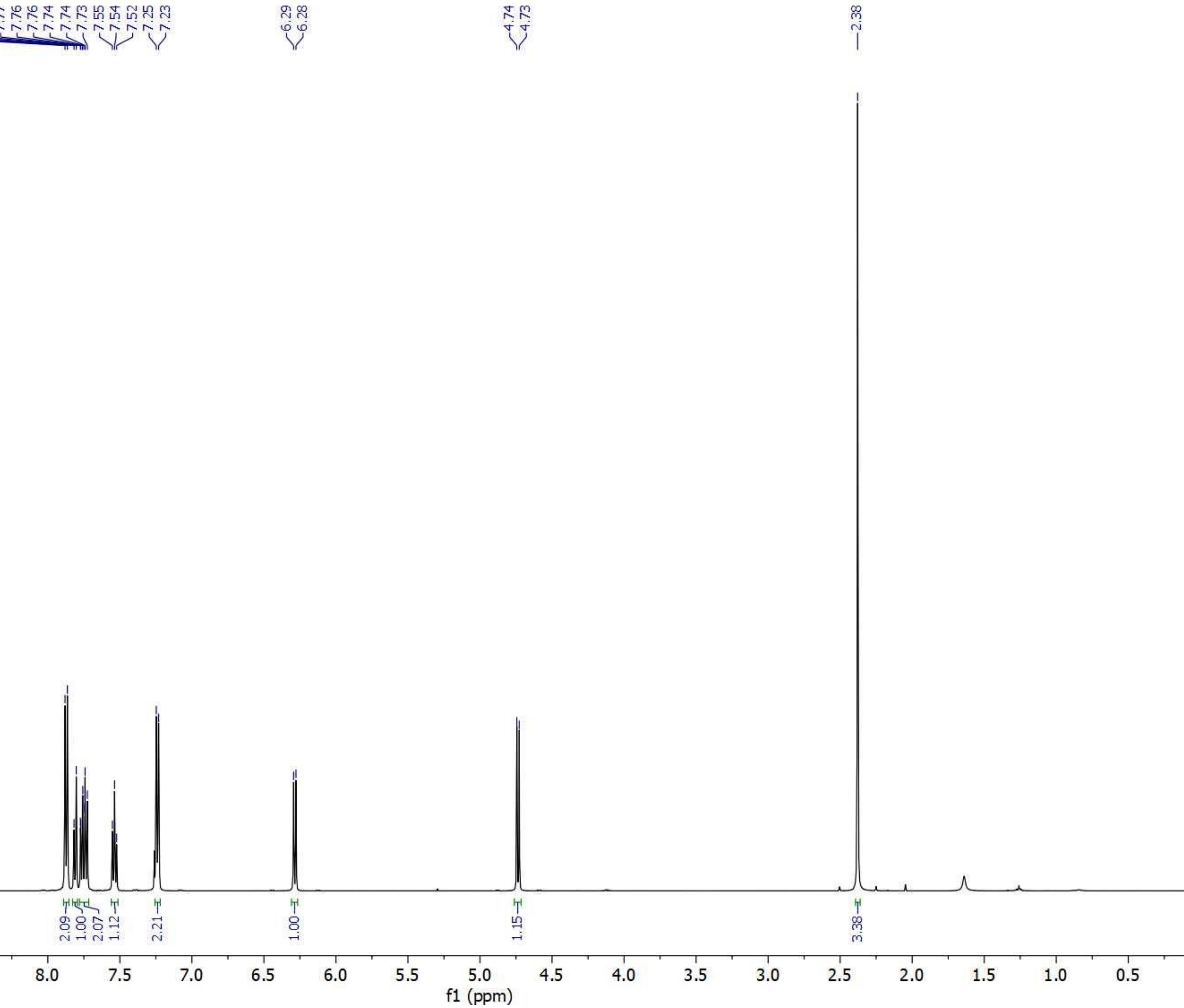

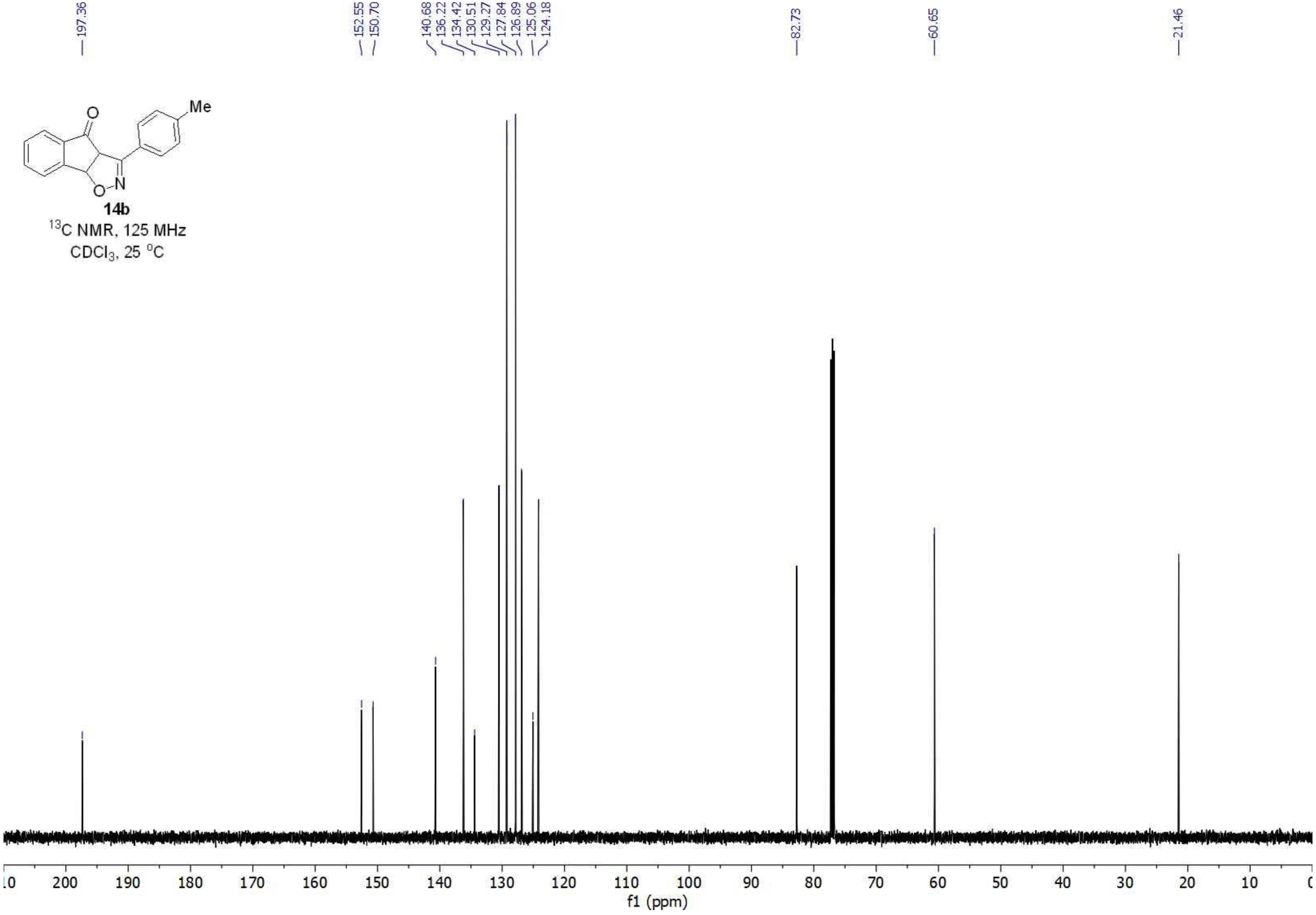
197.36
152.55
150.70
140.68
136.22
134.42
130.51
129.27
127.84
126.89
125.06
124.18
82.73
60.65
21.46
Me
O
O
N
14b
^{13}C NMR, 125 MHz
$CDCl_3$, 25 °C
200
190
180
170
160
150
140
130
120
110
100
90
80
70
60
50
40
30
20
10
f1 (ppm)

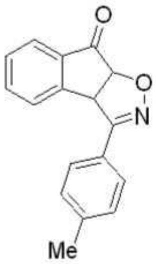

21b
^{1}H NMR, 500 MHz
$CDCl_3$, 25 ^oC

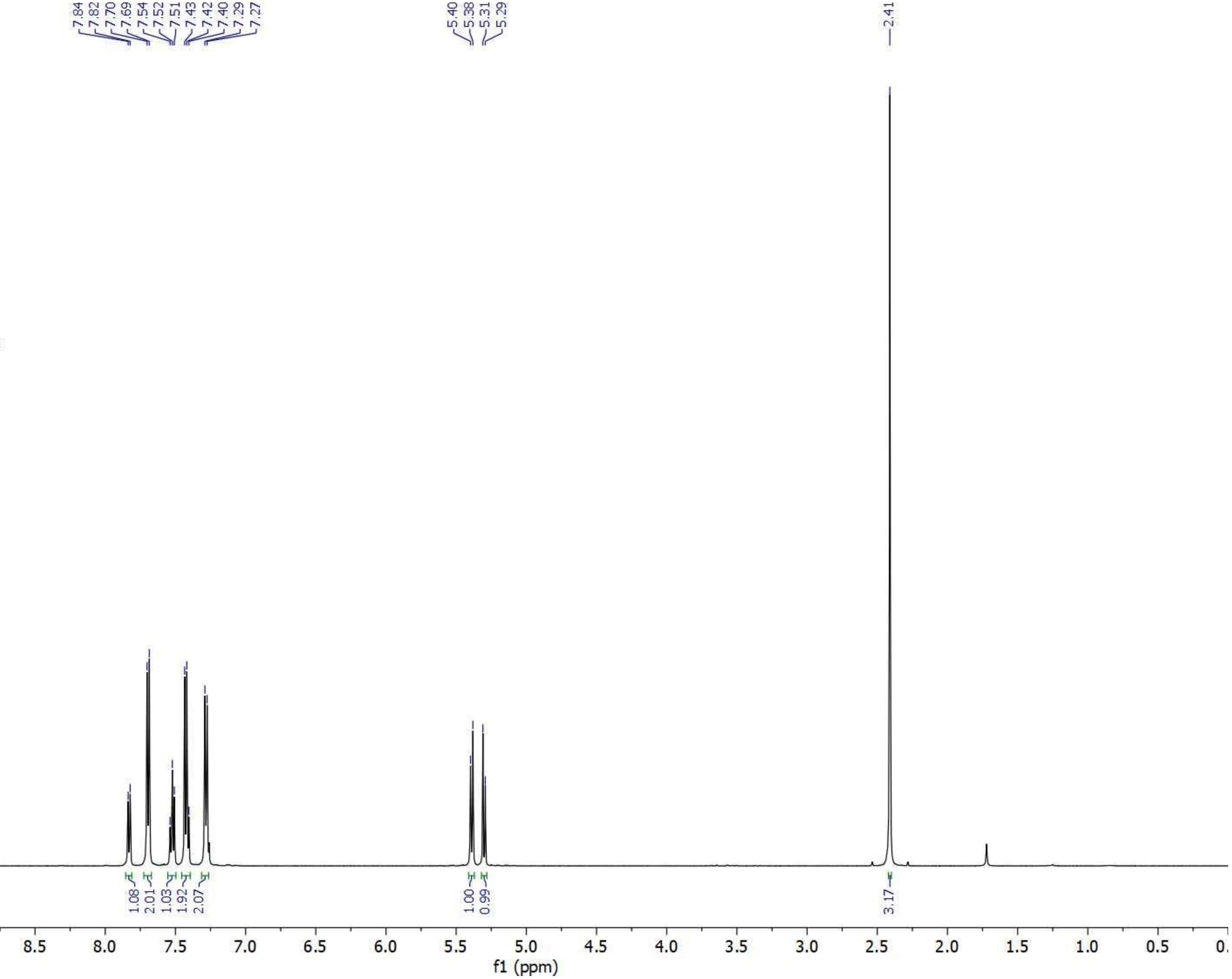

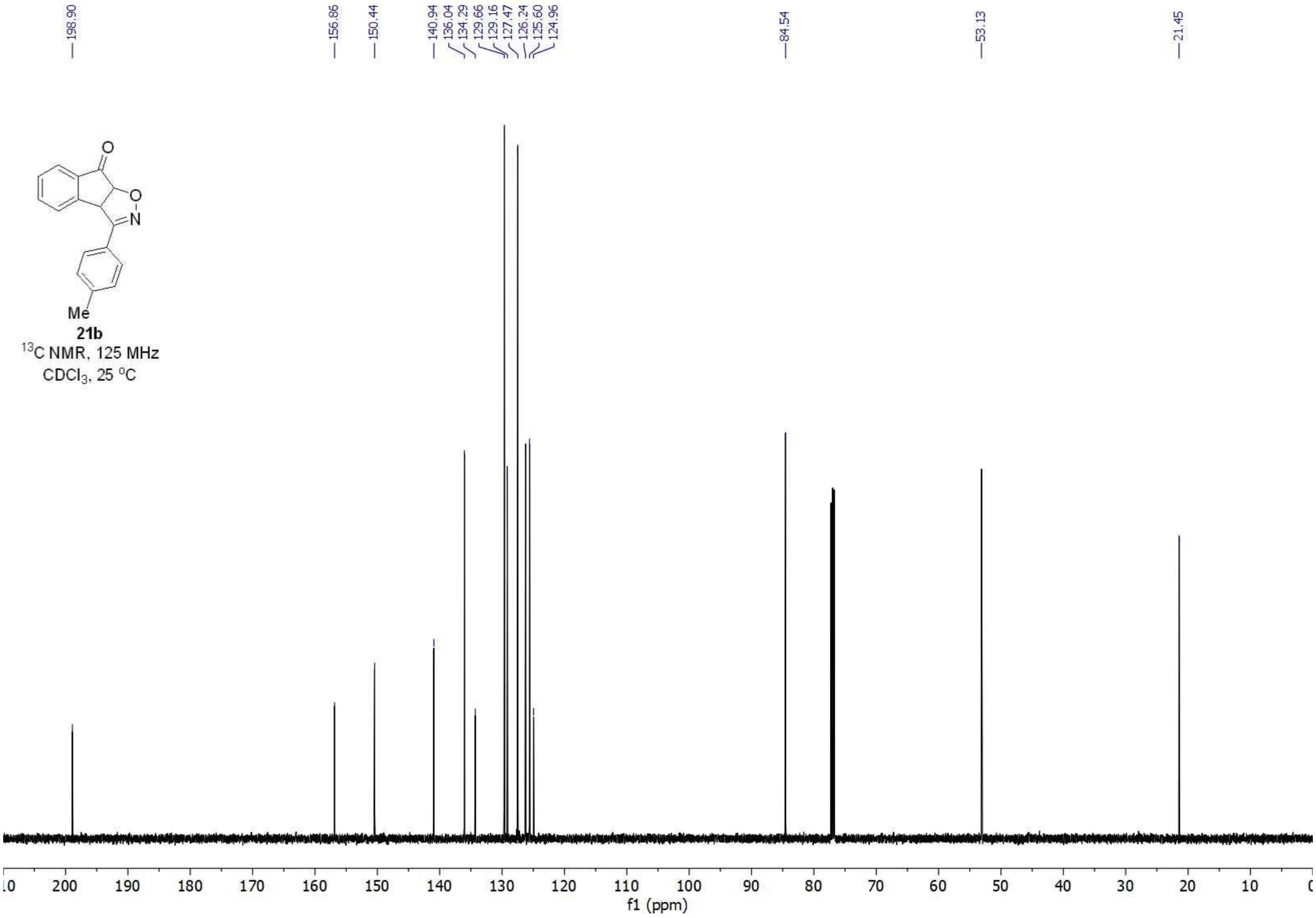

21b

^{13}C NMR, 125 MHz

$CDCl_3$, 25 °C

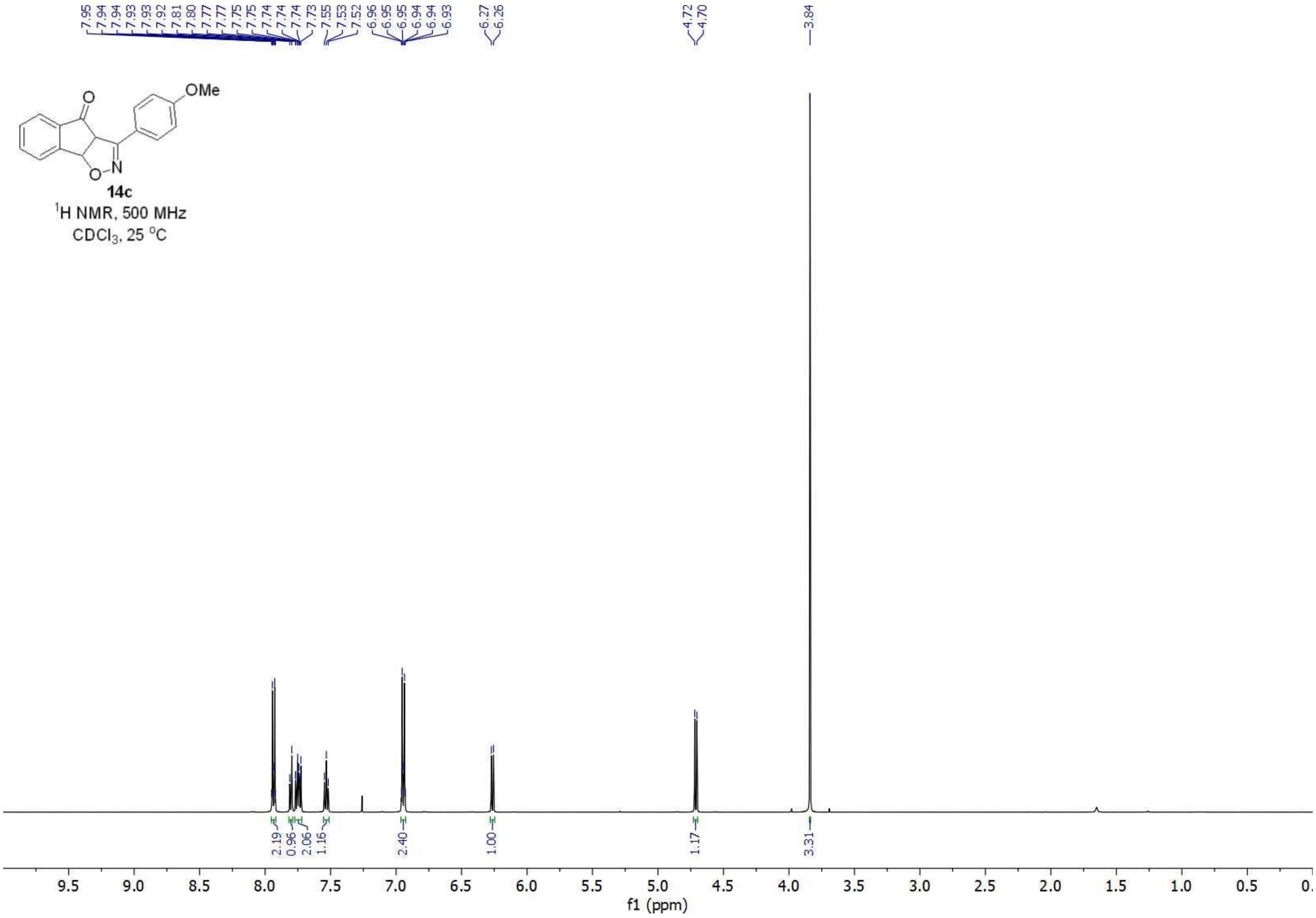
7.95 7.94 7.94 7.93 7.93 7.92 7.81 7.80 7.77 7.77 7.75 7.75 7.74 7.74 7.74 7.73 7.55 7.53 7.52 6.96 6.95 6.95 6.94 6.94 6.93
6.27 6.26
4.72 4.70
3.84
OMe
O
N
14c
^{1}H NMR, 500 MHz
CDCl$_3$, 25 °C
2.19 0.96 2.06 1.16
2.40
1.00
1.17
3.31
9.5 9.0 8.5 8.0 7.5 7.0 6.5 6.0 5.5 5.0 4.5 4.0 3.5 3.0 2.5 2.0 1.5 1.0 0.5 0.
f1 (ppm)

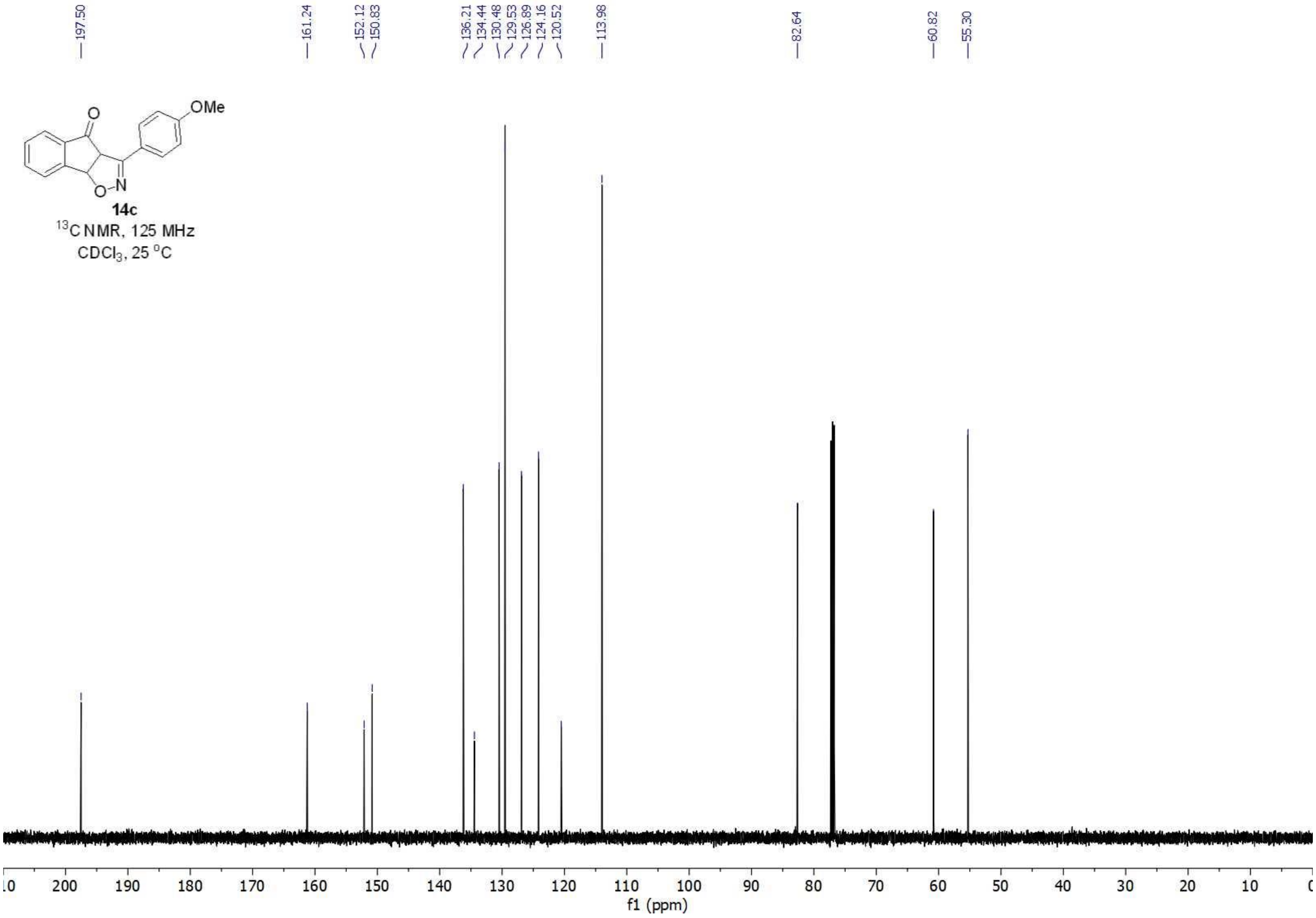
197.50
161.24
152.12
150.83
136.21
134.44
130.48
129.53
126.89
124.16
120.52
113.98
82.64
60.82
55.30
OMe
O
O
N
14c
^{13}C NMR, 125 MHz
$CDCl_3$, 25 °C
200
190
180
170
160
150
140
130
120
110
100
90
80
70
60
50
40
30
20
10
f1 (ppm)

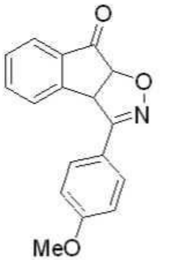

21c

^{1}H NMR, 500 MHz

$CDCl_3$, 25 °C

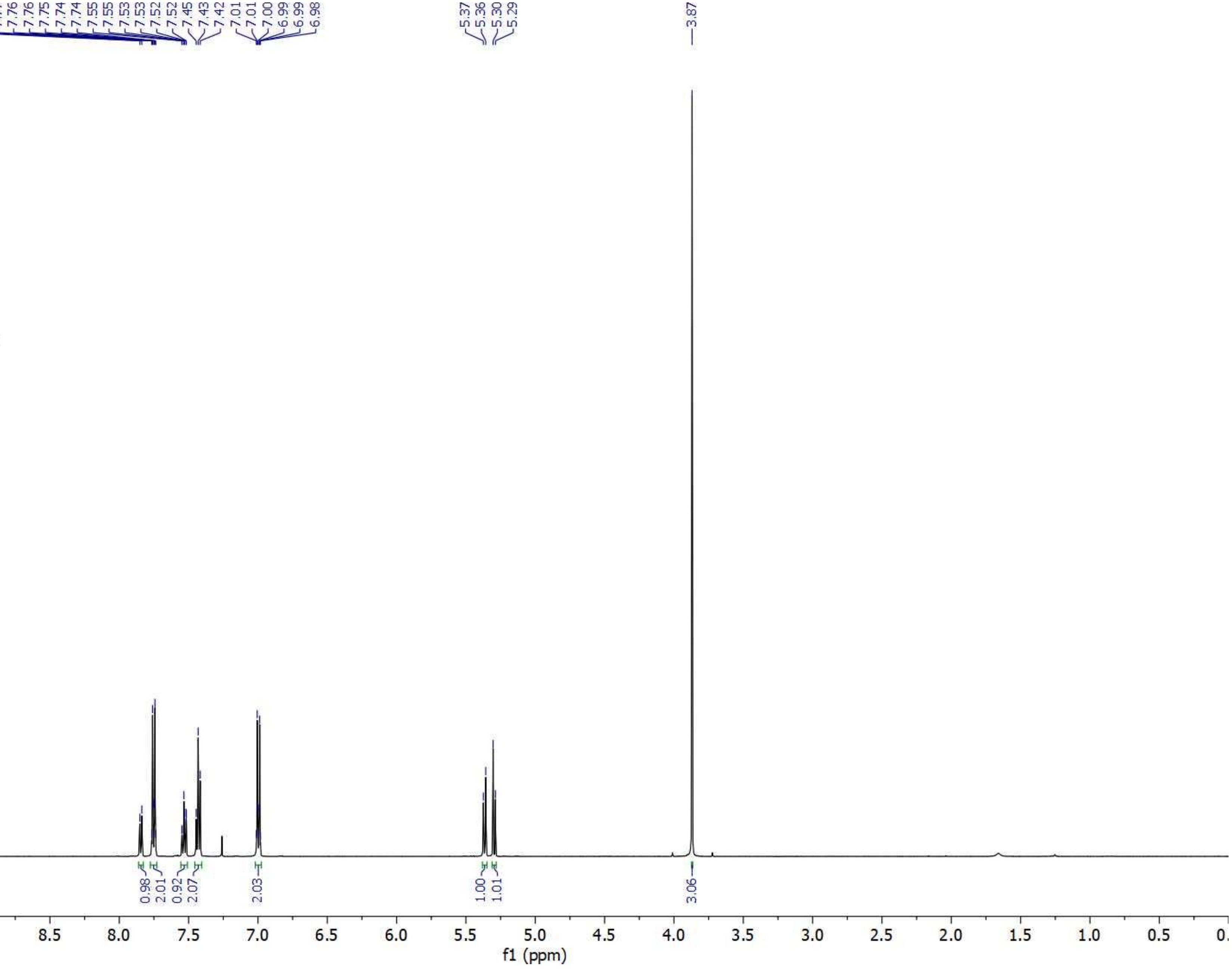

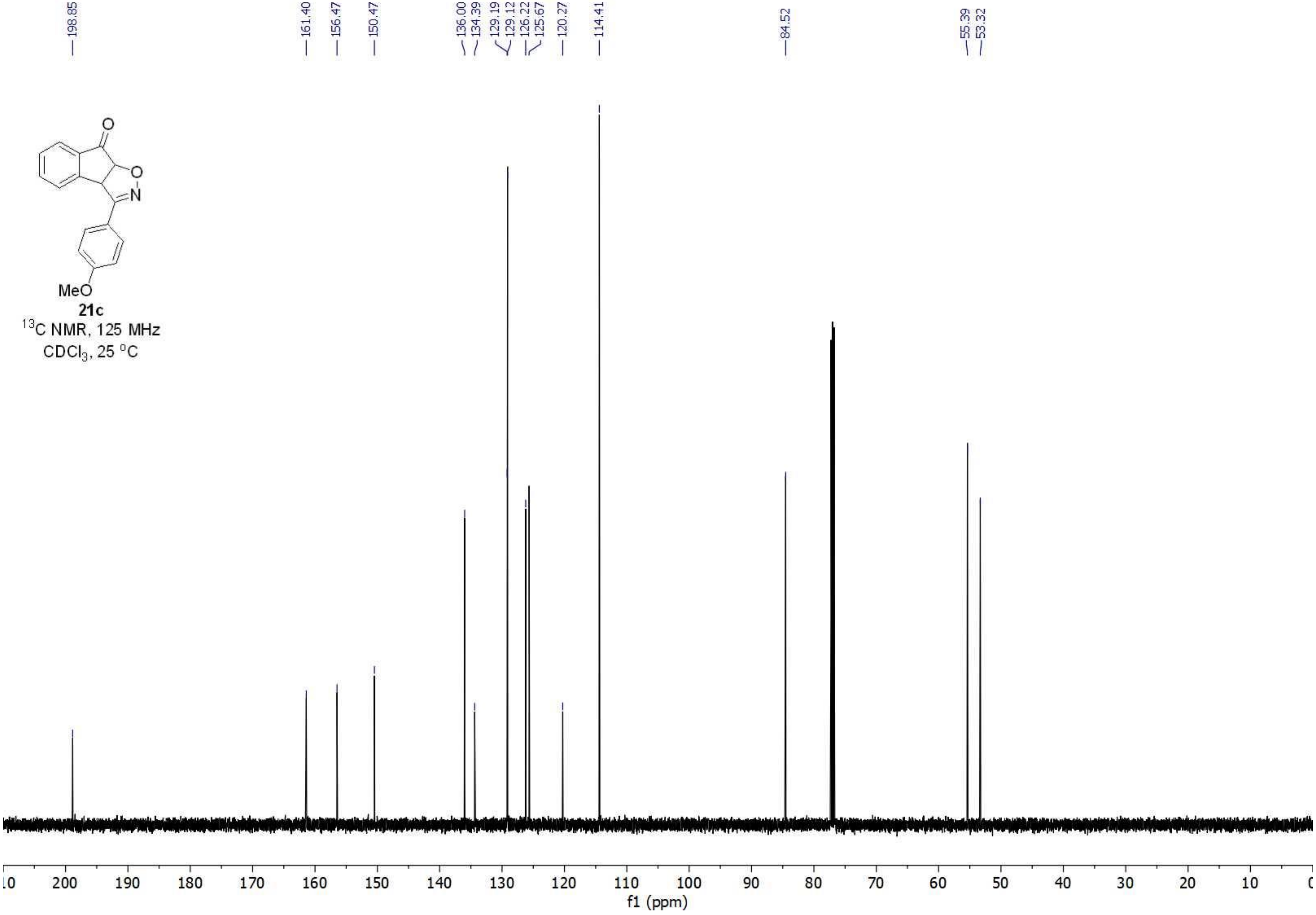
198.85
161.40
156.47
150.47
136.00
134.39
129.19
129.12
126.22
125.67
120.27
114.41
84.52
55.39
53.32
O
O
N
MeO
21c
13C NMR, 125 MHz
CDCl3, 25 °C
200
190
180
170
160
150
140
130
120
110
100
90
80
70
60
50
40
30
20
10
f1 (ppm)

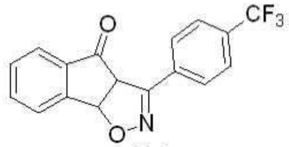

14d

^{1}H NMR, 500 MHz

$CDCl_3$, 25 ^{o}C

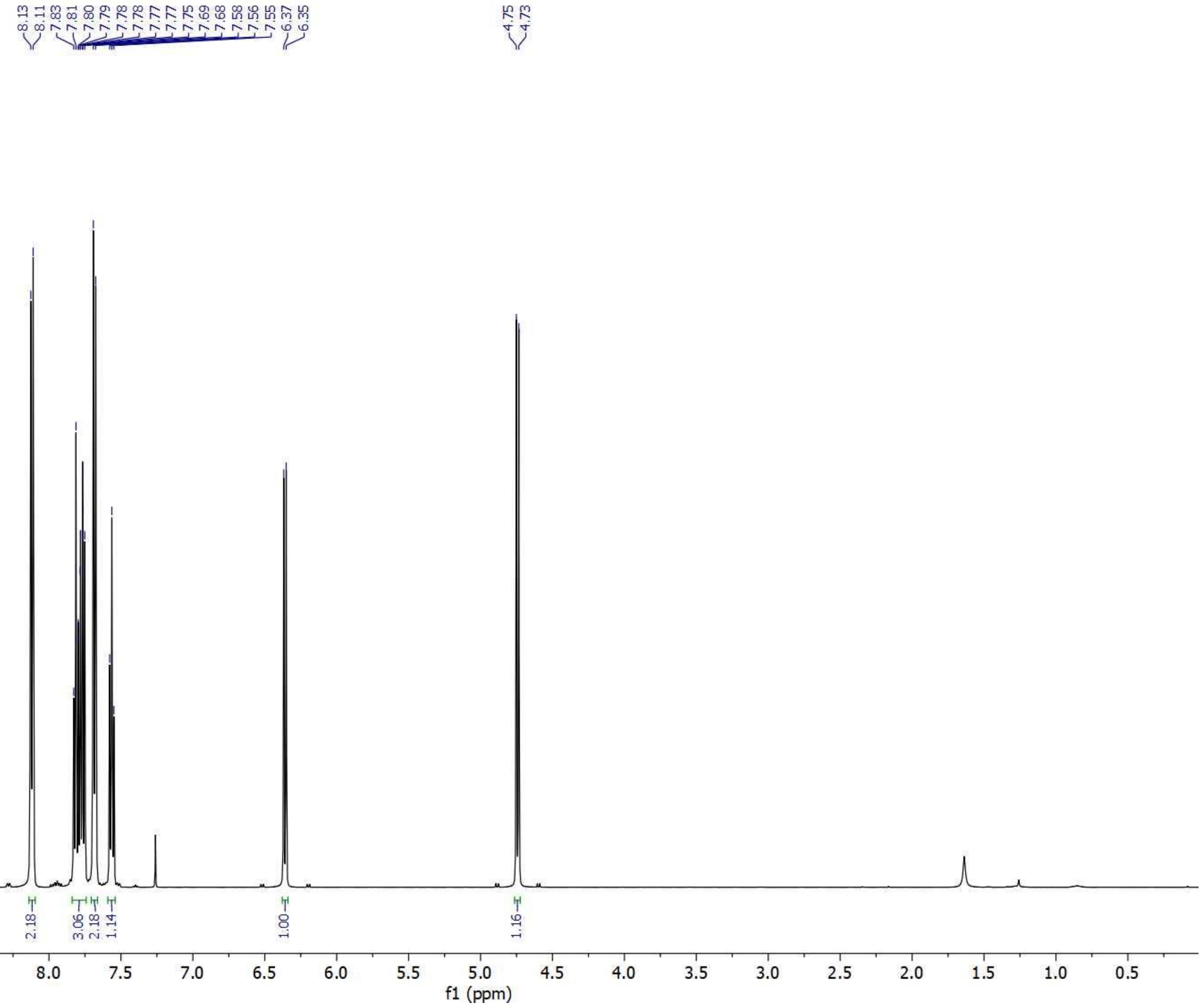

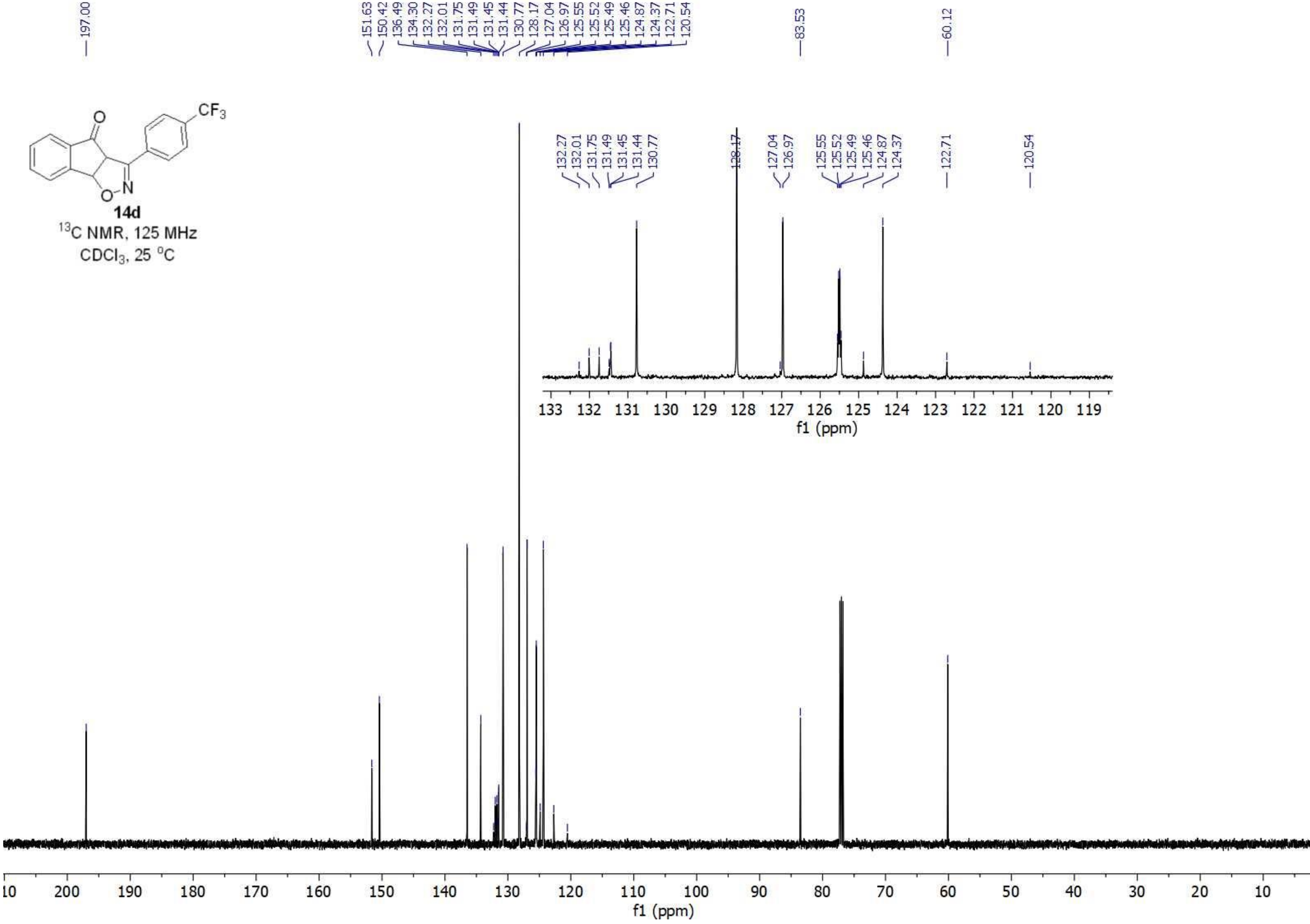
197.00
151.63
150.42
136.49
134.30
132.27
132.01
131.75
131.49
131.45
131.44
130.77
128.17
127.04
126.97
125.55
125.52
125.49
125.46
124.87
124.37
122.71
120.54
83.53
60.12
CF3
O
O
N
14d
13C NMR, 125 MHz
CDCl3, 25 °C
133 132 131 130 129 128 127 126 125 124 123 122 121 120 119
f1 (ppm)
10 200 190 180 170 160 150 140 130 120 110 100 90 80 70 60 50 40 30 20 10
f1 (ppm)

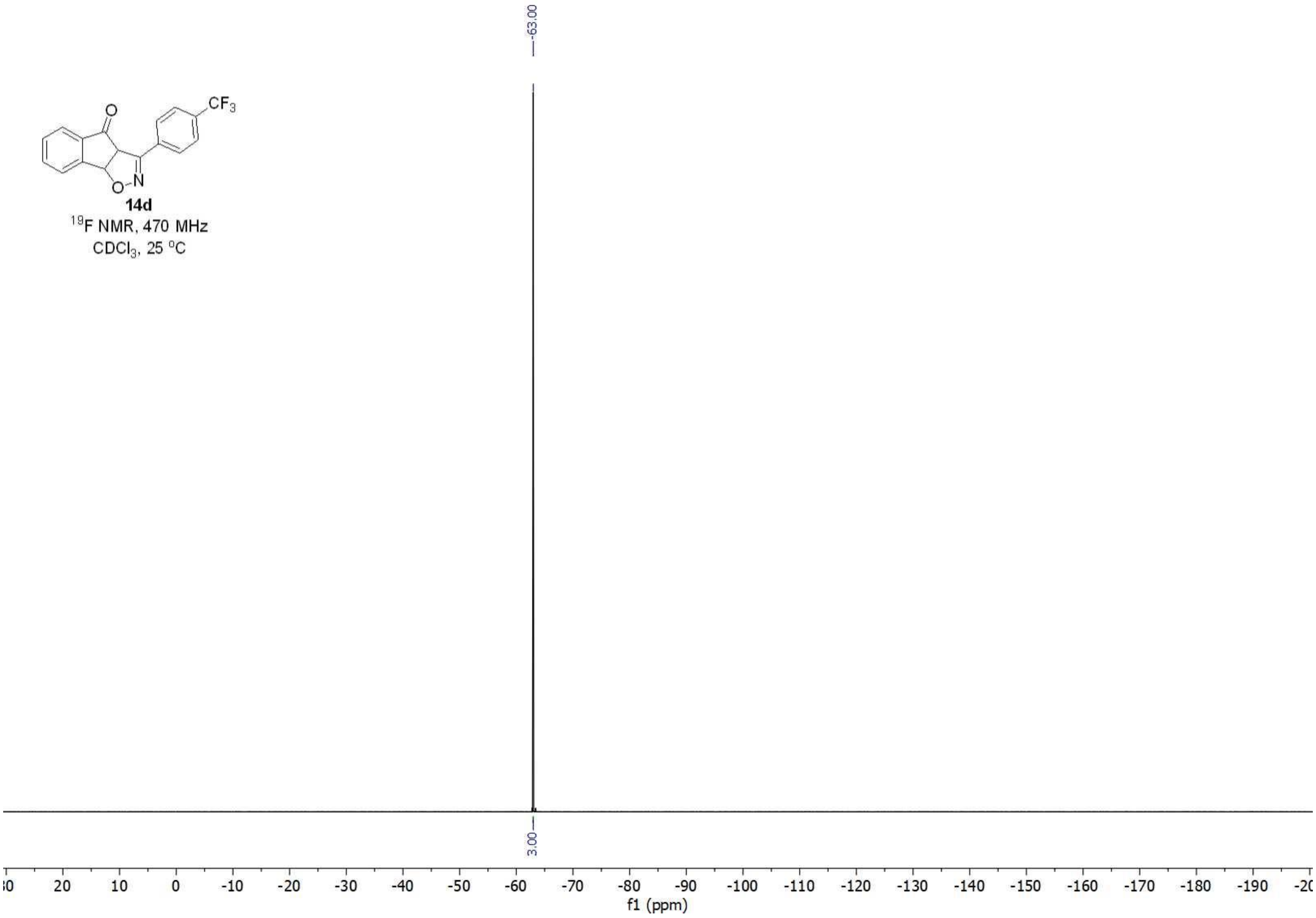

-63.00
CF3
O
O
N
14d
19F NMR, 470 MHz
CDCl3, 25 °C
3.00
20
10
0
-10
-20
-30
-40
-50
-60
-70
-80
-90
-100
-110
-120
-130
-140
-150
-160
-170
-180
-190
f1 (ppm)

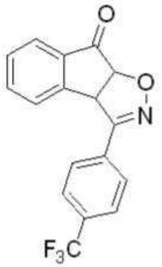

21d

^1H NMR, 500 MHz

$CDCl_3$, 25 ^oC

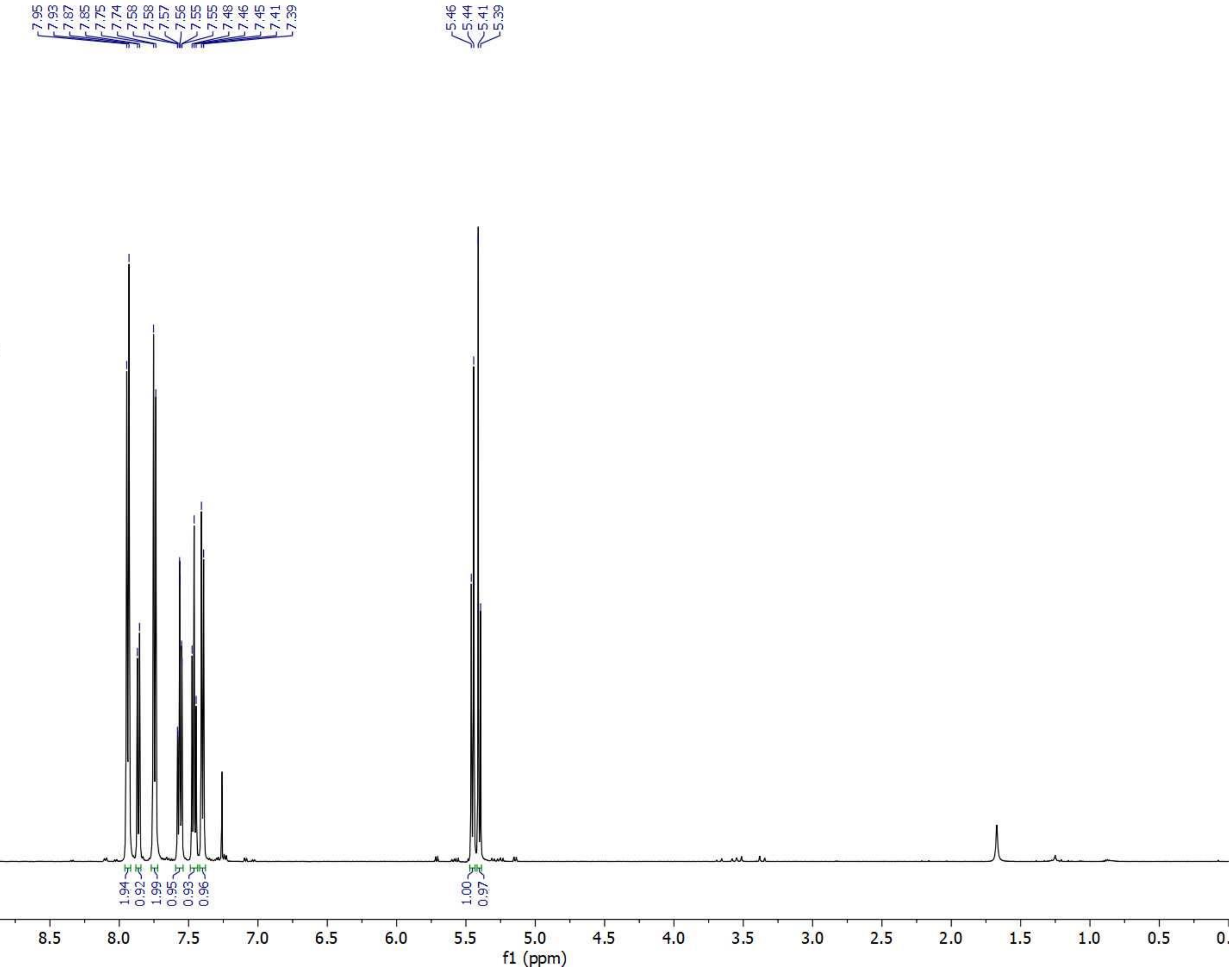

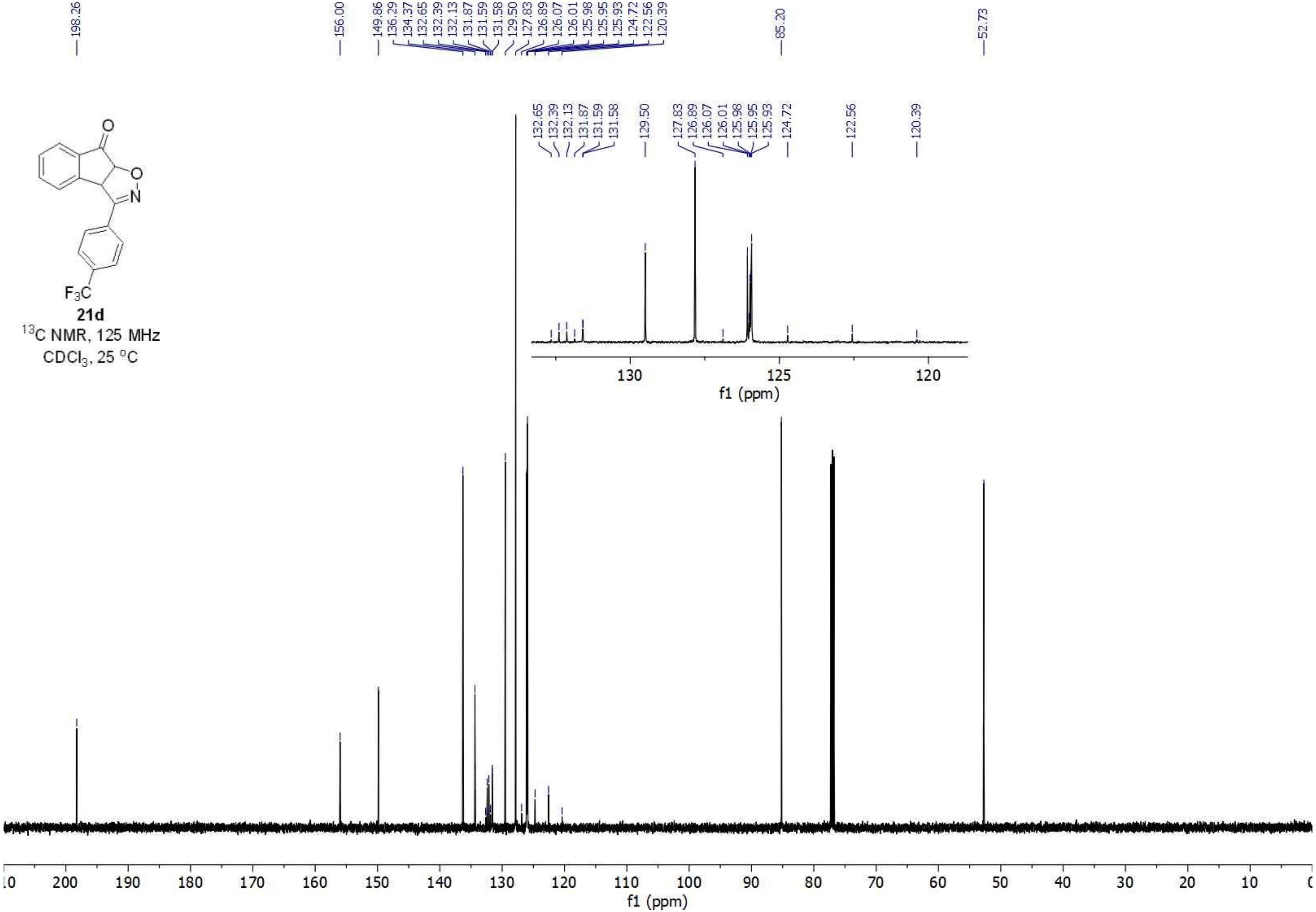

21d

^{13}C NMR, 125 MHz

$CDCl_3$, 25 °C

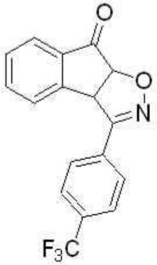

21d
^{19}F NMR, 470 MHz
$CDCl_3$, 25 ^{o}C

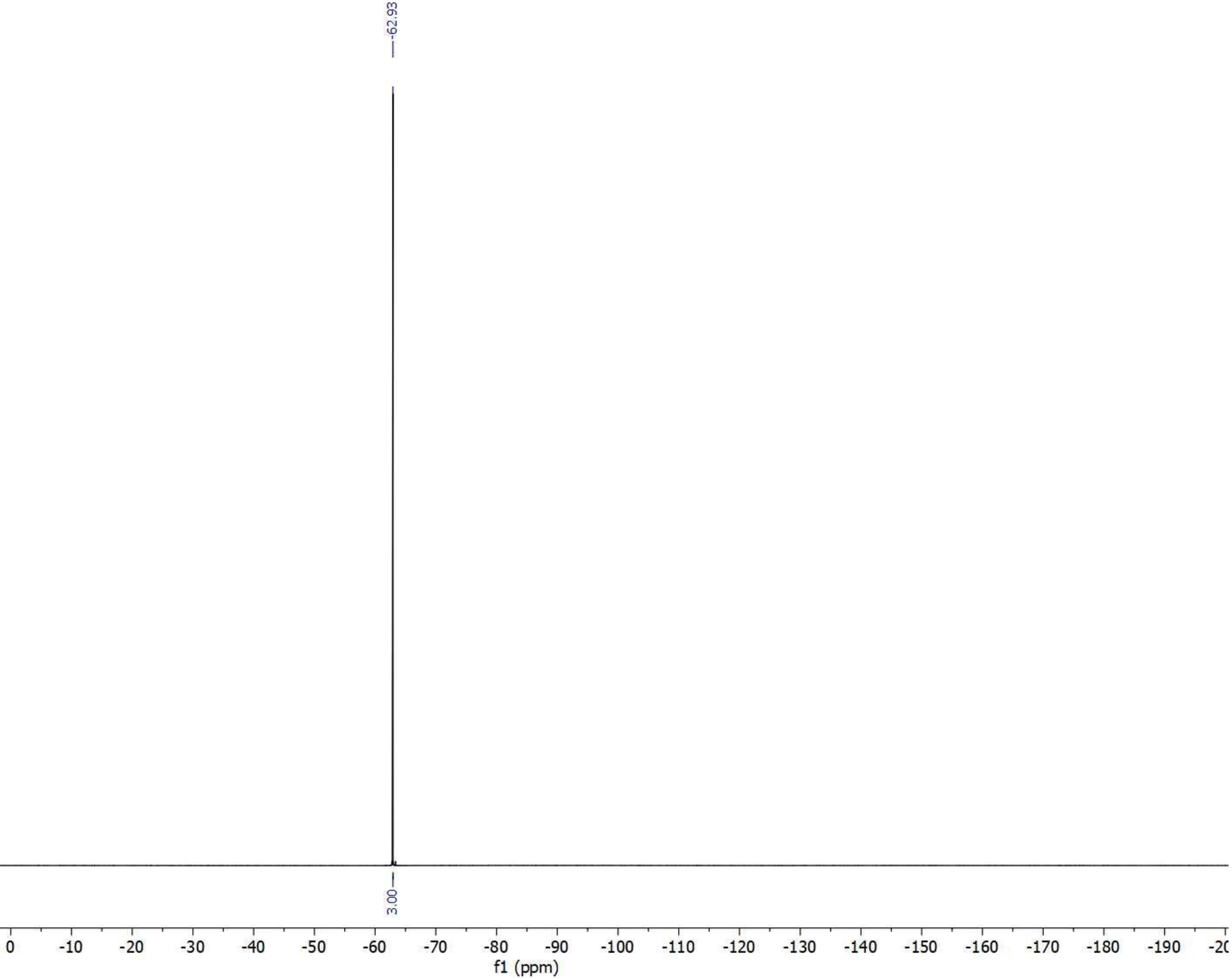

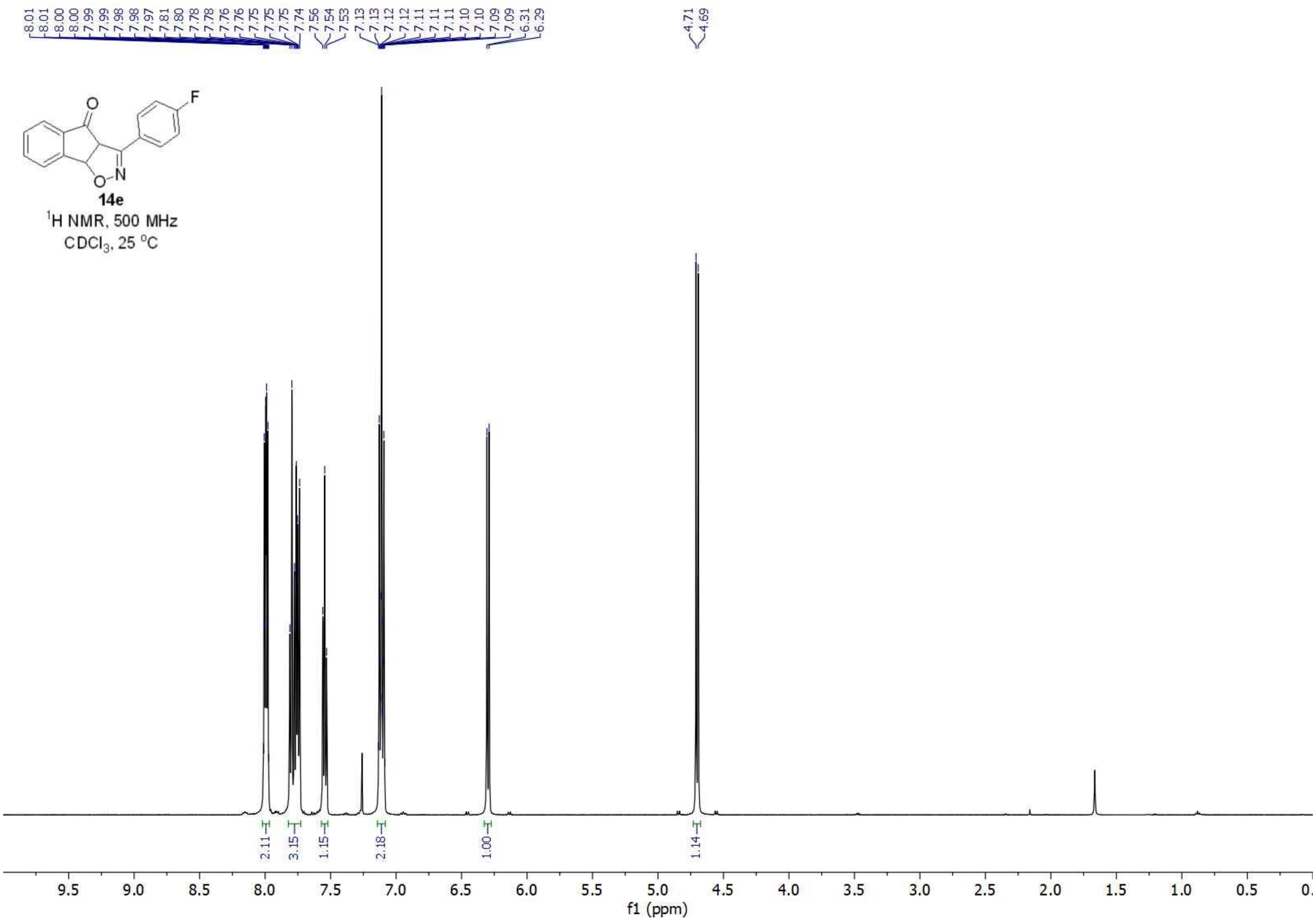
8.01 8.01 8.00 7.99 7.99 7.98 7.98 7.97 7.81 7.80 7.78 7.78 7.76 7.76 7.75 7.75 7.75 7.74 7.56 7.54 7.53 7.13 7.13 7.12 7.11 7.11 7.11 7.10 7.09 7.09 6.31 6.29
4.71 4.69
F
O
N
14e
^{1}H NMR, 500 MHz
$CDCl_3$, 25 °C
2.11 3.15 1.15 2.18 1.00 1.14
9.5 9.0 8.5 8.0 7.5 7.0 6.5 6.0 5.5 5.0 4.5 4.0 3.5 3.0 2.5 2.0 1.5 1.0 0.5 0.
f1 (ppm)

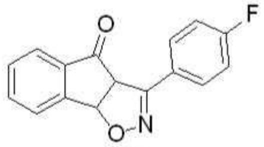

14e

^{13}C NMR, 125 MHz

$CDCl_3$, 25 °C

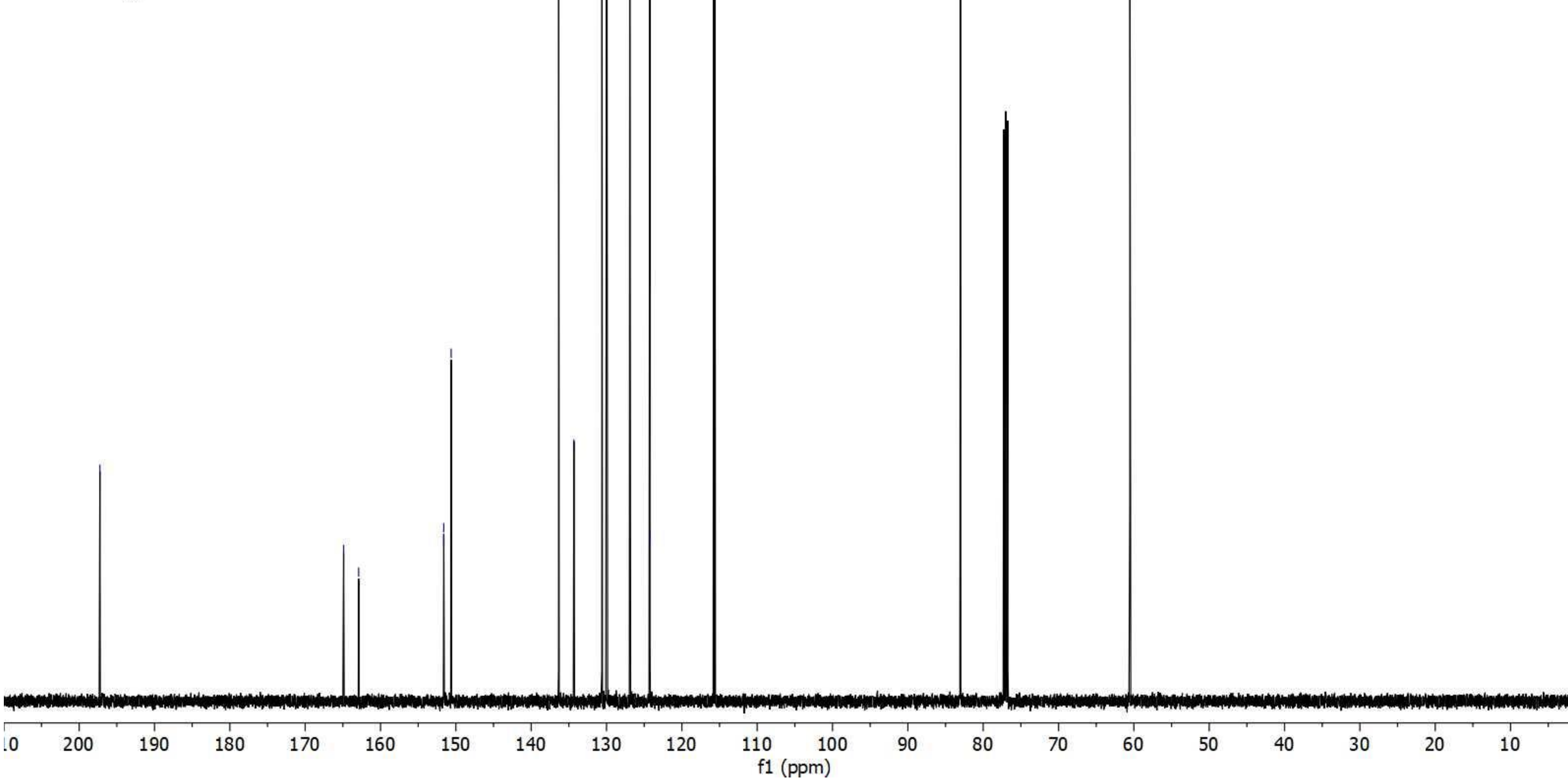

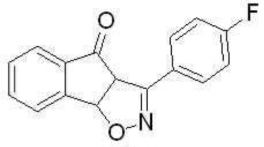

14e

^{19}F NMR, 470 MHz

$CDCl_3$, 25 ^{o}C

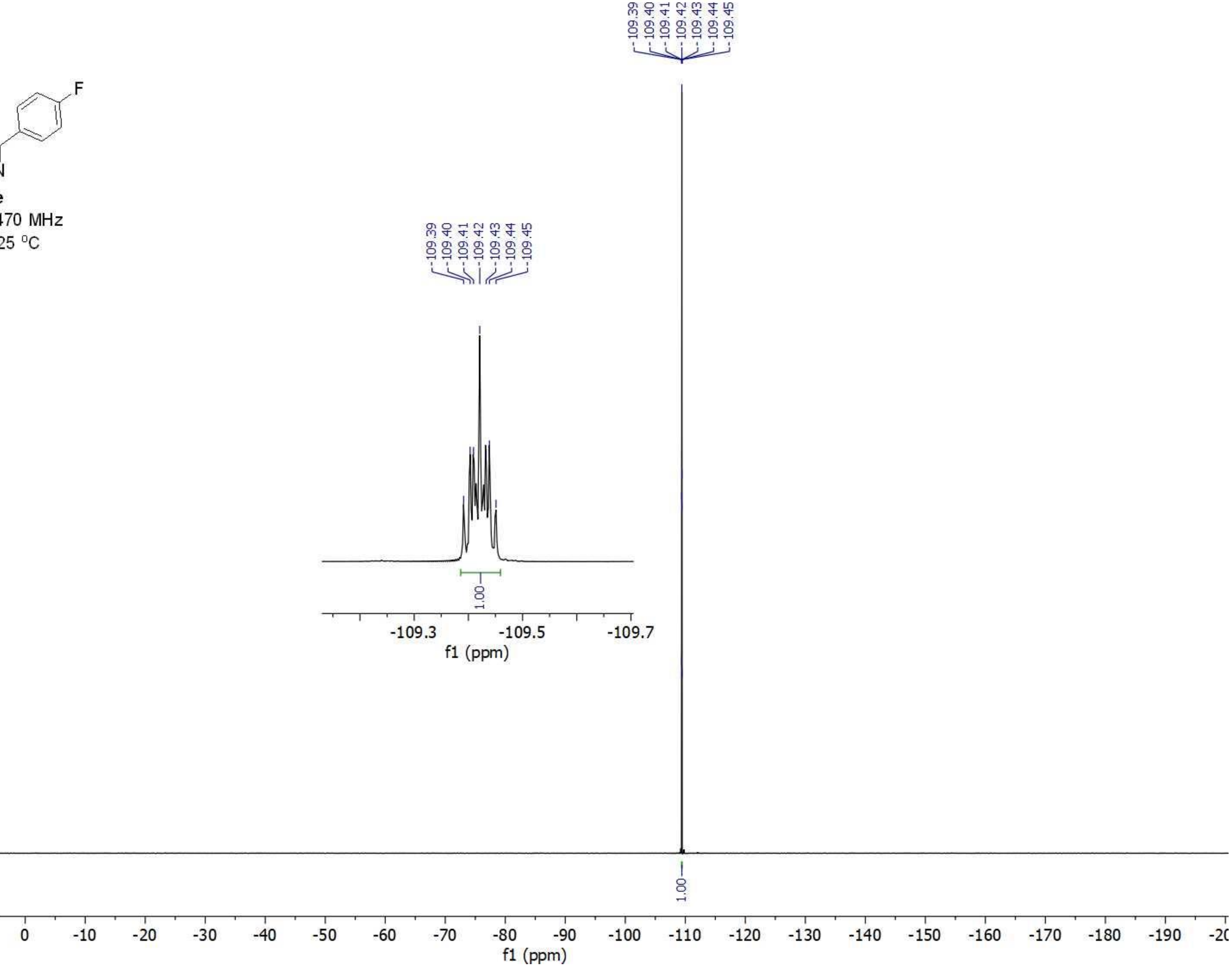

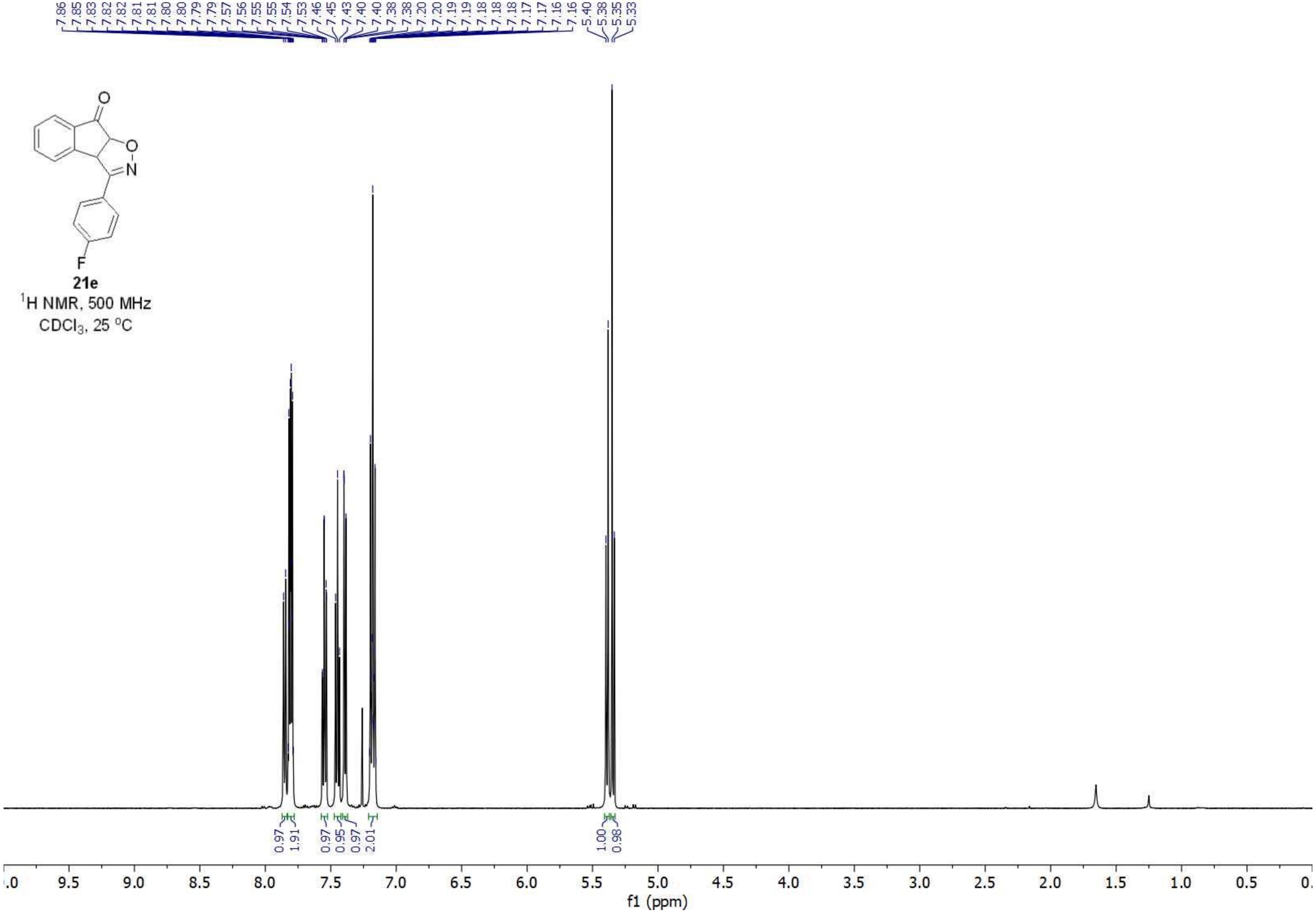
7.86 7.85 7.83 7.82 7.82 7.81 7.81 7.80 7.80 7.79 7.79 7.57 7.56 7.55 7.55 7.54 7.53 7.46 7.45 7.43 7.40 7.40 7.38 7.38 7.20 7.20 7.19 7.19 7.18 7.18 7.17 7.17 7.16 7.16 5.40 5.38 5.35 5.33
O
O
N
F
21e
1H NMR, 500 MHz
CDCl3, 25 °C
0.97 1.91 0.97 0.95 0.97 2.01 1.00 0.98
f1 (ppm)

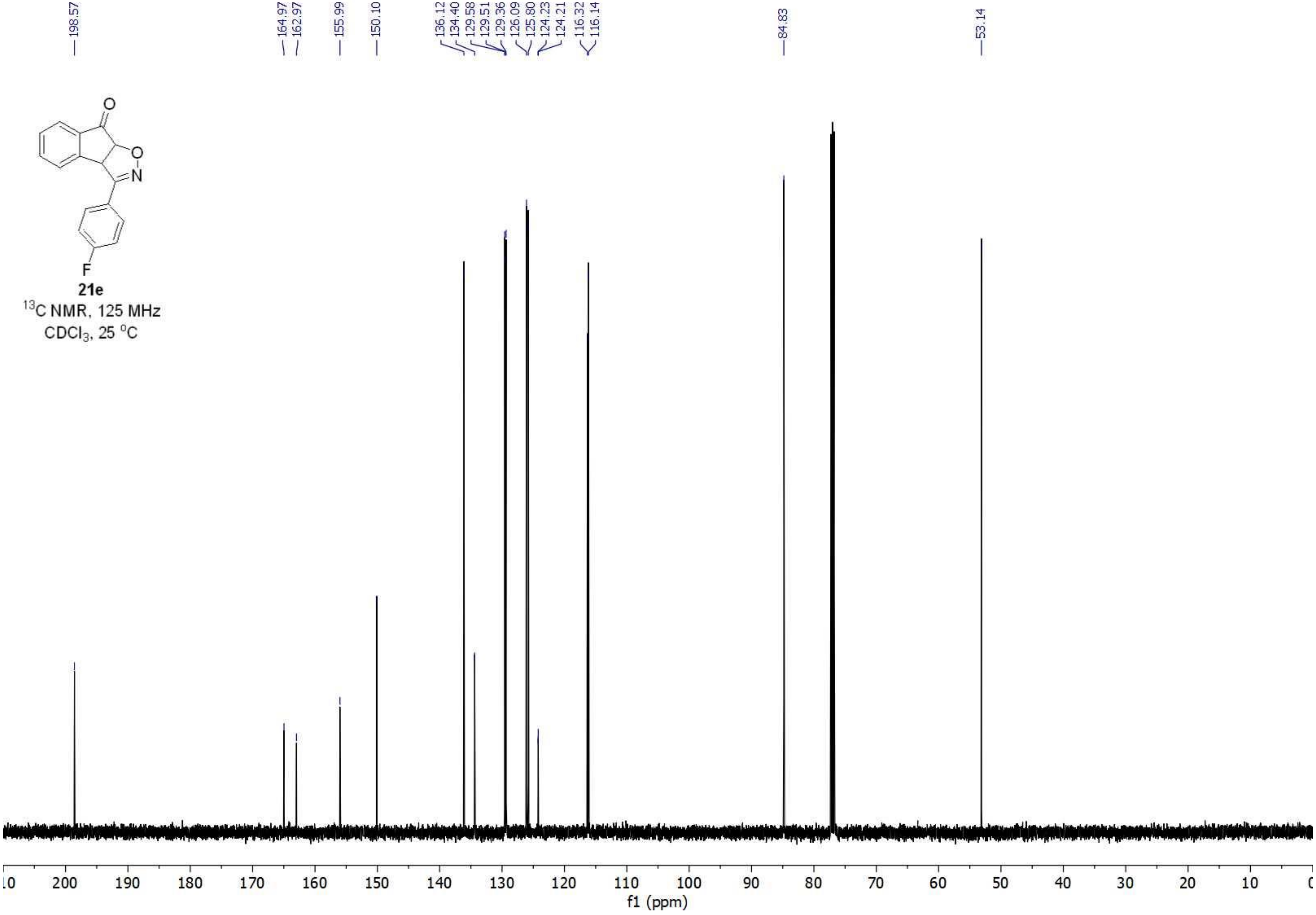

21e

^{13}C NMR, 125 MHz

$CDCl_3$, 25 °C

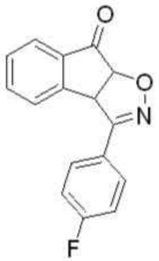

21e
^{19}F NMR, 470 MHz
$CDCl_3$, 25 ^{o}C

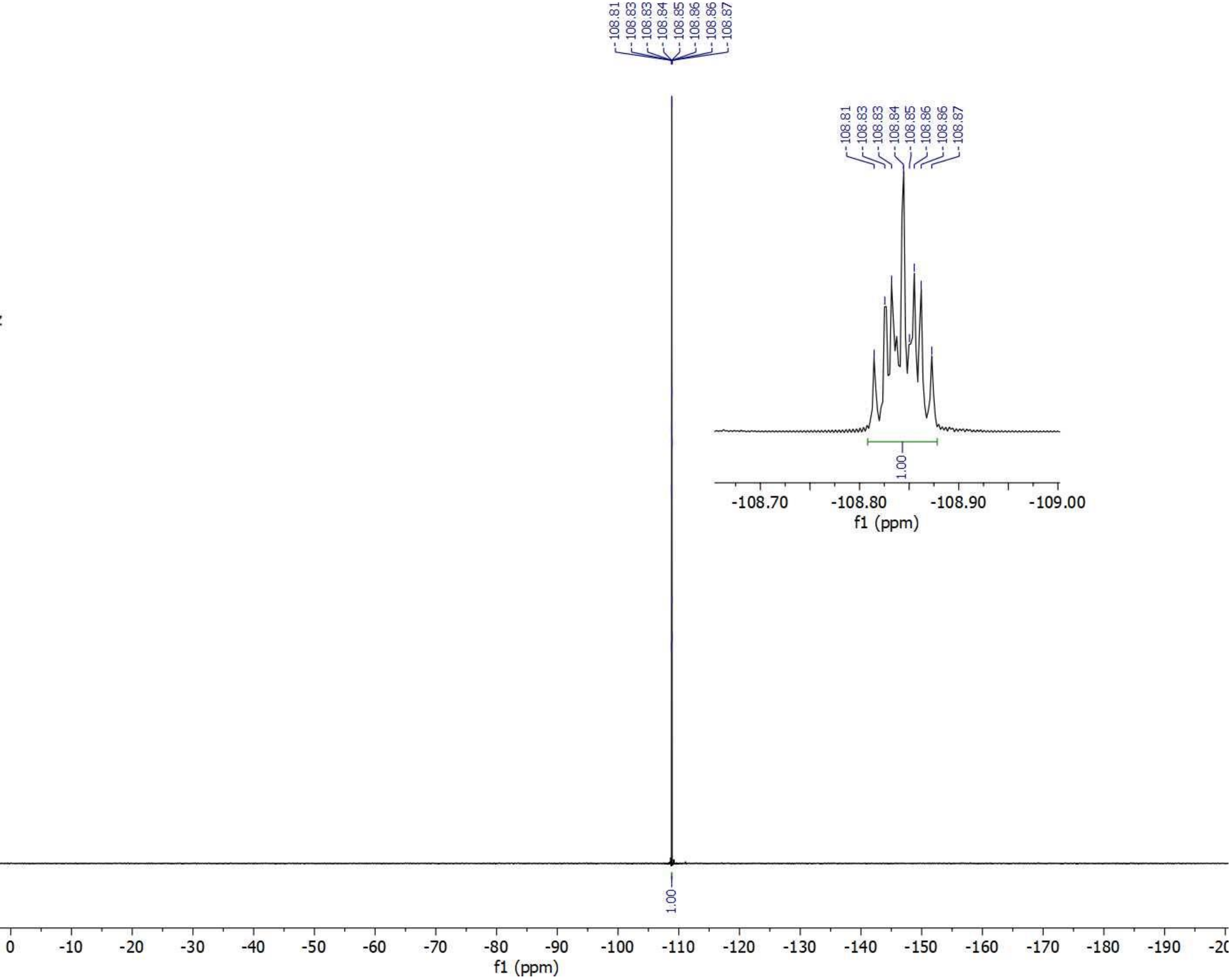

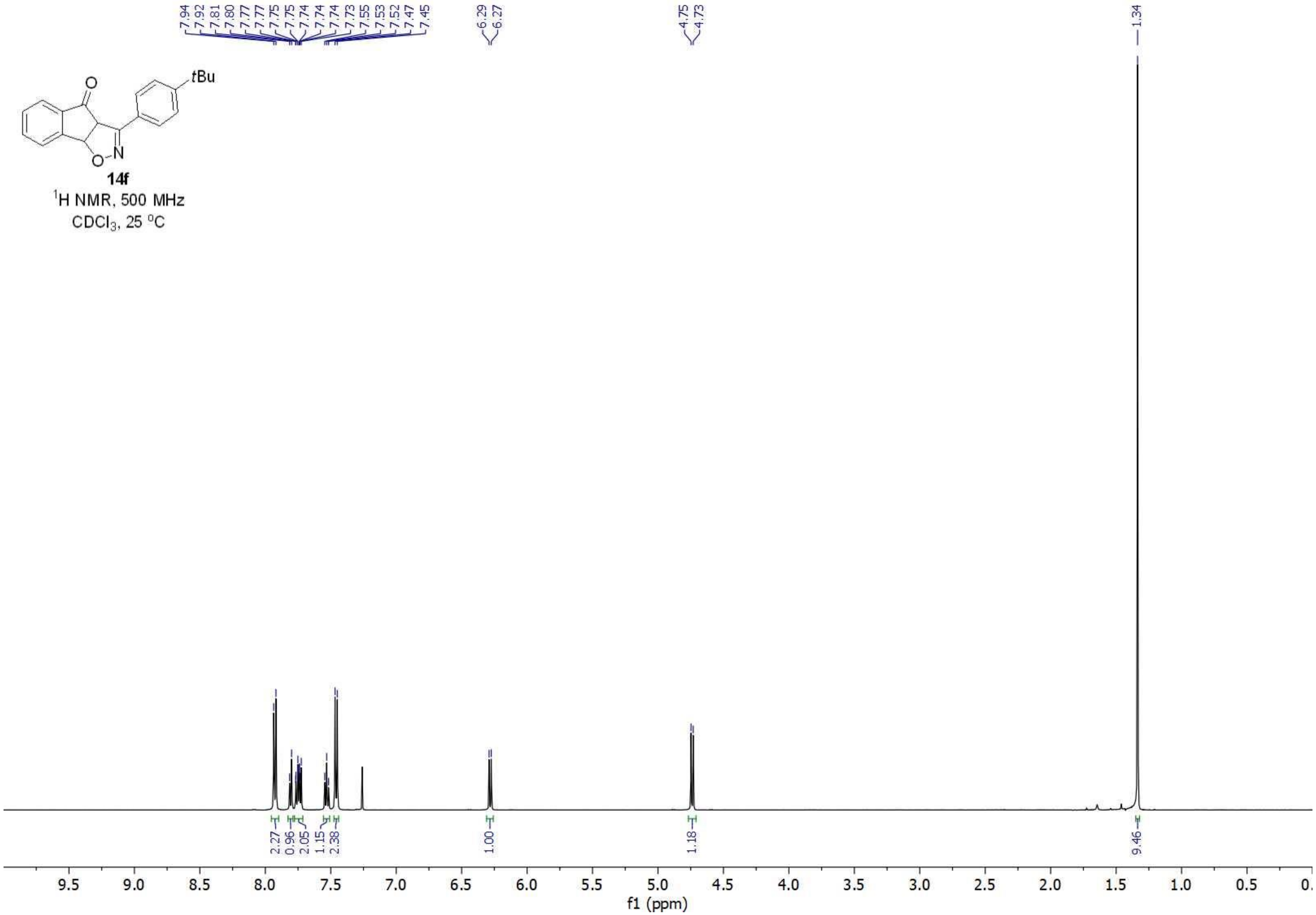

7.94
7.92
7.81
7.80
7.77
7.77
7.75
7.75
7.74
7.74
7.74
7.73
7.55
7.53
7.52
7.47
7.45
6.29
6.27
4.75
4.73
1.34
tBu
O
N
14f
^{1}H NMR, 500 MHz
CDCl3, 25 °C
2.27
0.96
2.05
1.15
2.38
1.00
1.18
9.46
9.5
9.0
8.5
8.0
7.5
7.0
6.5
6.0
5.5
5.0
4.5
4.0
3.5
3.0
2.5
2.0
1.5
1.0
0.5
f1 (ppm)

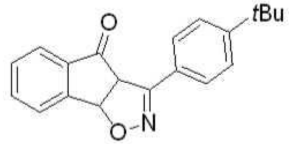

14f
^{13}C NMR, 125 MHz
$CDCl_3$, 25 °C

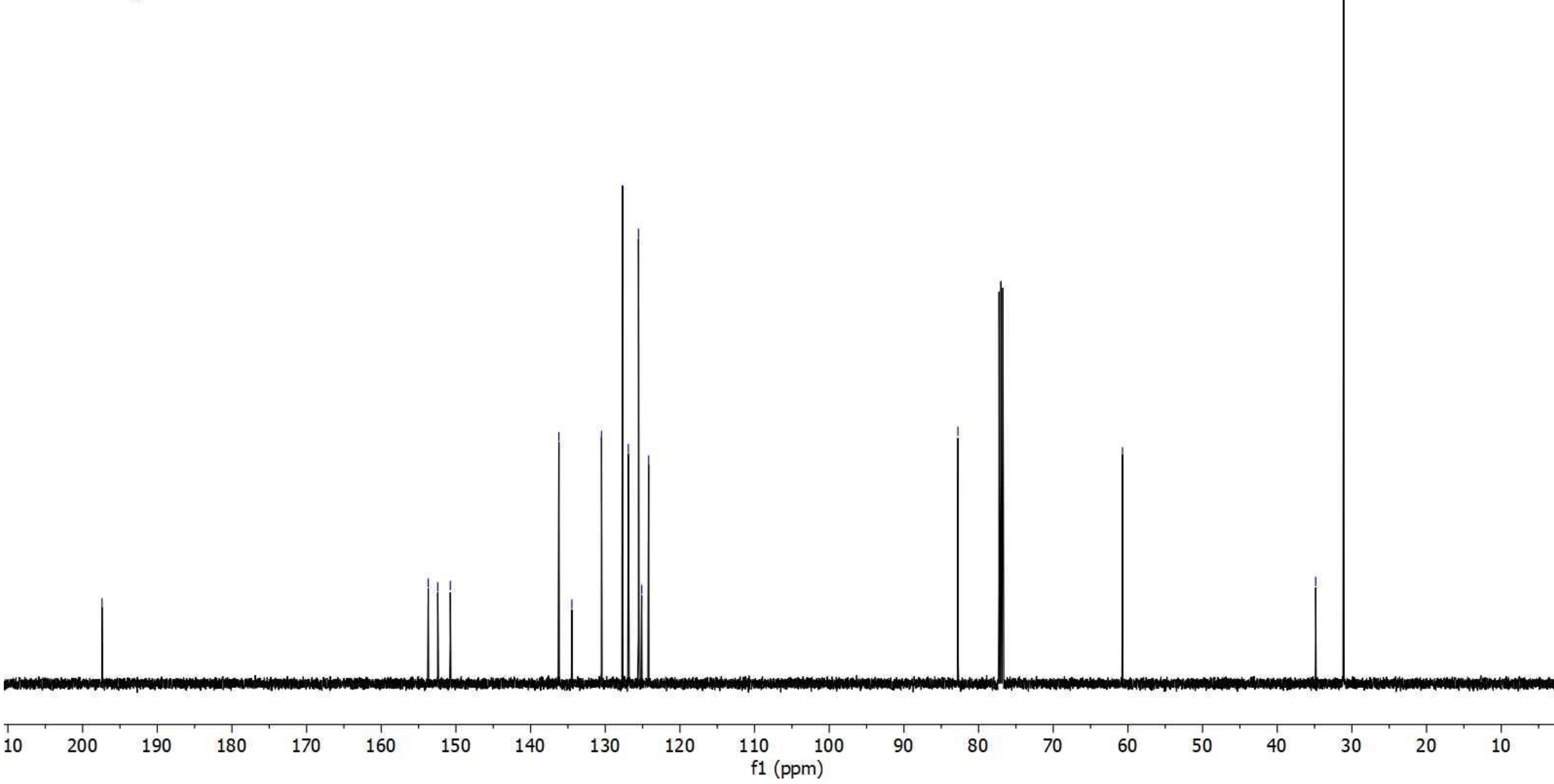

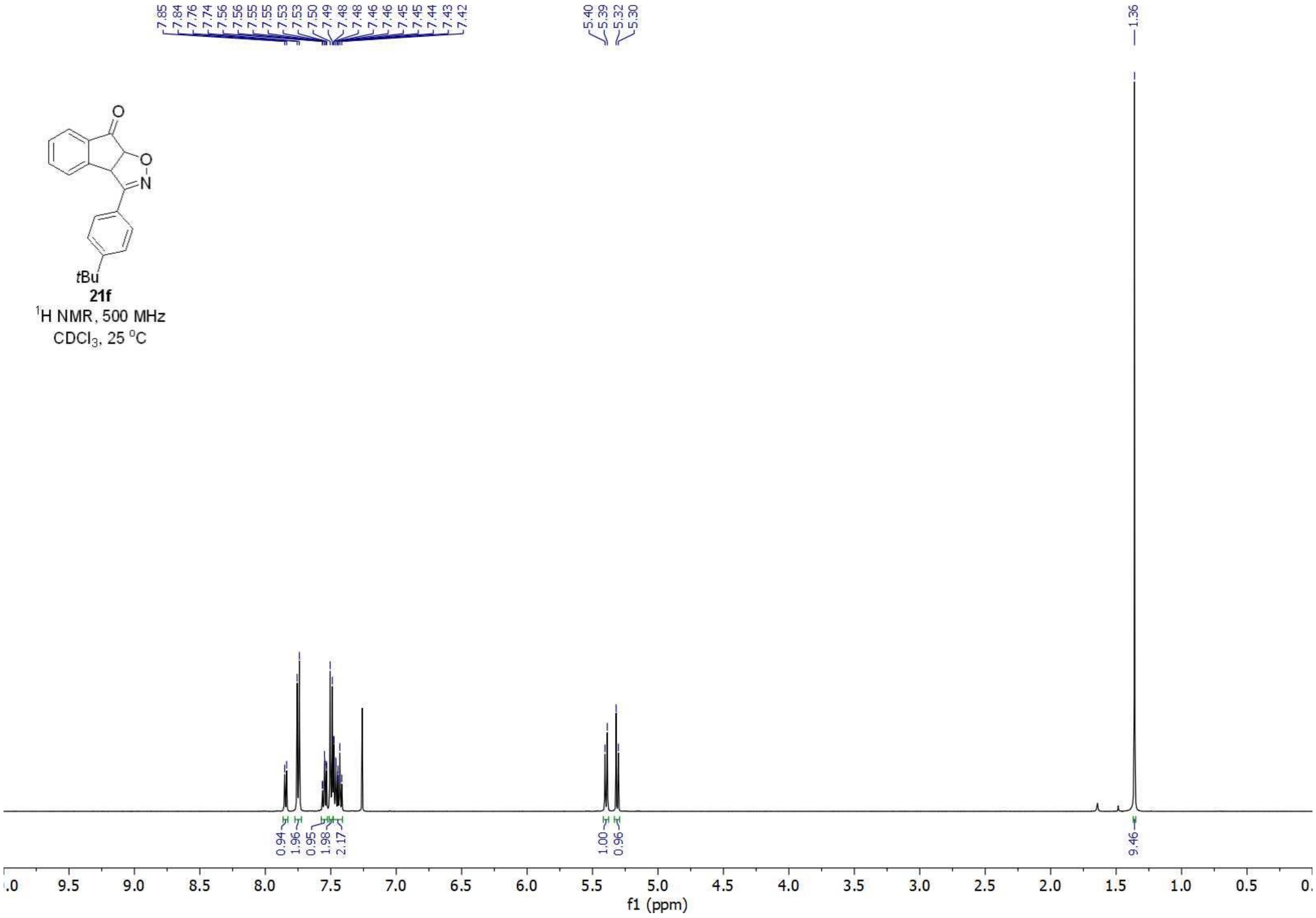
7.85
7.84
7.76
7.74
7.56
7.56
7.55
7.55
7.53
7.53
7.50
7.49
7.48
7.48
7.46
7.46
7.45
7.45
7.44
7.43
7.42
5.40
5.39
5.32
5.30
1.36
O
O
N
tBu
21f
^{1}H NMR, 500 MHz
$CDCl_3$, 25 °C
0.94
1.96
0.95
1.98
2.17
1.00
0.96
9.46
9.5
9.0
8.5
8.0
7.5
7.0
6.5
6.0
5.5
5.0
4.5
4.0
3.5
3.0
2.5
2.0
1.5
1.0
0.5
f1 (ppm)

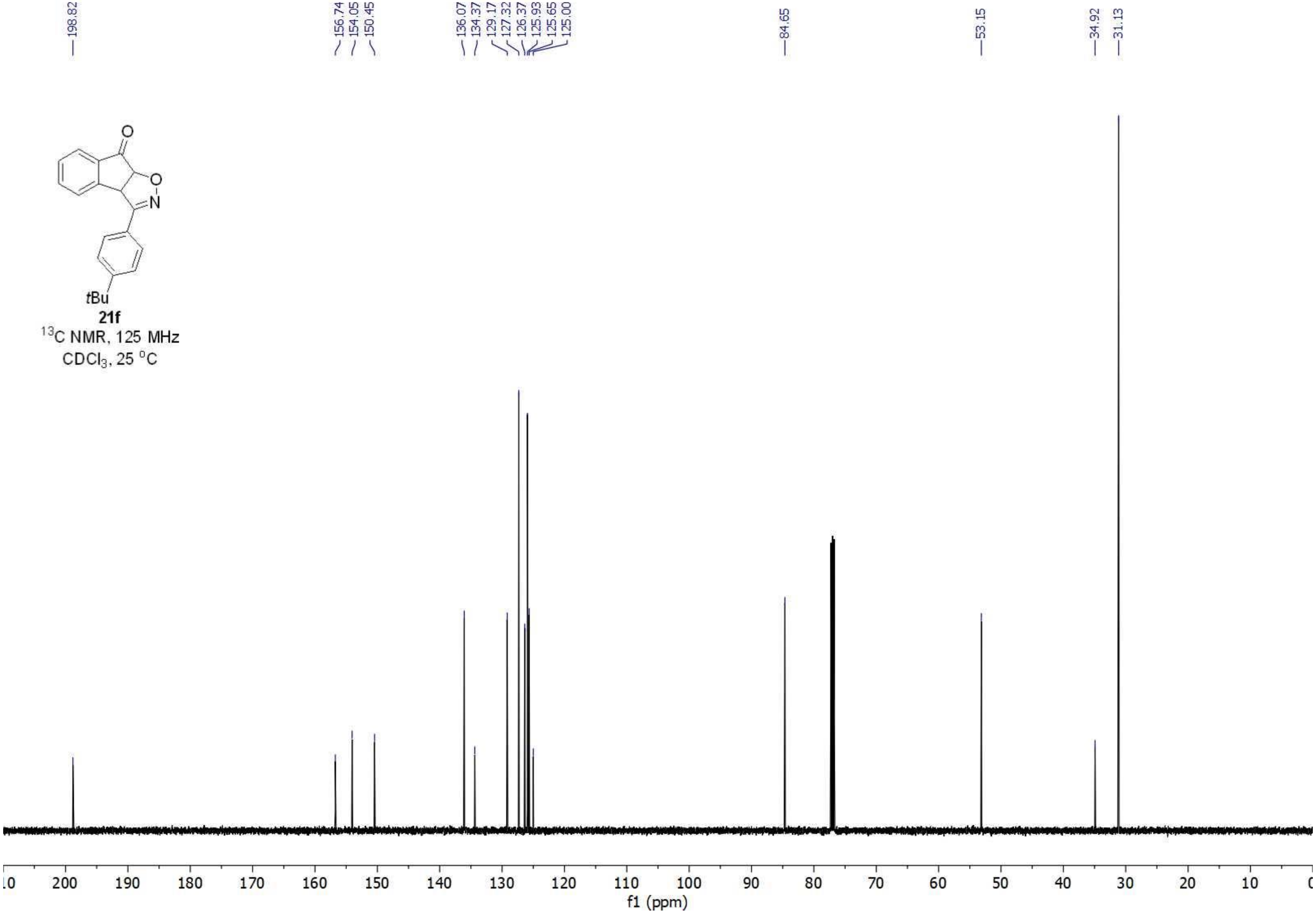
198.82
156.74
154.05
150.45
136.07
134.37
129.17
127.32
126.37
125.93
125.65
125.00
84.65
53.15
34.92
31.13
tBu
21f
^{13}C NMR, 125 MHz
$CDCl_3$, 25 °C
200
190
180
170
160
150
140
130
120
110
100
90
80
70
60
50
40
30
20
10
f1 (ppm)

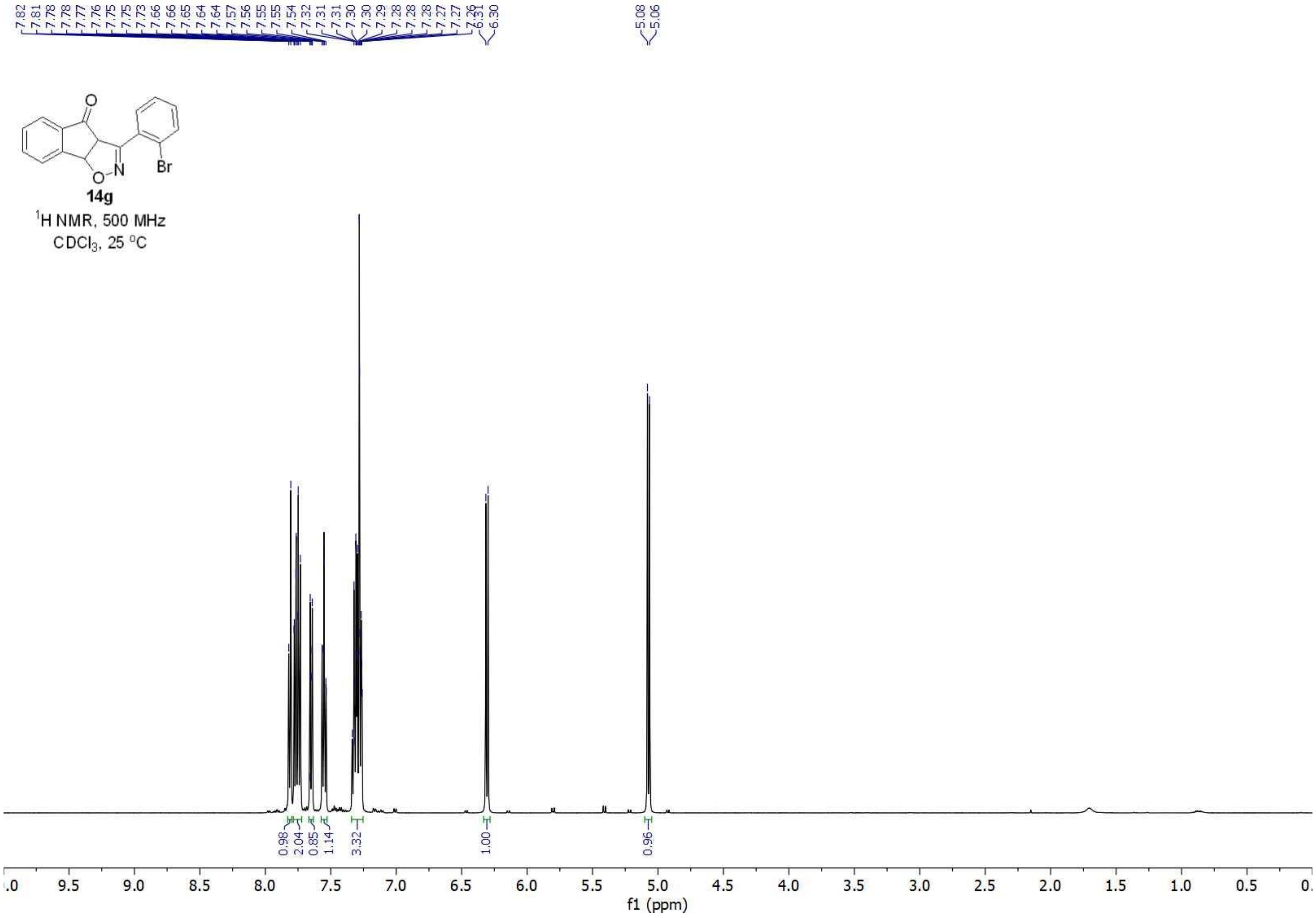
7.82 7.81 7.78 7.78 7.77 7.76 7.75 7.75 7.73 7.66 7.66 7.65 7.64 7.64 7.57 7.56 7.55 7.55 7.54 7.32 7.31 7.31 7.30 7.30 7.29 7.28 7.28 7.28 7.27 7.27 7.26 6.31 6.30
5.08 5.06
O
N
Br
14g
^{1}H NMR, 500 MHz
$CDCl_3$, 25 °C
0.98 2.04 0.85 1.14 3.32 1.00 0.96
9.5 9.0 8.5 8.0 7.5 7.0 6.5 6.0 5.5 5.0 4.5 4.0 3.5 3.0 2.5 2.0 1.5 1.0 0.5
f1 (ppm)

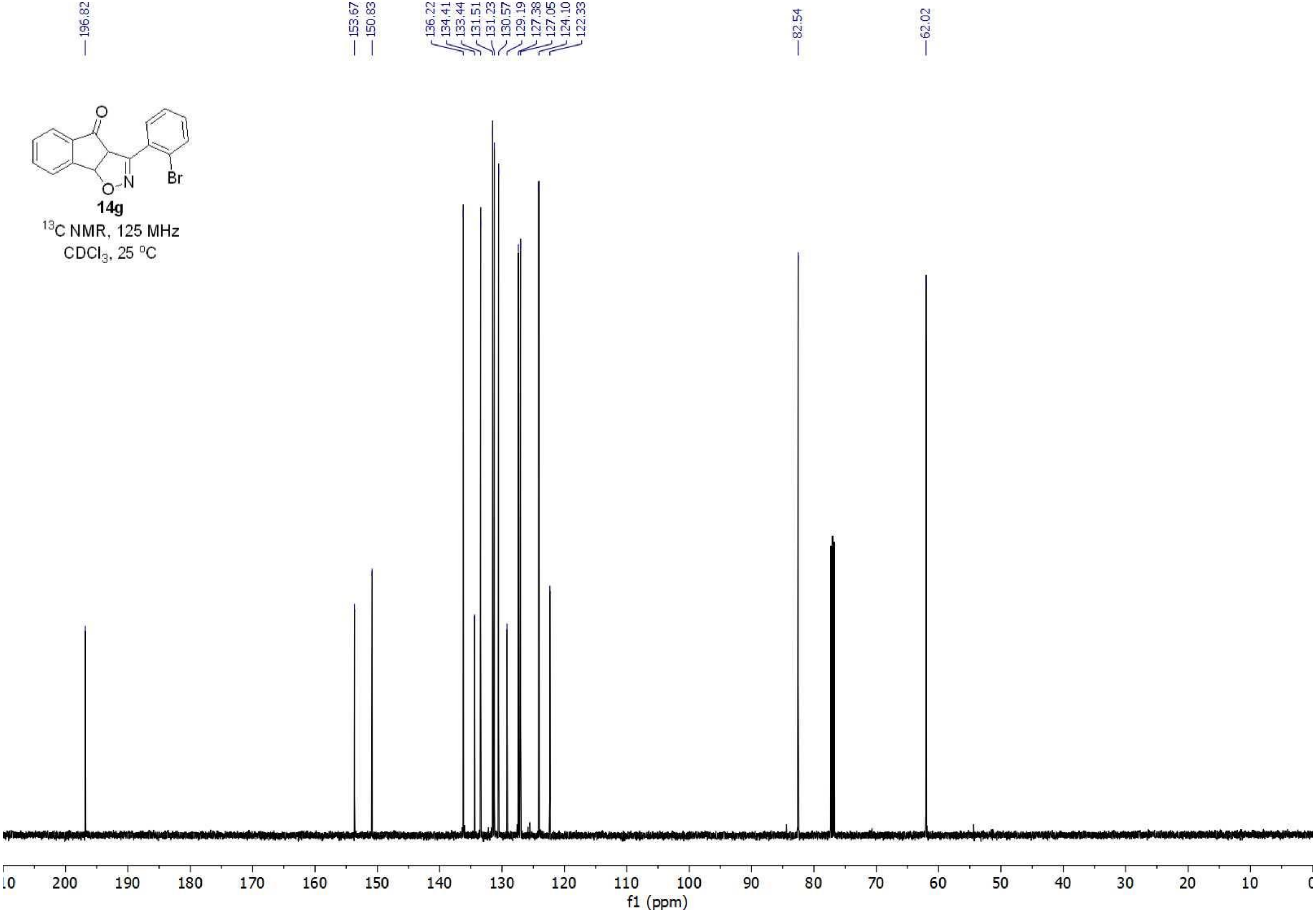
196.82
153.67
150.83
136.22
134.41
133.44
131.51
131.23
130.57
129.19
127.38
127.05
124.10
122.33
82.54
62.02
O
O
N
Br
14g
13C NMR, 125 MHz
CDCl3, 25 °C
f1 (ppm)

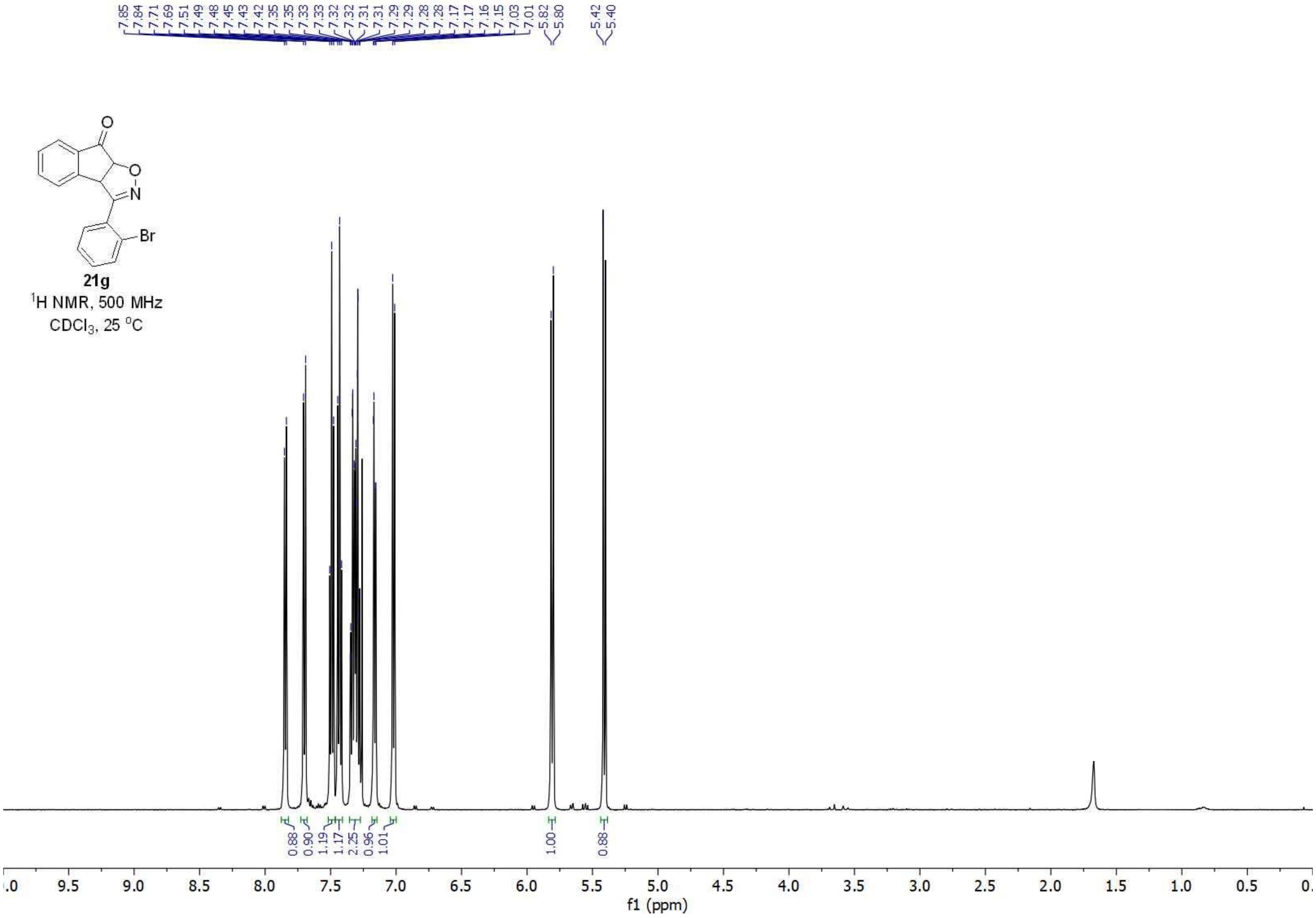
7.85
7.84
7.71
7.69
7.51
7.49
7.48
7.45
7.43
7.42
7.35
7.35
7.33
7.33
7.32
7.32
7.31
7.31
7.29
7.29
7.28
7.28
7.17
7.17
7.16
7.15
7.03
7.01
5.82
5.80
5.42
5.40
O
O
N
Br
21g
1H NMR, 500 MHz
CDCl3, 25 °C
0.88
0.90
1.19
1.17
2.25
0.96
1.01
1.00
0.88
9.5
9.0
8.5
8.0
7.5
7.0
6.5
6.0
5.5
5.0
4.5
4.0
3.5
3.0
2.5
2.0
1.5
1.0
0.5
f1 (ppm)

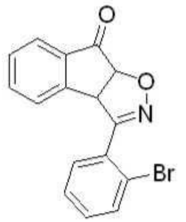

21g
^{13}C NMR, 125 MHz
$CDCl_3$, 25 °C

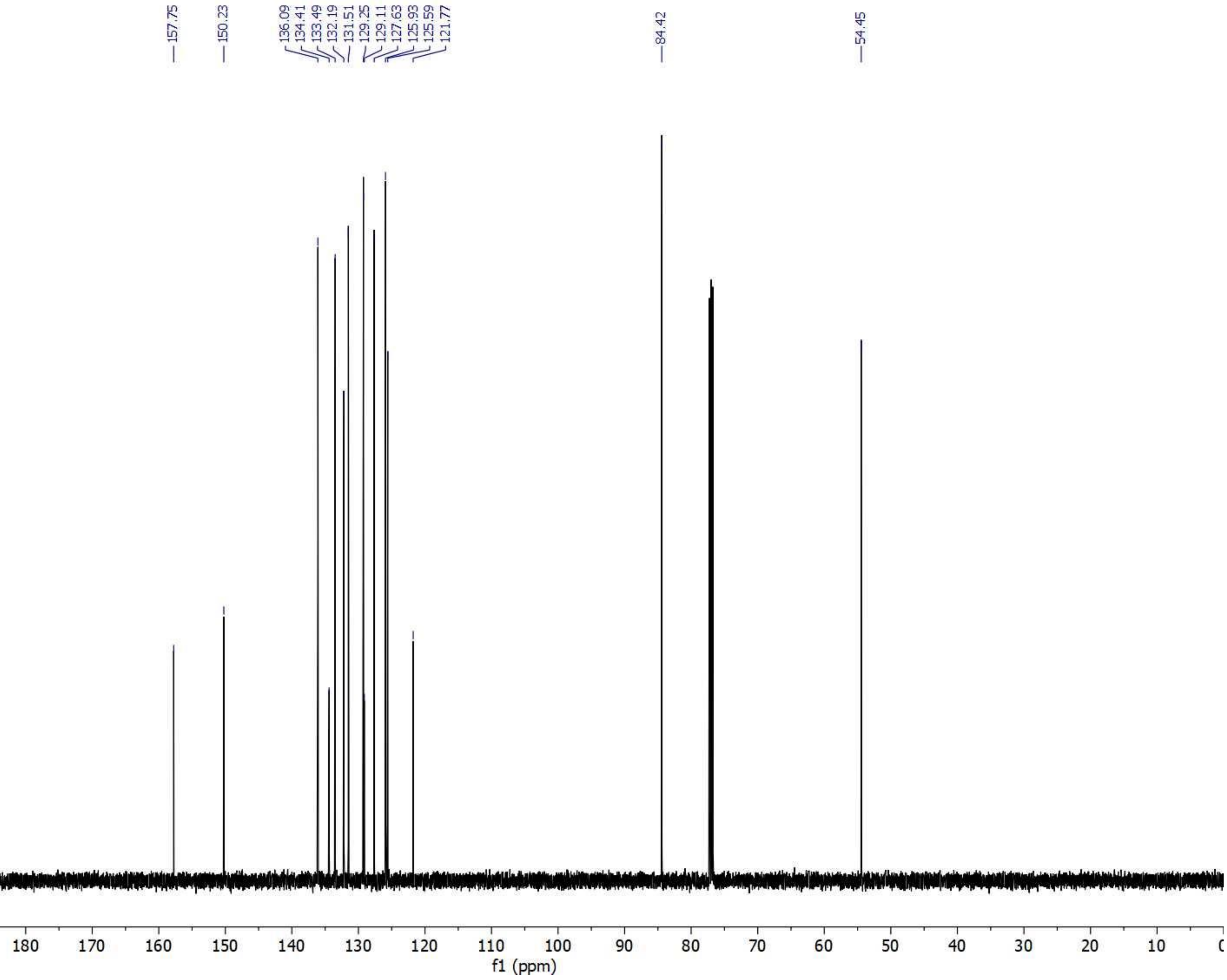

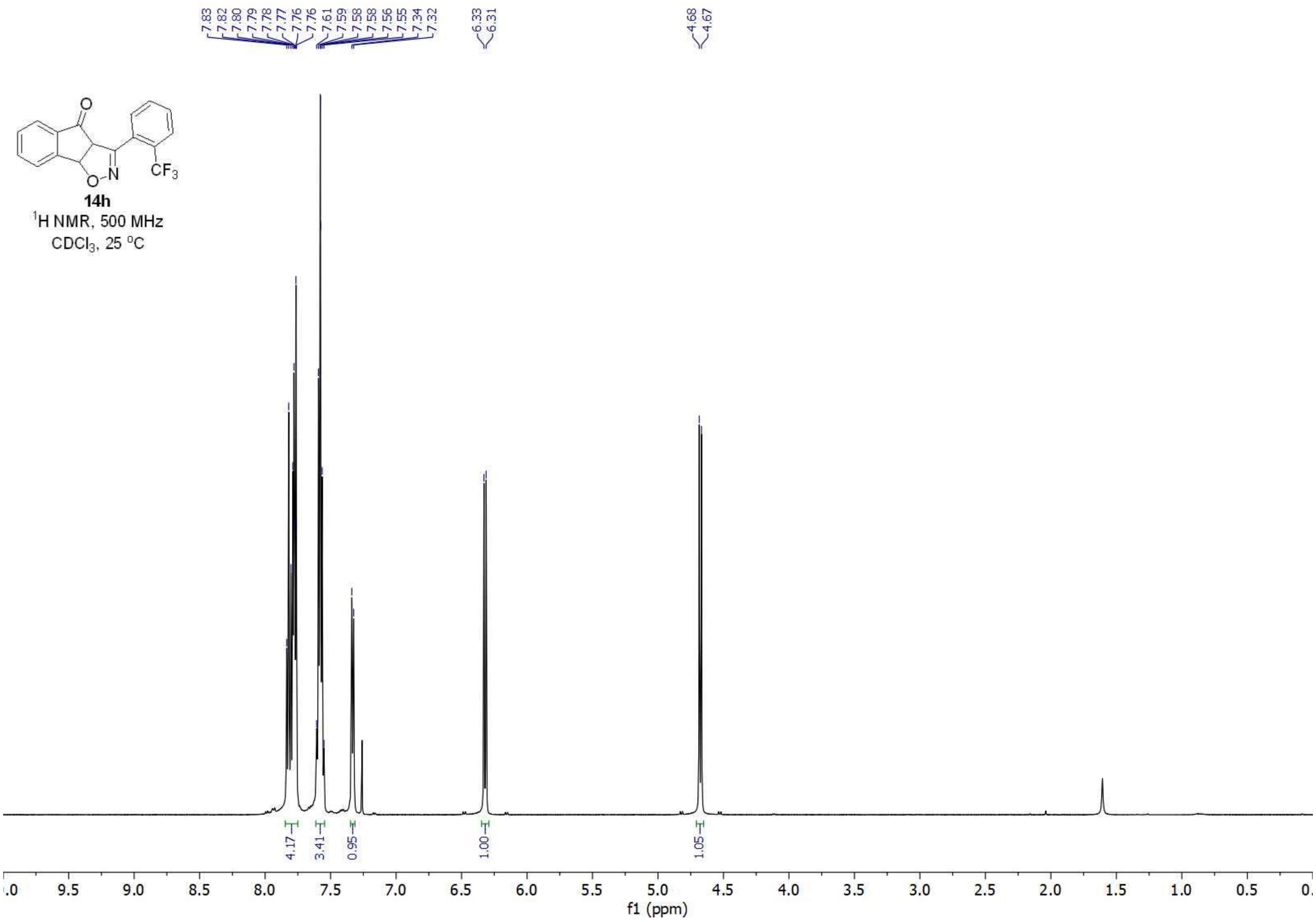
7.83 7.82 7.80 7.79 7.78 7.77 7.76 7.76 7.61 7.59 7.58 7.58 7.56 7.55 7.34 7.32
6.33 6.31
4.68 4.67
14h
^{1}H NMR, 500 MHz
$CDCl_3$, 25 °C
4.17
3.41
0.95
1.00
1.05
f1 (ppm)

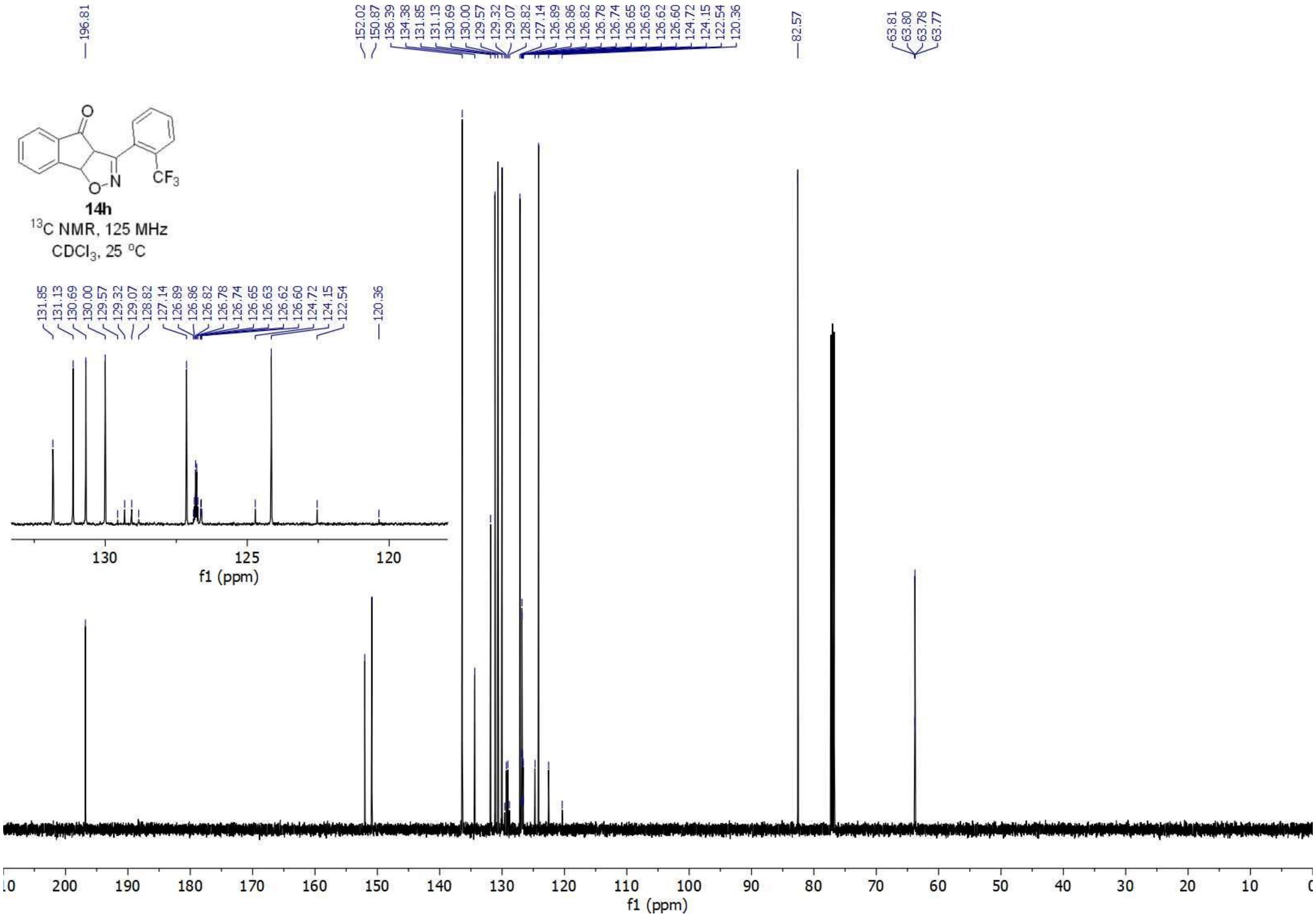
196.81
152.02
150.87
136.39
134.38
131.85
131.13
130.69
130.00
129.57
129.32
129.07
128.82
127.14
126.89
126.86
126.82
126.78
126.74
126.65
126.63
126.62
126.60
124.72
124.15
122.54
120.36
82.57
63.81
63.80
63.78
63.77
O
O
N
CF3
14h
13C NMR, 125 MHz
CDCl3, 25 °C
130
125
120
f1 (ppm)
200
190
180
170
160
150
140
130
120
110
100
90
80
70
60
50
40
30
20
10
f1 (ppm)

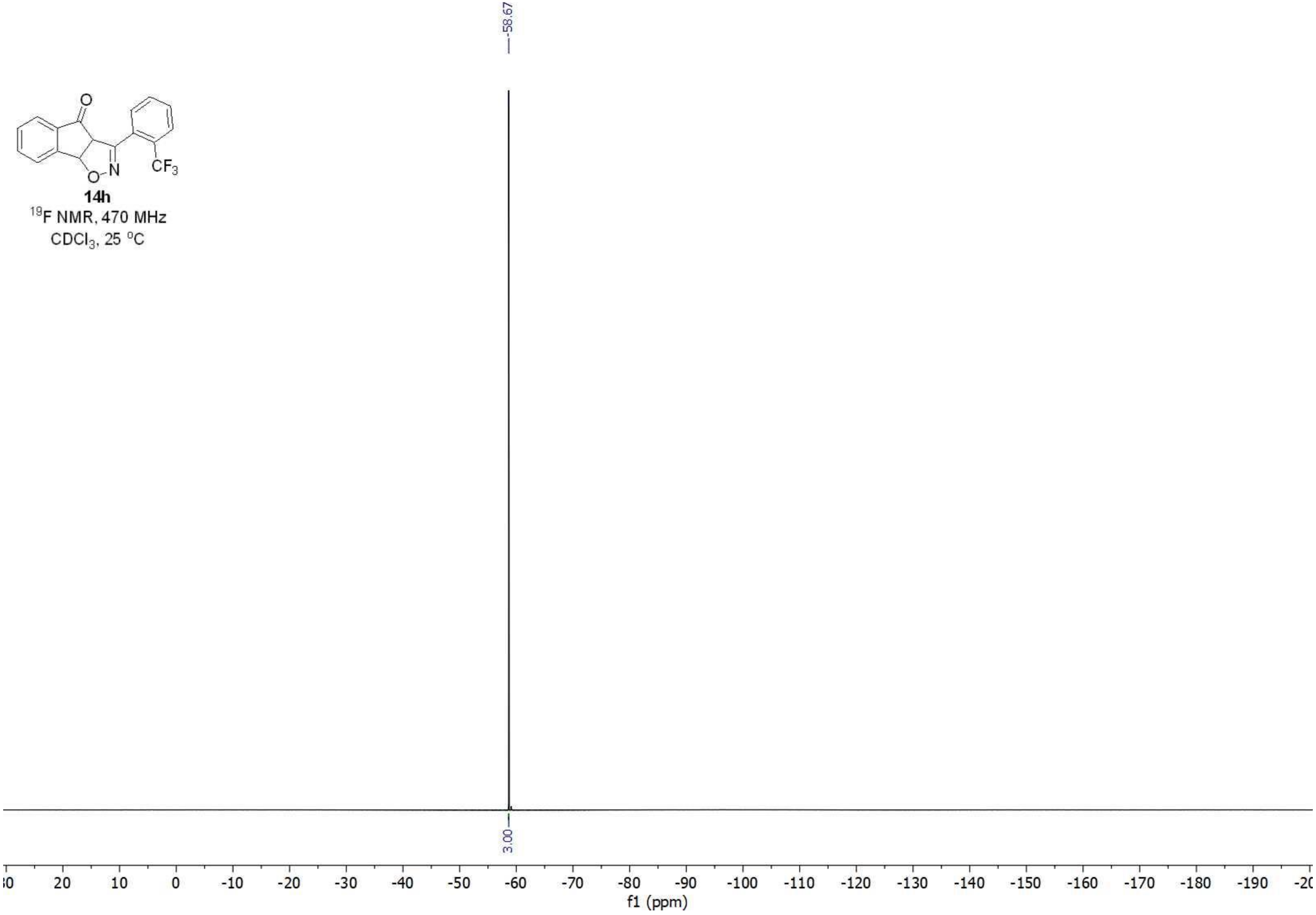

14h
19F NMR, 470 MHz
CDCl3, 25 °C
-58.67
3.00
20
10
0
-10
-20
-30
-40
-50
-60
-70
-80
-90
-100
-110
-120
-130
-140
-150
-160
-170
-180
-190
f1 (ppm)

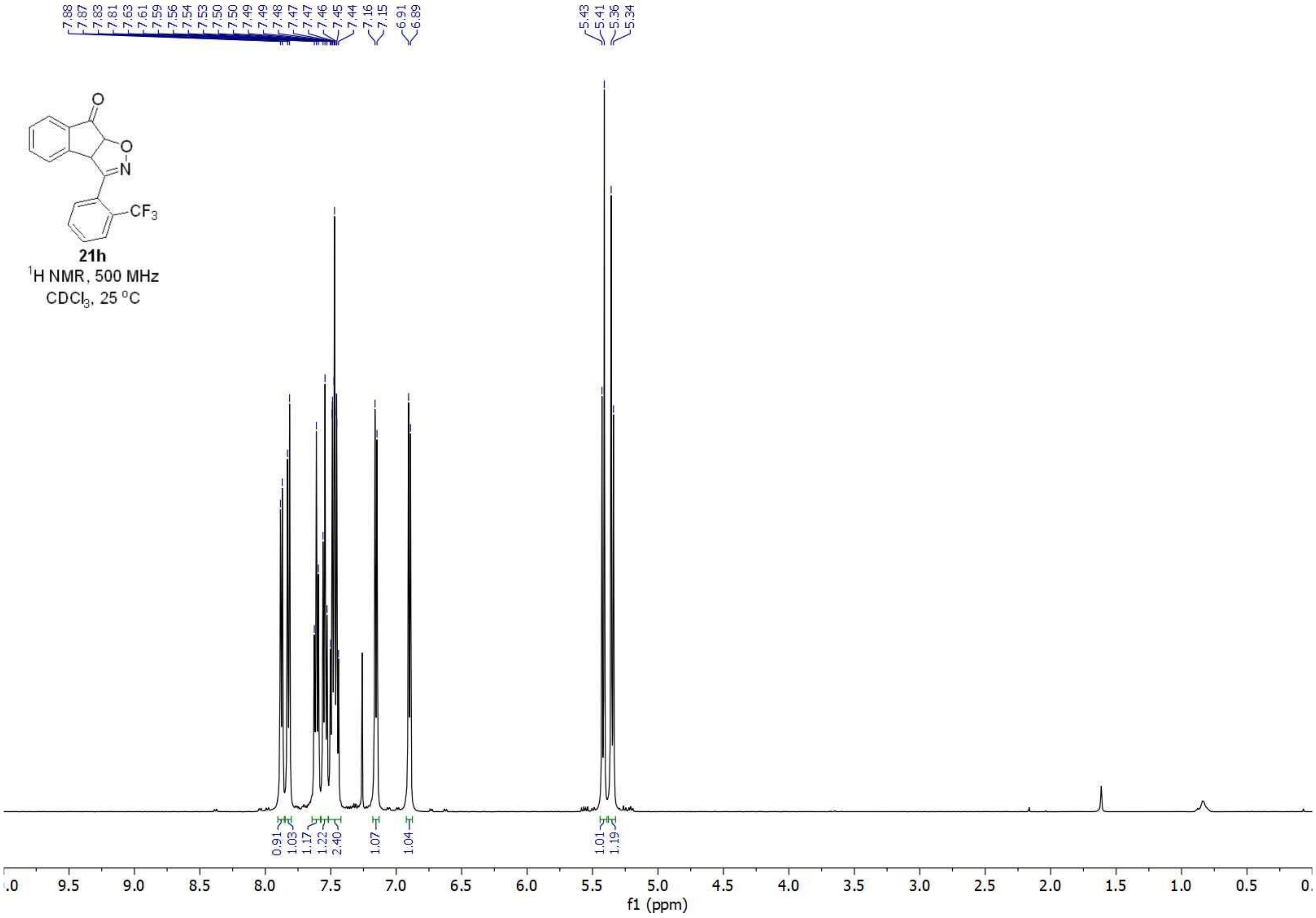

21h
^{1}H NMR, 500 MHz
$CDCl_3$, 25 °C

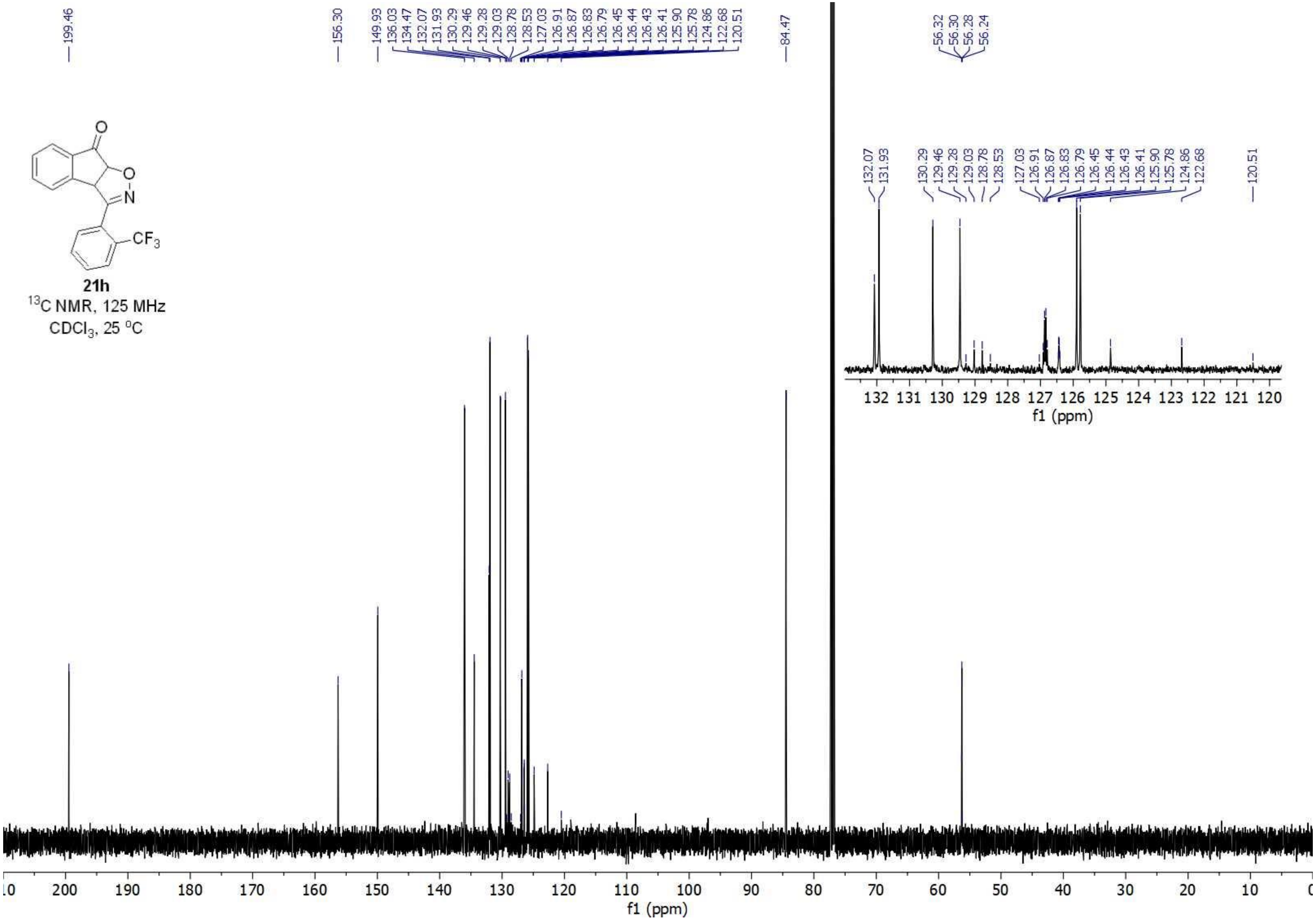
199.46
156.30
149.93
136.03
134.47
132.07
131.93
130.29
129.46
129.28
129.03
128.78
128.53
127.03
126.91
126.87
126.83
126.79
126.45
126.44
126.43
126.41
125.90
125.78
124.86
122.68
120.51
84.47
56.32
56.30
56.28
56.24
O
N
CF3
21h
13C NMR, 125 MHz
CDCl3, 25 °C
132 131 130 129 128 127 126 125 124 123 122 121 120
f1 (ppm)
200 190 180 170 160 150 140 130 120 110 100 90 80 70 60 50 40 30 20 10
f1 (ppm)

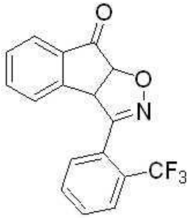

21h
^{19}F NMR, 470 MHz
$CDCl_3$, 25 ^{o}C

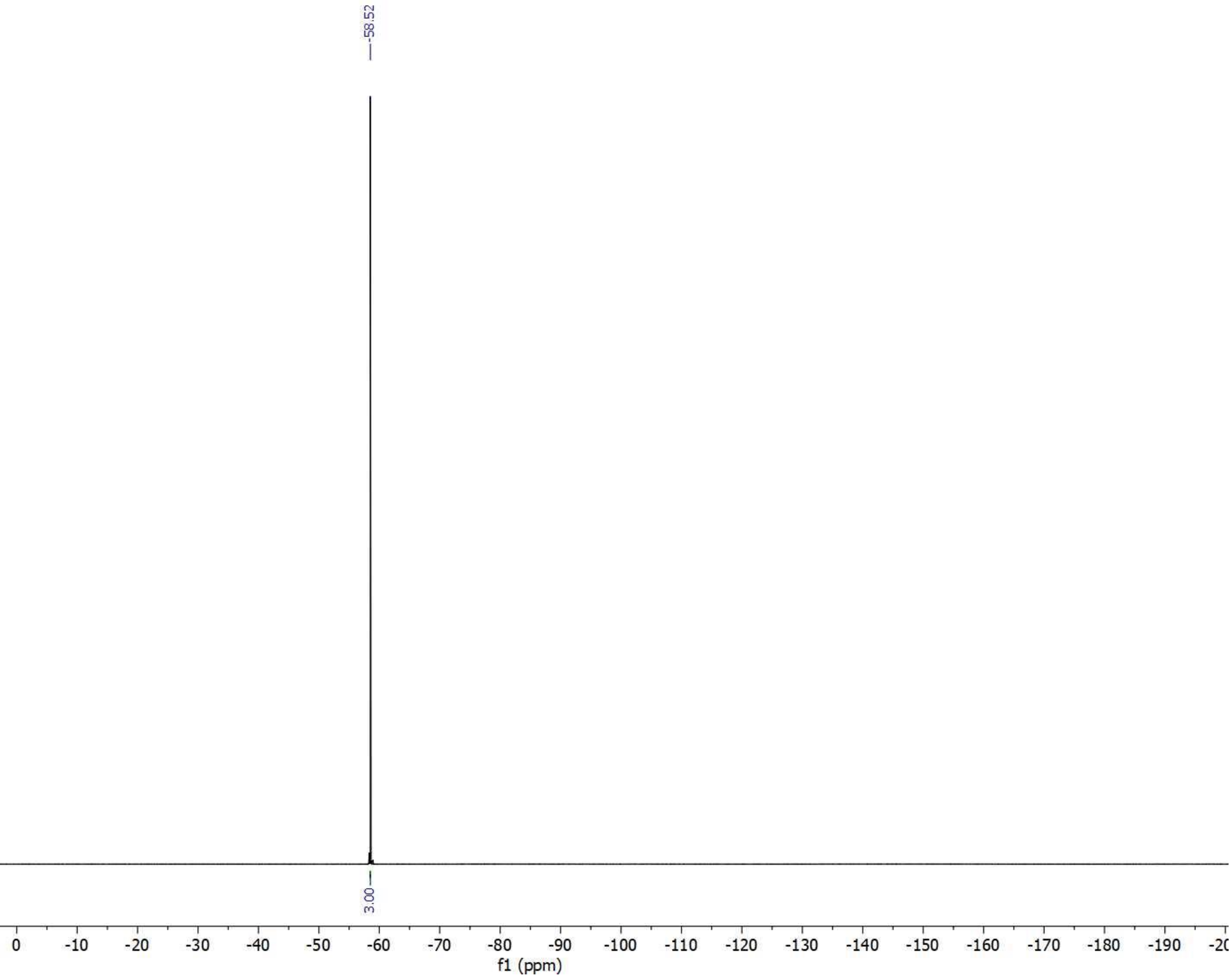

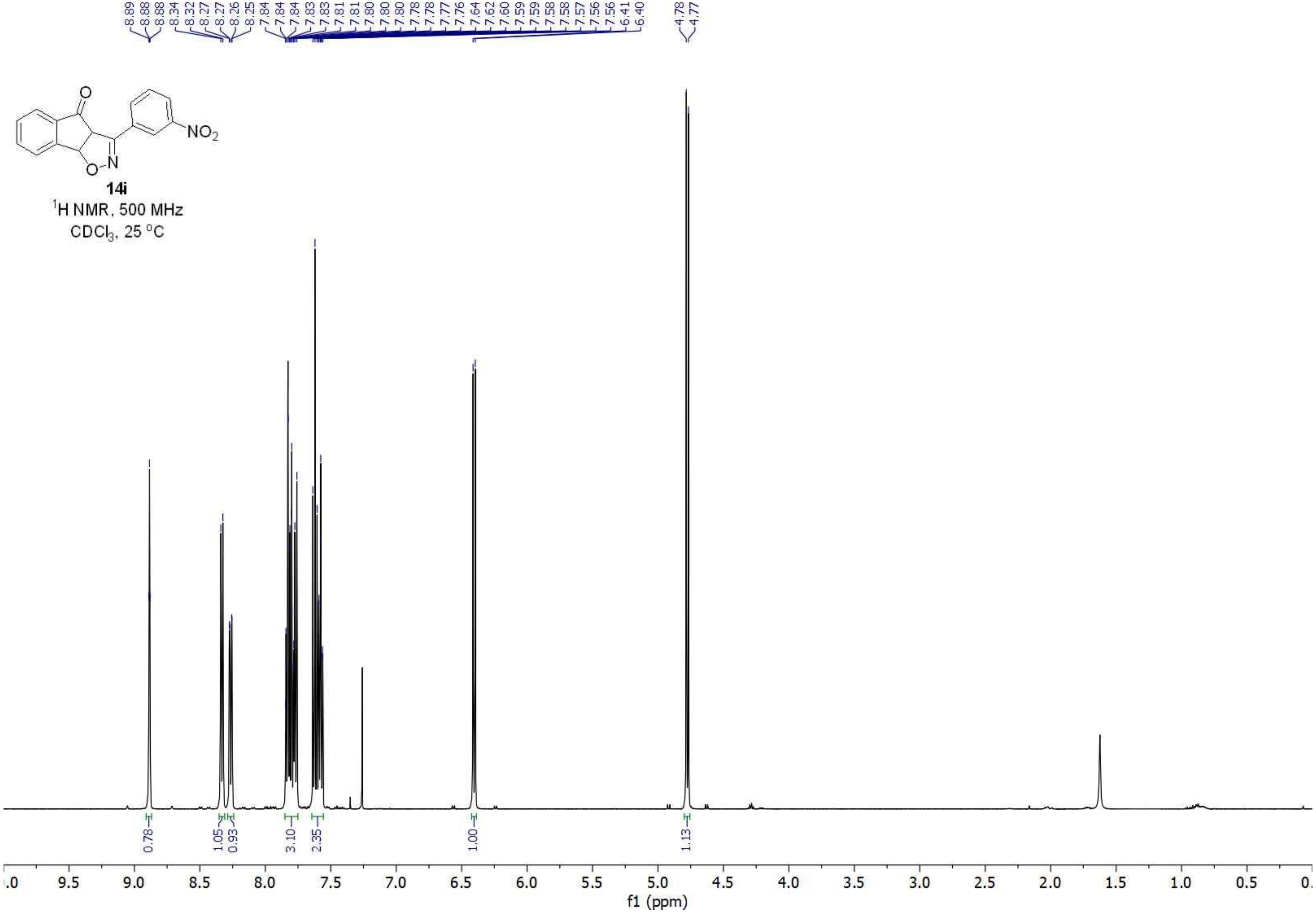
8.89
8.88
8.88
8.34
8.32
8.27
8.27
8.26
8.25
7.84
7.84
7.84
7.83
7.83
7.81
7.81
7.80
7.80
7.78
7.78
7.77
7.76
7.64
7.62
7.60
7.59
7.58
7.58
7.57
7.56
7.56
6.41
6.40
4.78
4.77
NO2
14i
1H NMR, 500 MHz
CDCl3, 25 °C
0.78
1.05
0.93
3.10
2.35
1.00
1.13
9.5
9.0
8.5
8.0
7.5
7.0
6.5
6.0
5.5
5.0
4.5
4.0
3.5
3.0
2.5
2.0
1.5
1.0
0.5
f1 (ppm)

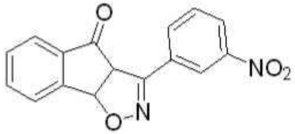

14i
^{13}C NMR, 125 MHz
$CDCl_3$, 25 °C

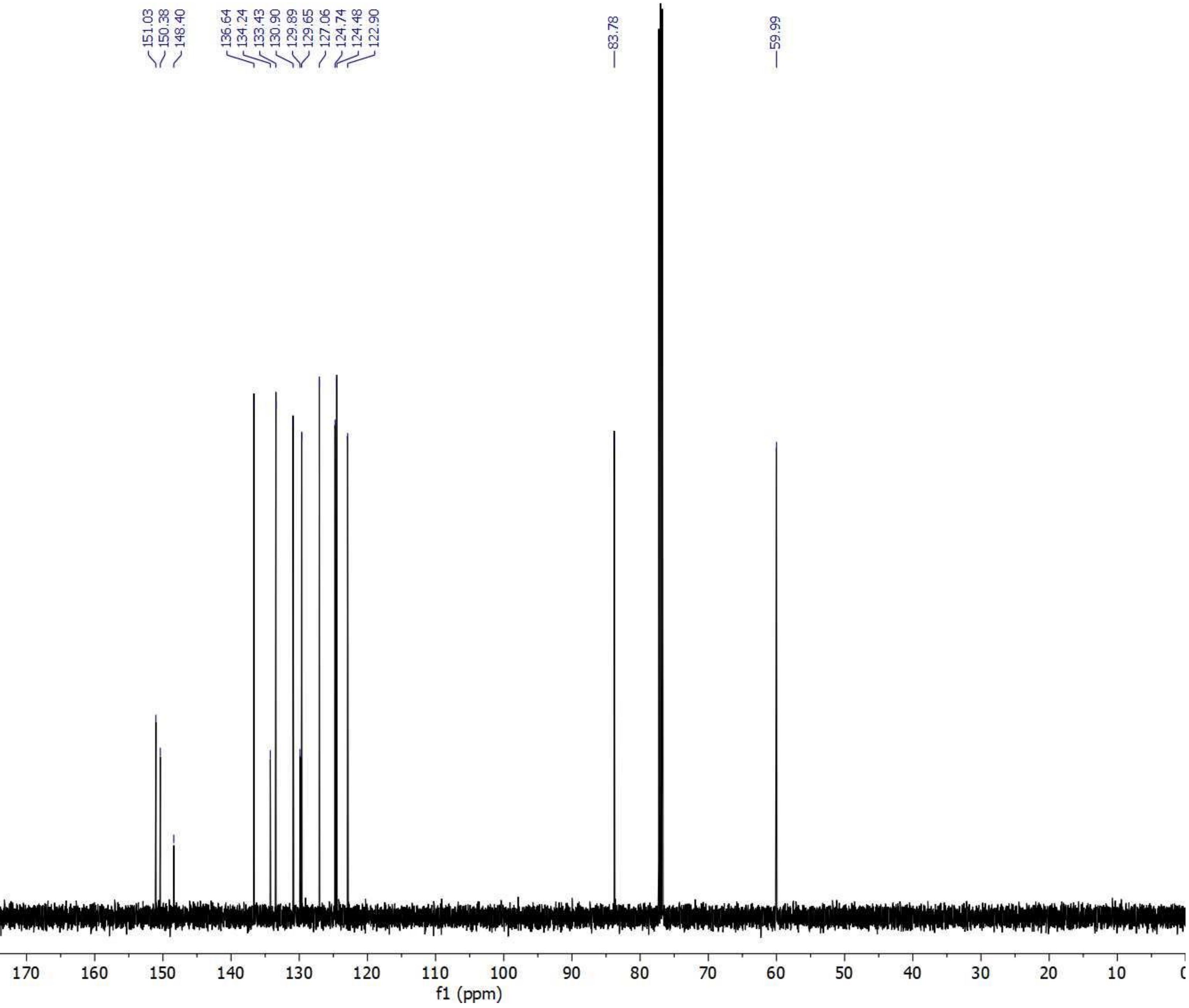

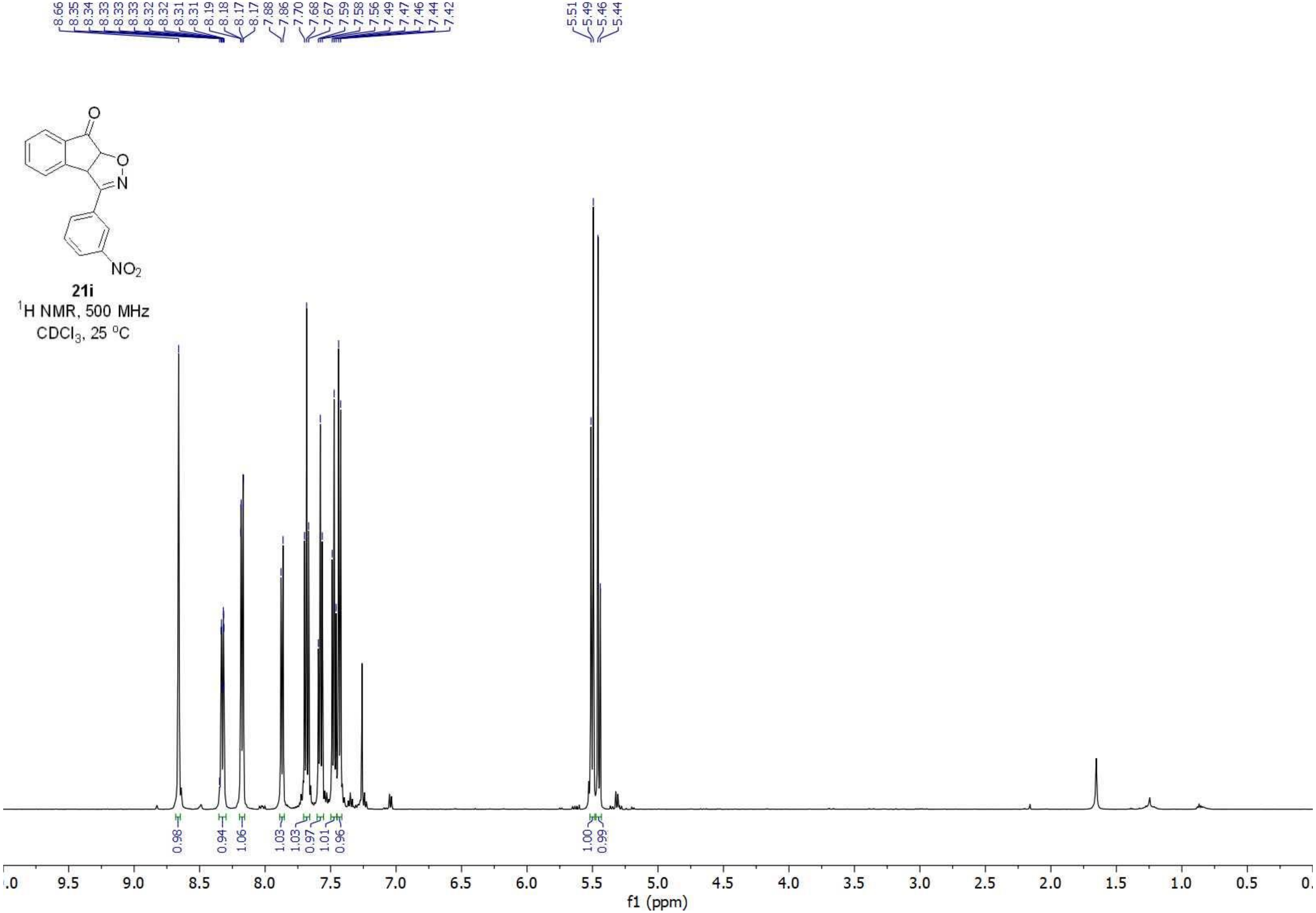
8.66
8.35
8.34
8.33
8.33
8.33
8.32
8.32
8.31
8.31
8.19
8.18
8.17
8.17
7.88
7.86
7.70
7.68
7.67
7.59
7.58
7.56
7.49
7.47
7.46
7.44
7.42
5.51
5.49
5.46
5.44
21i
1H NMR, 500 MHz
CDCl3, 25 °C
0.98
0.94
1.06
1.03
1.03
0.97
1.01
0.96
1.00
0.99
9.5
9.0
8.5
8.0
7.5
7.0
6.5
6.0
5.5
5.0
4.5
4.0
3.5
3.0
2.5
2.0
1.5
1.0
0.5
f1 (ppm)

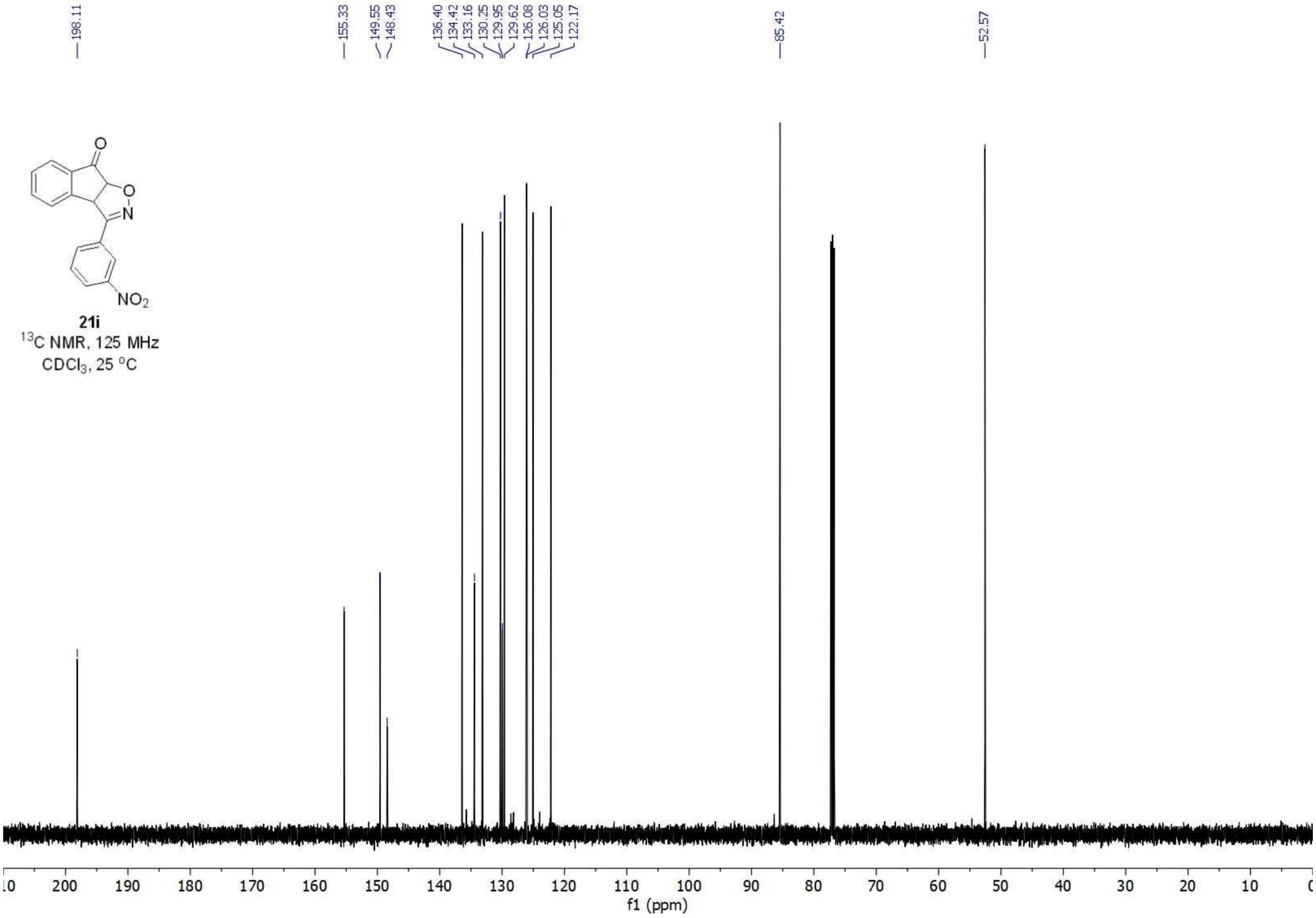
198.11
155.33
149.55
148.43
136.40
134.42
133.16
130.25
129.95
129.62
126.08
126.03
125.05
122.17
85.42
52.57
O
O
N
NO2
21i
13C NMR, 125 MHz
CDCl3, 25 °C
200
190
180
170
160
150
140
130
120
110
100
90
80
70
60
50
40
30
20
10
f1 (ppm)

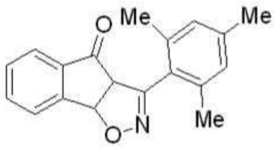

14j

^{1}H NMR, 500 MHz

$CDCl_3$, 25 °C

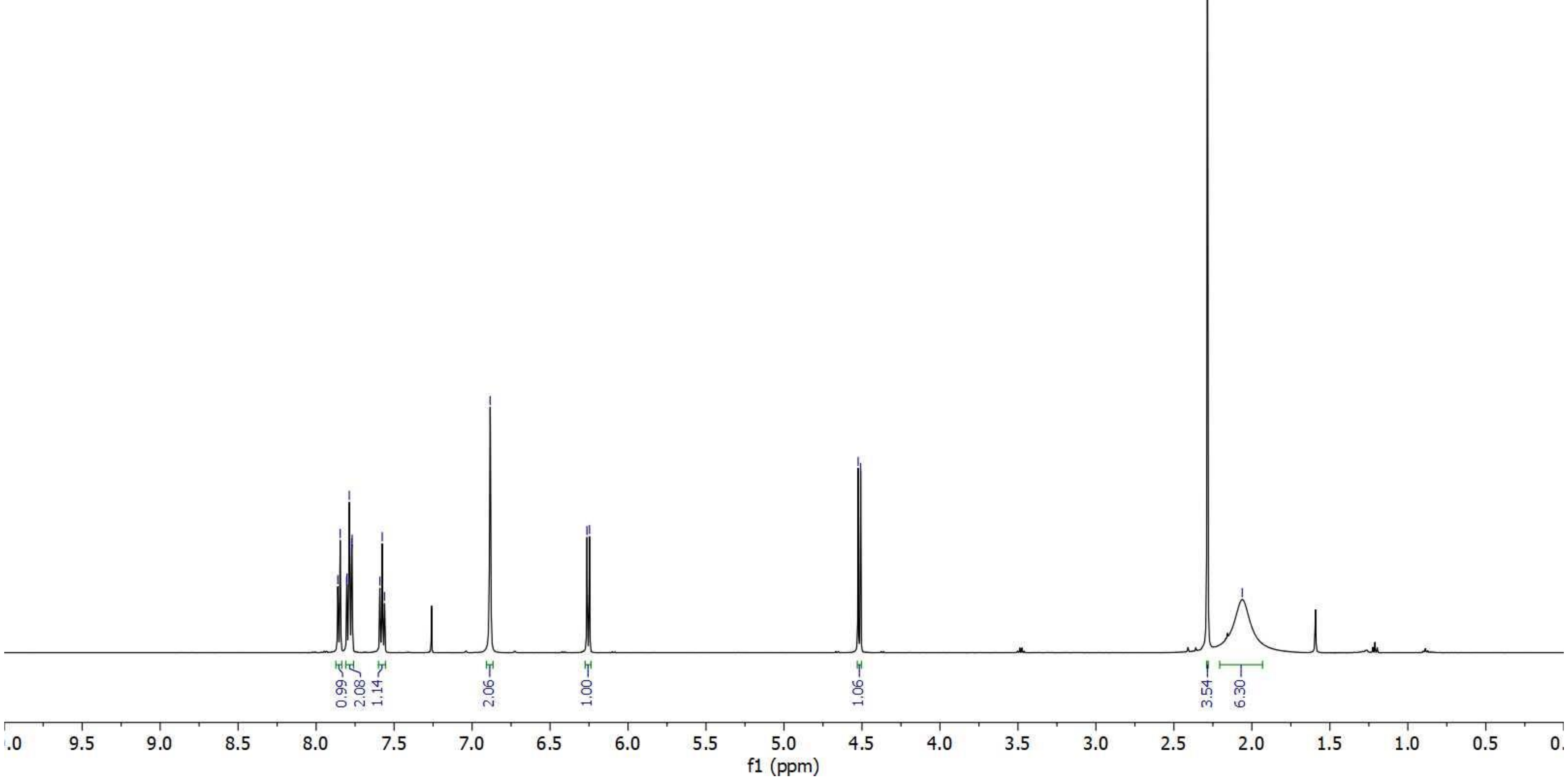

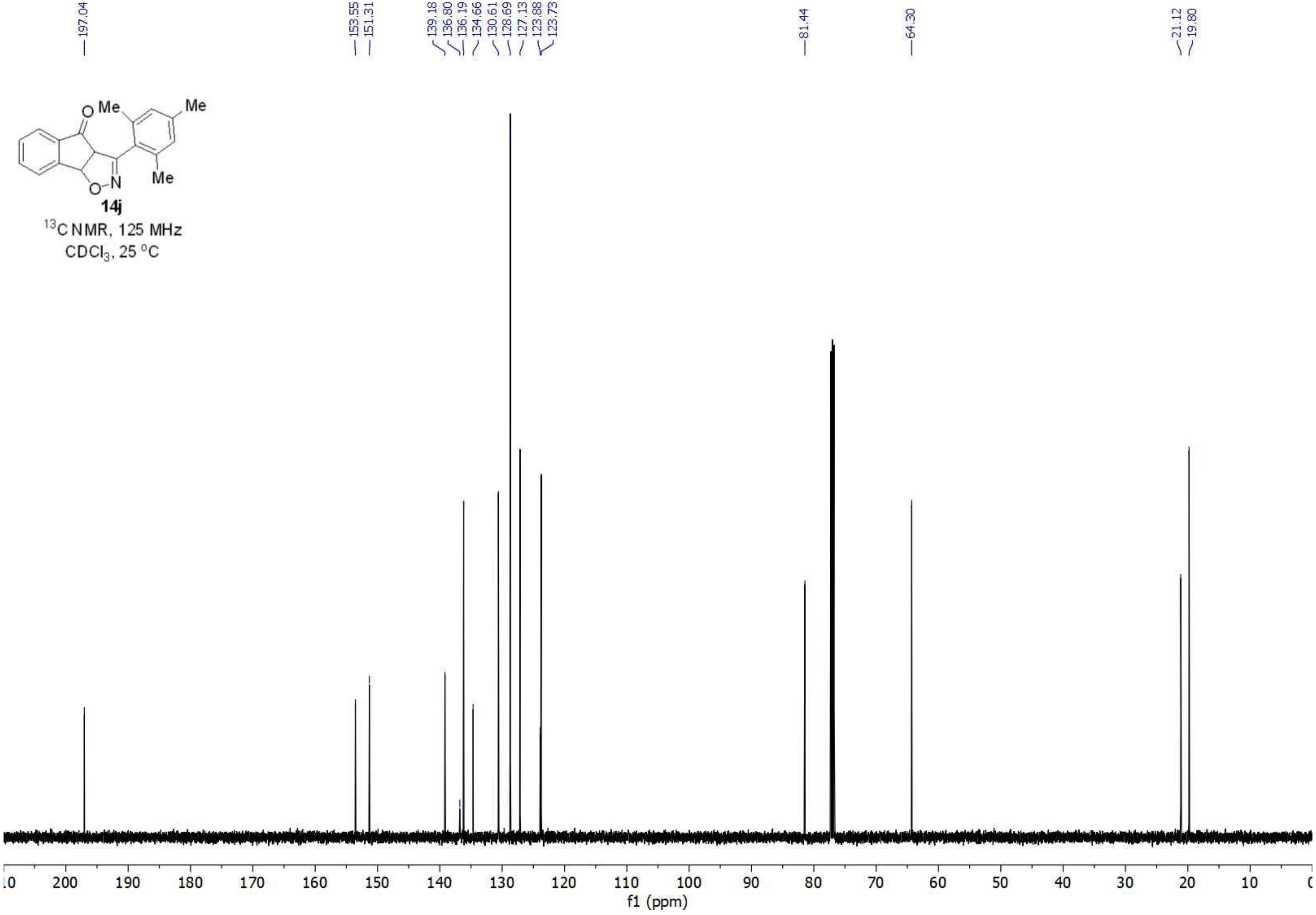
Me
Me
Me
O
O
N
14j
13C NMR, 125 MHz
CDCl3, 25 °C
197.04
153.55
151.31
139.18
136.80
136.19
134.66
130.61
128.69
127.13
123.88
123.73
81.44
64.30
21.12
19.80
f1 (ppm)

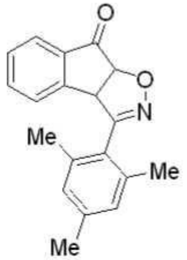

21j
^{1}H NMR, 500 MHz
$CDCl_3$, 25 ^{o}C

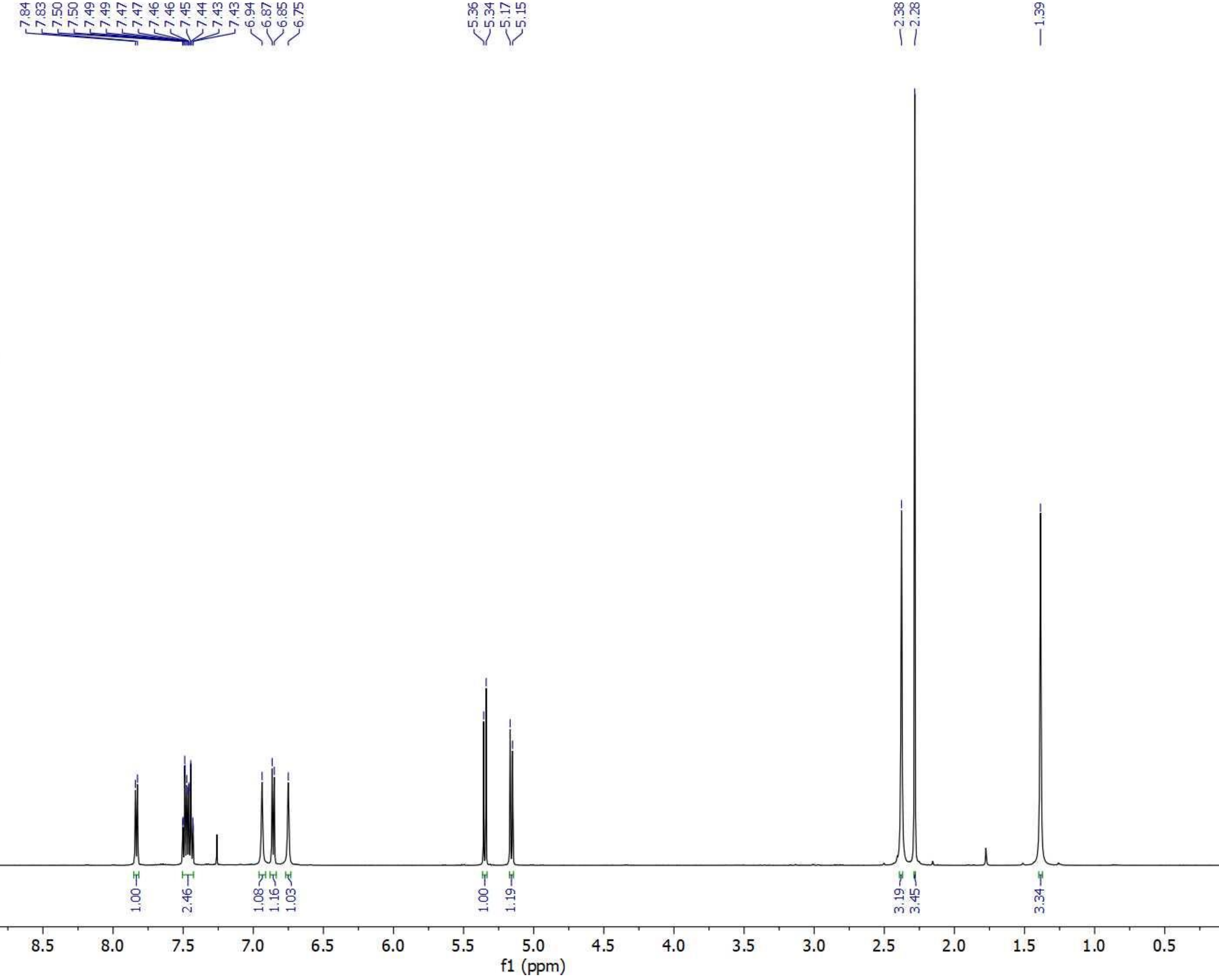

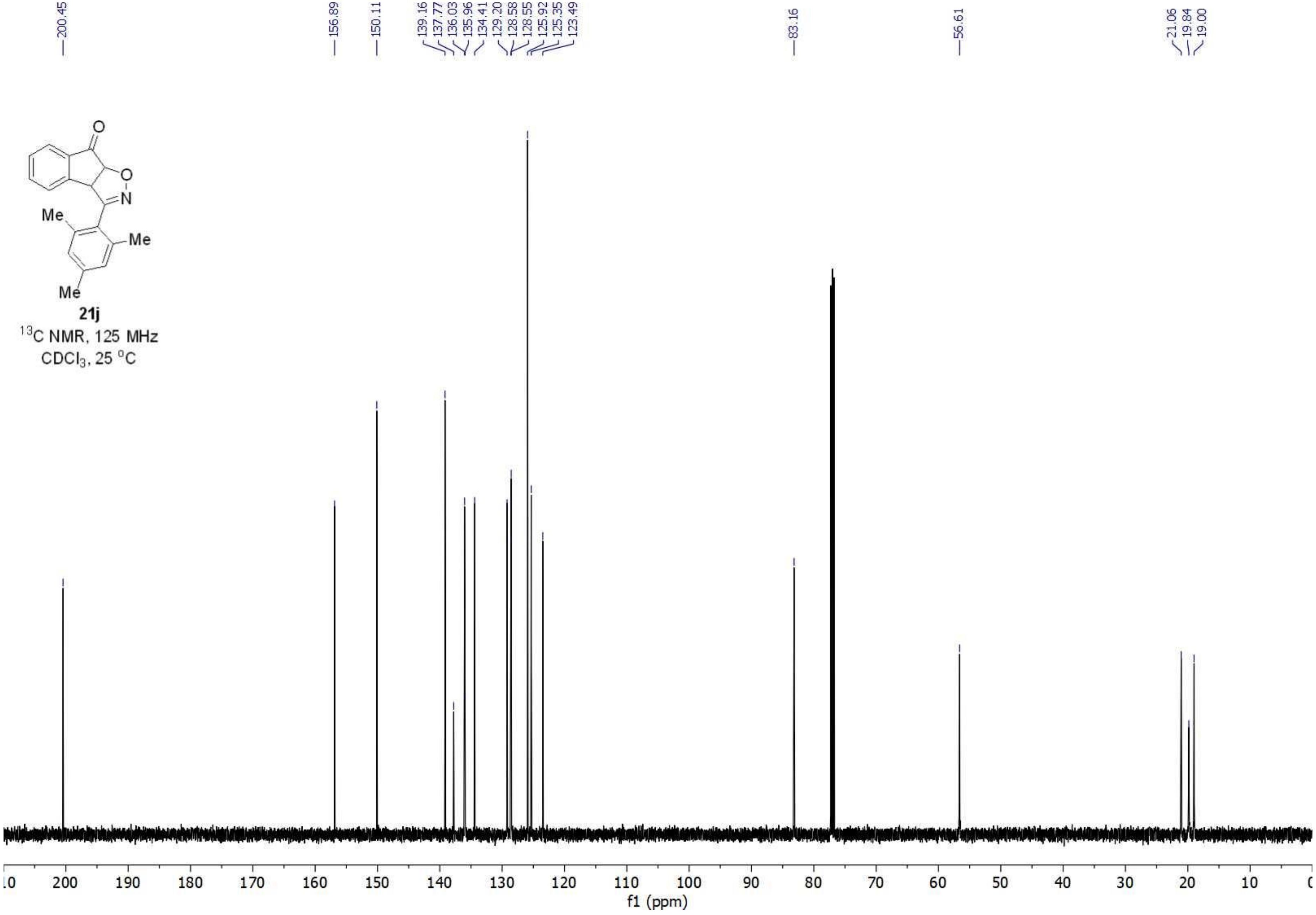
200.45
156.89
150.11
139.16
137.77
136.03
135.96
134.41
129.20
128.58
128.55
125.92
125.35
123.49
83.16
56.61
21.06
19.84
19.00
Me
Me
Me
O
O
N
21j
^{13}C NMR, 125 MHz
$CDCl_3$, 25 °C
f1 (ppm)

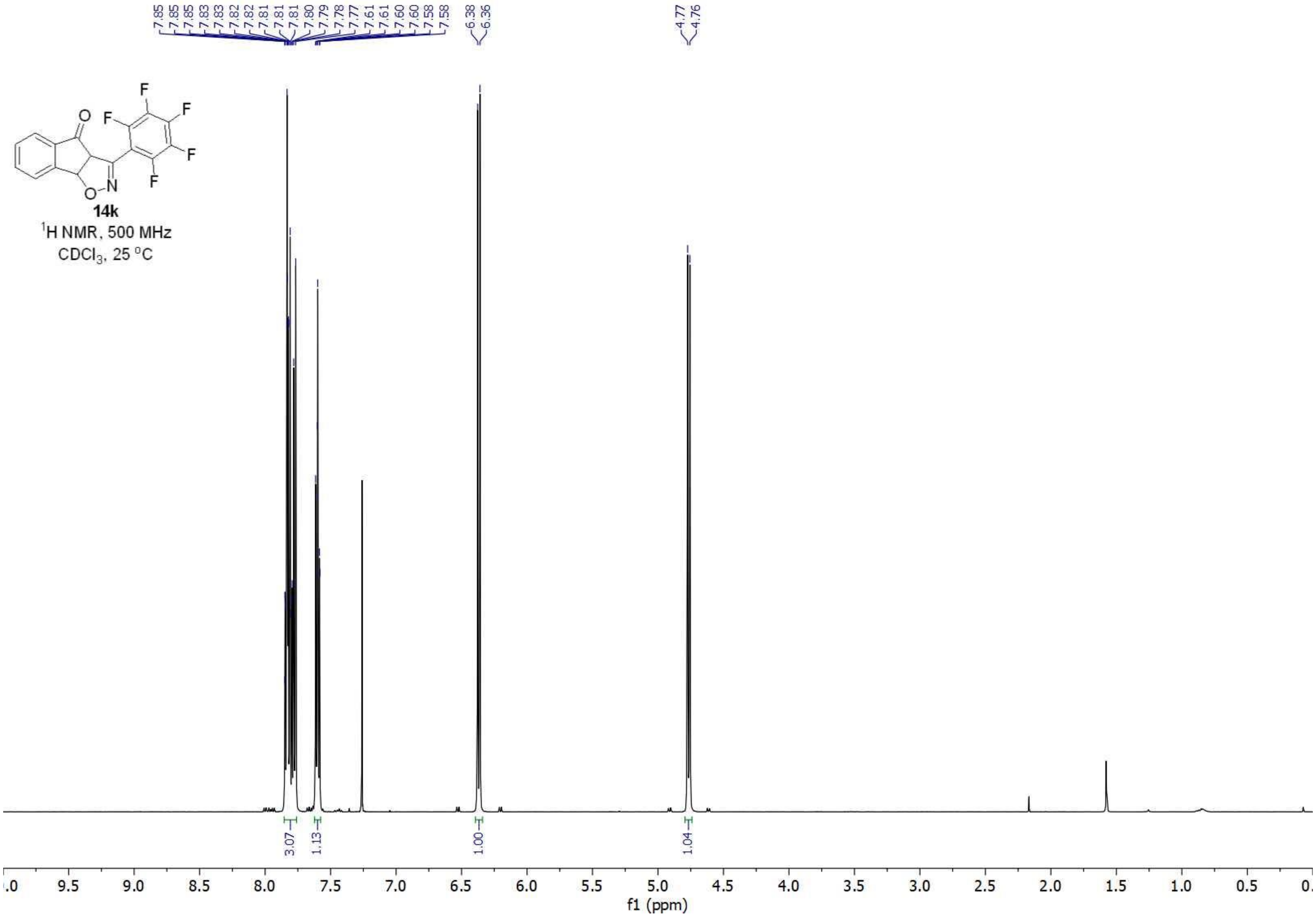
14k
¹H NMR, 500 MHz
CDCl₃, 25 °C
7.85
7.85
7.83
7.83
7.82
7.82
7.81
7.81
7.81
7.80
7.79
7.78
7.77
7.61
7.61
7.60
7.60
7.58
7.58
6.38
6.36
4.77
4.76
3.07
1.13
1.00
1.04
f1 (ppm)

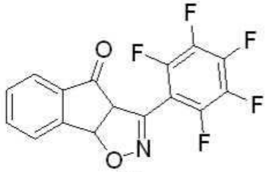

14k

^{13}C NMR, 125 MHz

$CDCl_3$, 25 ^oC

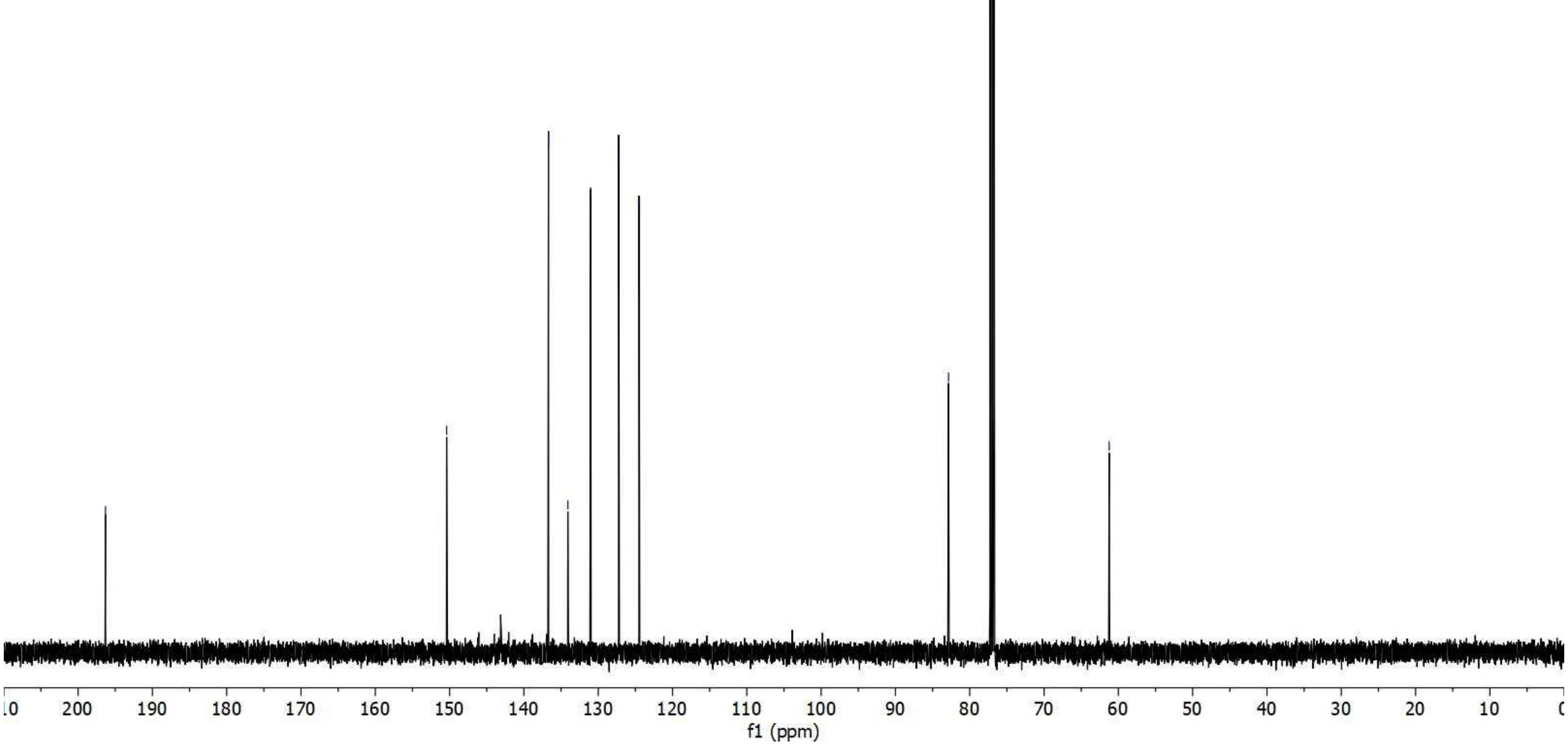

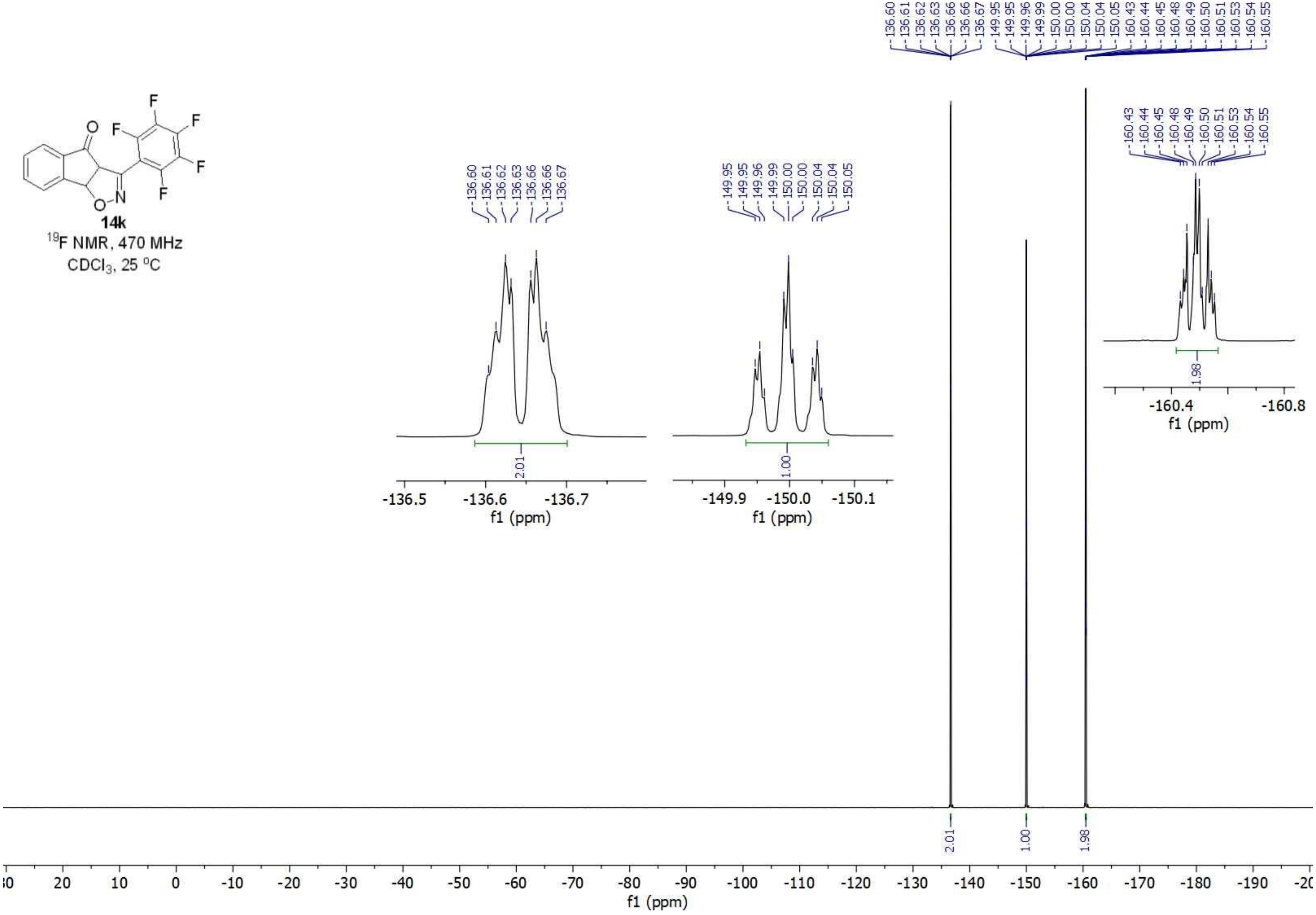
14k
^{19}F NMR, 470 MHz
CDCl$_3$, 25 °C

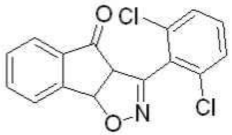

14l

^{1}H NMR, 500 MHz

$CDCl_3$, 25 ^{o}C

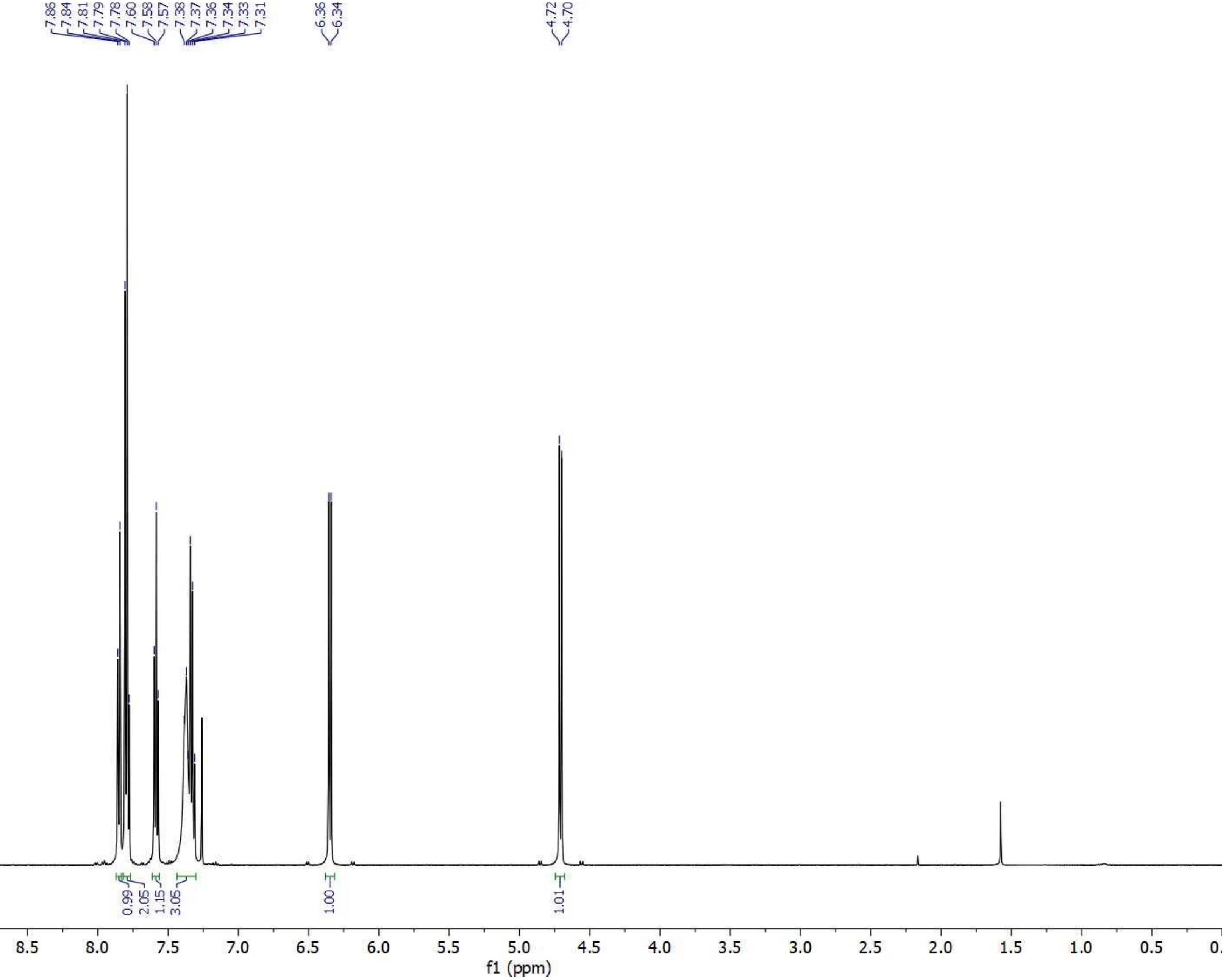

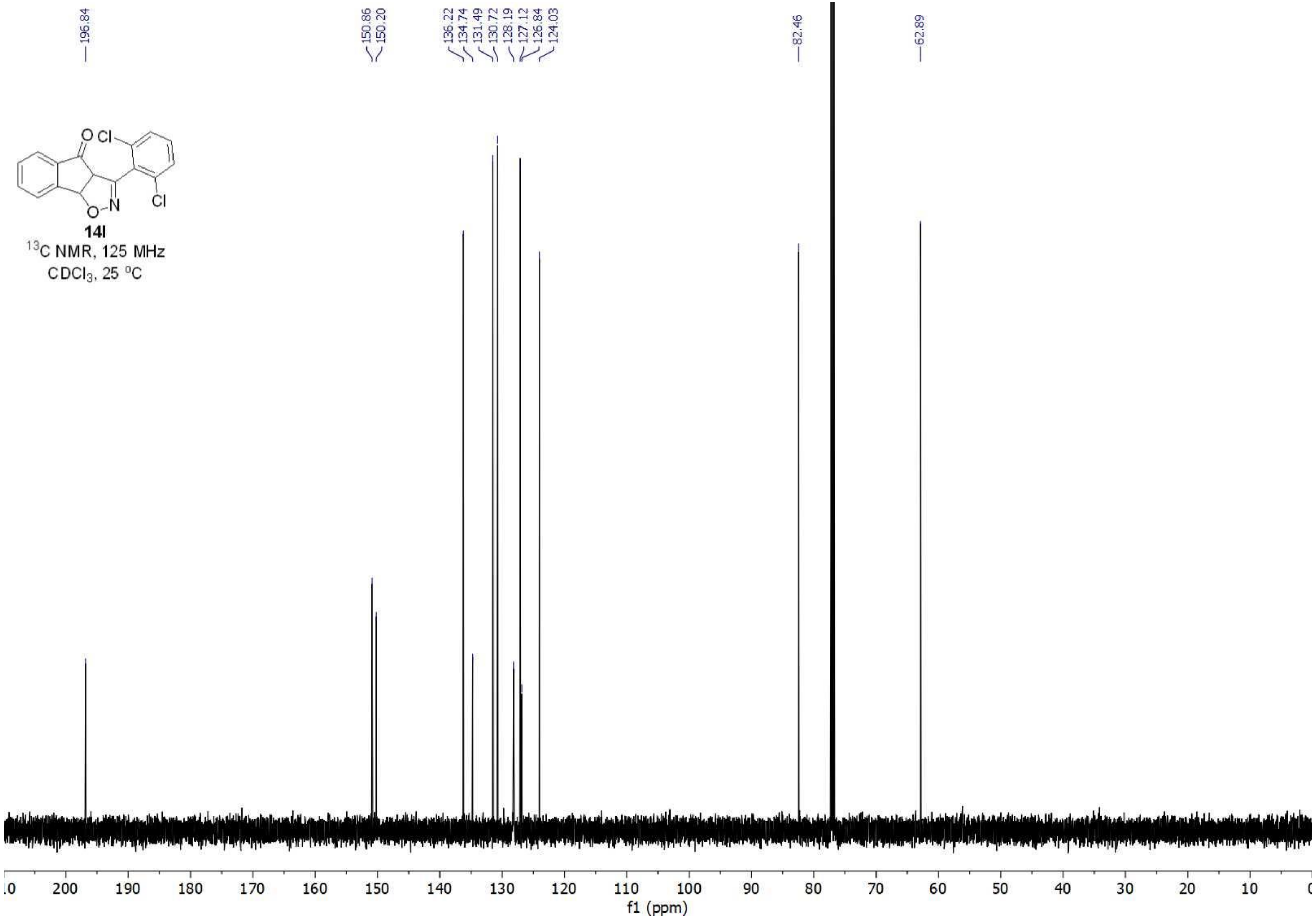
196.84
150.86
150.20
136.22
134.74
131.49
130.72
128.19
127.12
126.84
124.03
82.46
62.89
O Cl
N
O
Cl
14l
^{13}C NMR, 125 MHz
$CDCl_3$, 25 °C
210 200 190 180 170 160 150 140 130 120 110 100 90 80 70 60 50 40 30 20 10 0
f1 (ppm)

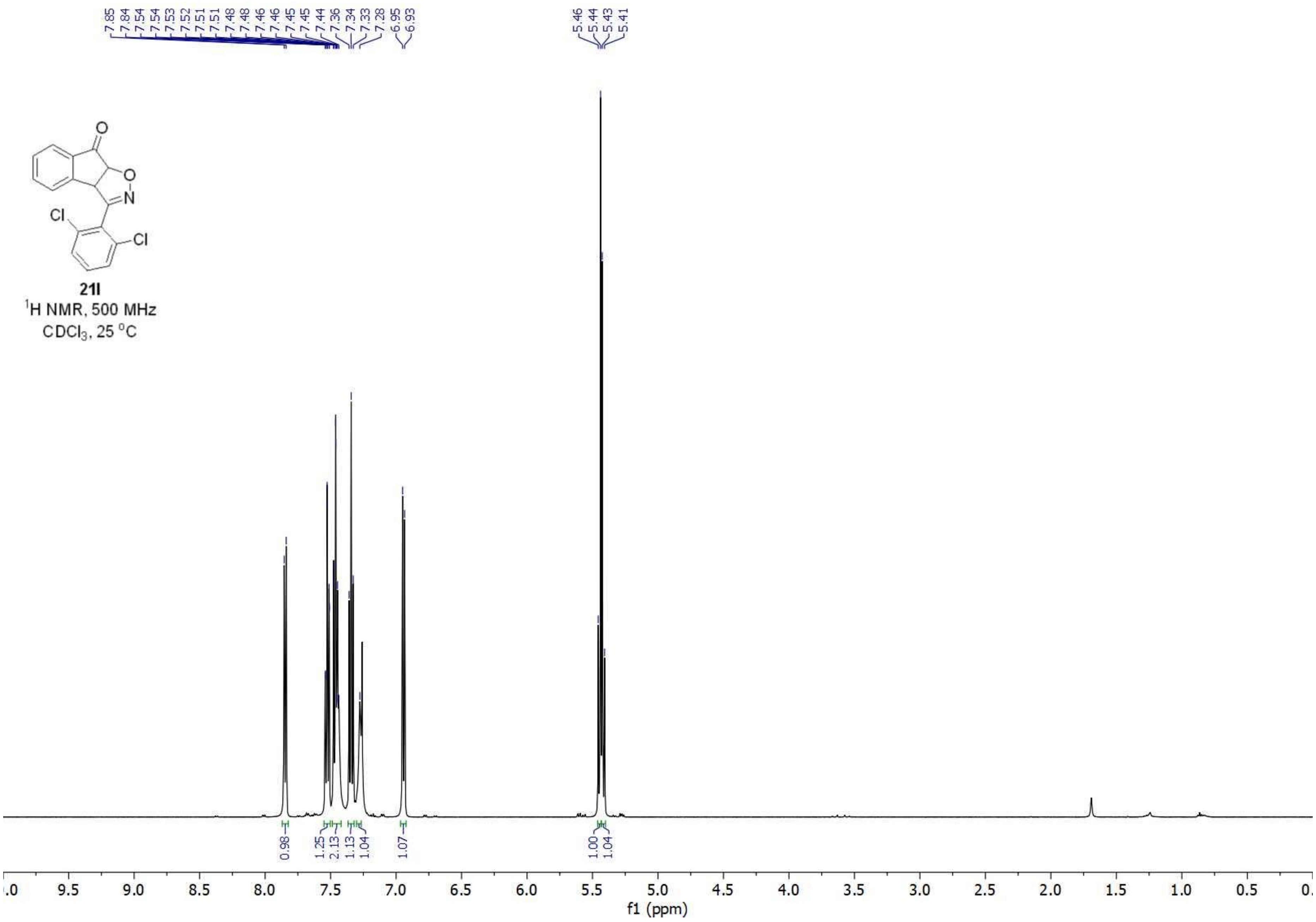
7.85
7.84
7.54
7.54
7.53
7.52
7.51
7.51
7.48
7.48
7.46
7.46
7.45
7.45
7.44
7.36
7.34
7.33
7.28
6.95
6.93
5.46
5.44
5.43
5.41
O
O
N
Cl
Cl
21l
^{1}H NMR, 500 MHz
$CDCl_3$, 25 °C
0.98
1.25
2.13
1.13
1.04
1.07
1.00
1.04
f1 (ppm)

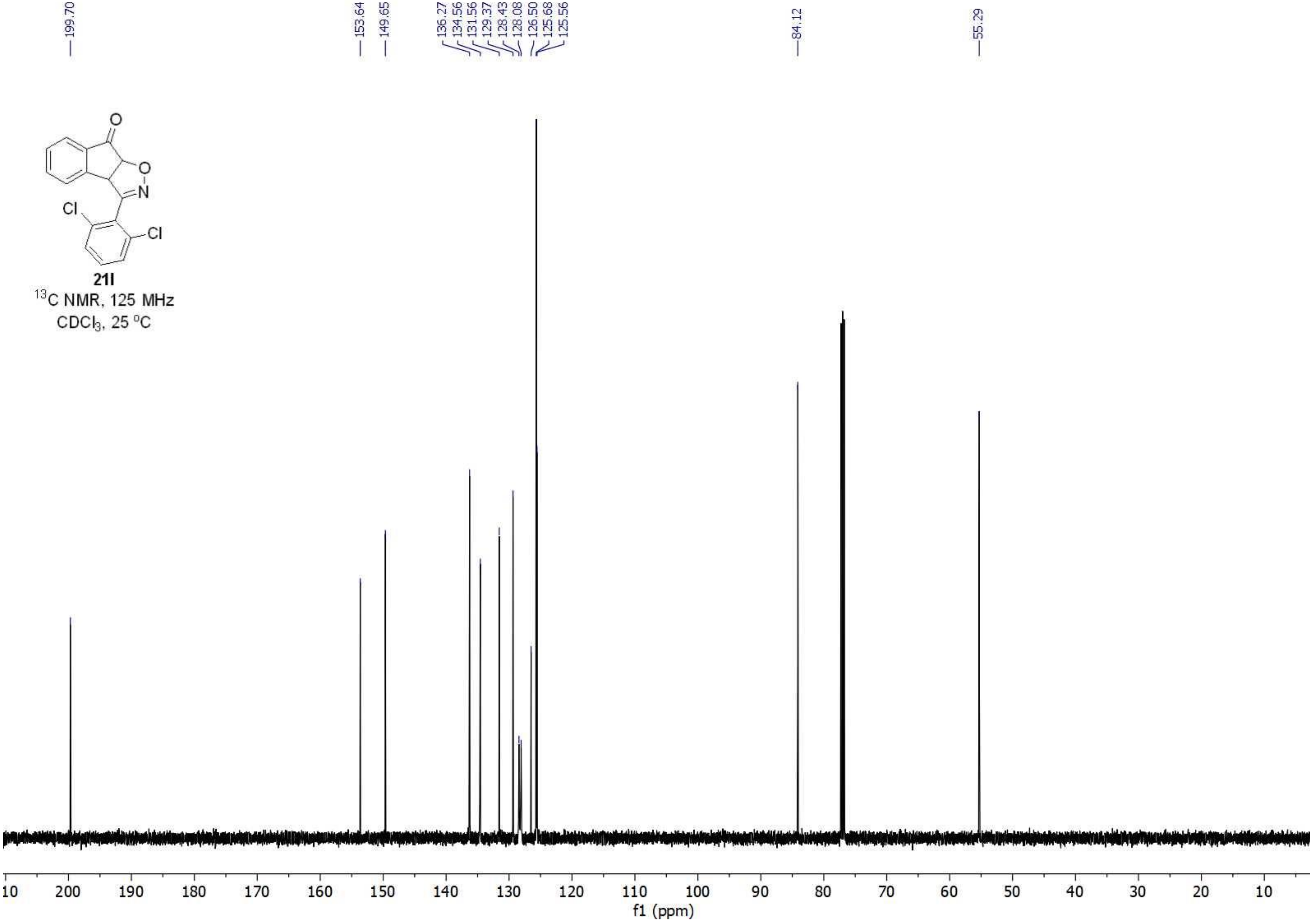
199.70
153.64
149.65
136.27
134.56
131.56
129.37
128.43
128.08
126.50
125.68
125.56
84.12
55.29
O
O
N
Cl
Cl
21l
^{13}C NMR, 125 MHz
$CDCl_3$, 25 °C
10 200 190 180 170 160 150 140 130 120 110 100 90 80 70 60 50 40 30 20 10
f1 (ppm)

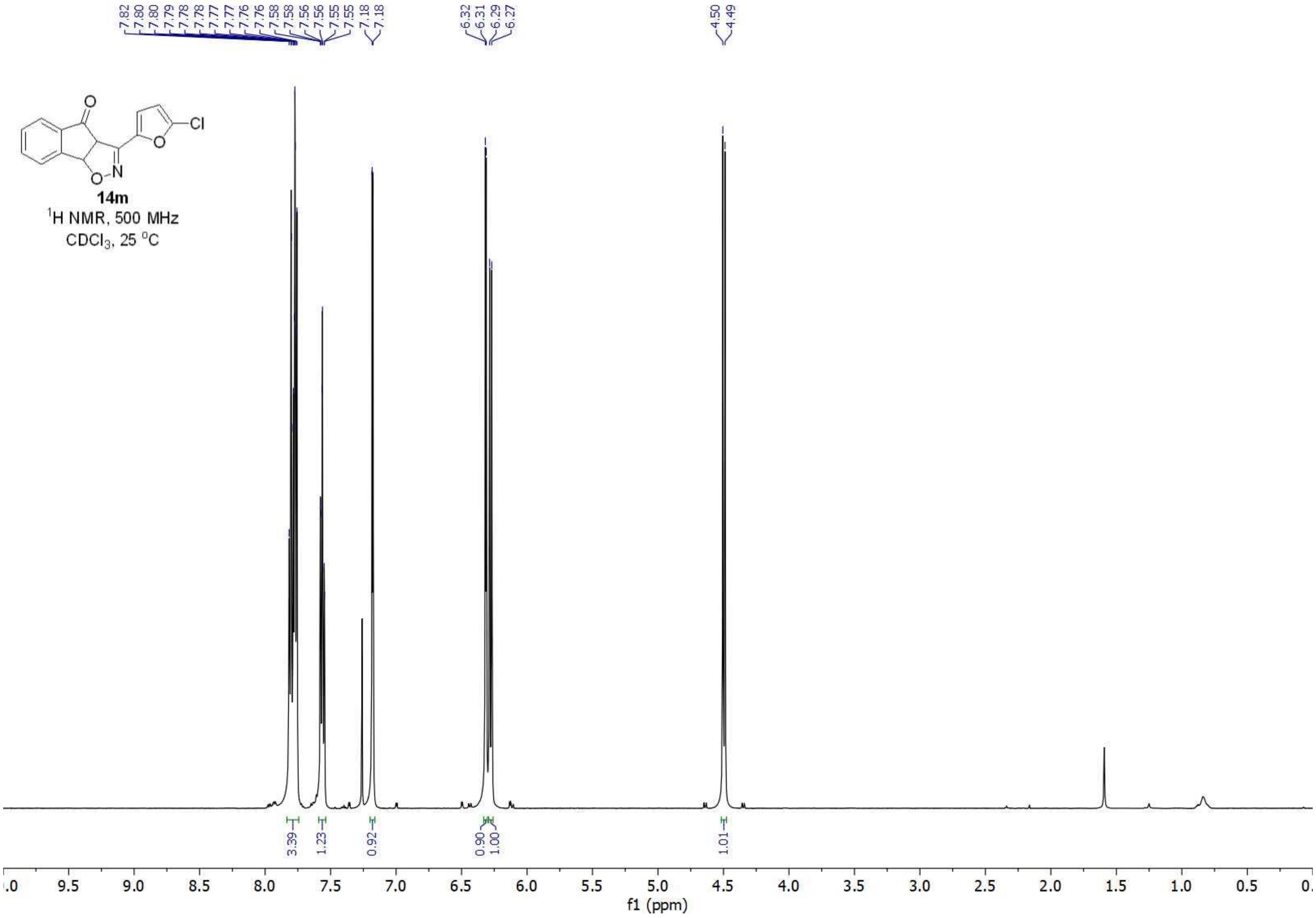
7.82
7.80
7.80
7.79
7.78
7.78
7.77
7.77
7.76
7.76
7.58
7.58
7.56
7.56
7.55
7.55
7.18
7.18
6.32
6.31
6.29
6.27
4.50
4.49
O
Cl
O
O
N
14m
^{1}H NMR, 500 MHz
CDCl3, 25 °C
3.39
1.23
0.92
0.90
1.00
1.01
f1 (ppm)

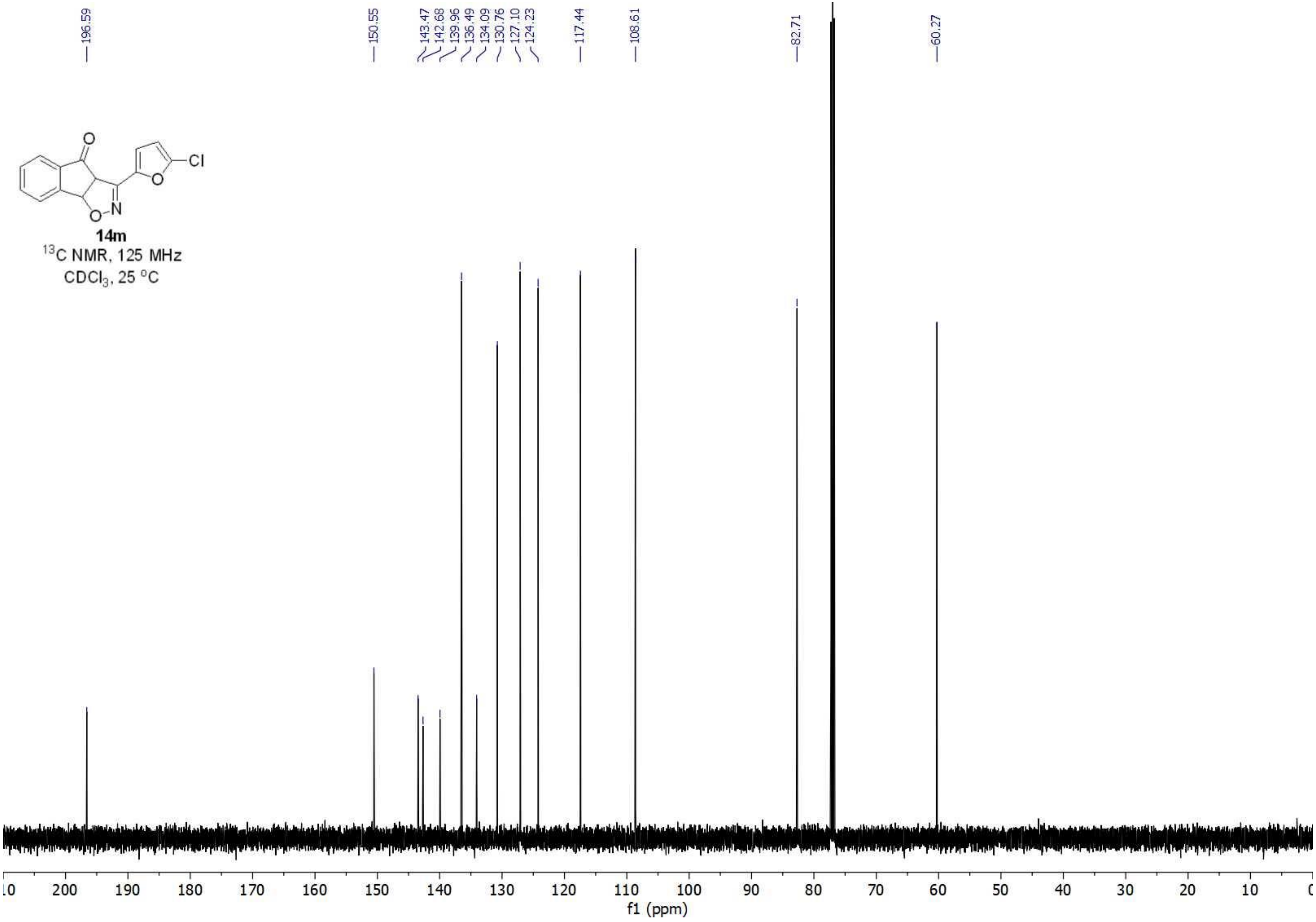
196.59
150.55
143.47
142.68
139.96
136.49
134.09
130.76
127.10
124.23
117.44
108.61
82.71
60.27
Cl
O
N
14m
^{13}C NMR, 125 MHz
$CDCl_3$, 25 °C
f1 (ppm)

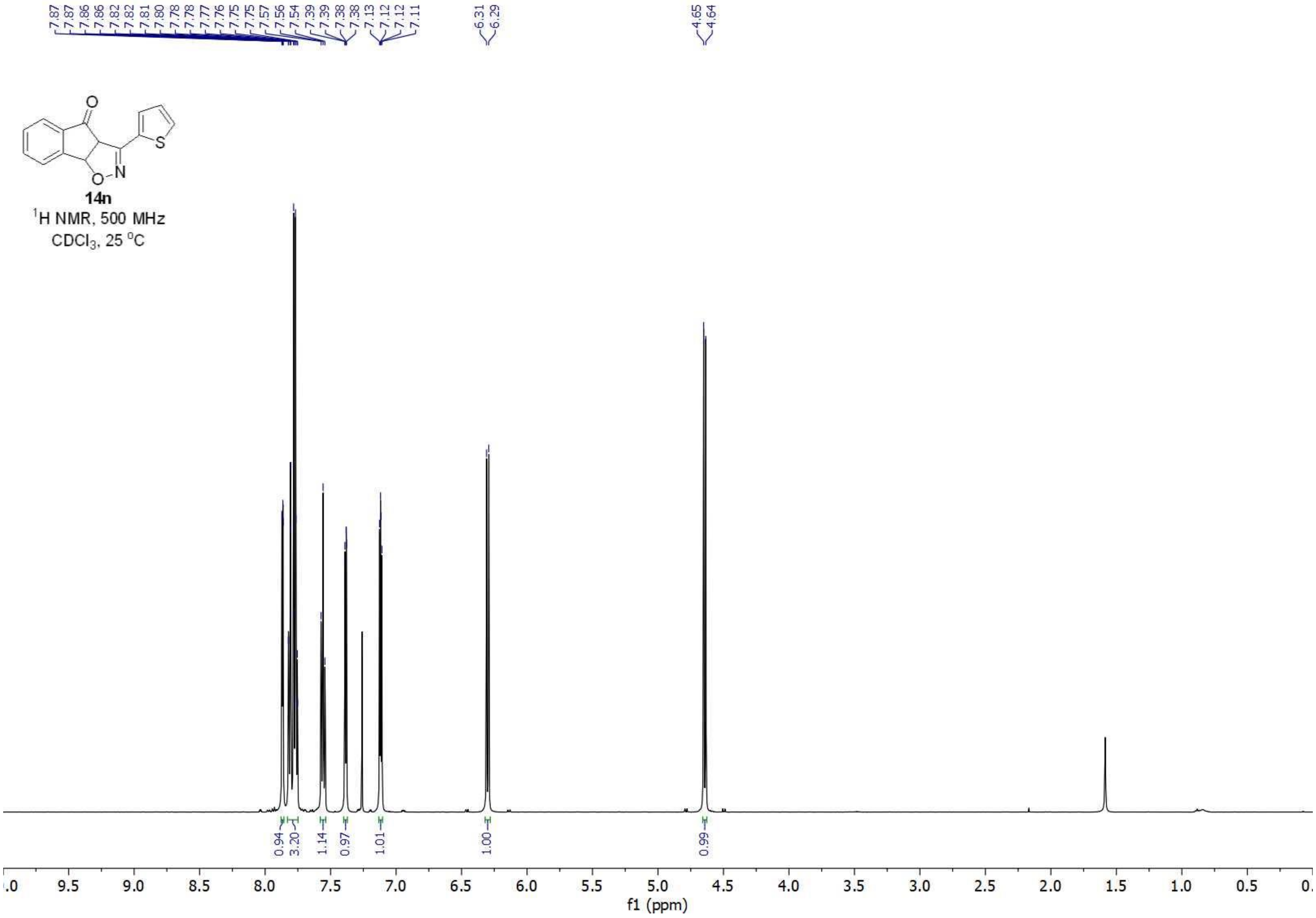
7.87
7.87
7.86
7.86
7.82
7.82
7.81
7.80
7.78
7.78
7.77
7.76
7.75
7.75
7.57
7.56
7.54
7.39
7.39
7.38
7.38
7.13
7.12
7.12
7.11
6.31
6.29
4.65
4.64
O
S
N
14n
^{1}H NMR, 500 MHz
$CDCl_3$, 25 °C
0.94
3.20
1.14
0.97
1.01
1.00
0.99
9.5
9.0
8.5
8.0
7.5
7.0
6.5
6.0
5.5
5.0
4.5
4.0
3.5
3.0
2.5
2.0
1.5
1.0
0.5
f1 (ppm)

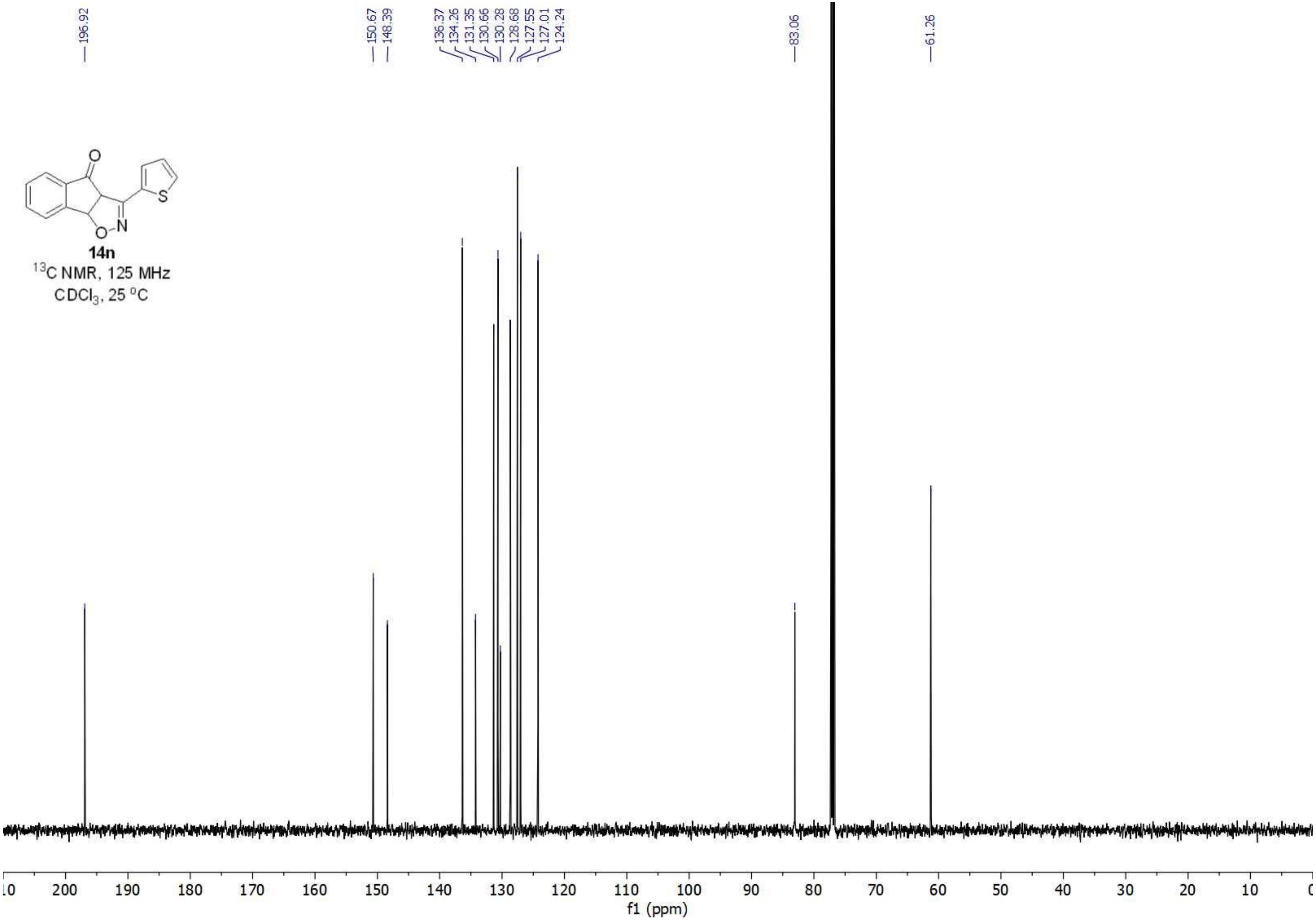
196.92
150.67
148.39
136.37
134.26
131.35
130.66
130.28
128.68
127.55
127.01
124.24
83.06
61.26
O
S
O
N
14n
^{13}C NMR, 125 MHz
$CDCl_3$, 25 °C
200
190
180
170
160
150
140
130
120
110
100
90
80
70
60
50
40
30
20
10
f1 (ppm)

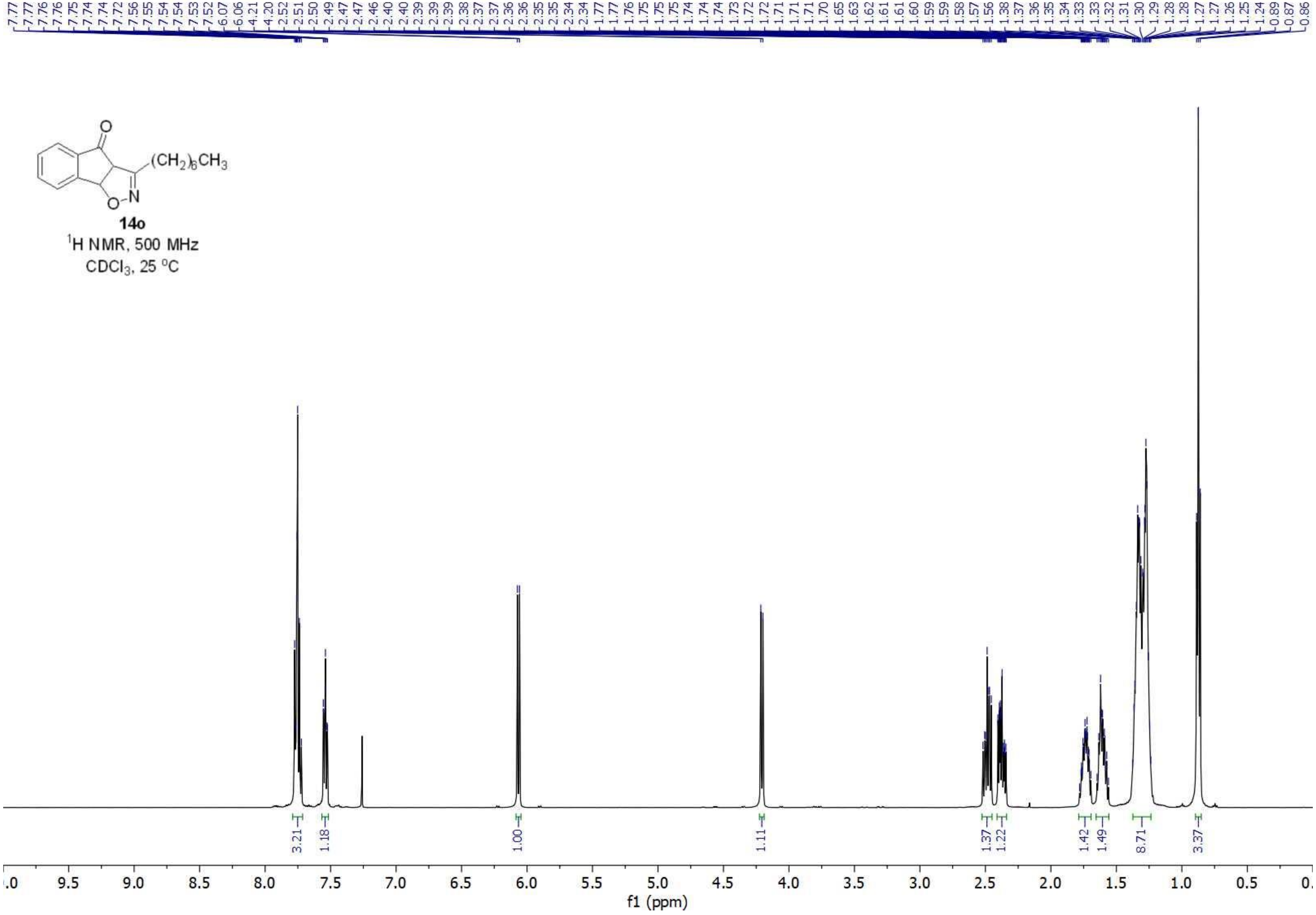
(CH2)6CH3
O
N
O
14o
1H NMR, 500 MHz
CDCl3, 25 °C
f1 (ppm)

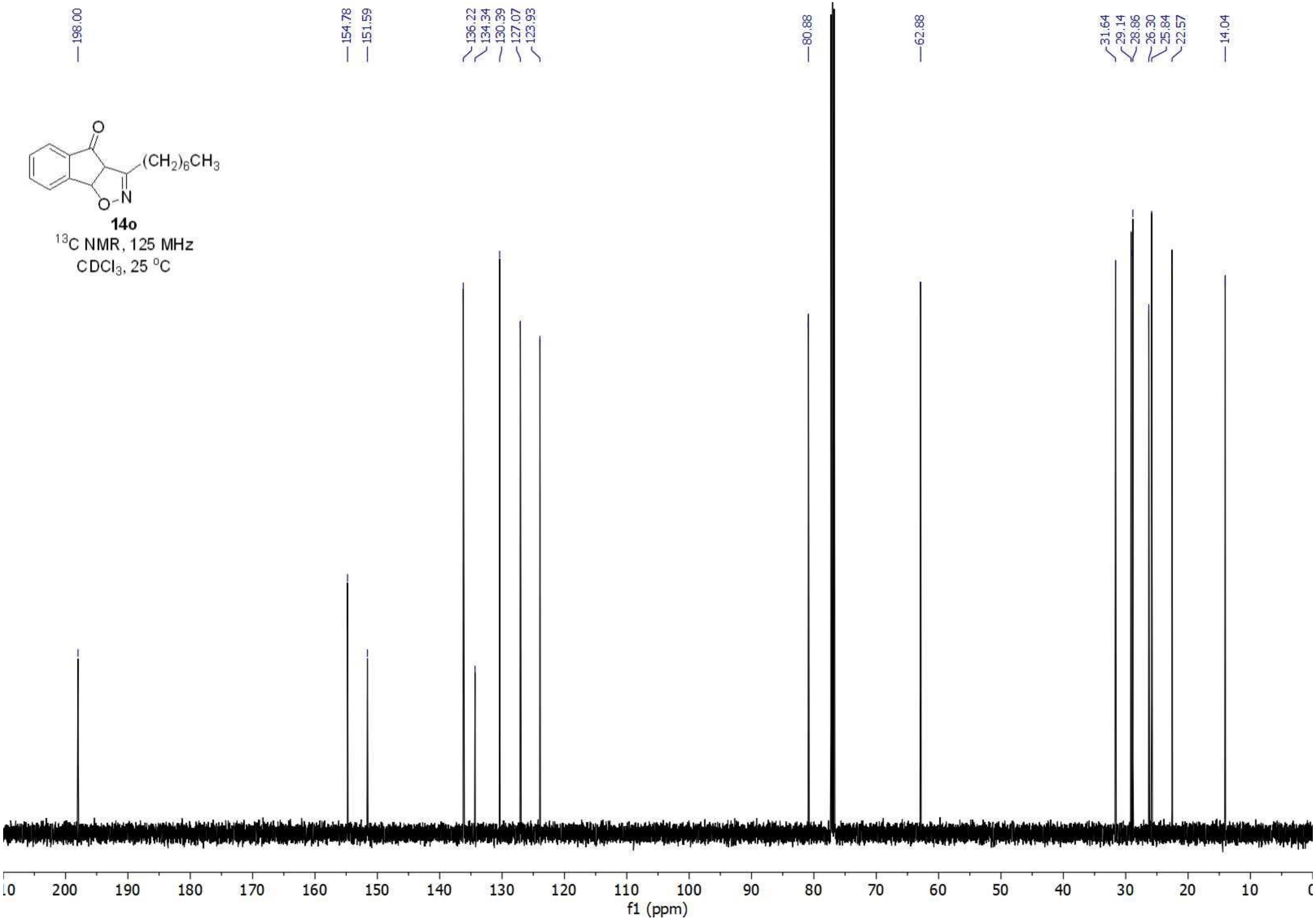
198.00
154.78
151.59
136.22
134.34
130.39
127.07
123.93
80.88
62.88
31.64
29.14
28.86
26.30
25.84
22.57
14.04
O
(CH2)6CH3
N
O
14o
13C NMR, 125 MHz
CDCl3, 25 °C
200
190
180
170
160
150
140
130
120
110
100
90
80
70
60
50
40
30
20
10
f1 (ppm)

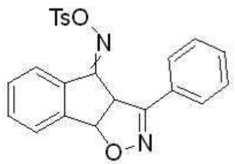

13a

^{1}H NMR, 500 MHz

$CDCl_3$, 25 ^{o}C

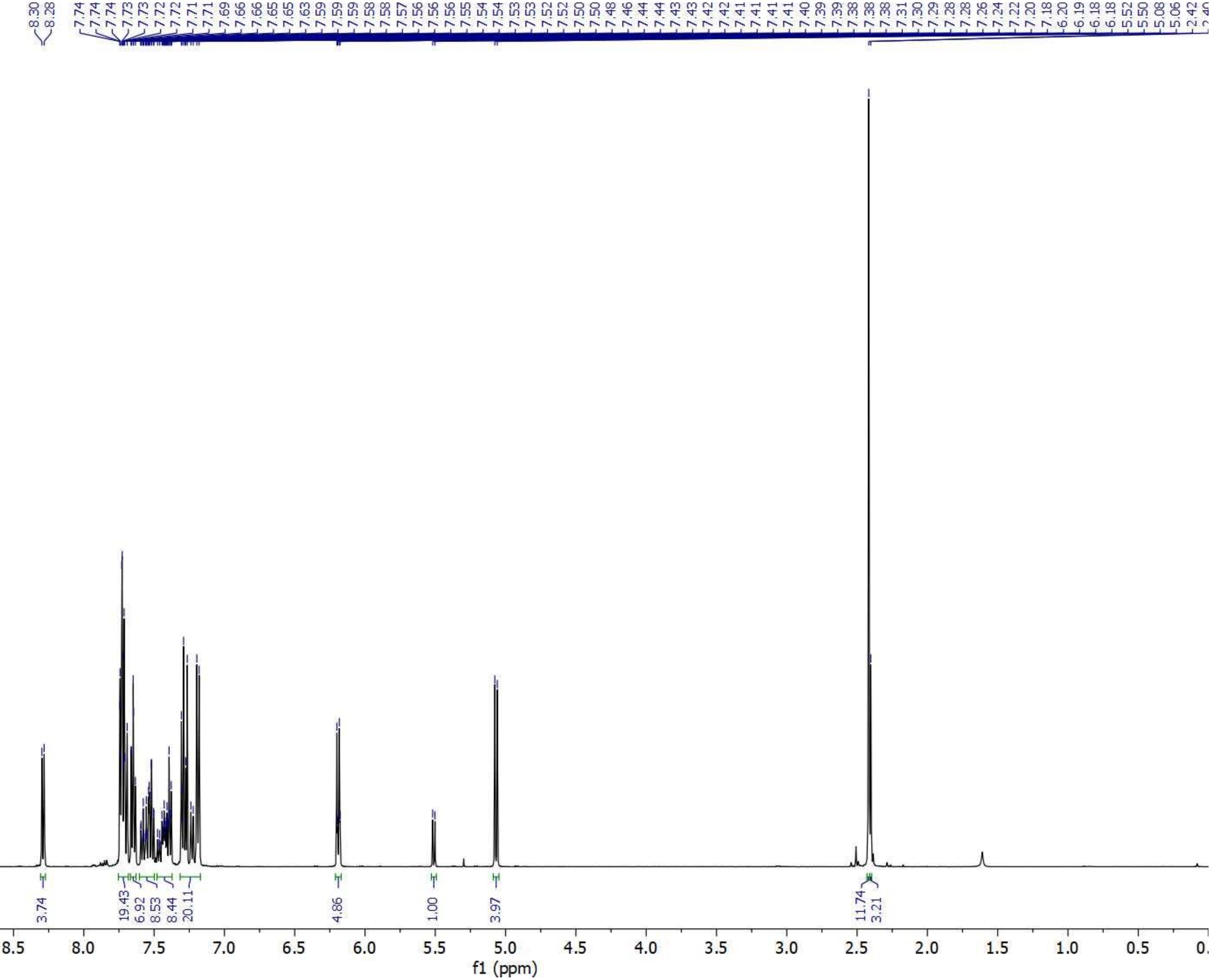

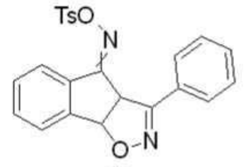

13a

^{13}C NMR, 125 MHz

$CDCl_3$, 25 °C

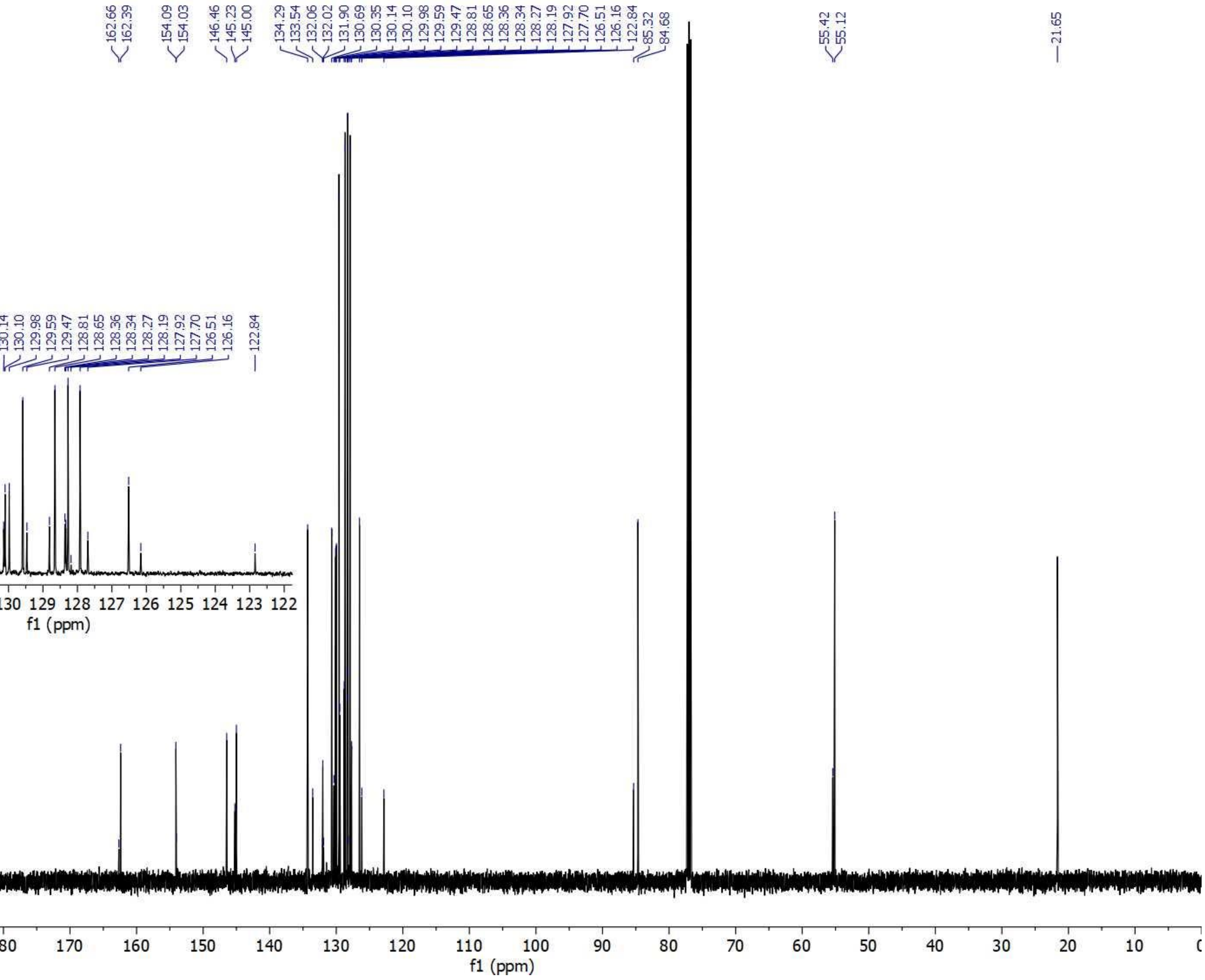

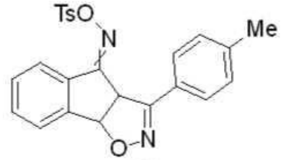

13b
^{1}H NMR, 500 MHz
$CDCl_3$, 25 ^oC

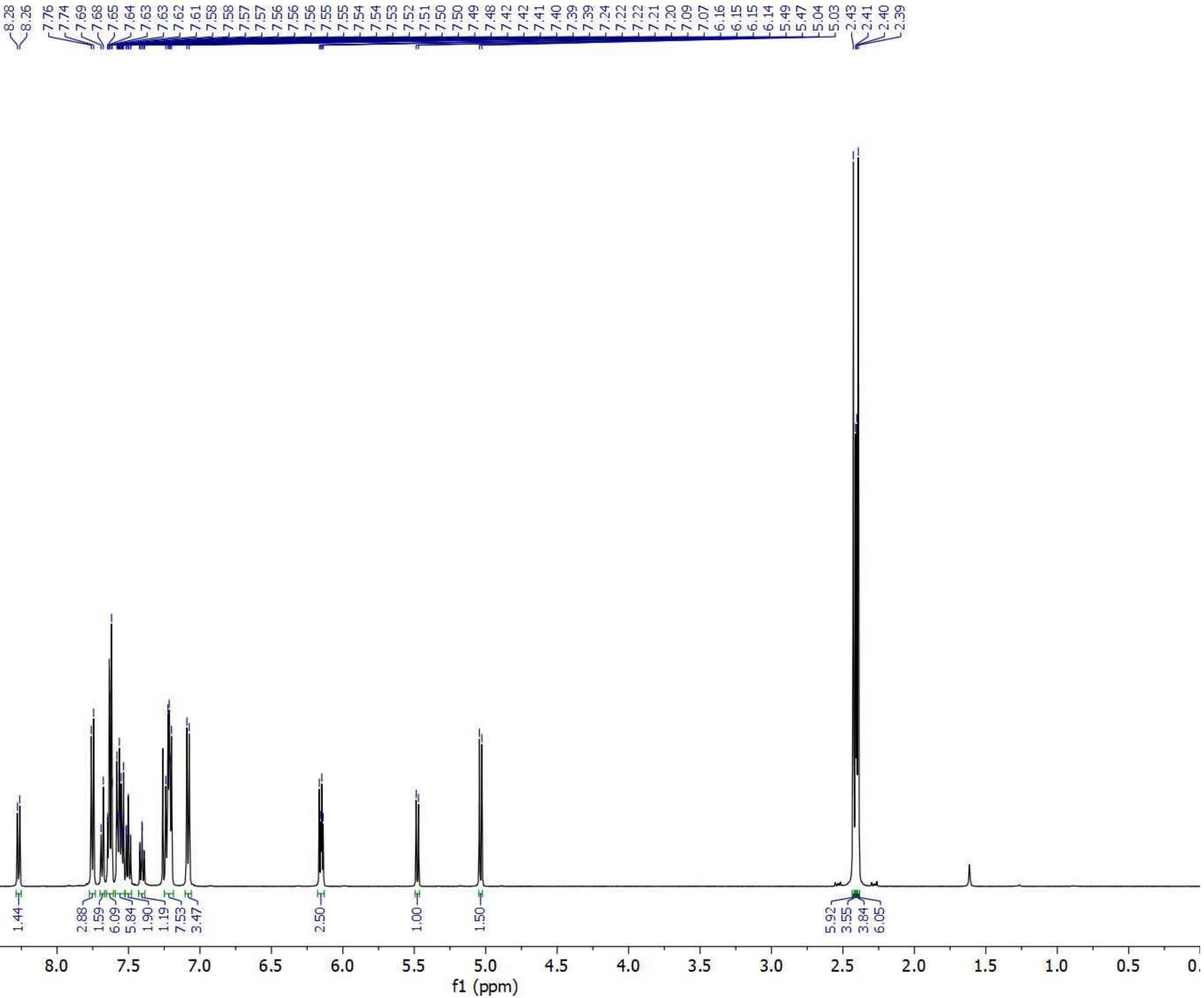

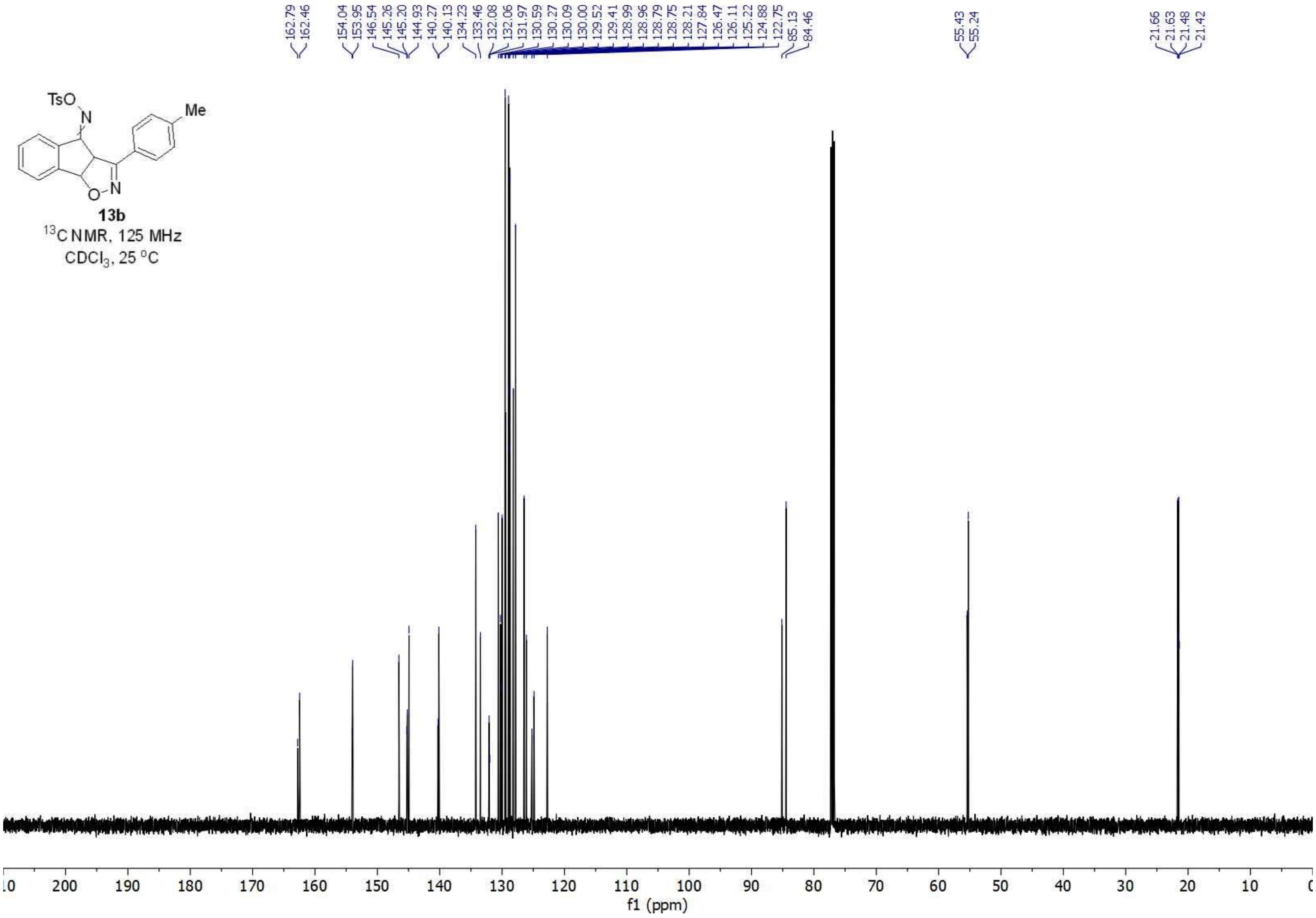

TsO
N
Me
O
N
13b
^{13}C NMR, 125 MHz
CDCl$_3$, 25 °C
162.79
162.46
154.04
153.95
146.54
145.26
145.20
144.93
140.27
140.13
134.23
133.46
132.08
132.06
131.97
130.59
130.27
130.09
130.00
129.52
129.41
128.99
128.96
128.79
128.75
128.21
127.84
126.47
126.11
125.22
124.88
122.75
85.13
84.46
55.43
55.24
21.66
21.63
21.48
21.42
200
190
180
170
160
150
140
130
120
110
100
90
80
70
60
50
40
30
20
10
f1 (ppm)

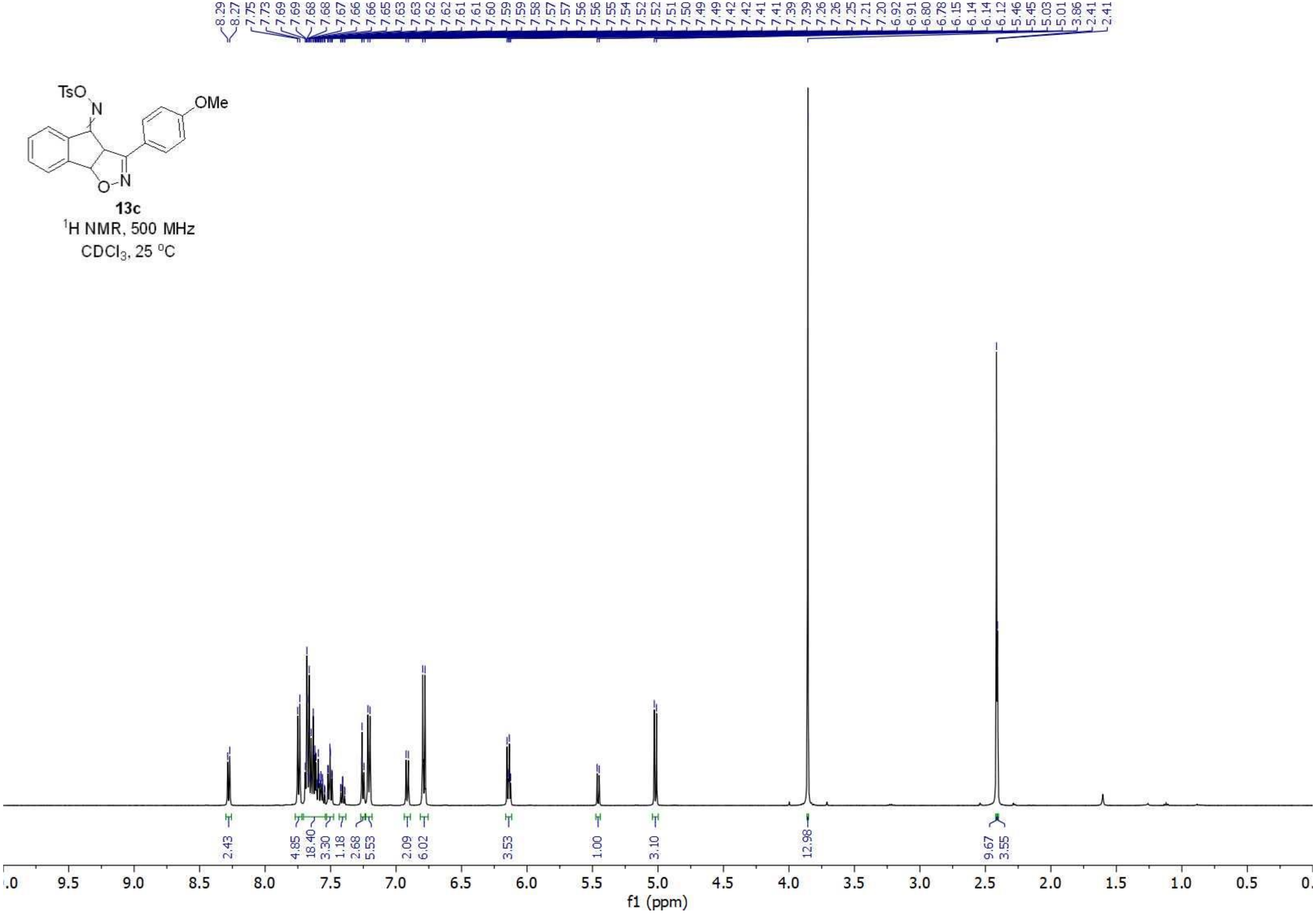
TsO
N
OMe
O
N
13c
1H NMR, 500 MHz
CDCl3, 25 °C
8.29 8.27 7.75 7.73 7.69 7.69 7.68 7.68 7.67 7.66 7.66 7.65 7.63 7.63 7.62 7.62 7.61 7.61 7.60 7.59 7.59 7.58 7.57 7.57 7.56 7.56 7.55 7.54 7.52 7.52 7.51 7.50 7.49 7.49 7.42 7.42 7.41 7.41 7.39 7.39 7.26 7.26 7.25 7.21 7.20 6.92 6.91 6.80 6.78 6.15 6.14 6.14 6.12 5.46 5.45 5.03 5.01 3.86 2.41 2.41
2.43
4.85
18.40
3.30
1.18
2.68
5.53
2.09
6.02
3.53
1.00
3.10
12.98
9.67
3.55
f1 (ppm)

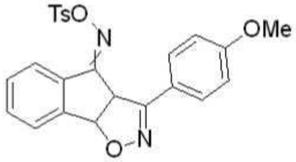

13c

^{13}C NMR, 125 MHz

$CDCl_3$, 25 °C

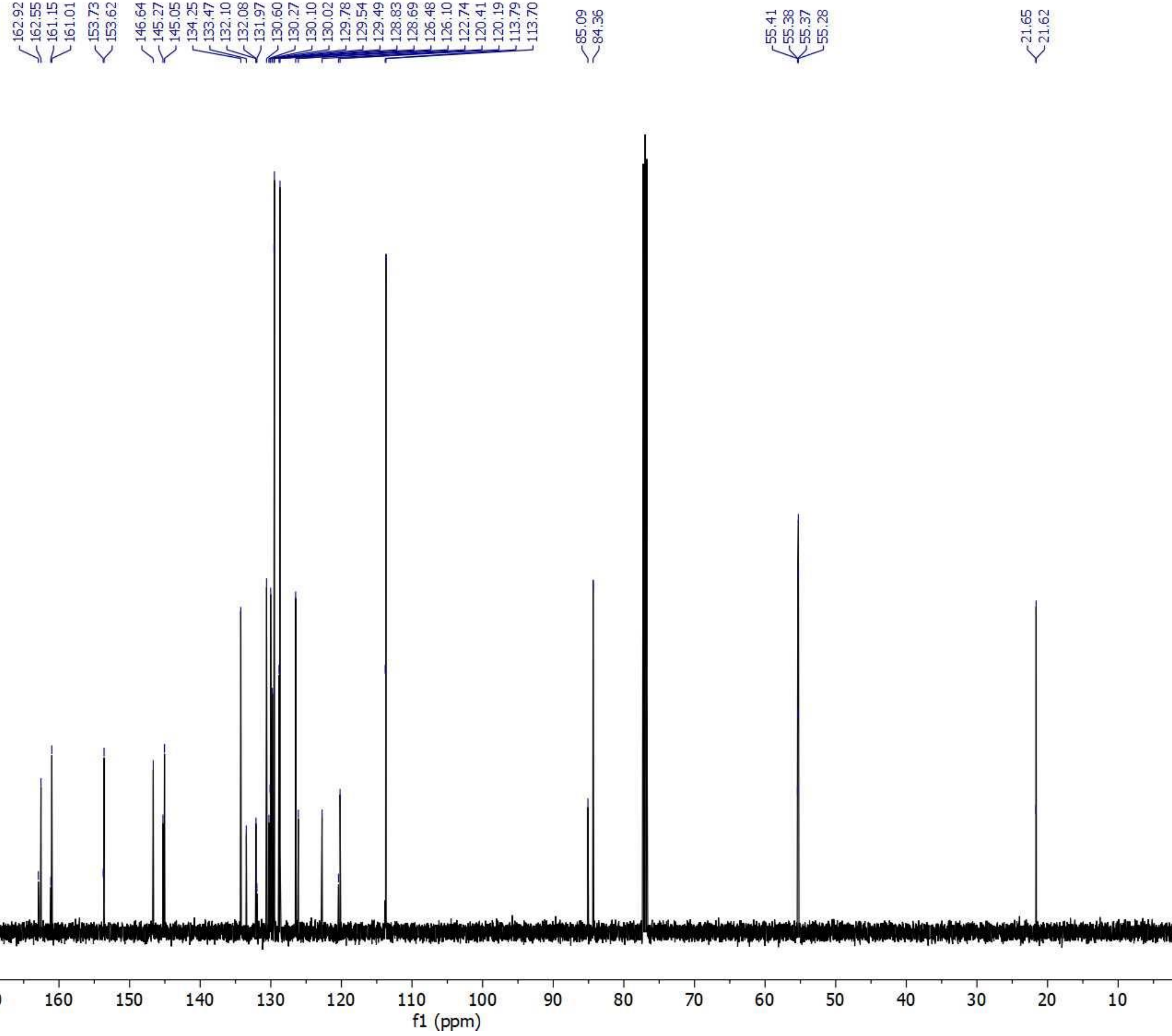

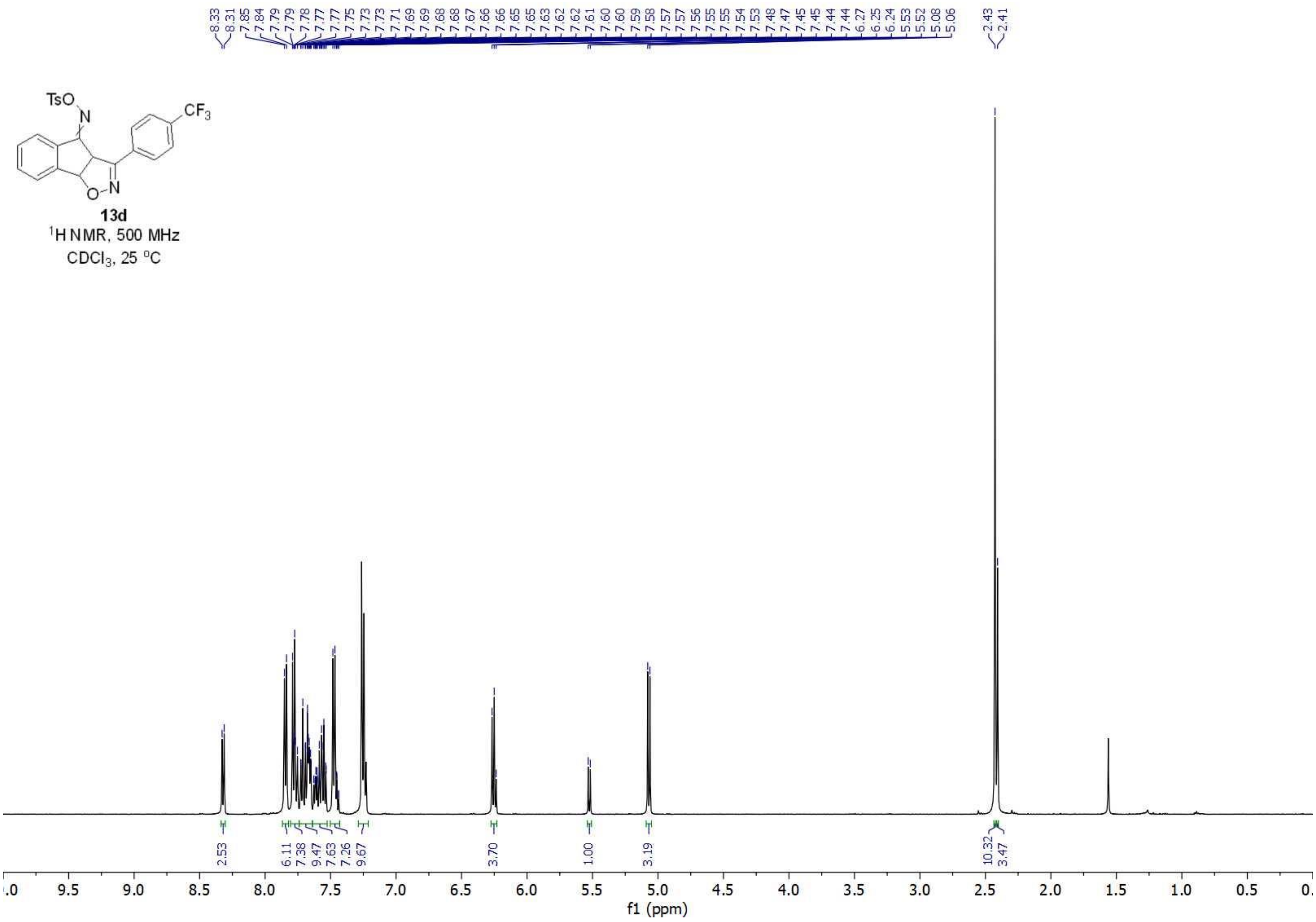
TsO
N
CF3
O–N
13d
1H NMR, 500 MHz
CDCl3, 25 °C
f1 (ppm)

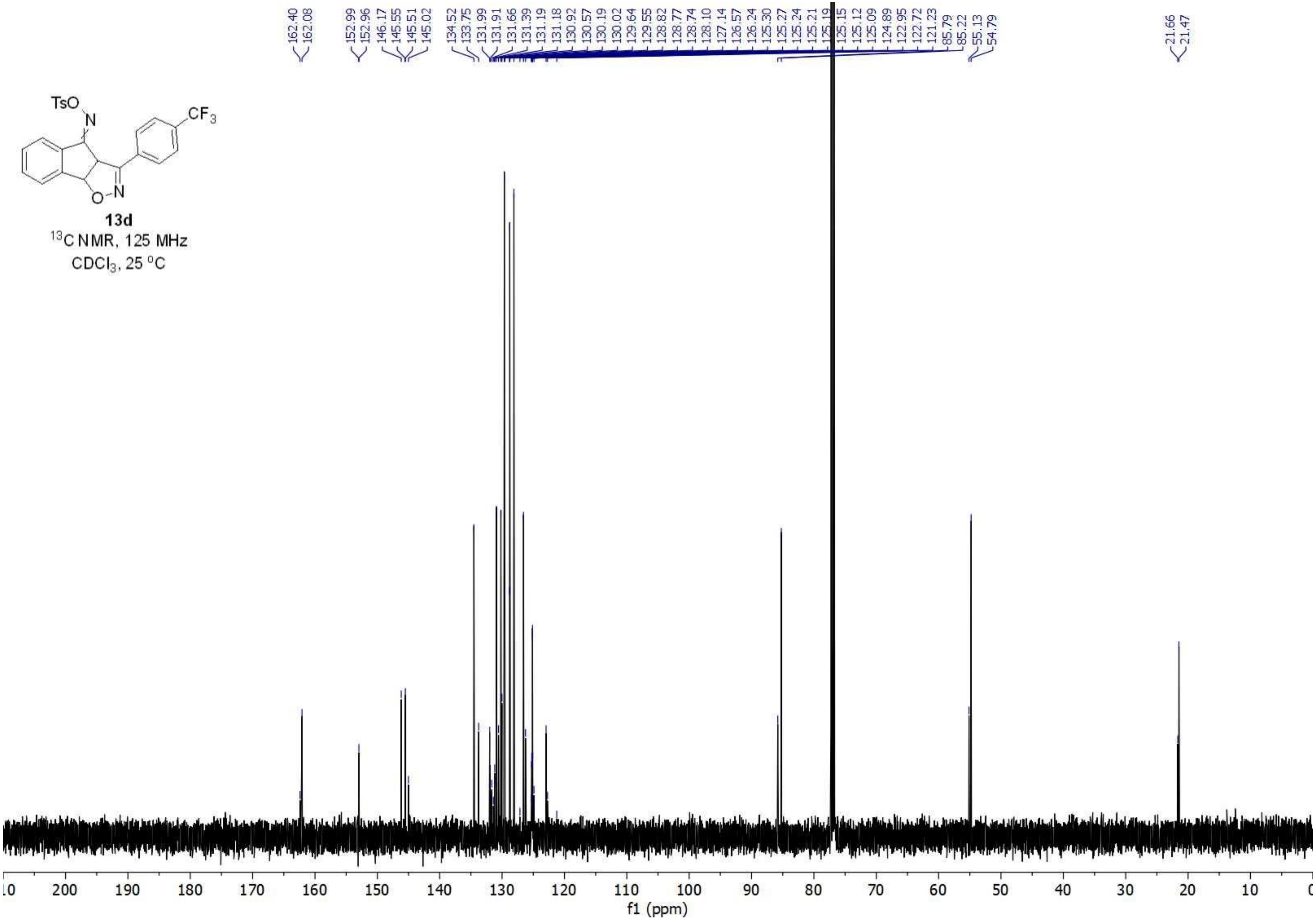
TsO
N
CF3
O
N
13d
13C NMR, 125 MHz
CDCl3, 25 °C
162.40
162.08
152.99
152.96
146.17
145.55
145.51
145.02
134.52
133.75
131.99
131.91
131.66
131.39
131.19
131.18
130.92
130.57
130.19
130.02
129.64
129.55
128.82
128.77
128.74
128.10
127.14
126.57
126.24
125.30
125.27
125.24
125.21
125.19
125.15
125.12
125.09
124.89
122.95
122.72
121.23
85.79
85.22
55.13
54.79
21.66
21.47
f1 (ppm)

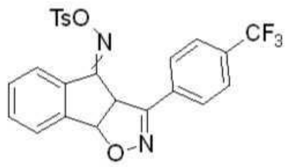

13d

^{19}F NMR, 470 MHz

$CDCl_3$, 25 °C

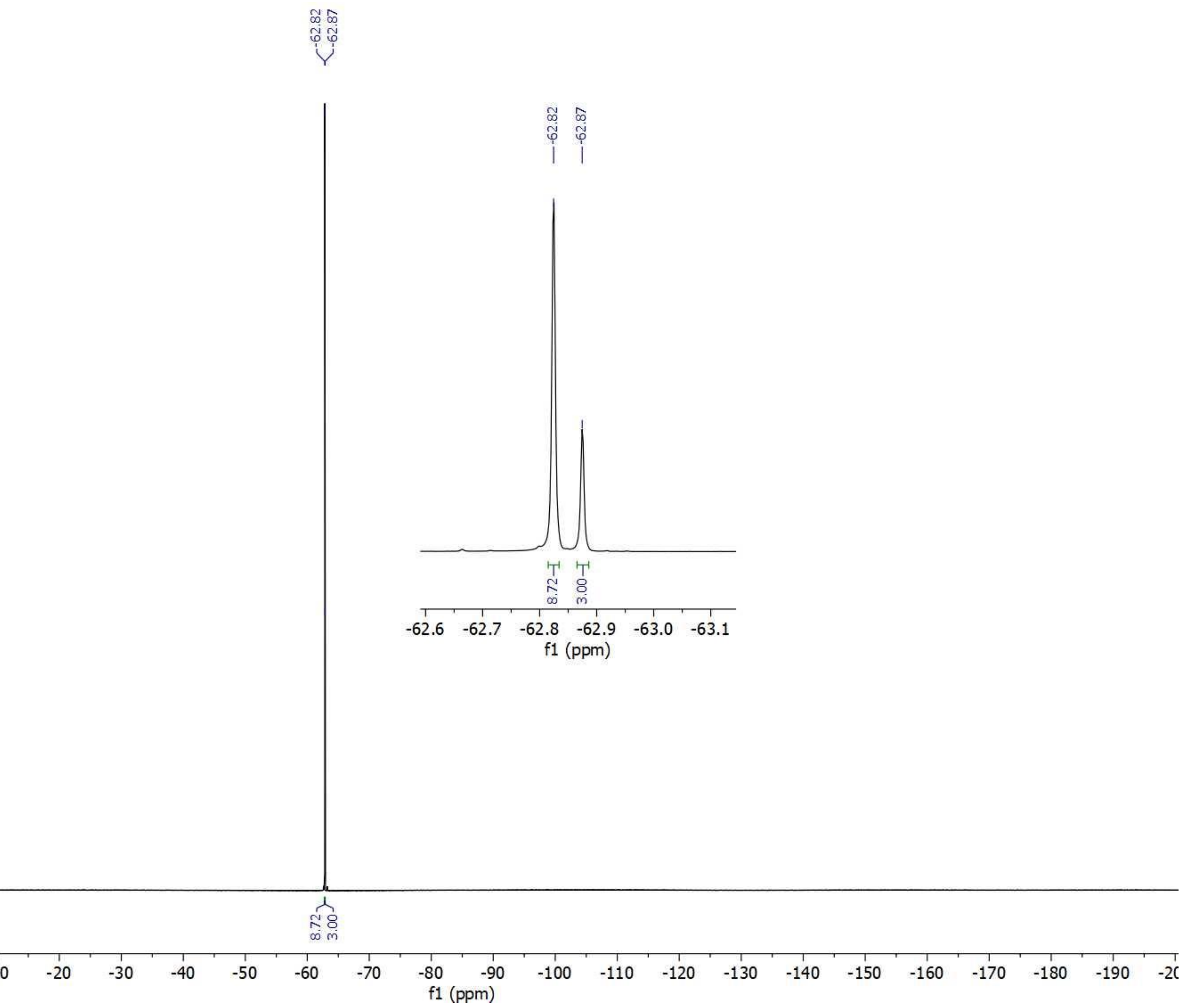

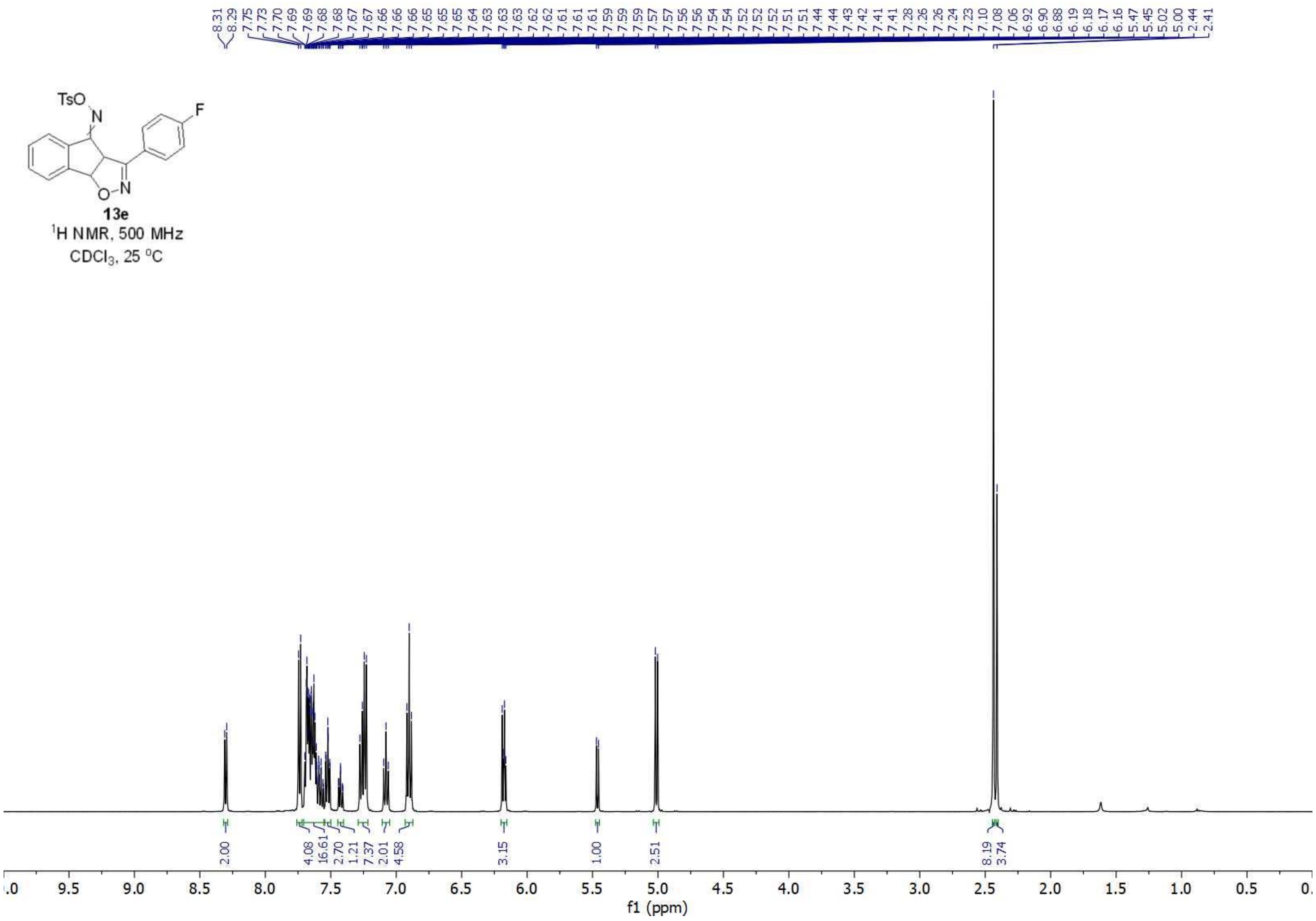
TsO
N
F
O
N
13e
^{1}H NMR, 500 MHz
$CDCl_3$, 25 °C
f1 (ppm)

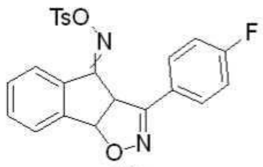

13e

^{13}C NMR, 125 MHz

$CDCl_3$, 25 °C

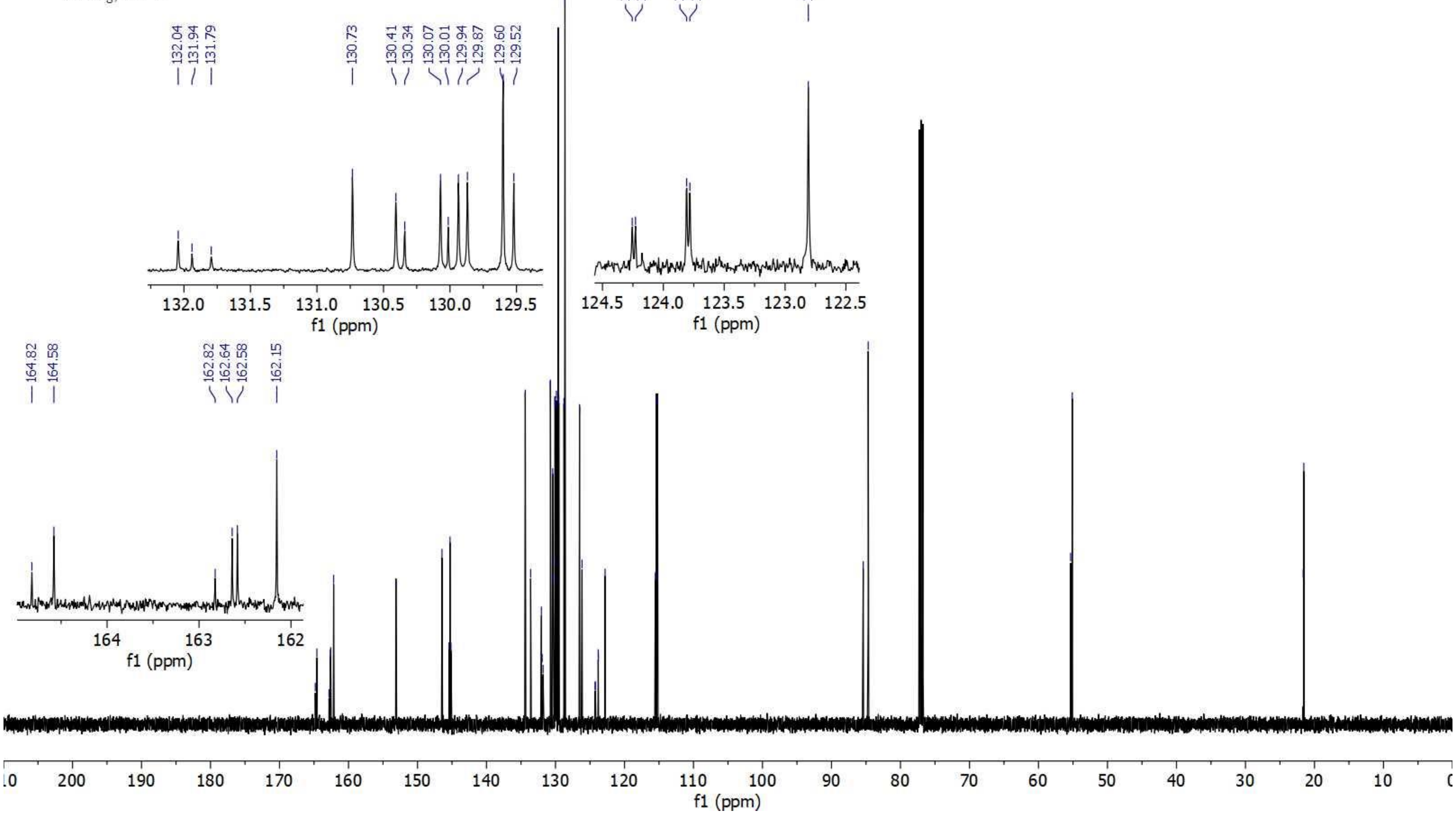

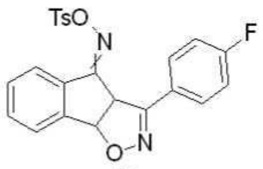

13e
^{19}F NMR, 470 MHz
$CDCl_3$, 25 °C

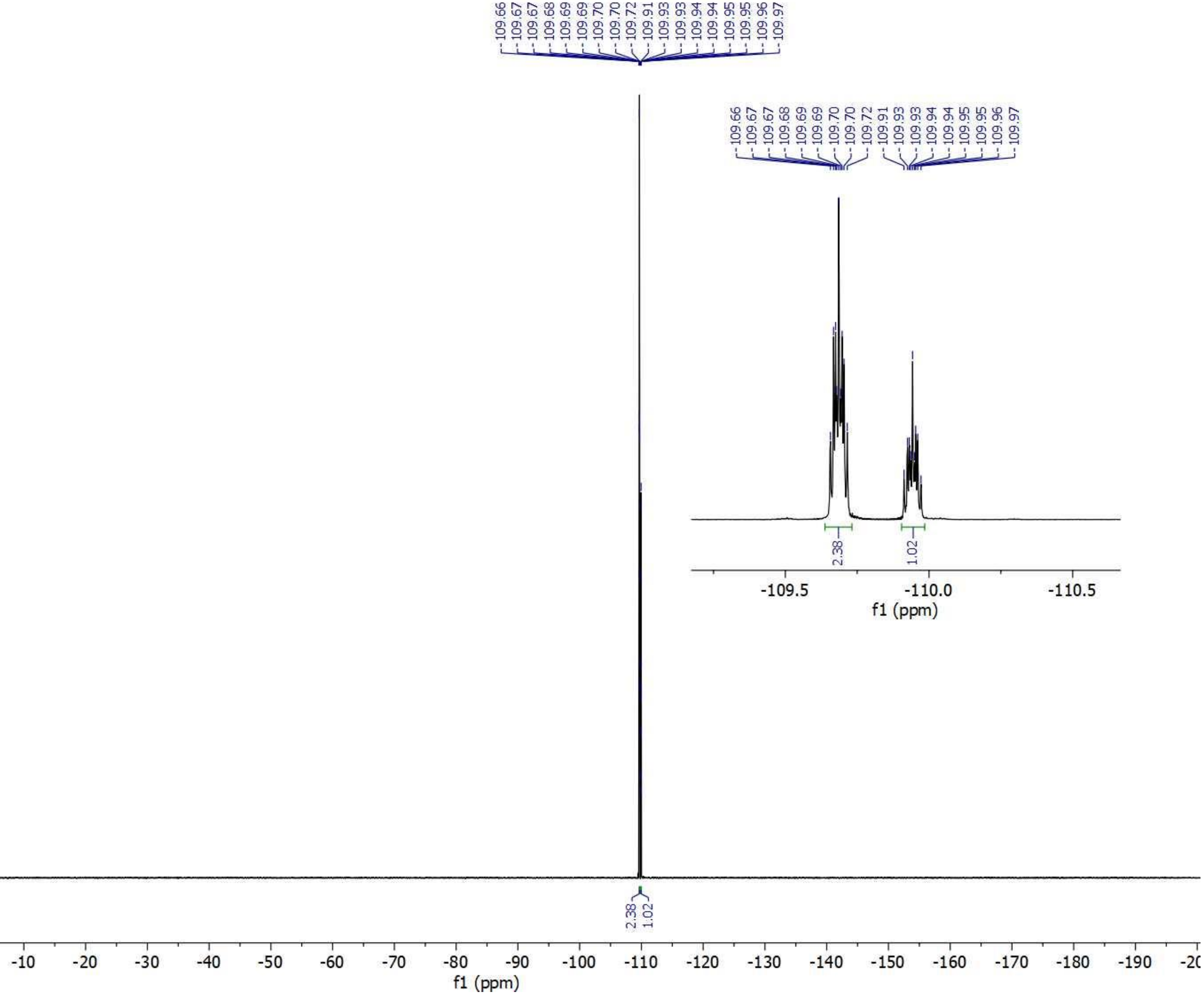

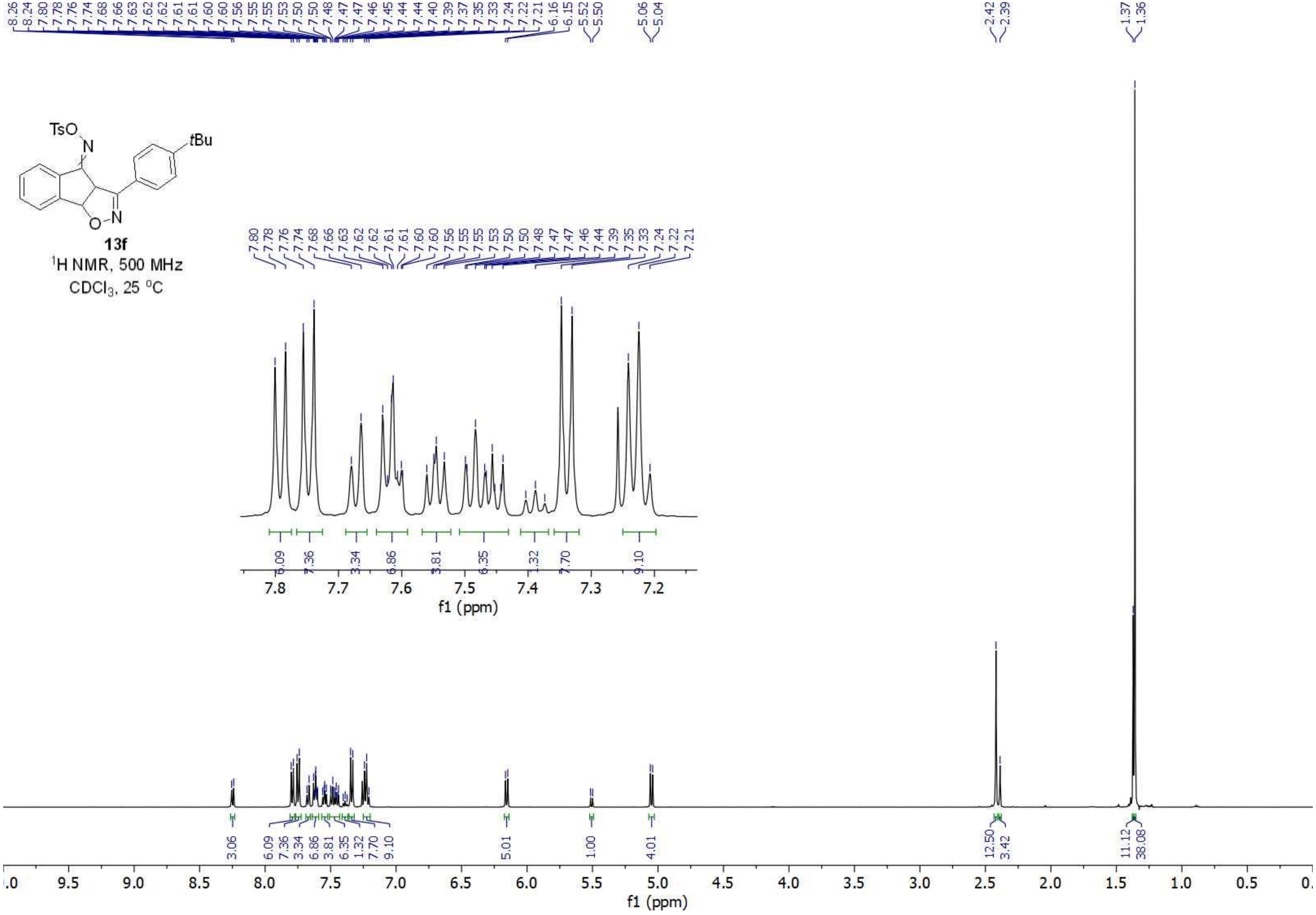

13f
^{1}H NMR, 500 MHz
$CDCl_3$, 25 °C

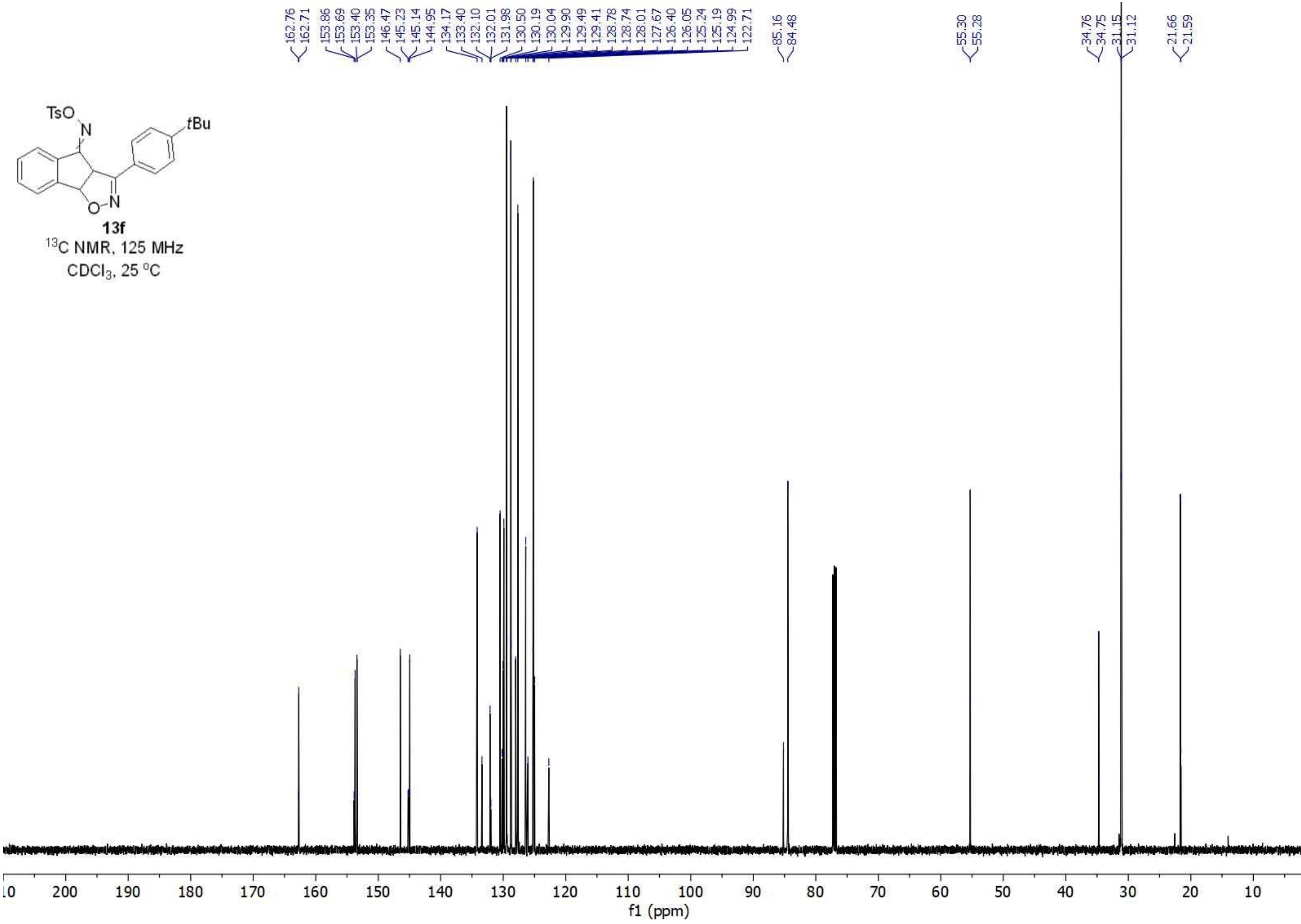

13f
^{13}C NMR, 125 MHz
$CDCl_3$, 25 °C

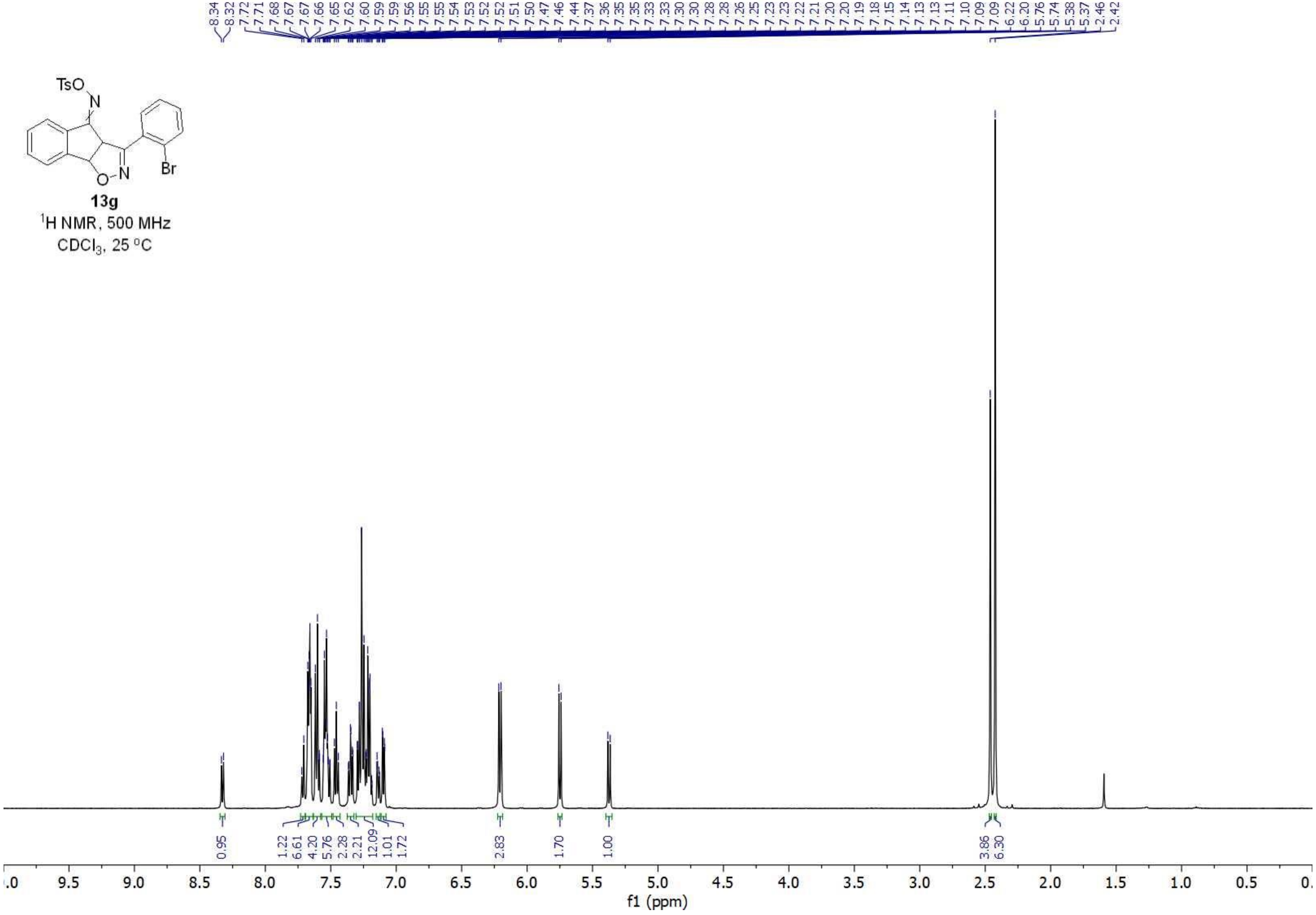
TsO
N
N
O
Br
13g
^{1}H NMR, 500 MHz
$CDCl_3$, 25 °C
f1 (ppm)

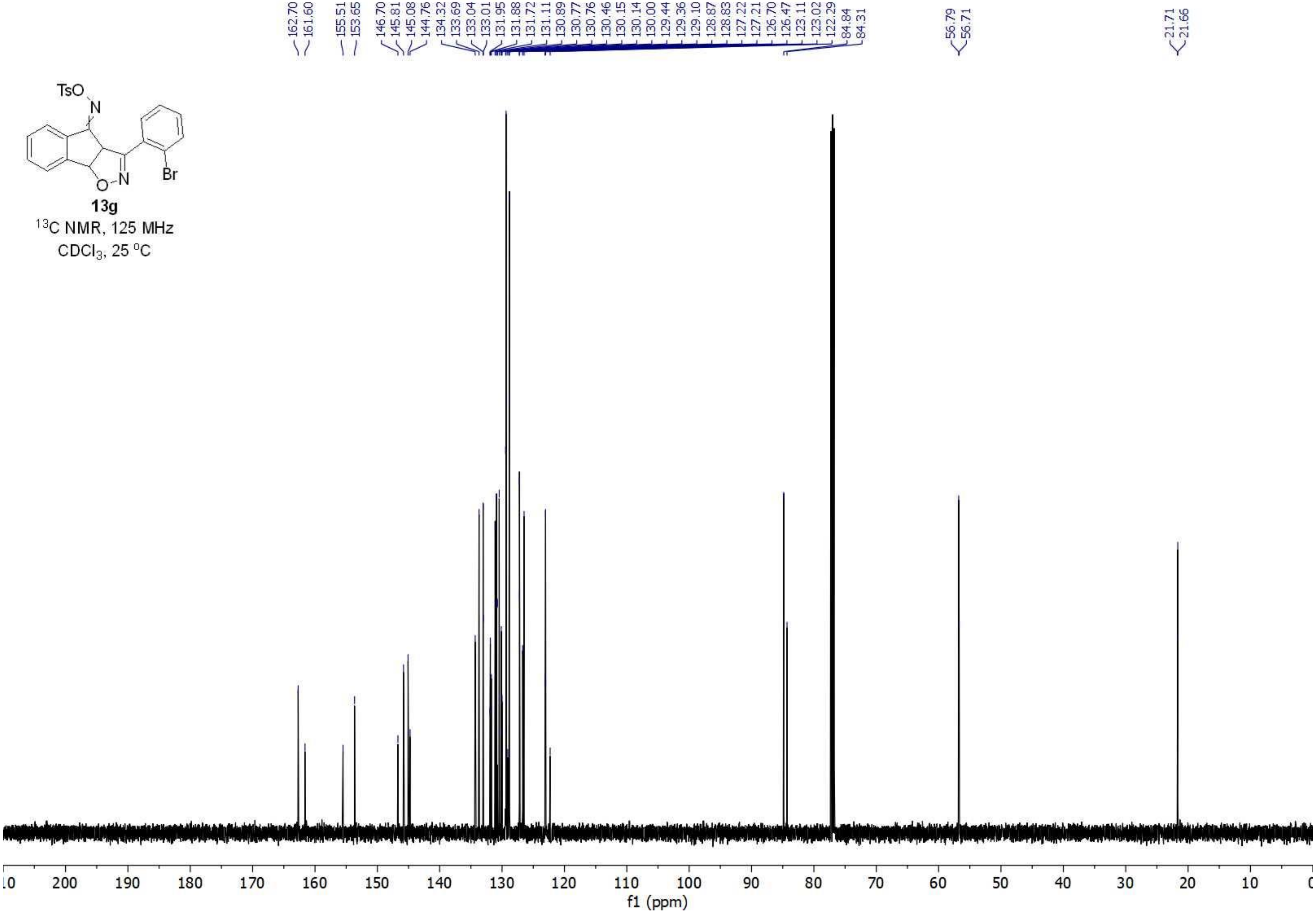

162.70
161.60
155.51
153.65
146.70
145.81
145.08
144.76
134.32
133.69
133.04
133.01
131.95
131.88
131.72
131.11
130.89
130.77
130.76
130.46
130.15
130.14
130.00
129.44
129.36
129.10
128.87
128.83
127.22
127.21
126.70
126.47
123.11
123.02
122.29
84.84
84.31
56.79
56.71
21.71
21.66
TsO
N
O
N
Br
13g
^{13}C NMR, 125 MHz
$CDCl_3$, 25 ^oC
f1 (ppm)

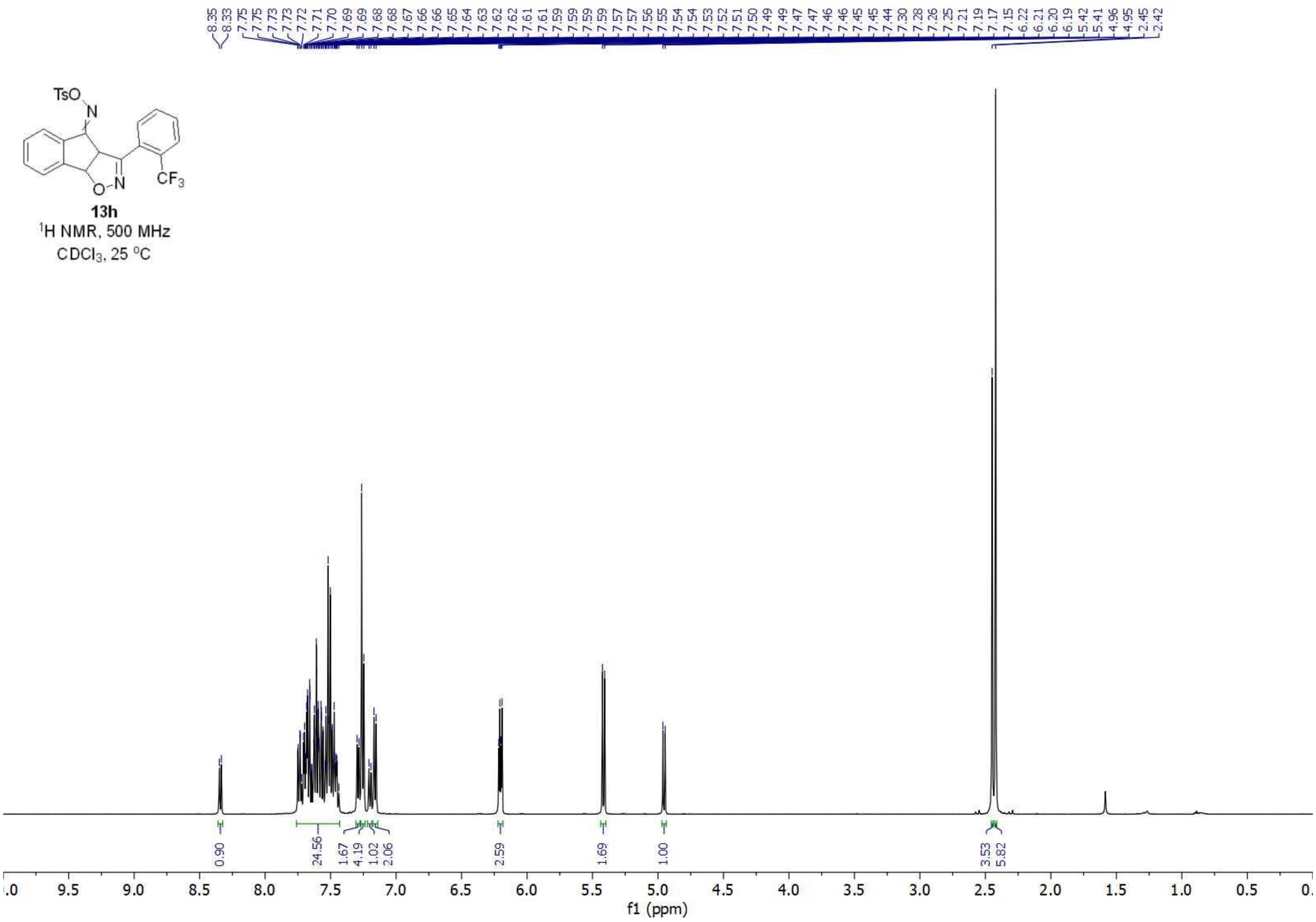
TsO
N
CF3
O-N
13h
1H NMR, 500 MHz
CDCl3, 25 °C
0.90
24.56
1.67
4.19
1.02
2.06
2.59
1.69
1.00
3.53
5.82
f1 (ppm)

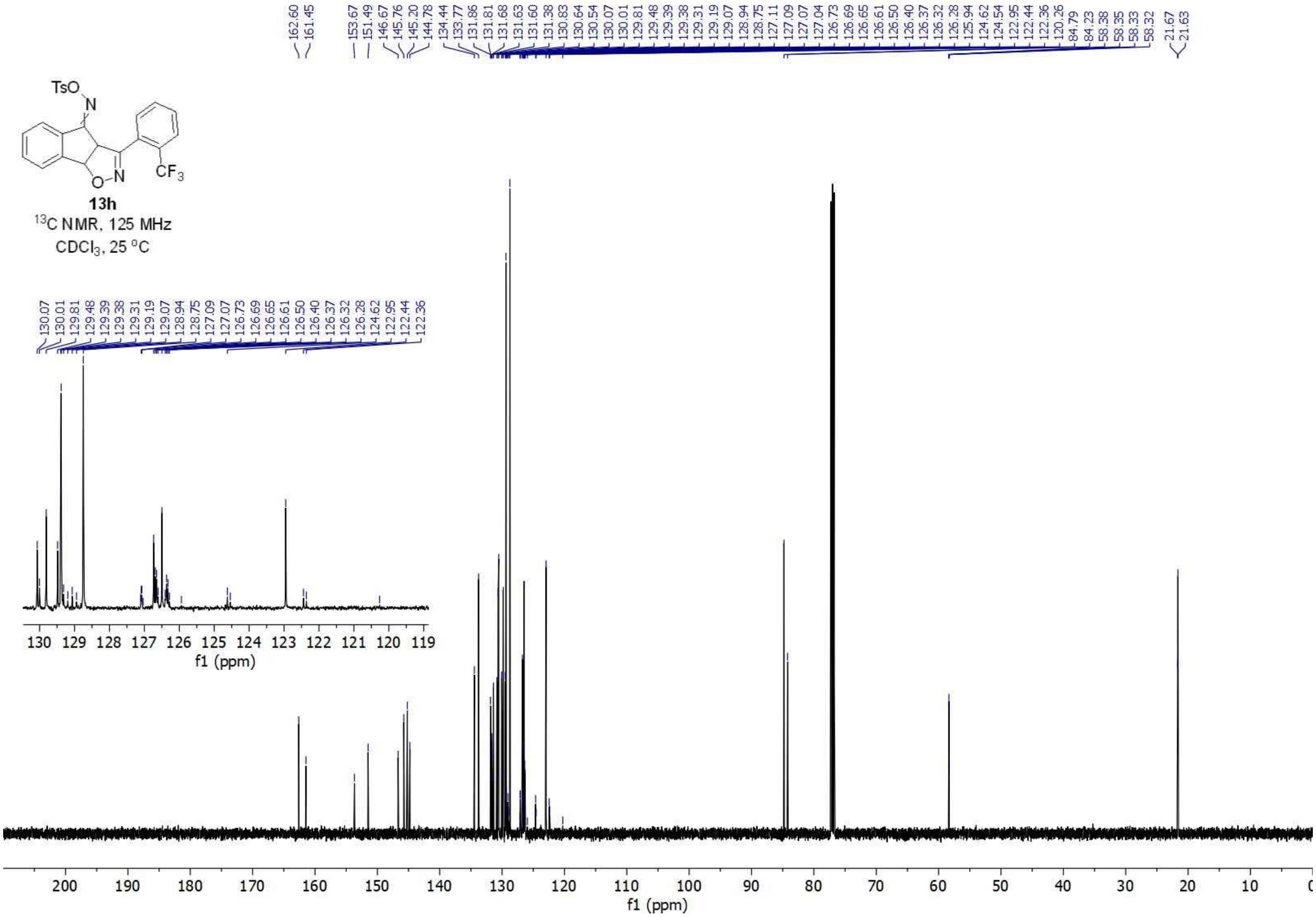

13h

^{13}C NMR, 125 MHz

$CDCl_3$, 25 °C

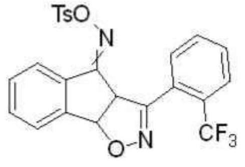

13h

^{19}F NMR, 470 MHz

$CDCl_3$, 25 °C

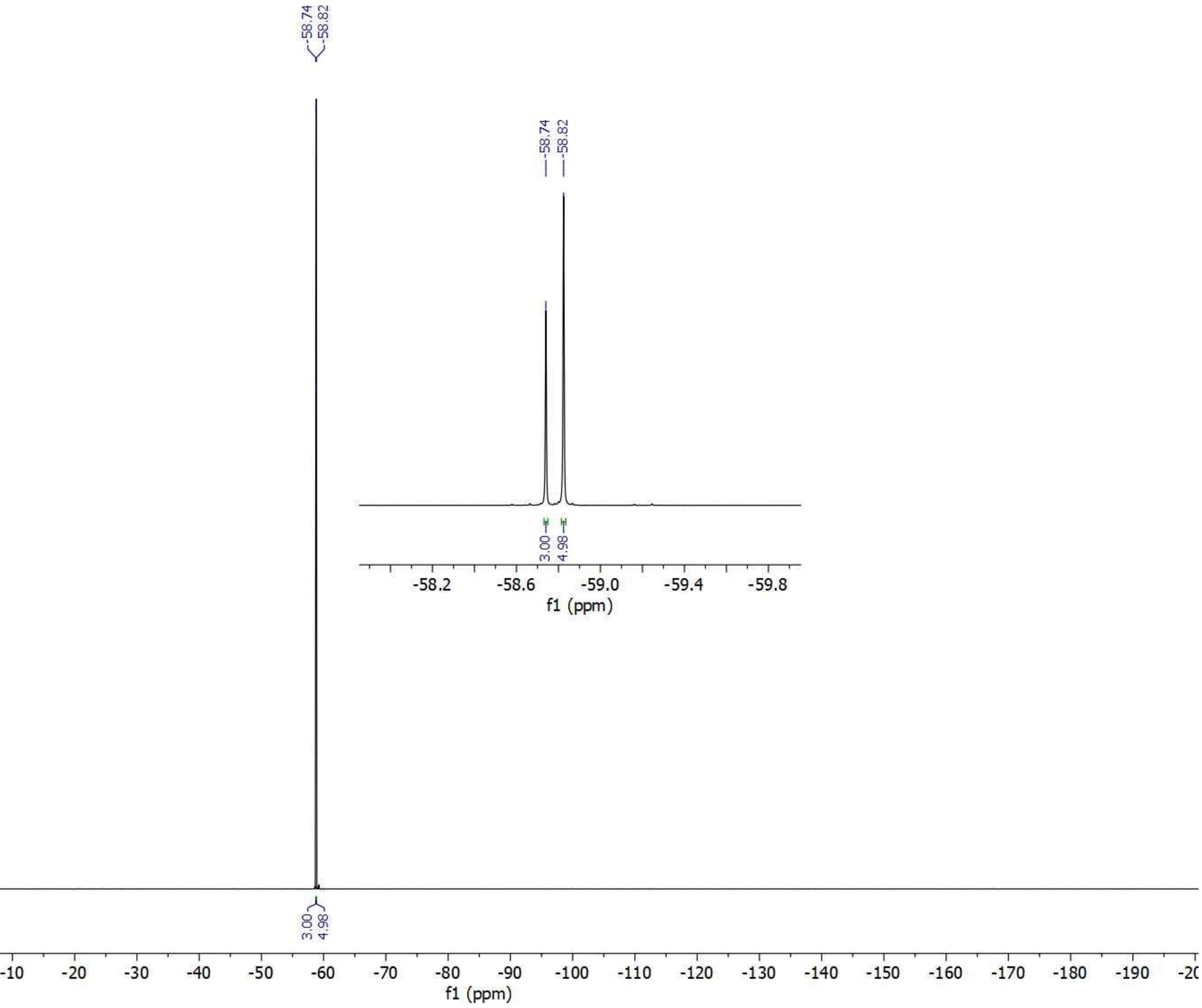

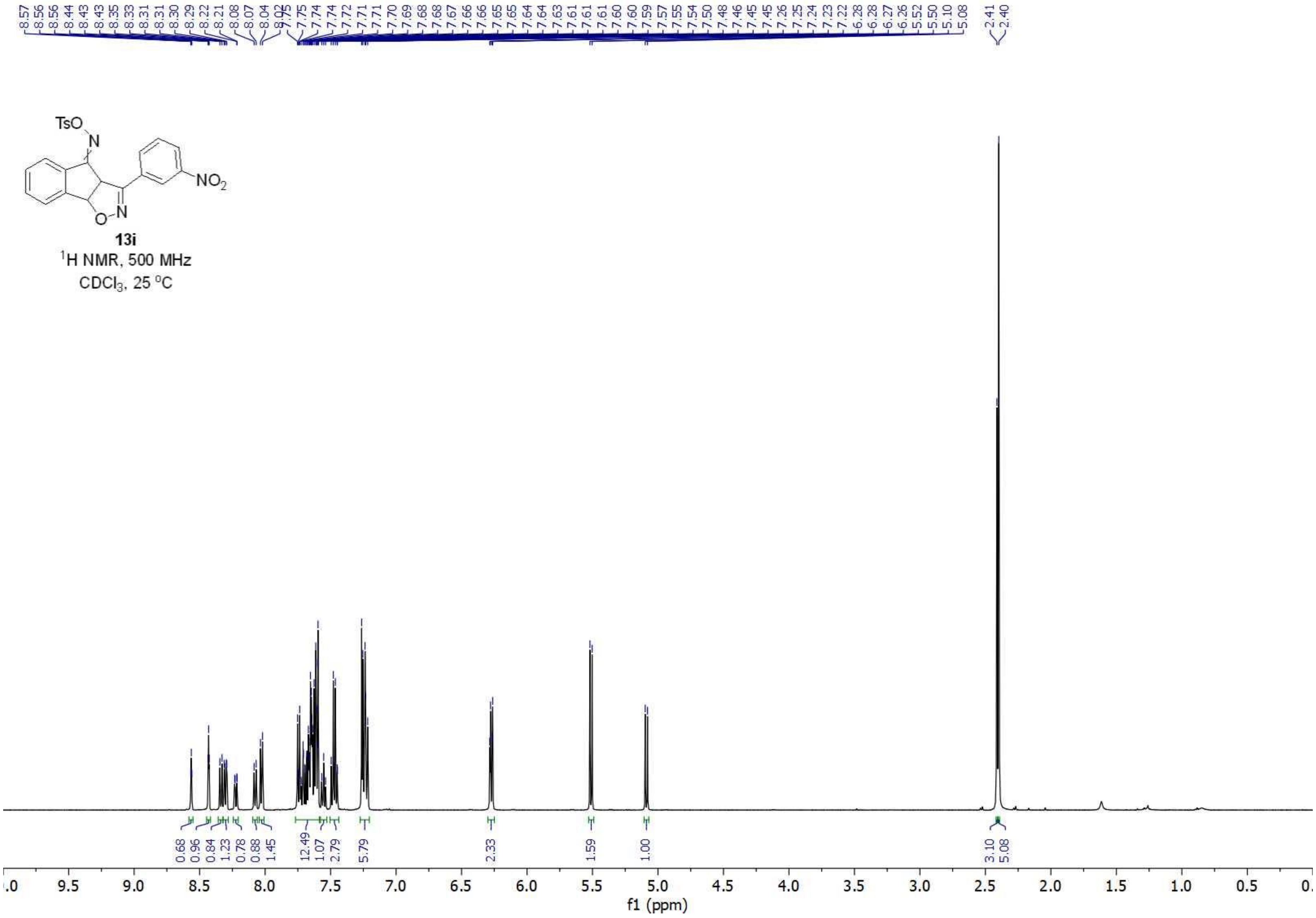
8.57 8.56 8.44 8.43 8.43 8.35 8.33 8.31 8.31 8.30 8.29 8.22 8.21 8.08 8.07 8.04 8.02 7.75 7.75 7.74 7.74 7.72 7.71 7.71 7.70 7.69 7.68 7.67 7.66 7.65 7.65 7.64 7.64 7.63 7.61 7.61 7.61 7.60 7.60 7.59 7.57 7.55 7.54 7.50 7.48 7.46 7.45 7.45 7.26 7.25 7.24 7.23 7.22 6.28 6.28 6.27 6.26 5.52 5.50 5.10 5.08 2.41 2.40
TsO
N
NO2
O
N
13i
1H NMR, 500 MHz
CDCl3, 25 °C
0.68 0.96 0.84 1.23 0.78 0.88 1.45 12.49 1.07 2.79 5.79 2.33 1.59 1.00 3.10 5.08
9.5 9.0 8.5 8.0 7.5 7.0 6.5 6.0 5.5 5.0 4.5 4.0 3.5 3.0 2.5 2.0 1.5 1.0 0.5
f1 (ppm)

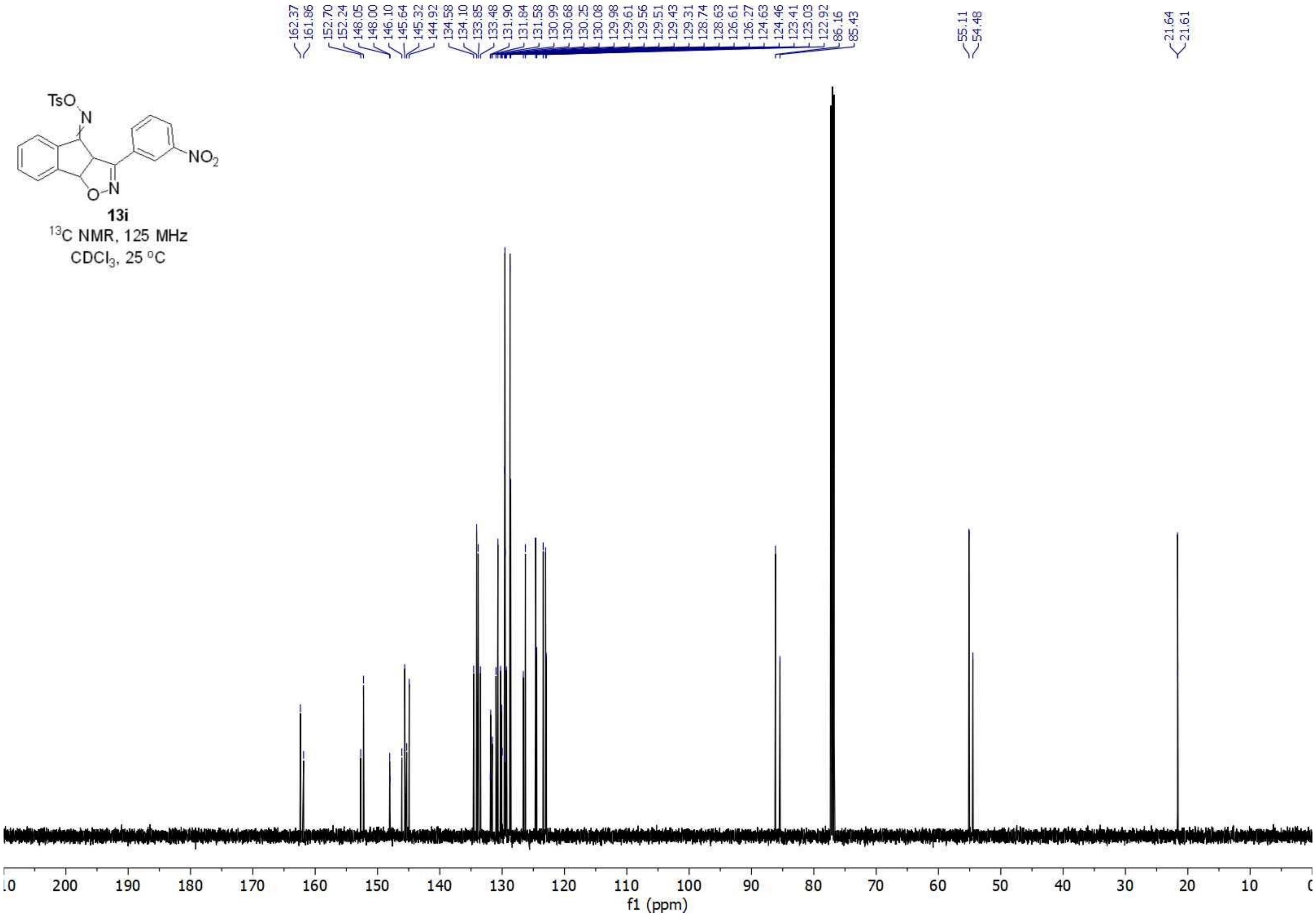
TsO
N
NO2
O
N
13i
13C NMR, 125 MHz
CDCl3, 25 °C
162.37
161.86
152.70
152.24
148.05
148.00
146.10
145.64
145.32
144.92
134.58
134.10
133.85
133.48
131.90
131.84
131.58
130.99
130.68
130.25
130.08
129.98
129.61
129.56
129.51
129.43
129.31
128.74
128.63
126.61
126.27
124.63
124.46
123.41
123.03
122.92
86.16
85.43
55.11
54.48
21.64
21.61
f1 (ppm)

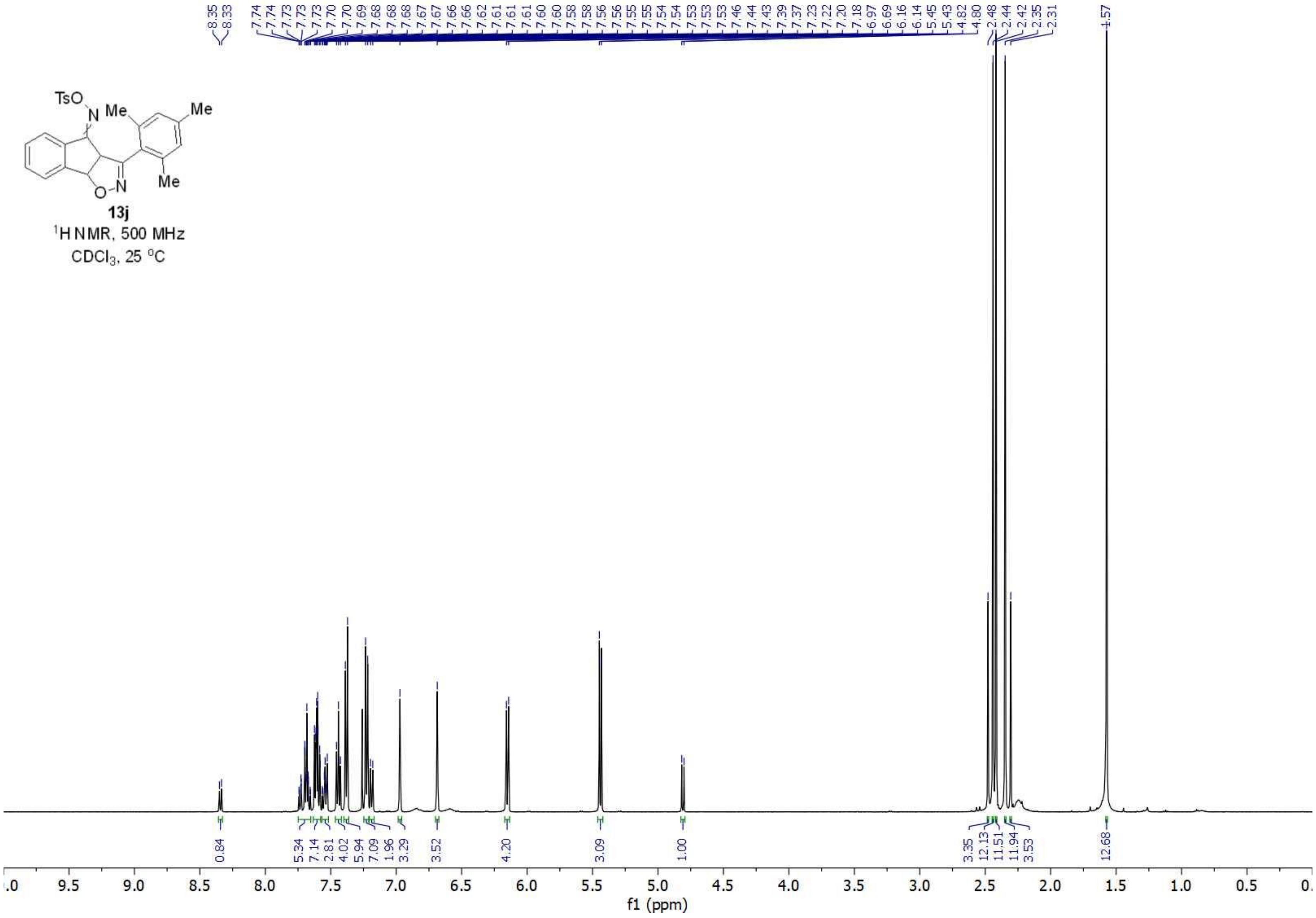
TsO
N
Me
Me
Me
O
N
13j
¹H NMR, 500 MHz
CDCl3, 25 °C
f1 (ppm)

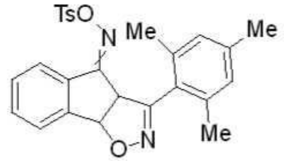

13j

^{13}C NMR, 125 MHz

$CDCl_3$, 25 °C

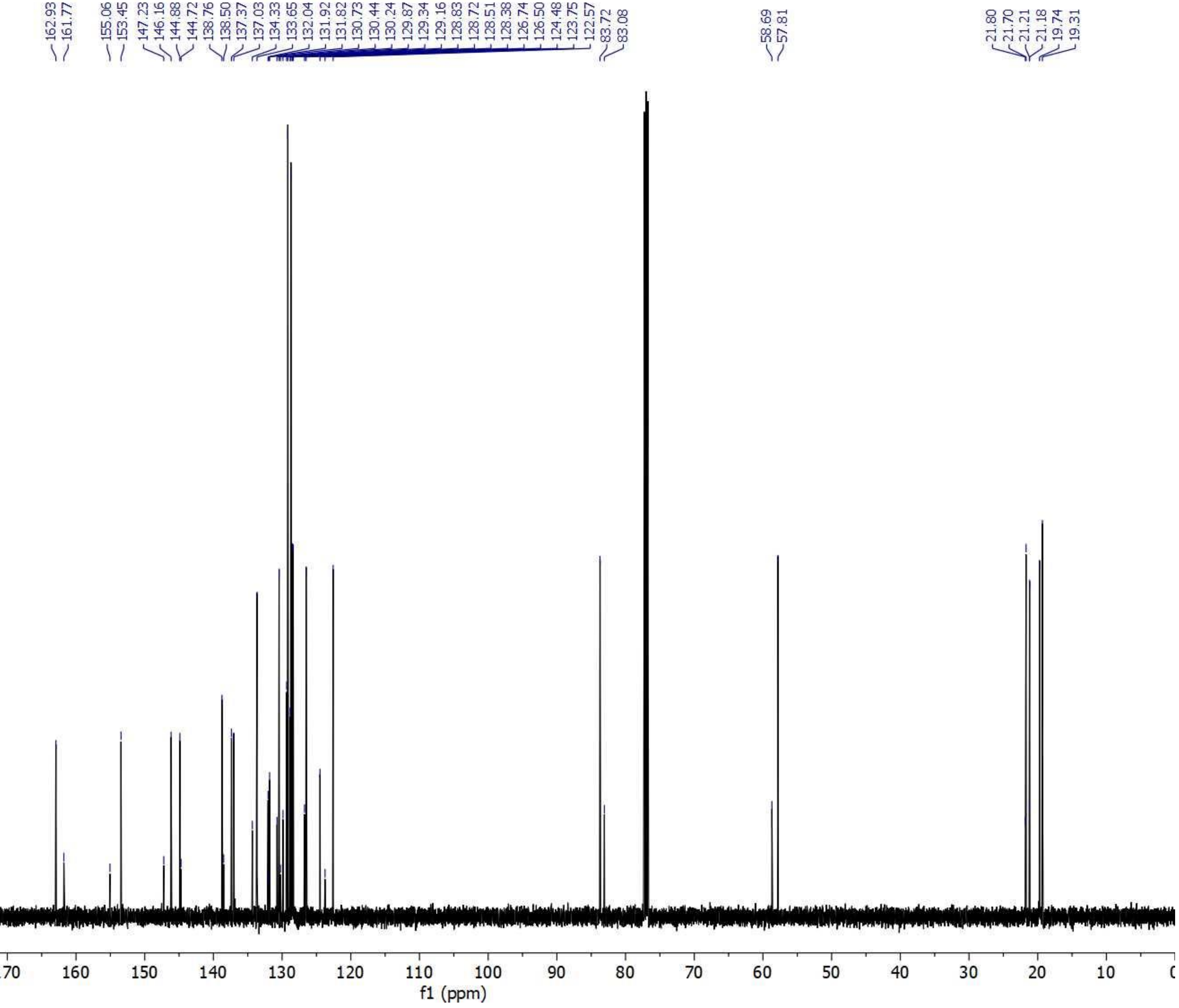

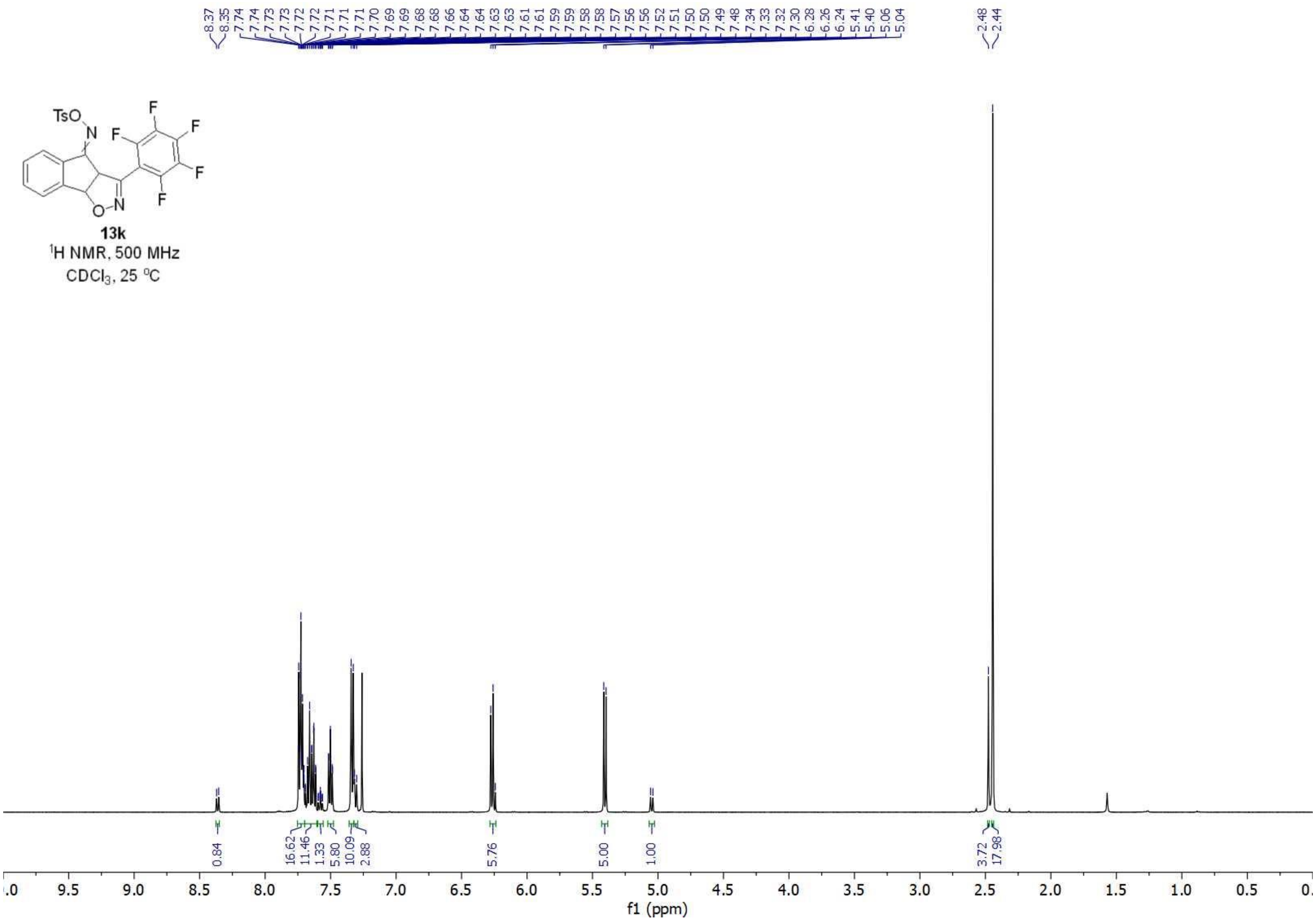
13k
¹H NMR, 500 MHz
CDCl3, 25 °C
f1 (ppm)

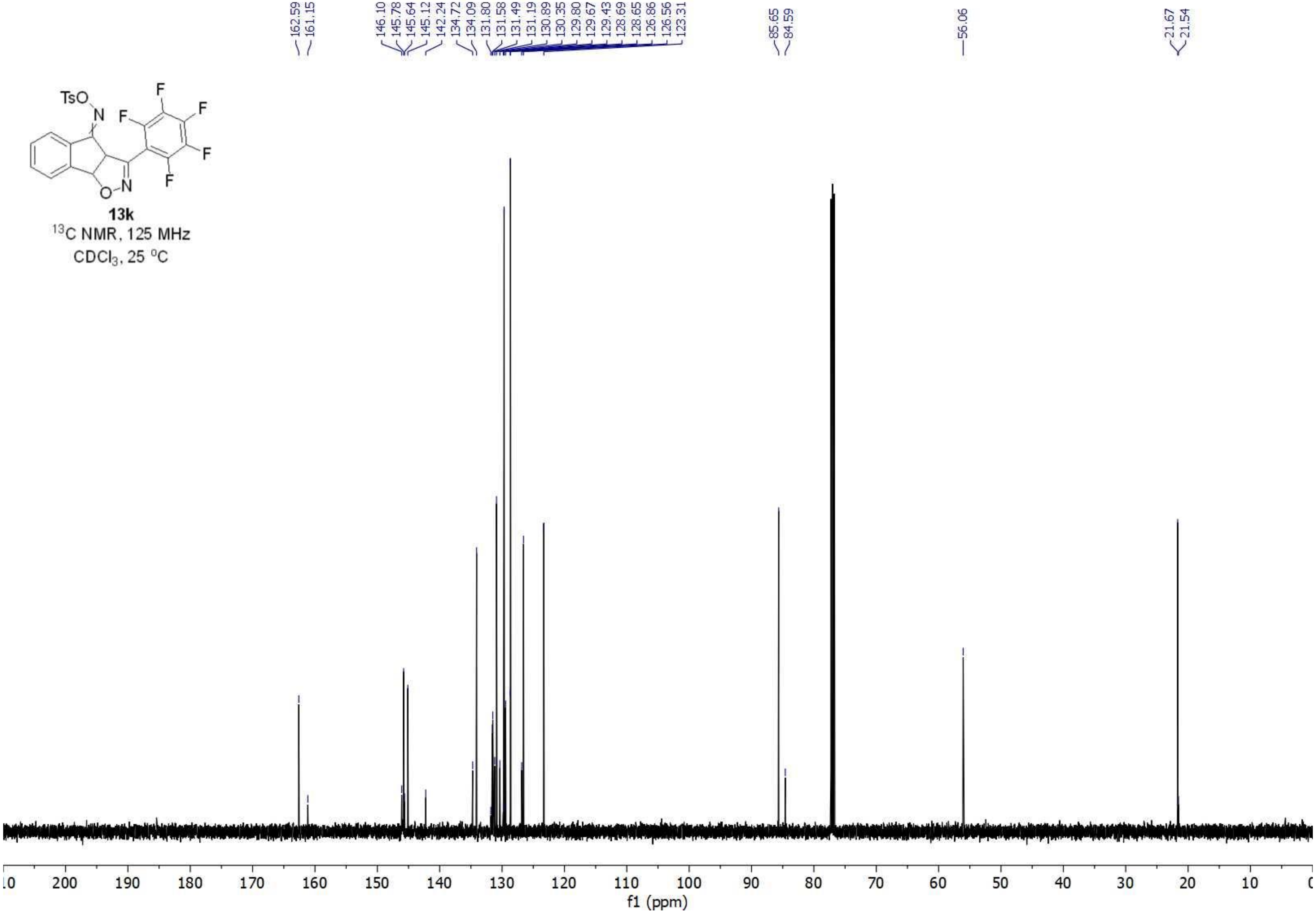
162.59
161.15
146.10
145.78
145.64
145.12
142.24
134.72
134.09
131.80
131.58
131.49
131.19
130.89
130.35
129.80
129.67
129.43
128.69
128.65
126.86
126.56
123.31
85.65
84.59
56.06
21.67
21.54
TsO
N
F
F
F
F
F
O
N
13k
^{13}C NMR, 125 MHz
$CDCl_3$, 25 °C
200
190
180
170
160
150
140
130
120
110
100
90
80
70
60
50
40
30
20
10
f1 (ppm)

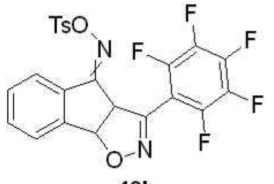

13k

^{19}F NMR, 470 MHz

$CDCl_3$, 25 °C

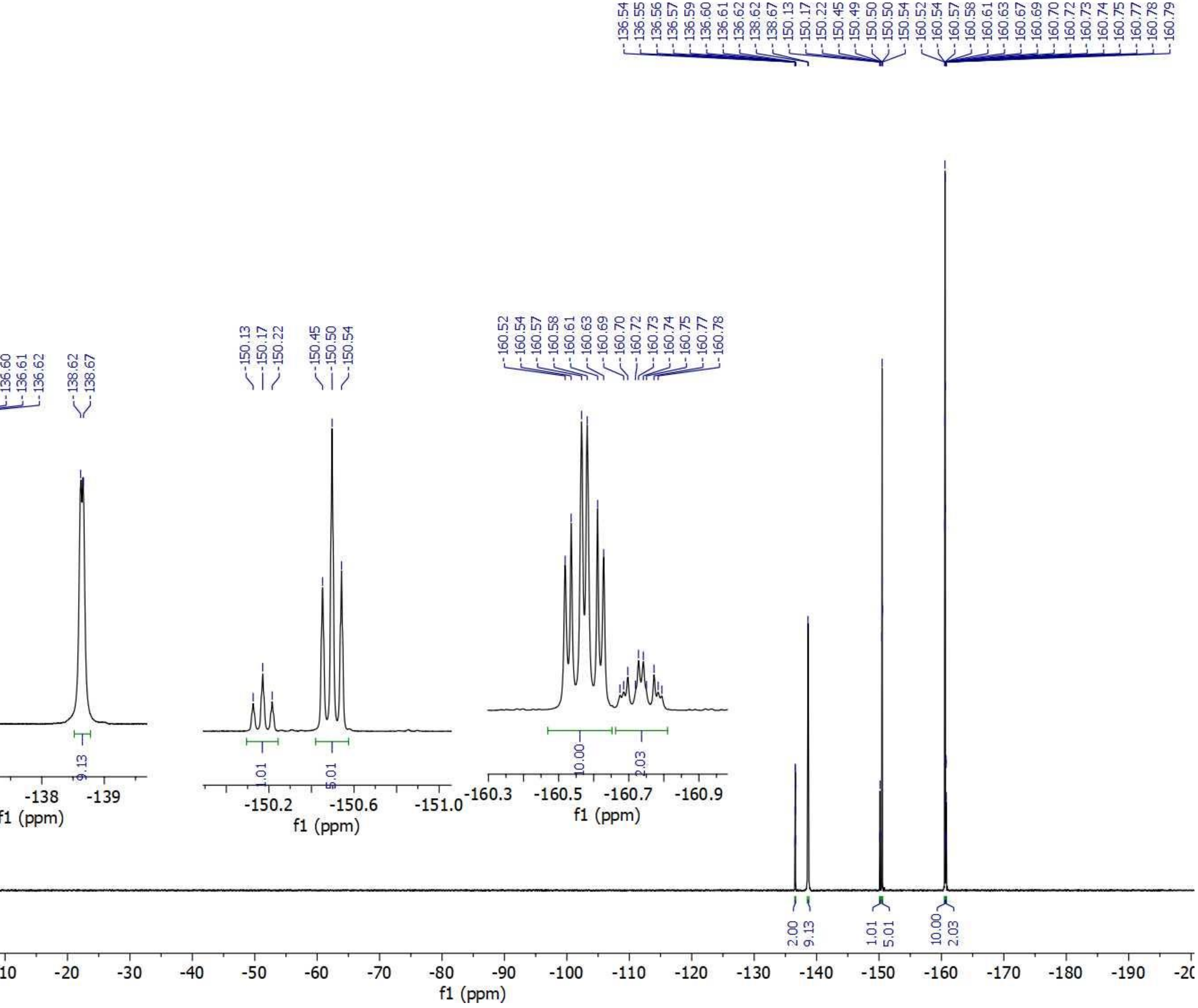

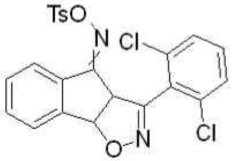

13l

^{1}H NMR, 500 MHz

$CDCl_3$, 25 °C

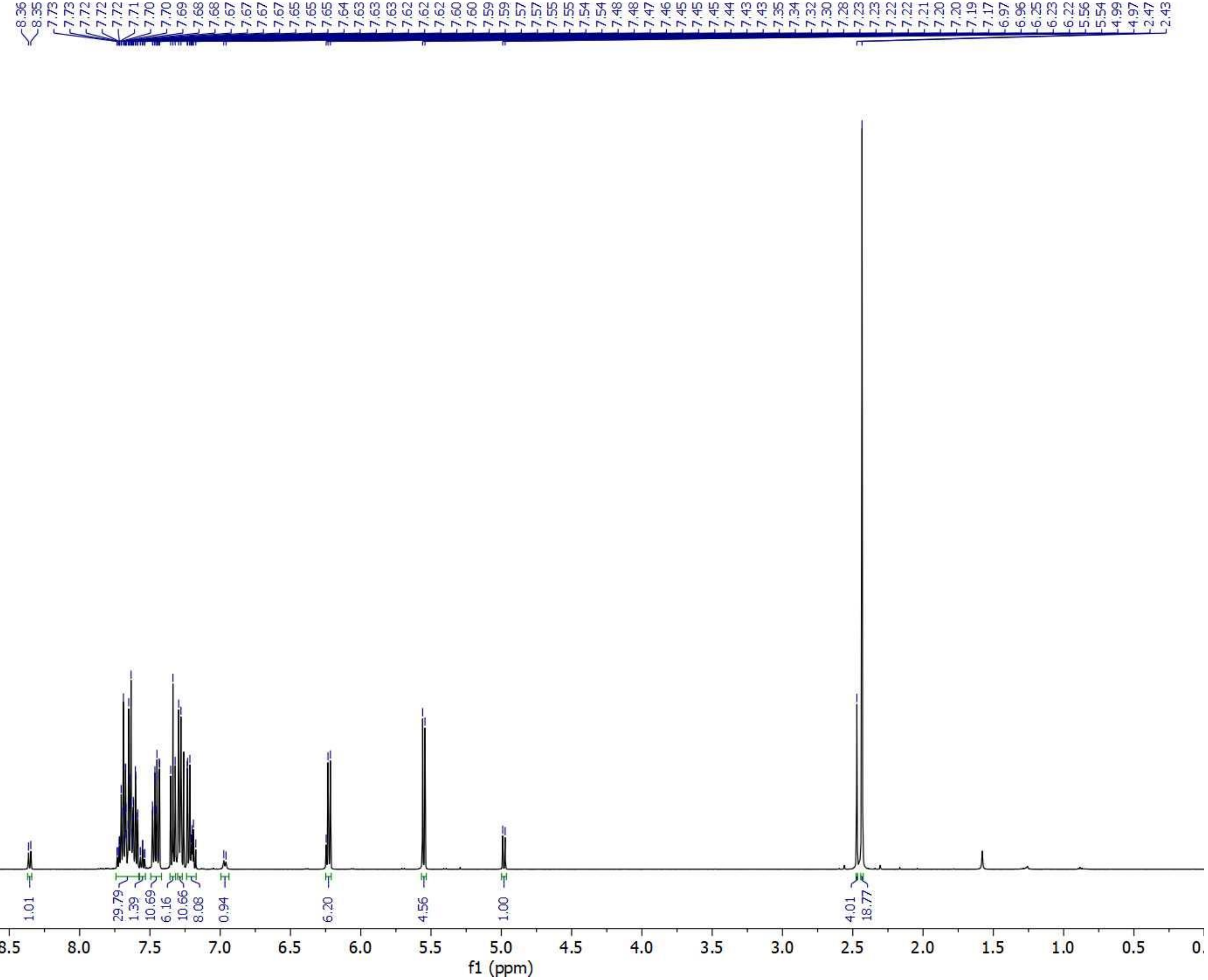

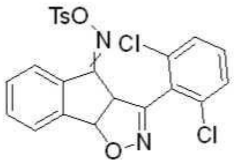

13l

^{13}C NMR, 125 MHz

$CDCl_3$, 25 °C

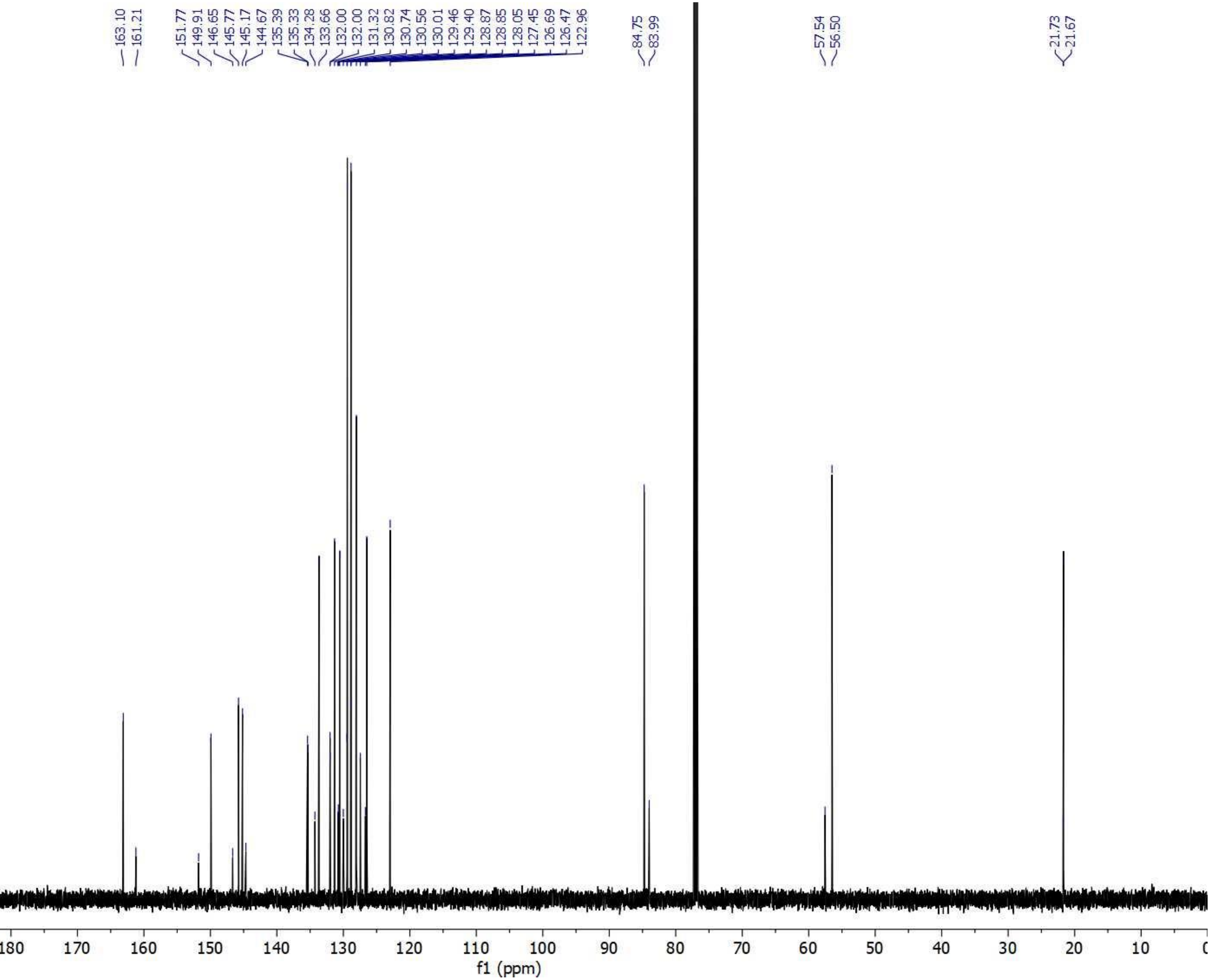

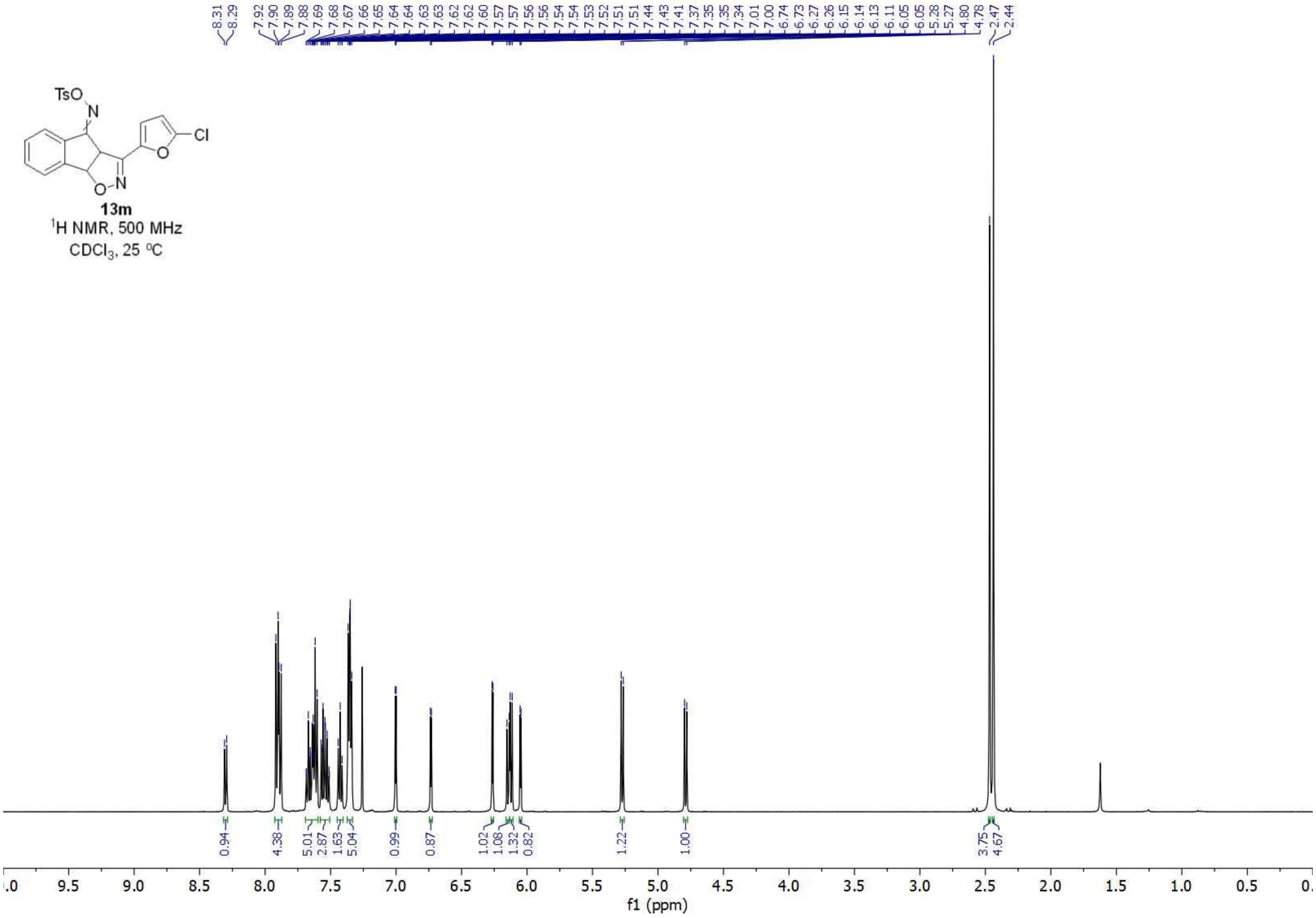
TsO
N
Cl
O
O
N
13m
¹H NMR, 500 MHz
CDCl3, 25 °C
f1 (ppm)

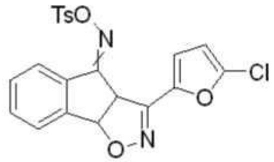

13m

^{13}C NMR, 125 MHz

$CDCl_3$, 25 °C

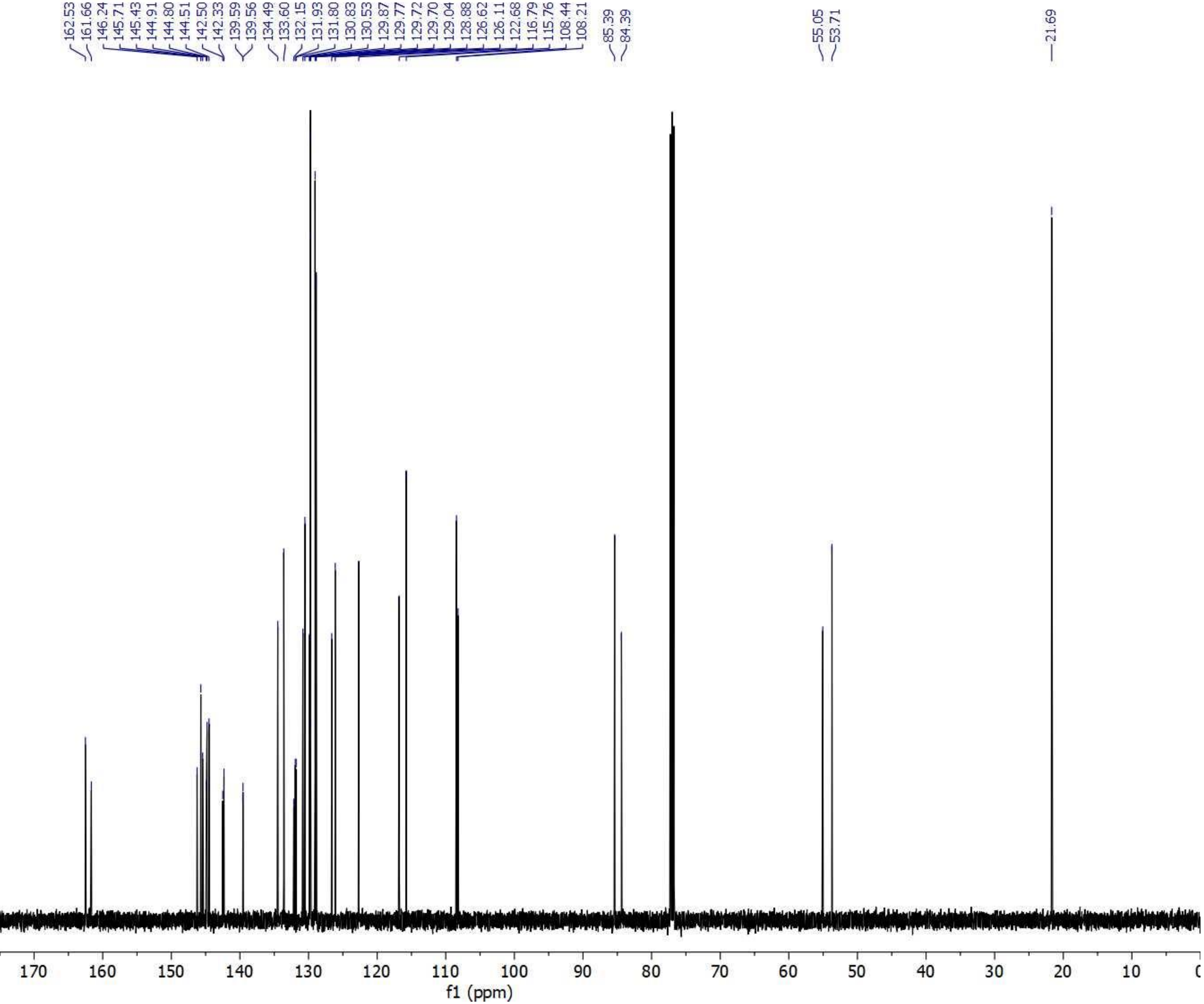

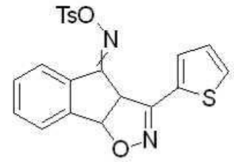

13n
^{1}H NMR, 500 MHz
$CDCl_3$, 25 °C

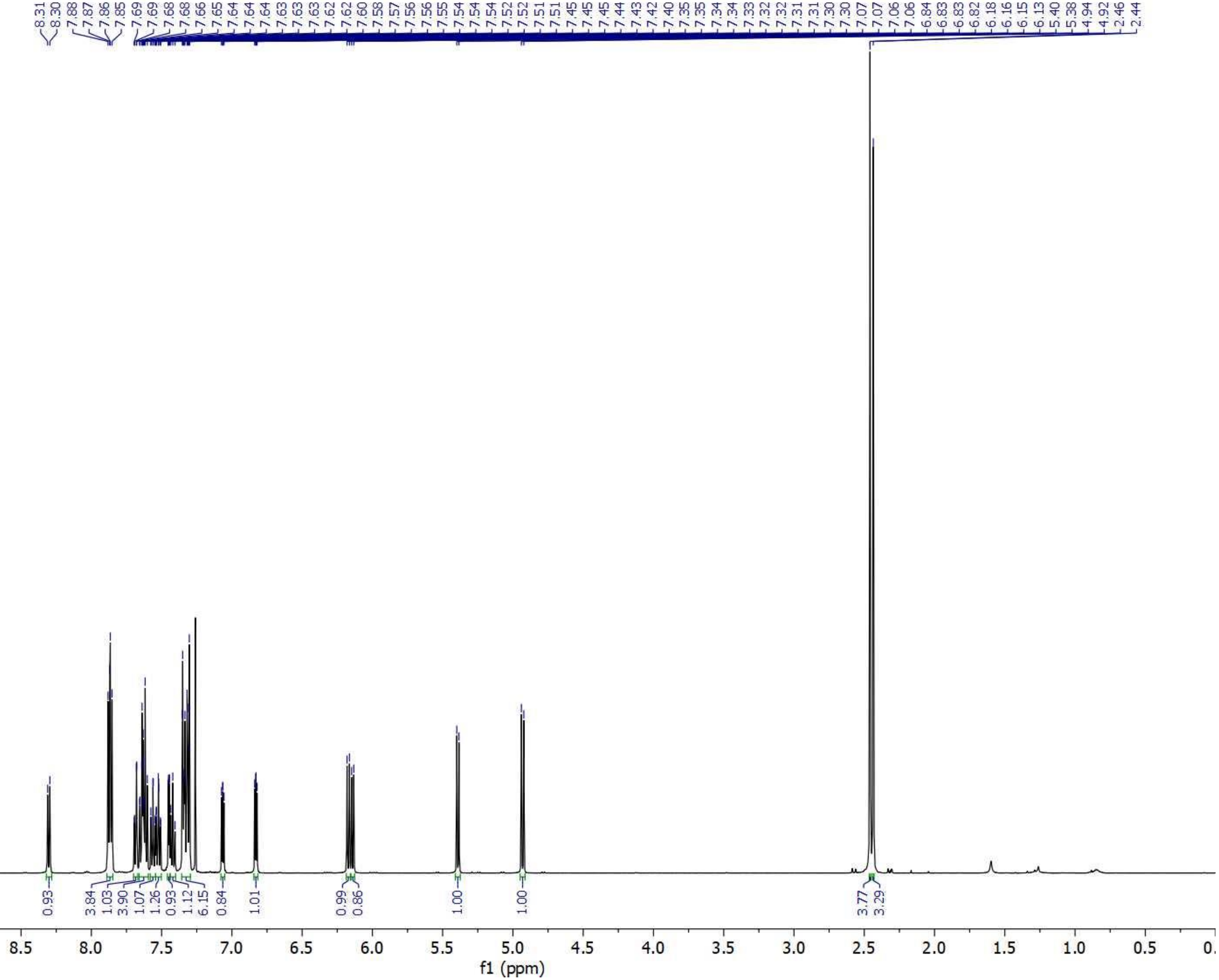

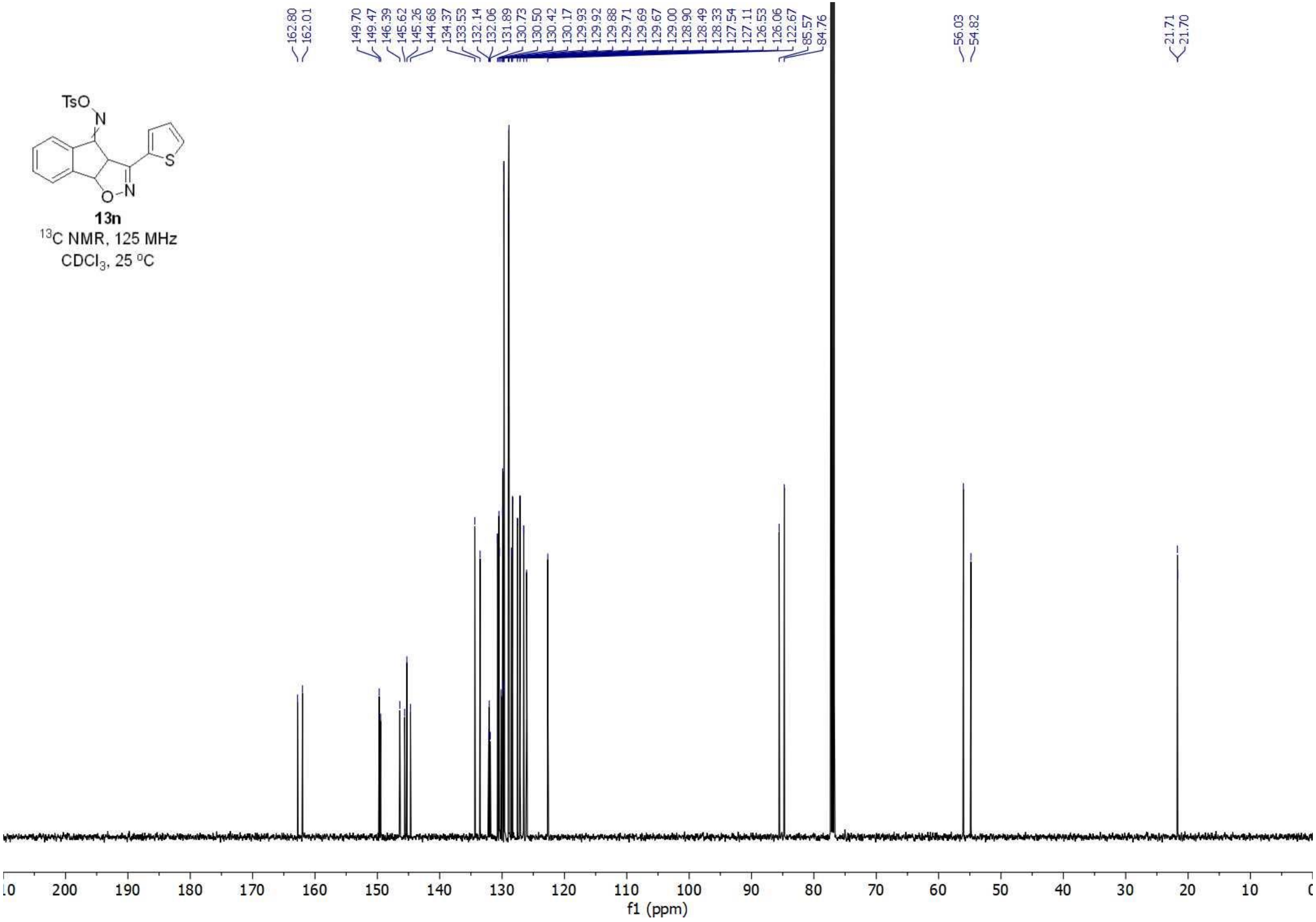

13n

^{13}C NMR, 125 MHz

$CDCl_3$, 25 °C

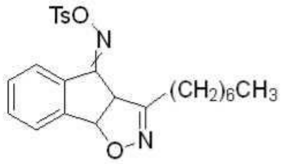

13o

^{1}H NMR, 500 MHz

$CDCl_3$, 25 °C

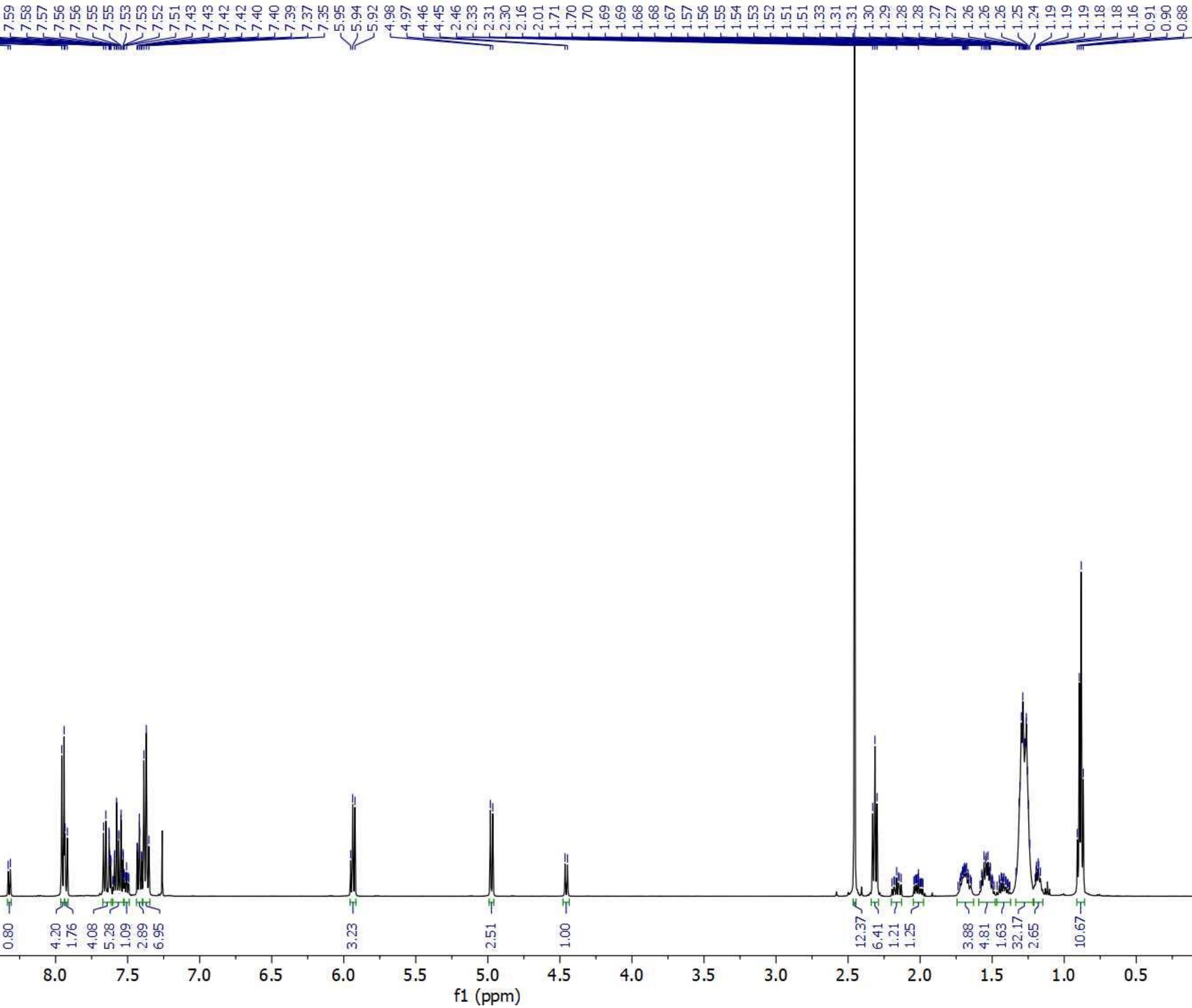

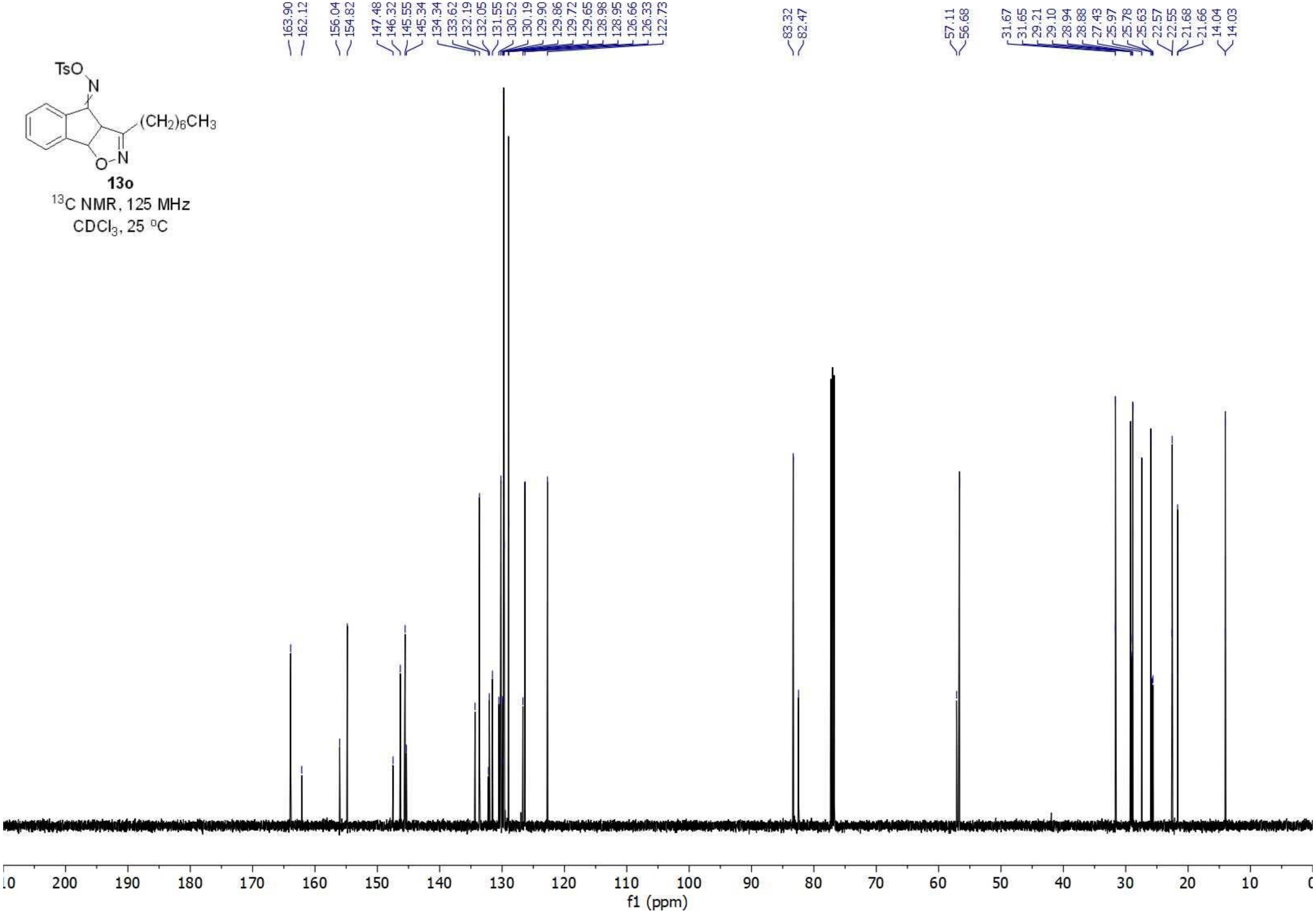
TsO
N
(CH2)6CH3
O
N
13o
13C NMR, 125 MHz
CDCl3, 25 °C
163.90
162.12
156.04
154.82
147.48
146.32
145.55
145.34
134.34
133.62
132.19
132.05
131.55
130.52
130.19
129.90
129.86
129.72
129.65
128.98
128.95
126.66
126.33
122.73
83.32
82.47
57.11
56.68
31.67
31.65
29.21
29.10
28.94
28.88
27.43
25.97
25.78
25.63
22.57
22.55
21.68
21.66
14.04
14.03
f1 (ppm)

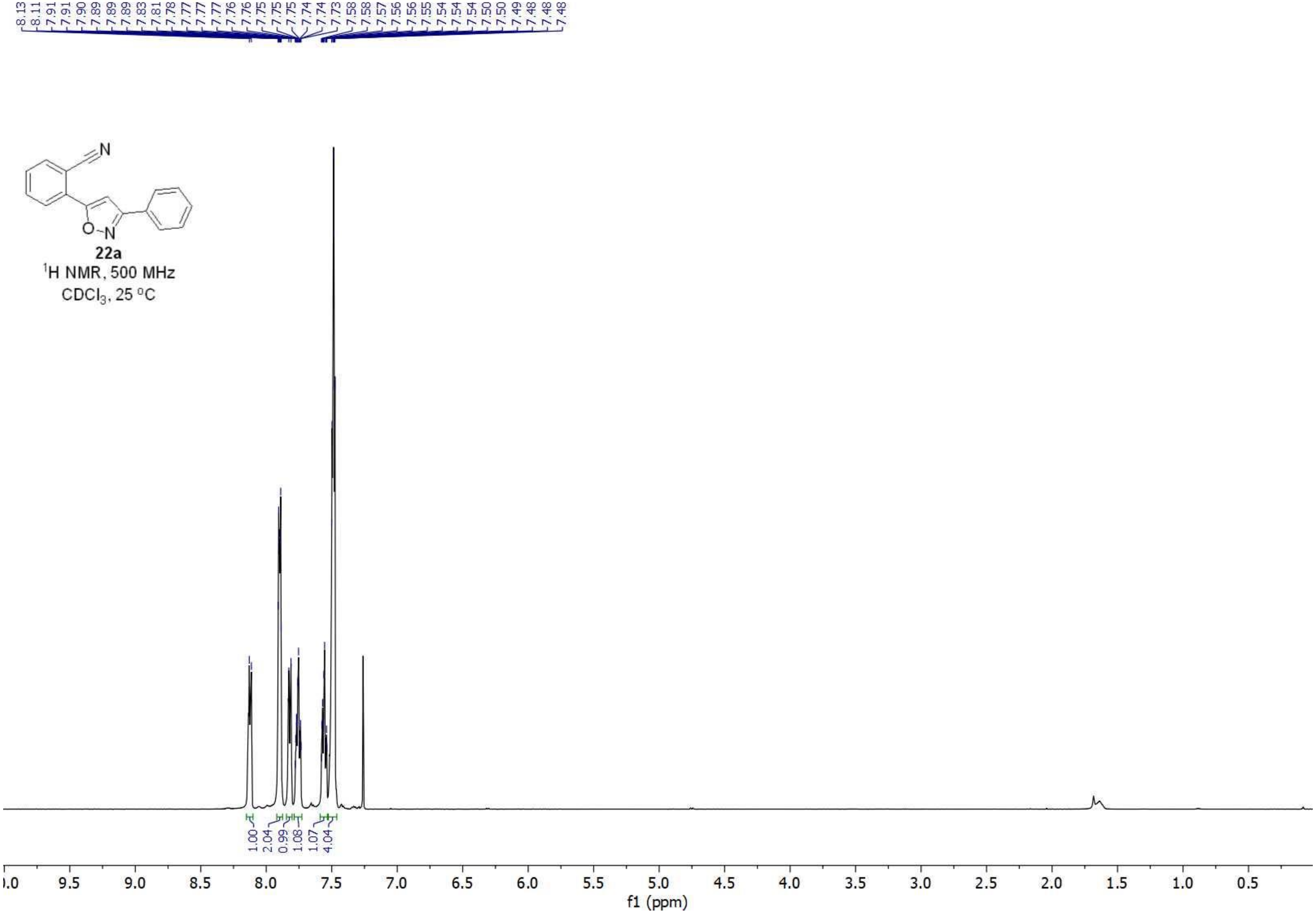
8.13
8.11
7.91
7.91
7.90
7.89
7.89
7.89
7.83
7.81
7.78
7.77
7.77
7.77
7.76
7.76
7.75
7.75
7.75
7.74
7.74
7.73
7.58
7.58
7.57
7.56
7.56
7.55
7.54
7.54
7.54
7.50
7.50
7.49
7.48
7.48
7.48
N
O
N
22a
^{1}H NMR, 500 MHz
$CDCl_3$, 25 °C
1.00
2.04
0.99
1.08
1.07
4.04
9.5
9.0
8.5
8.0
7.5
7.0
6.5
6.0
5.5
5.0
4.5
4.0
3.5
3.0
2.5
2.0
1.5
1.0
0.5
f1 (ppm)

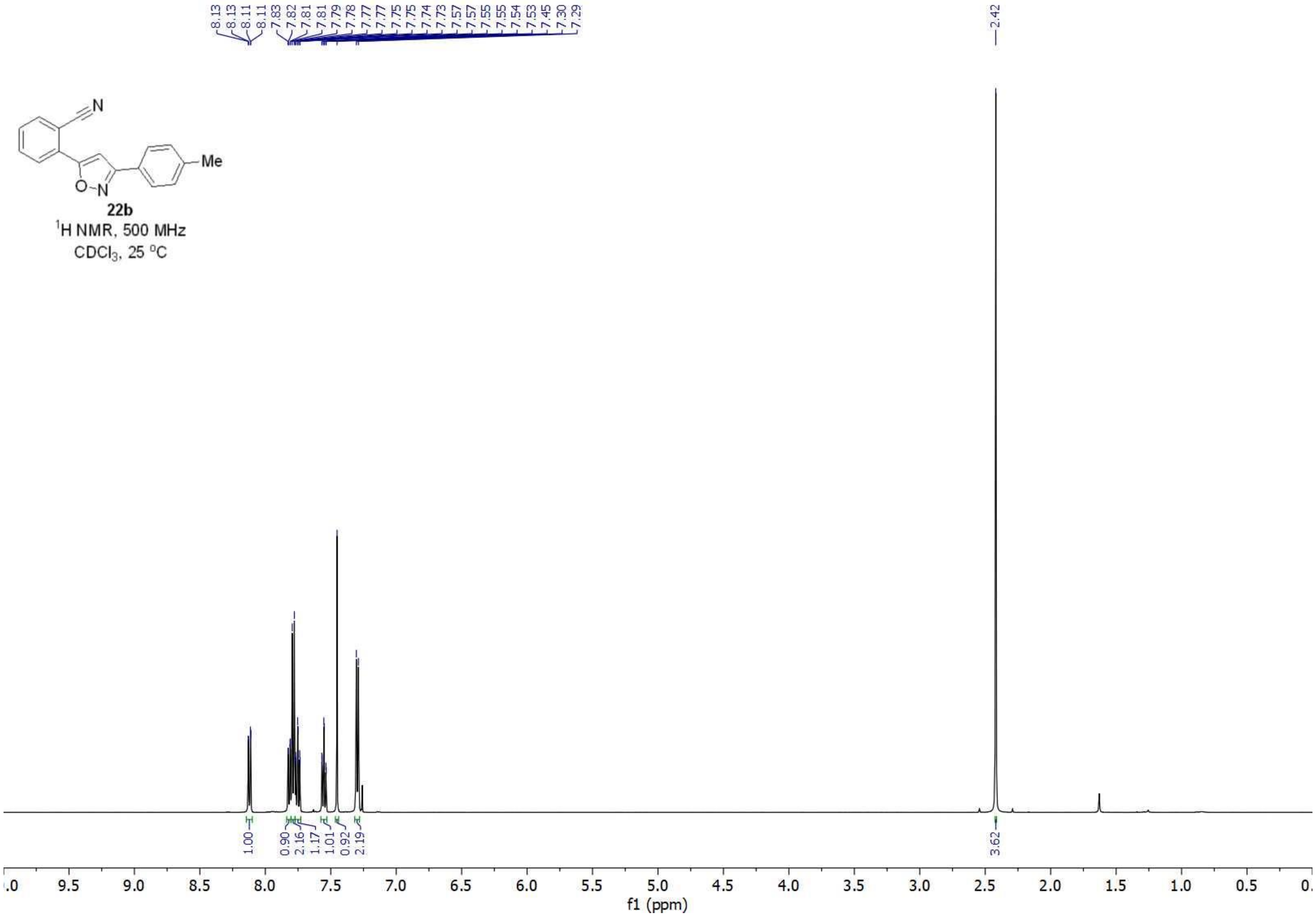
8.13
8.13
8.11
8.11
7.83
7.82
7.81
7.81
7.79
7.78
7.77
7.77
7.75
7.75
7.74
7.73
7.57
7.57
7.55
7.55
7.54
7.53
7.45
7.30
7.29
2.42
N
Me
O
N
22b
1H NMR, 500 MHz
CDCl3, 25 °C
1.00
0.90
2.16
1.17
1.01
0.92
2.19
3.62
9.5
9.0
8.5
8.0
7.5
7.0
6.5
6.0
5.5
5.0
4.5
4.0
3.5
3.0
2.5
2.0
1.5
1.0
0.5
f1 (ppm)

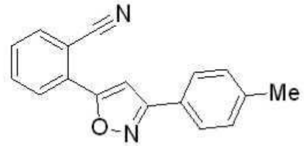

22b

^{13}C NMR, 125 MHz

$CDCl_3$, 25 °C

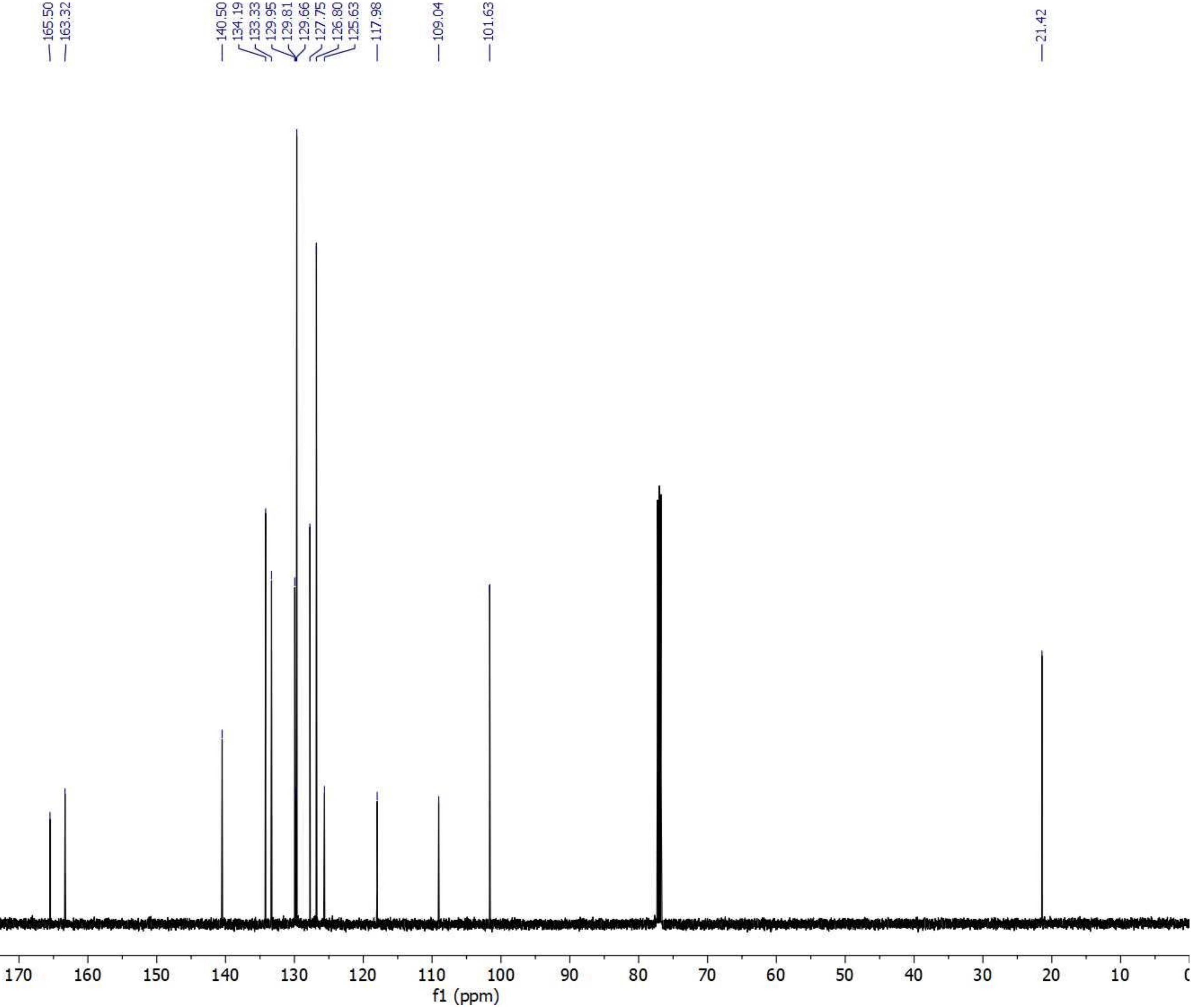

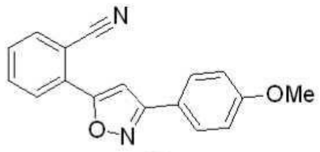

22c
^{1}H NMR, 500 MHz
$CDCl_3$, 25 °C

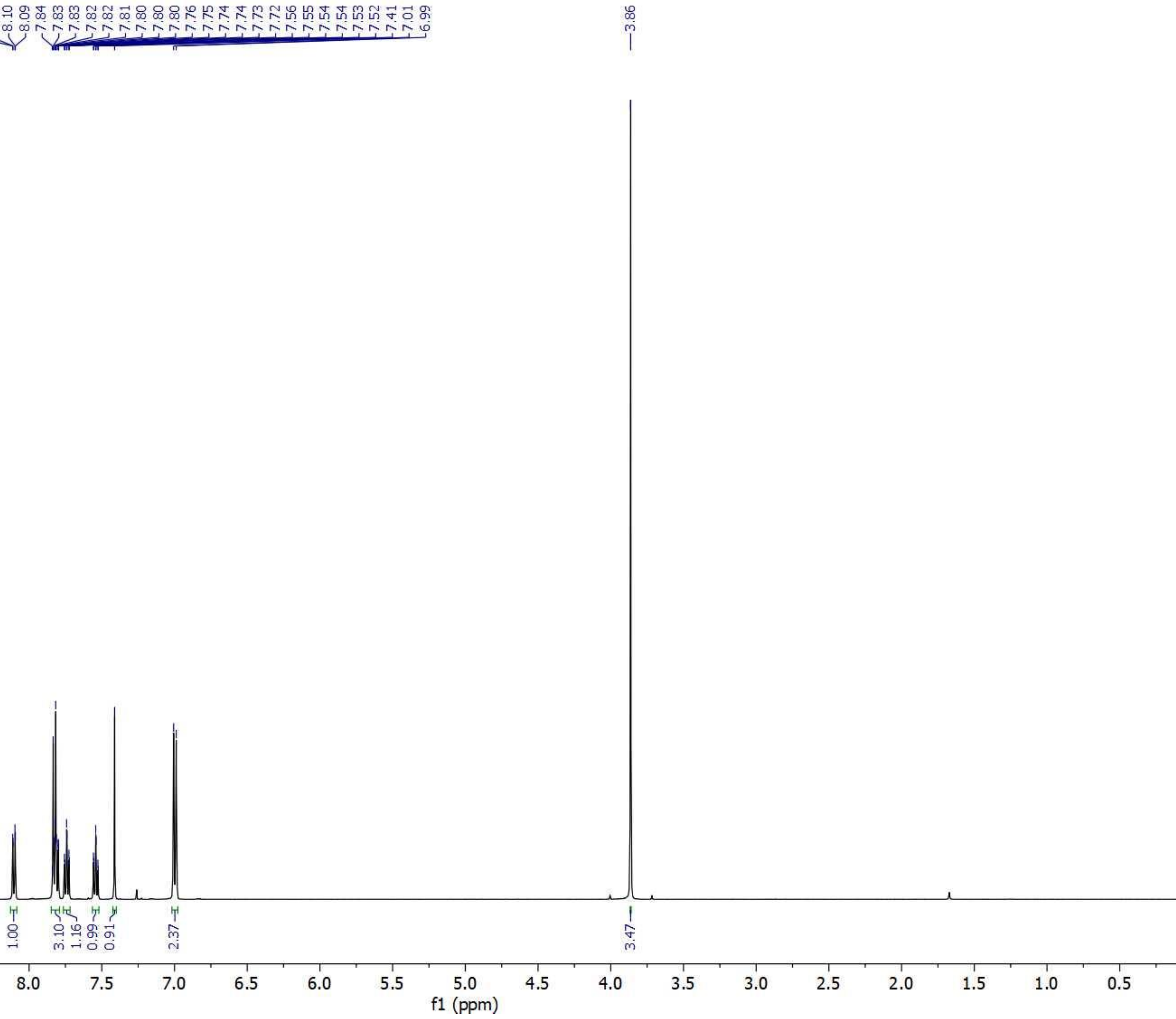

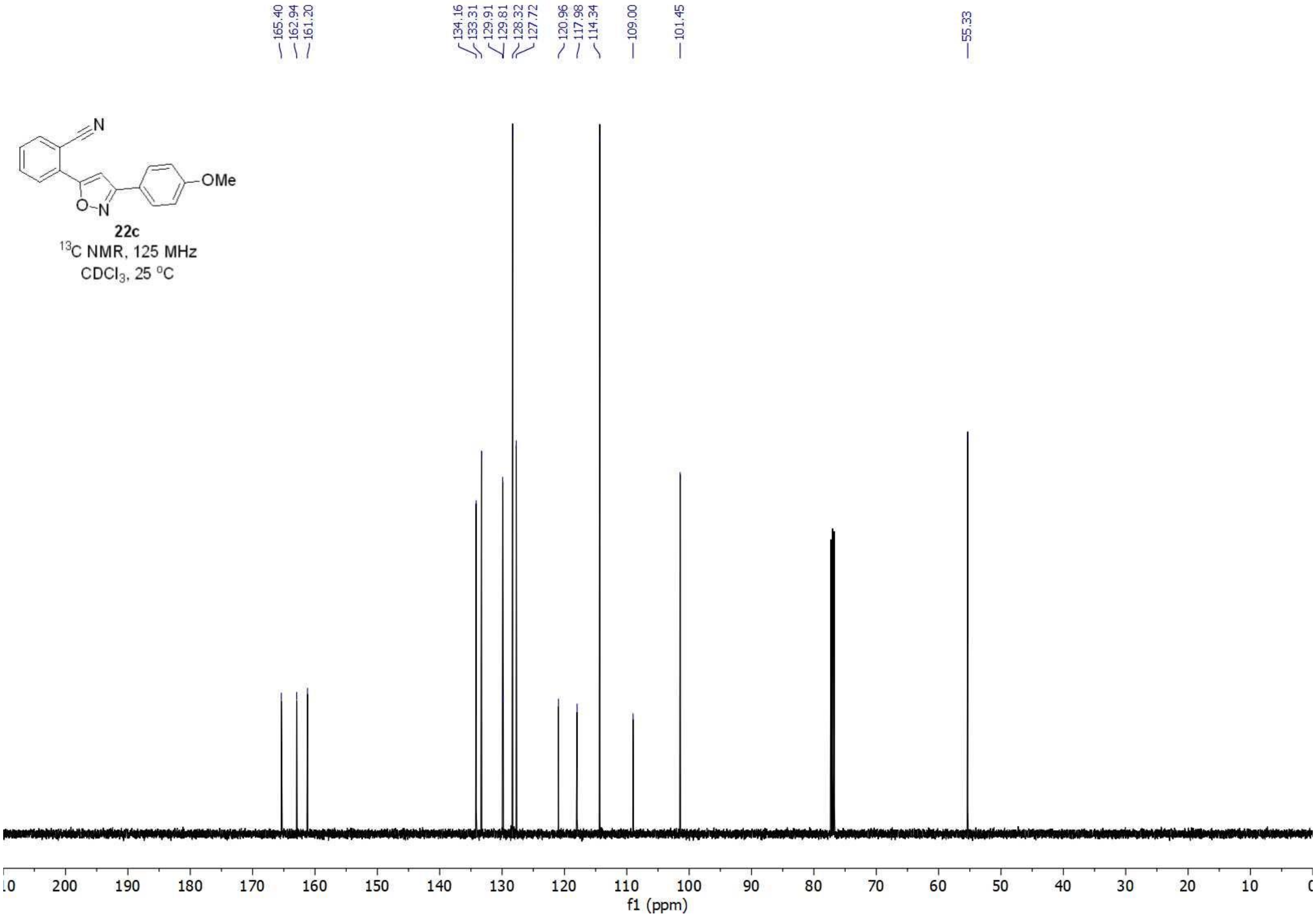

22c
^{13}C NMR, 125 MHz
$CDCl_3$, 25 °C

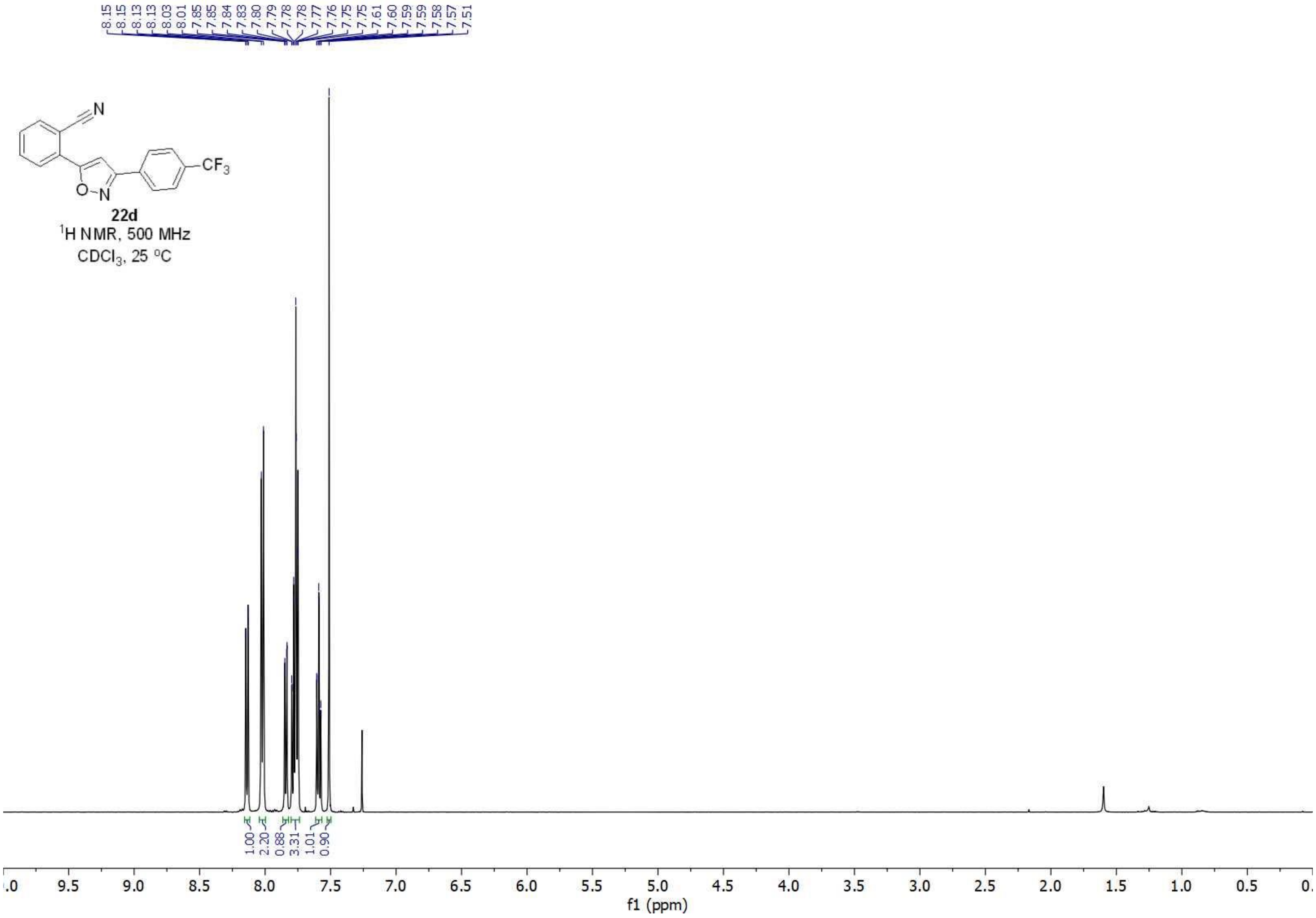
8.15
8.15
8.13
8.13
8.03
8.01
7.85
7.85
7.84
7.83
7.80
7.79
7.78
7.78
7.77
7.76
7.75
7.75
7.61
7.60
7.59
7.59
7.58
7.57
7.51
N
CF3
O
N
22d
1H NMR, 500 MHz
CDCl3, 25 °C
1.00
2.20
0.88
3.31
1.01
0.90
f1 (ppm)

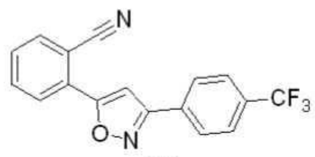

22d
^{13}C NMR, 125 MHz
$CDCl_3$, 25 °C

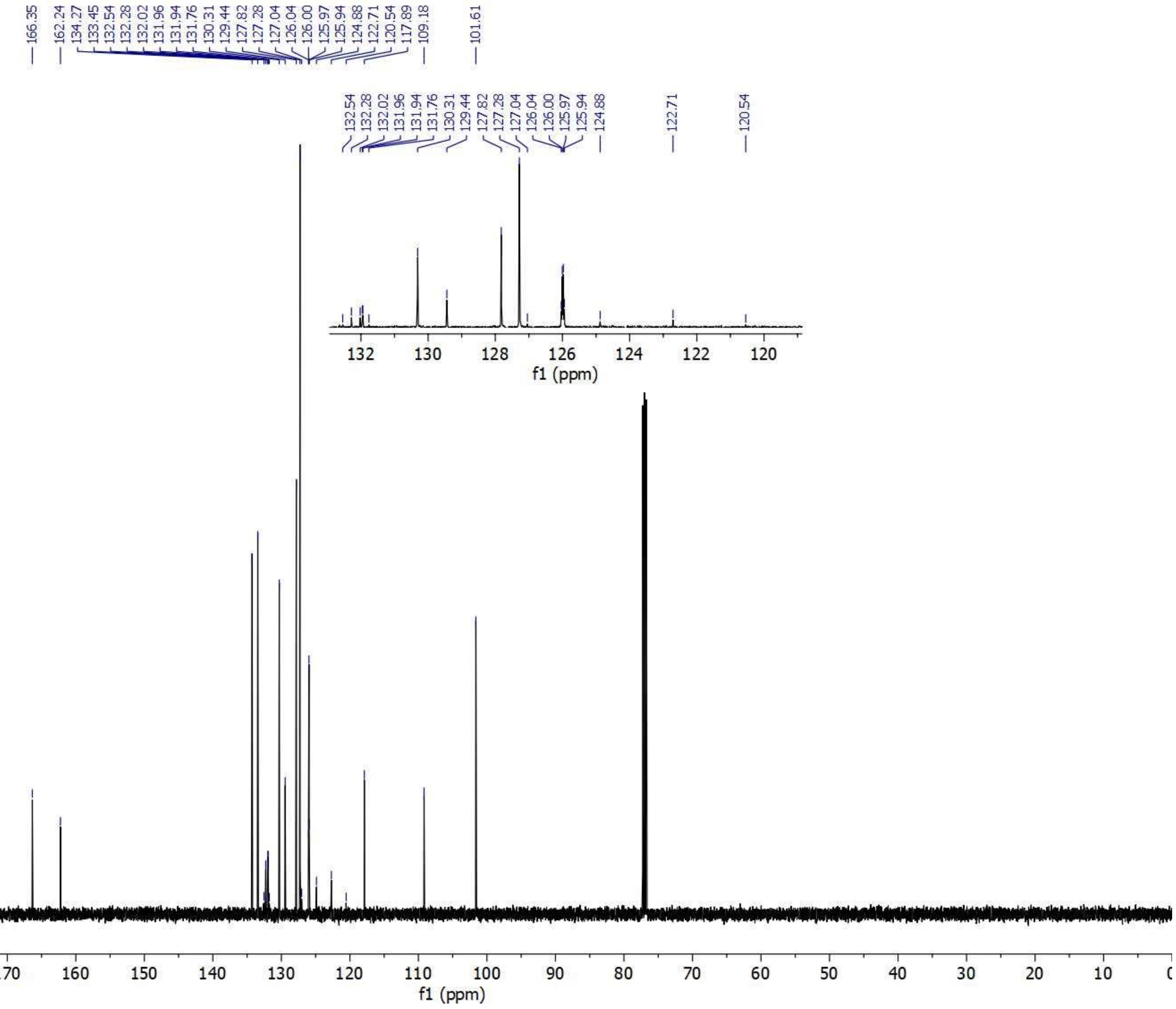

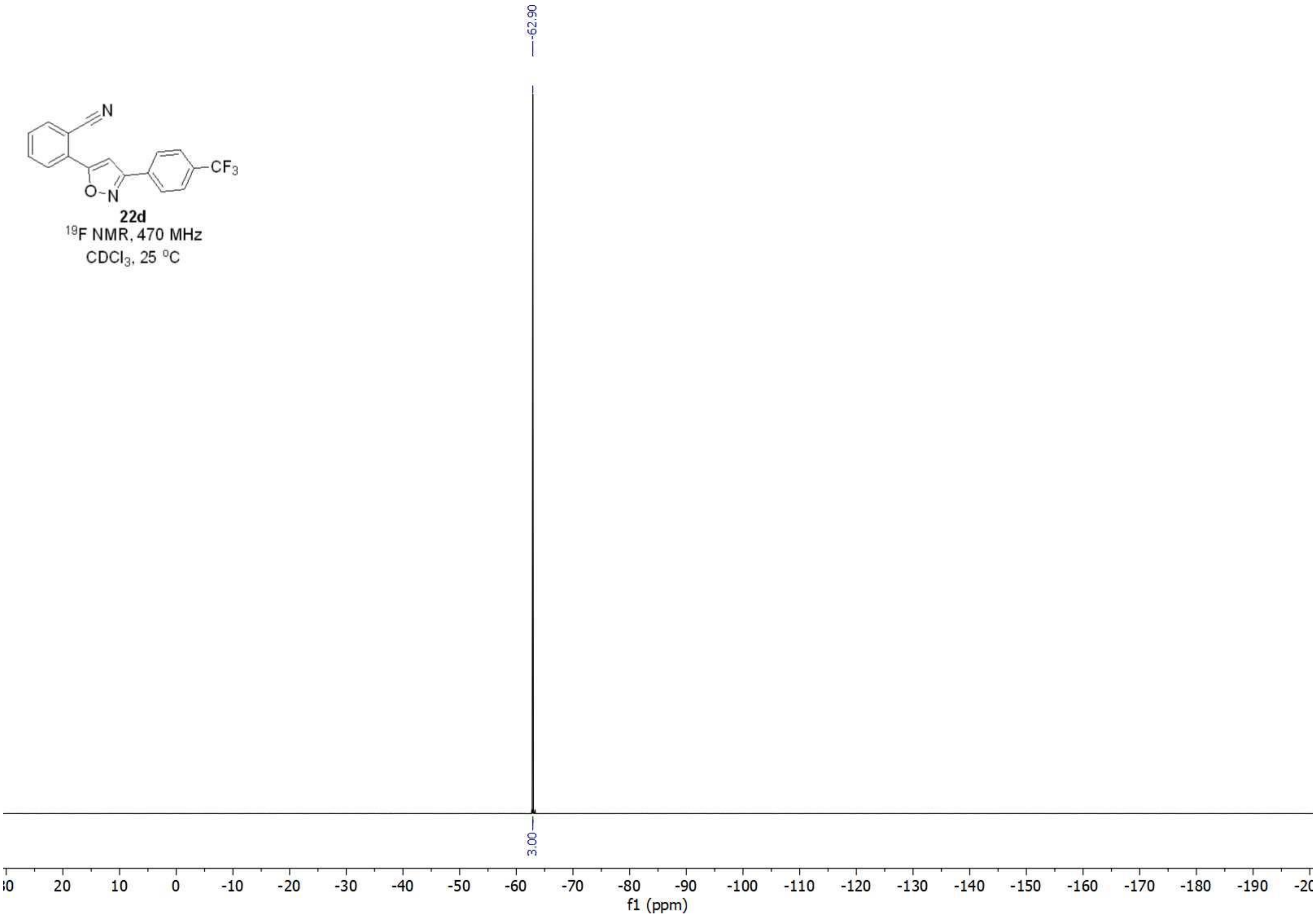

-62.90
N
CF3
O N
22d
19F NMR, 470 MHz
CDCl3, 25 °C
3.00
30 20 10 0 -10 -20 -30 -40 -50 -60 -70 -80 -90 -100 -110 -120 -130 -140 -150 -160 -170 -180 -190 -200
f1 (ppm)

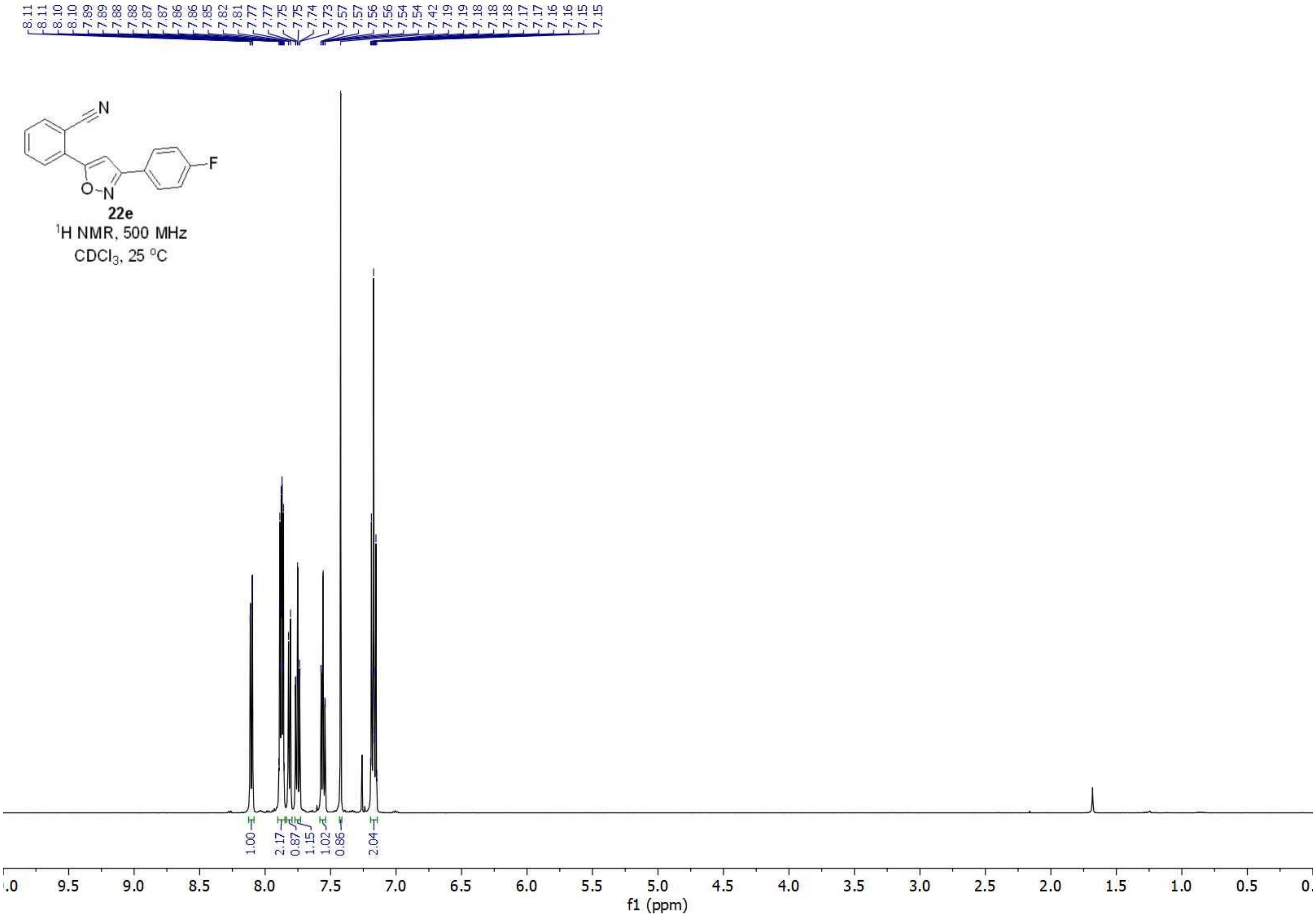
22e
¹H NMR, 500 MHz
CDCl3, 25 °C
f1 (ppm)

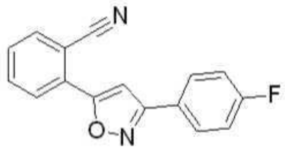

22e
^{13}C NMR, 125 MHz
$CDCl_3$, 25 °C

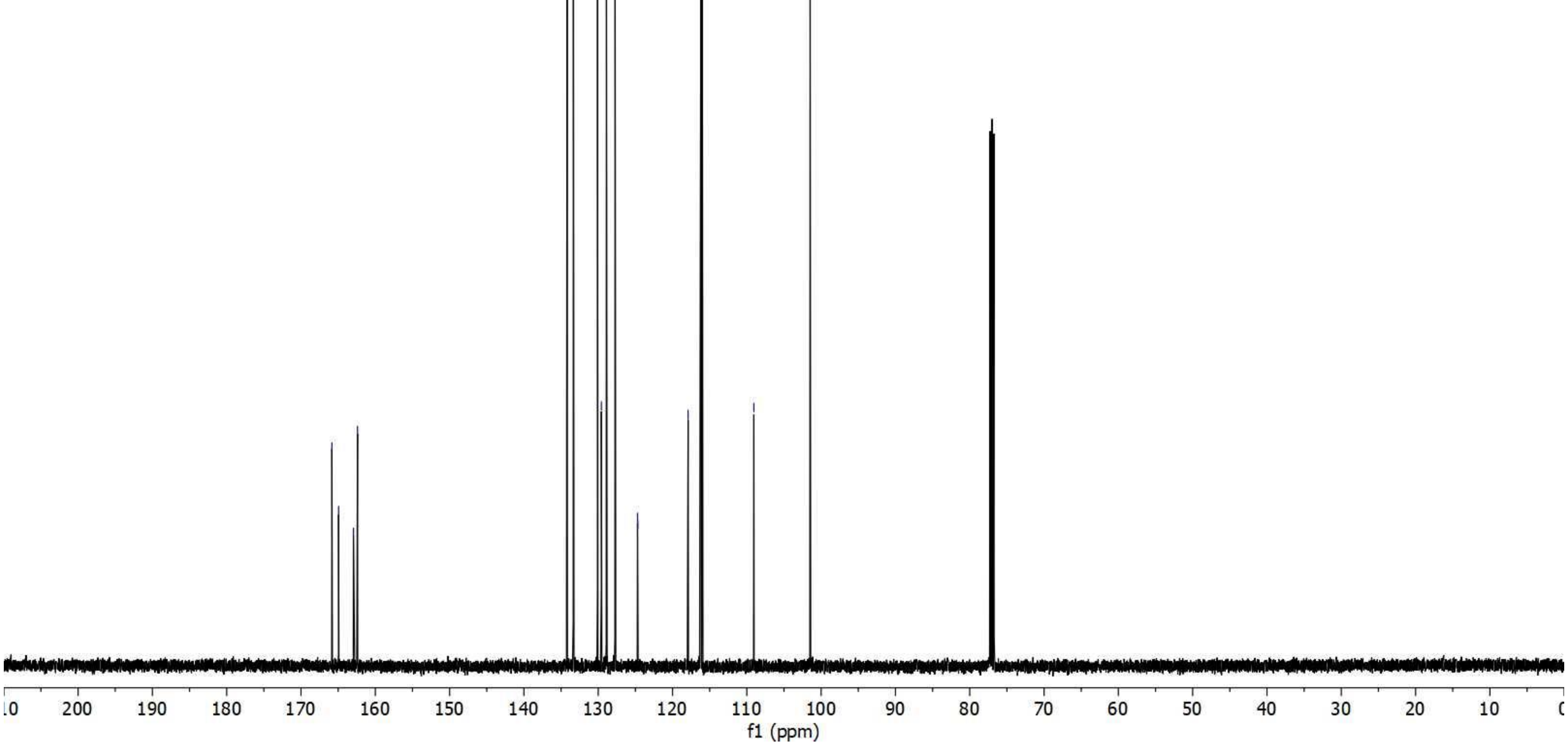

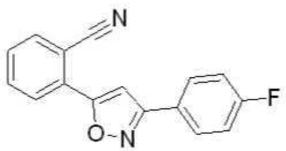

22e
^{19}F NMR, 470 MHz
$CDCl_3$, 25 °C

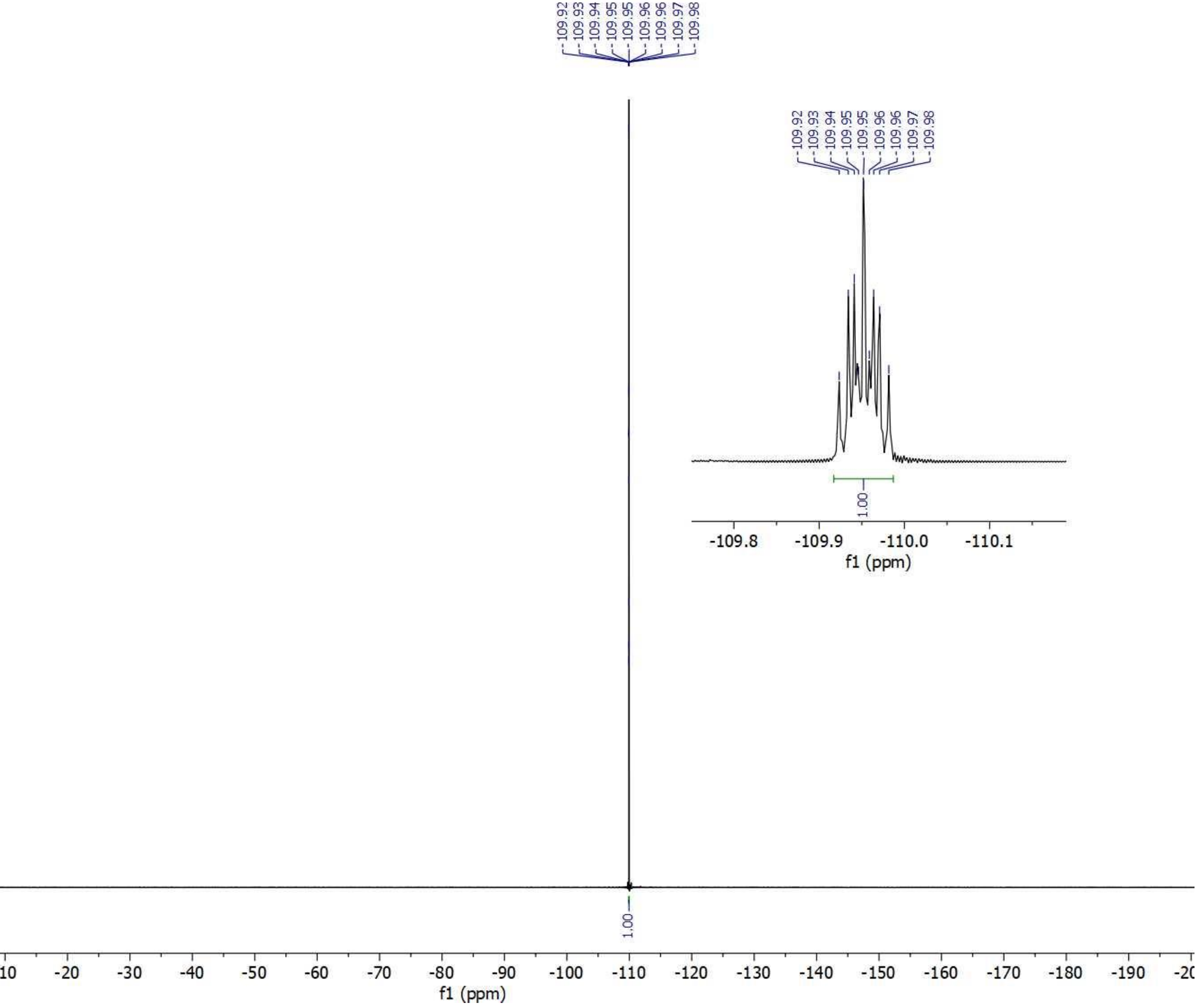

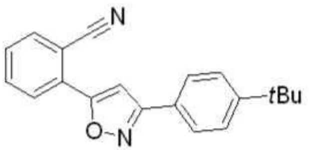

22f
^{1}H NMR, 500 MHz
$CDCl_3$, 25 °C

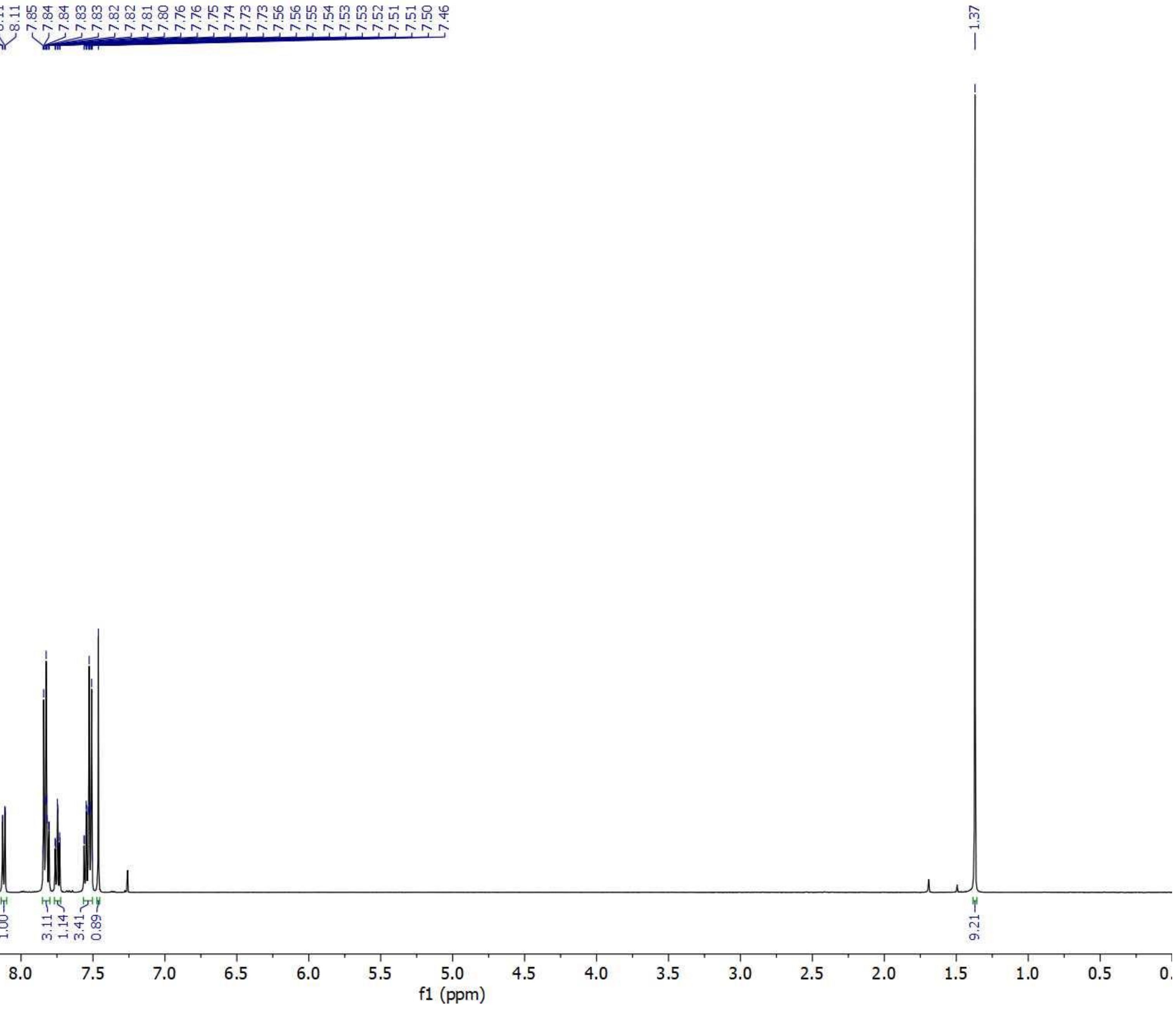

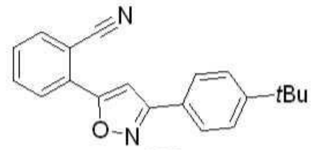

22f
^{13}C NMR, 125 MHz
$CDCl_3$, 25 ^{o}C

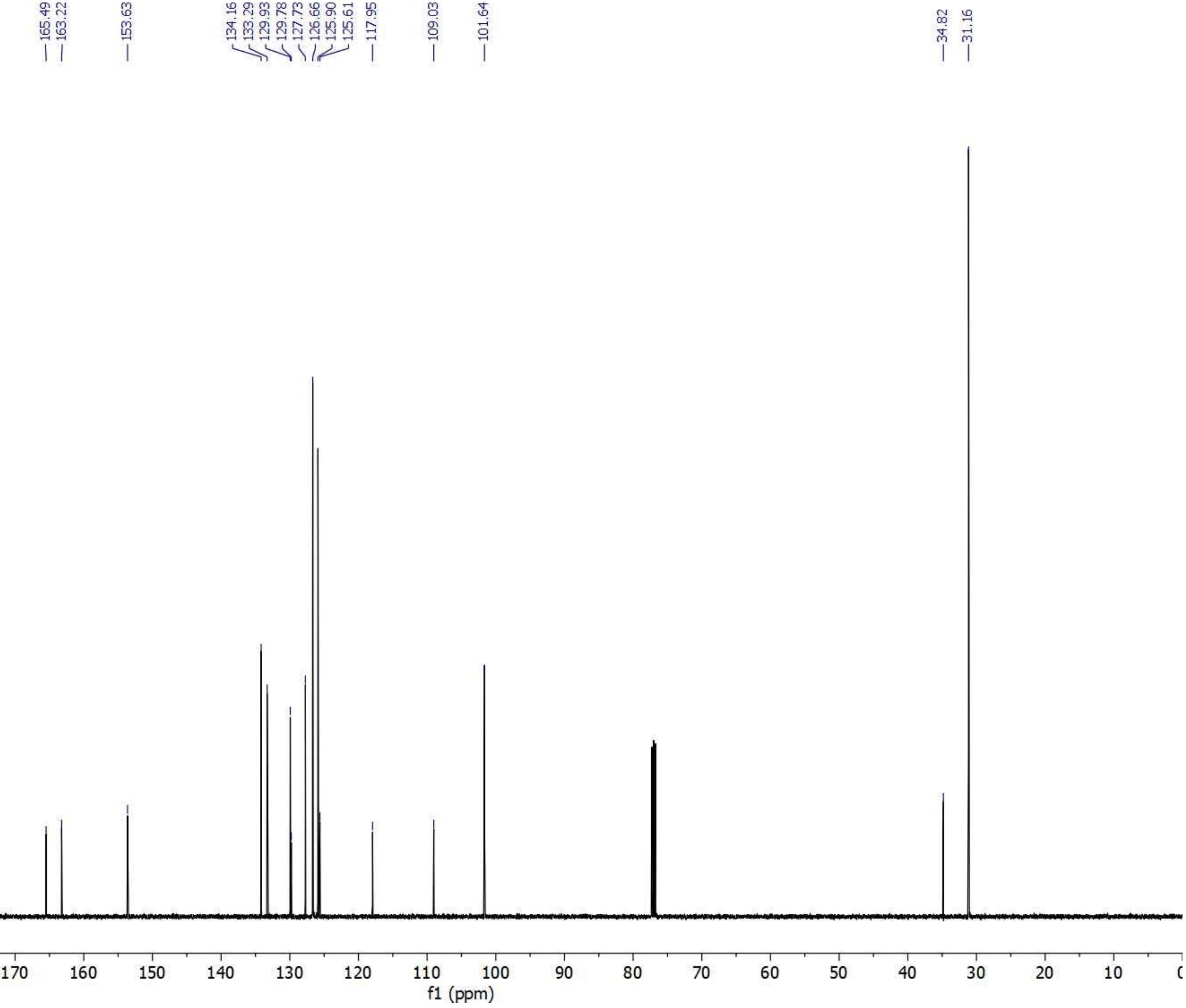

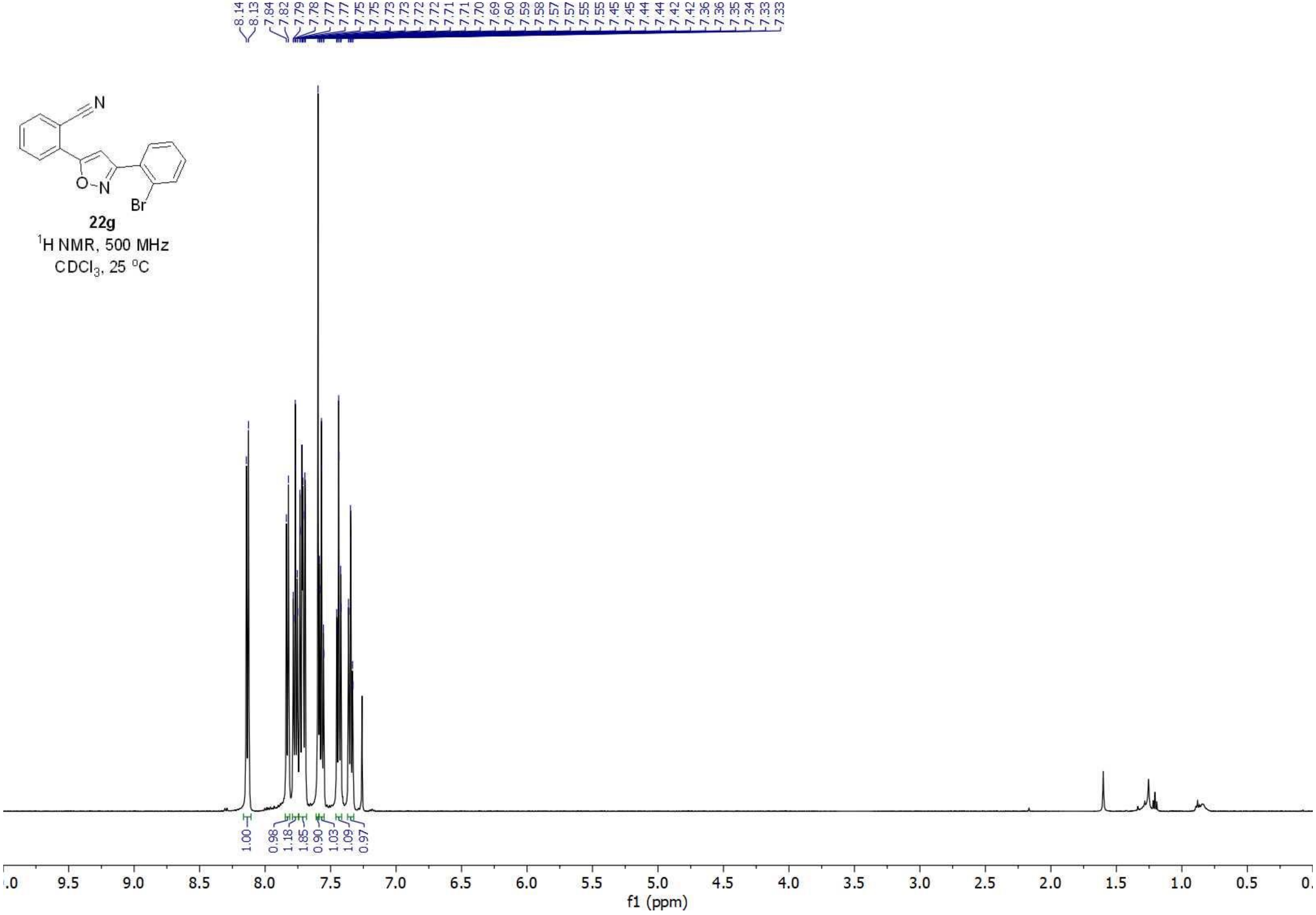
N
O
N
Br
22g
¹H NMR, 500 MHz
CDCl3, 25 °C
8.14
8.13
7.84
7.82
7.79
7.78
7.77
7.77
7.75
7.75
7.73
7.73
7.72
7.72
7.71
7.71
7.70
7.69
7.60
7.59
7.58
7.57
7.57
7.55
7.55
7.45
7.45
7.44
7.44
7.42
7.42
7.36
7.36
7.35
7.34
7.33
7.33
1.00
0.98
1.18
1.85
0.90
1.03
1.09
0.97
f1 (ppm)

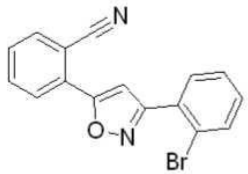

22g

^{13}C NMR, 125 MHz

$CDCl_3$, 25 °C

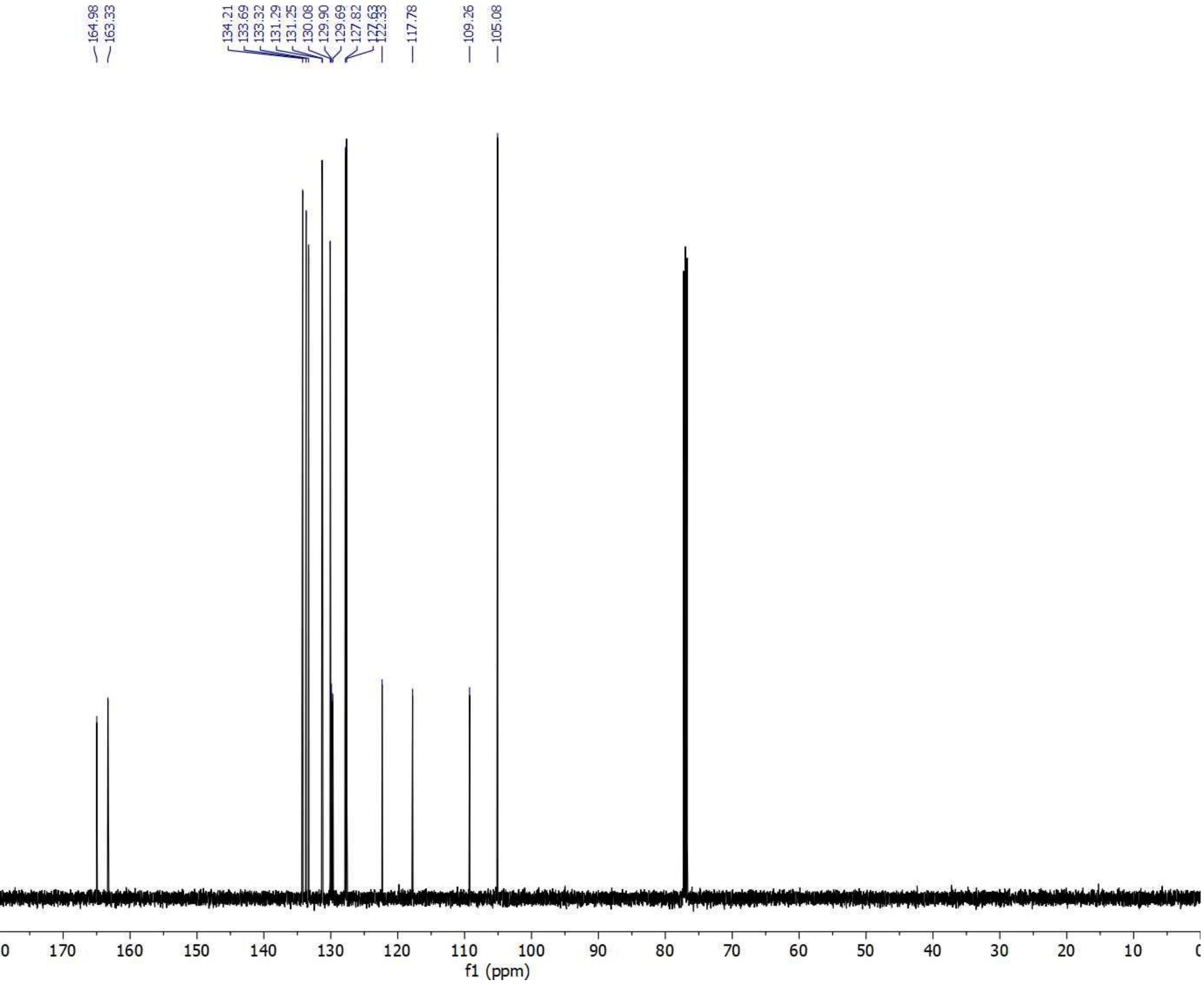

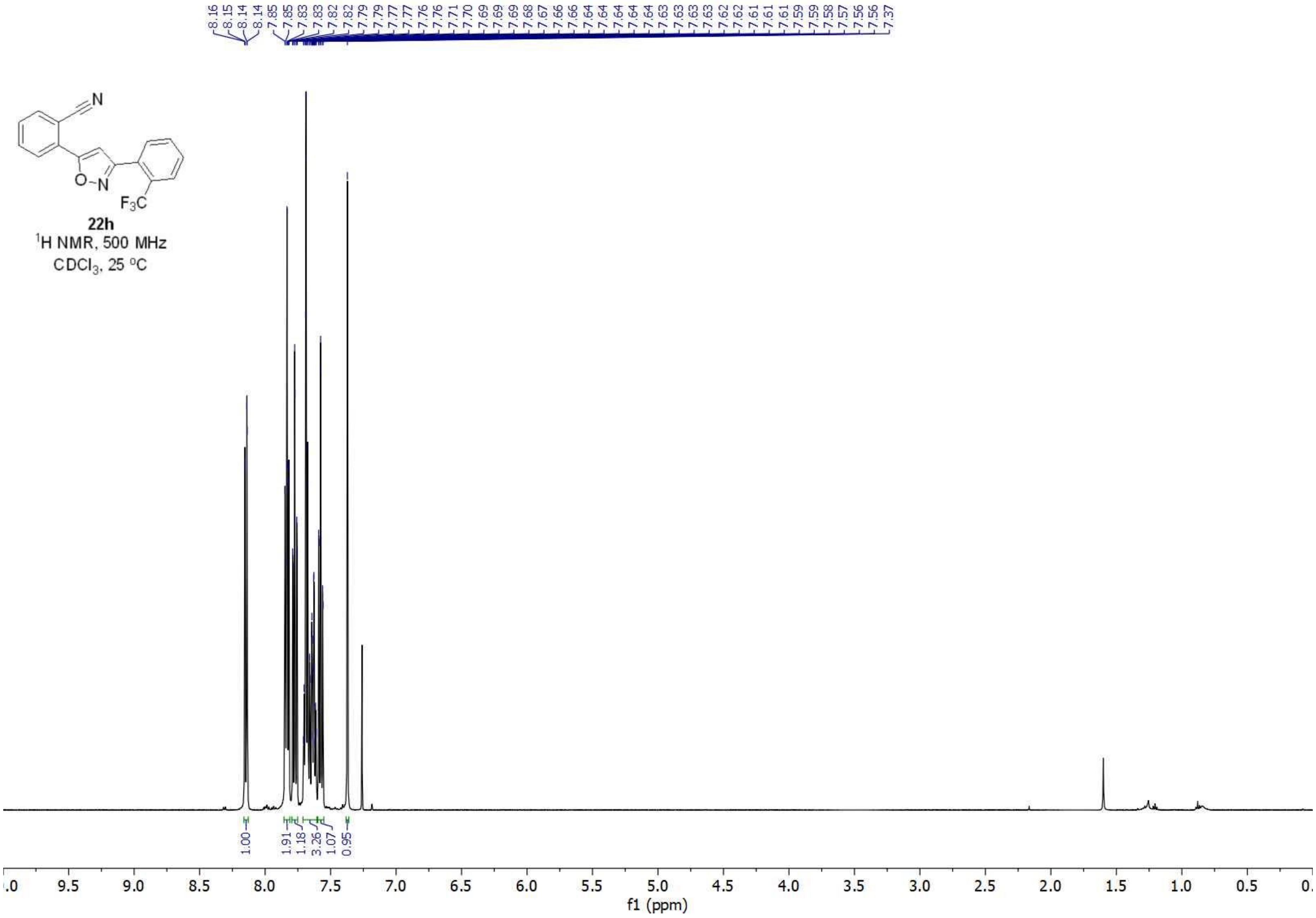
8.16
8.15
8.14
8.14
7.85
7.85
7.83
7.83
7.82
7.82
7.79
7.77
7.77
7.76
7.76
7.71
7.70
7.69
7.69
7.69
7.68
7.67
7.66
7.66
7.64
7.64
7.64
7.64
7.63
7.63
7.63
7.63
7.62
7.62
7.61
7.61
7.61
7.59
7.59
7.58
7.57
7.56
7.56
7.37
N
O
N
F_3C
22h
^{1}H NMR, 500 MHz
$CDCl_3$, 25 °C
1.00
1.91
1.18
3.26
1.07
0.95
9.5
9.0
8.5
8.0
7.5
7.0
6.5
6.0
5.5
5.0
4.5
4.0
3.5
3.0
2.5
2.0
1.5
1.0
0.5
f1 (ppm)

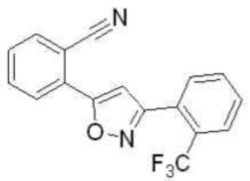

22h
^{13}C NMR, 125 MHz
$CDCl_3$, 25 °C

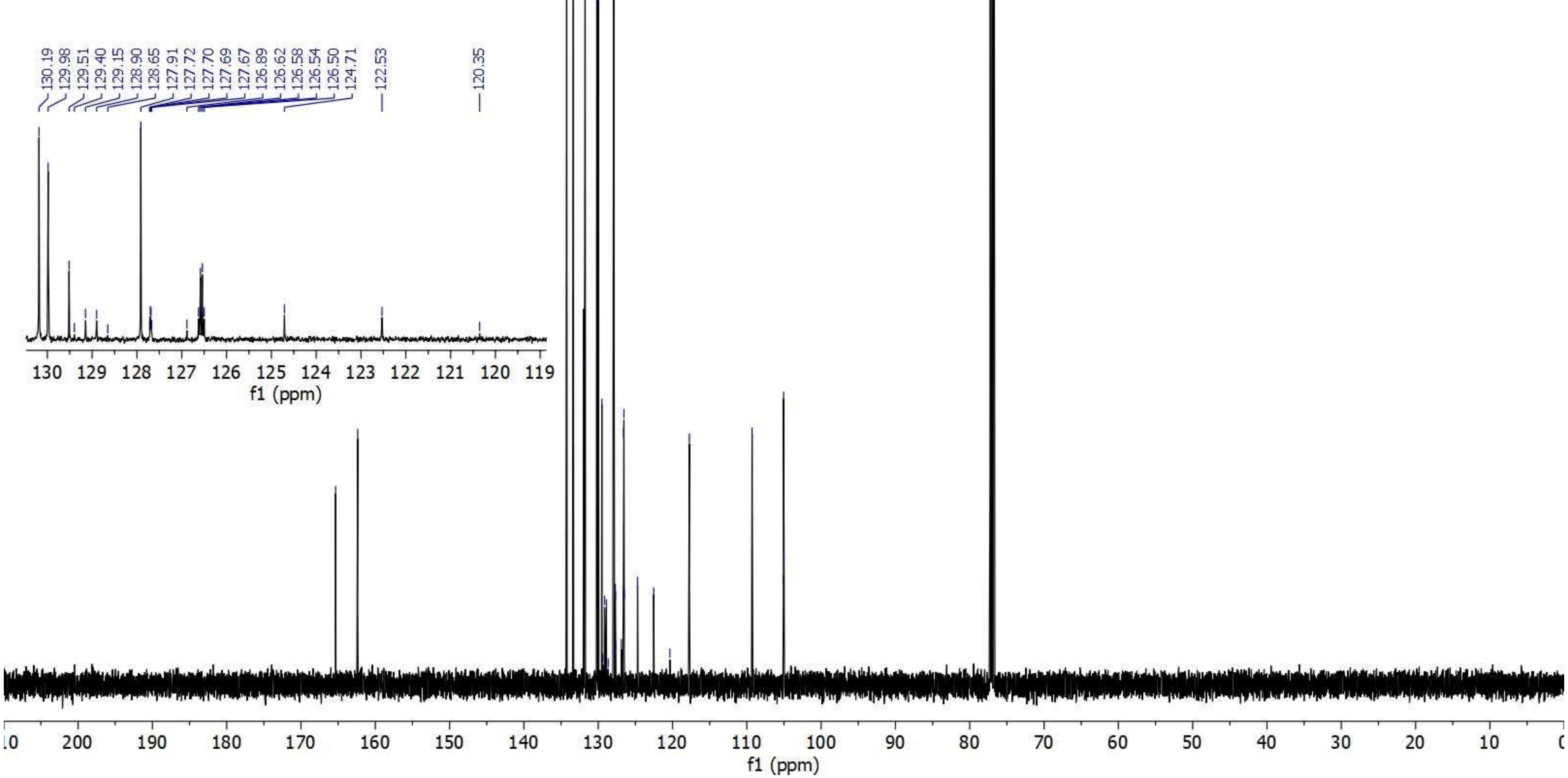

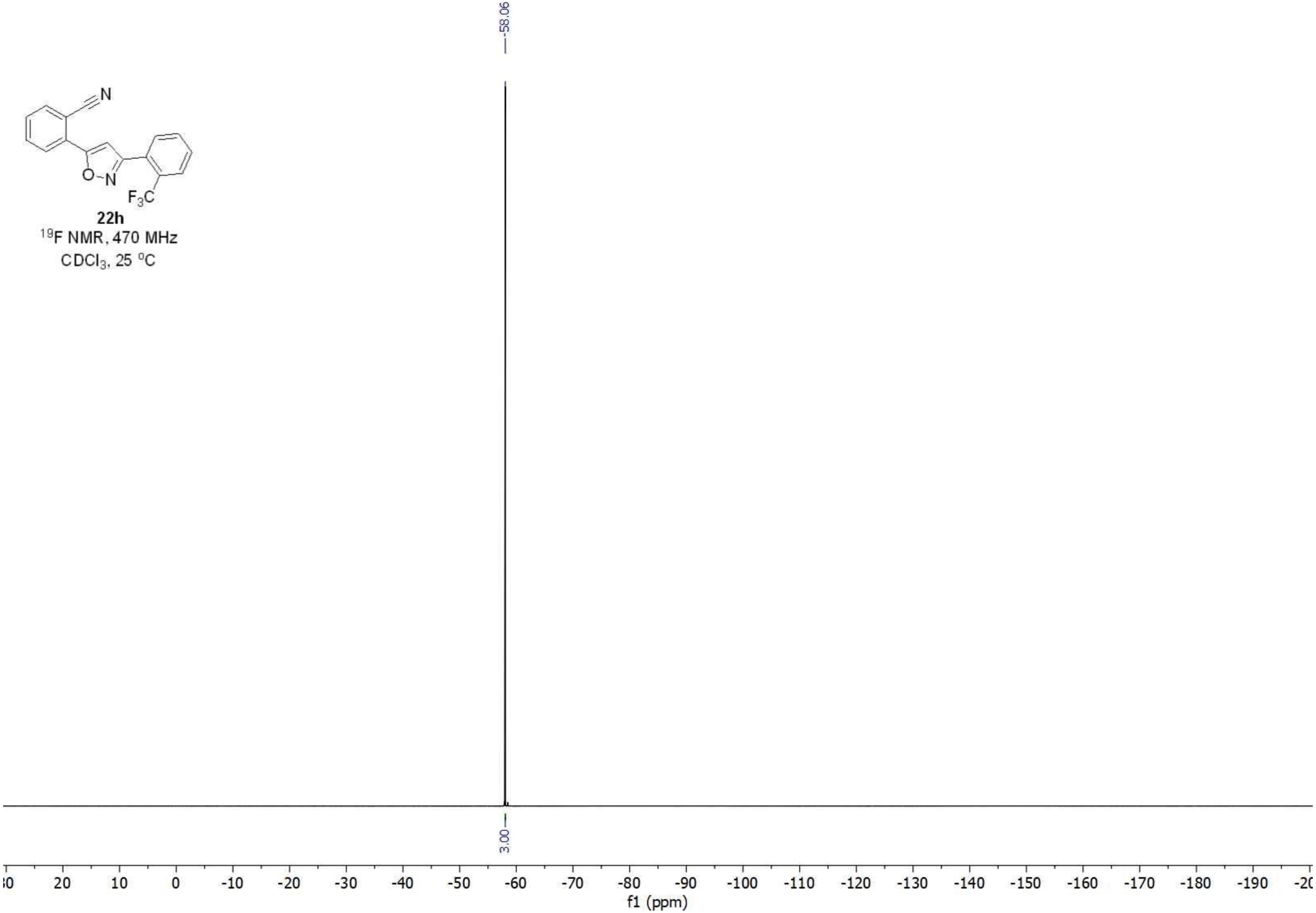
N
O
N
F_3C
22h
^{19}F NMR, 470 MHz
$CDCl_3$, 25 °C
-58.06
3.00
20
10
0
-10
-20
-30
-40
-50
-60
-70
-80
-90
-100
-110
-120
-130
-140
-150
-160
-170
-180
-190
f1 (ppm)

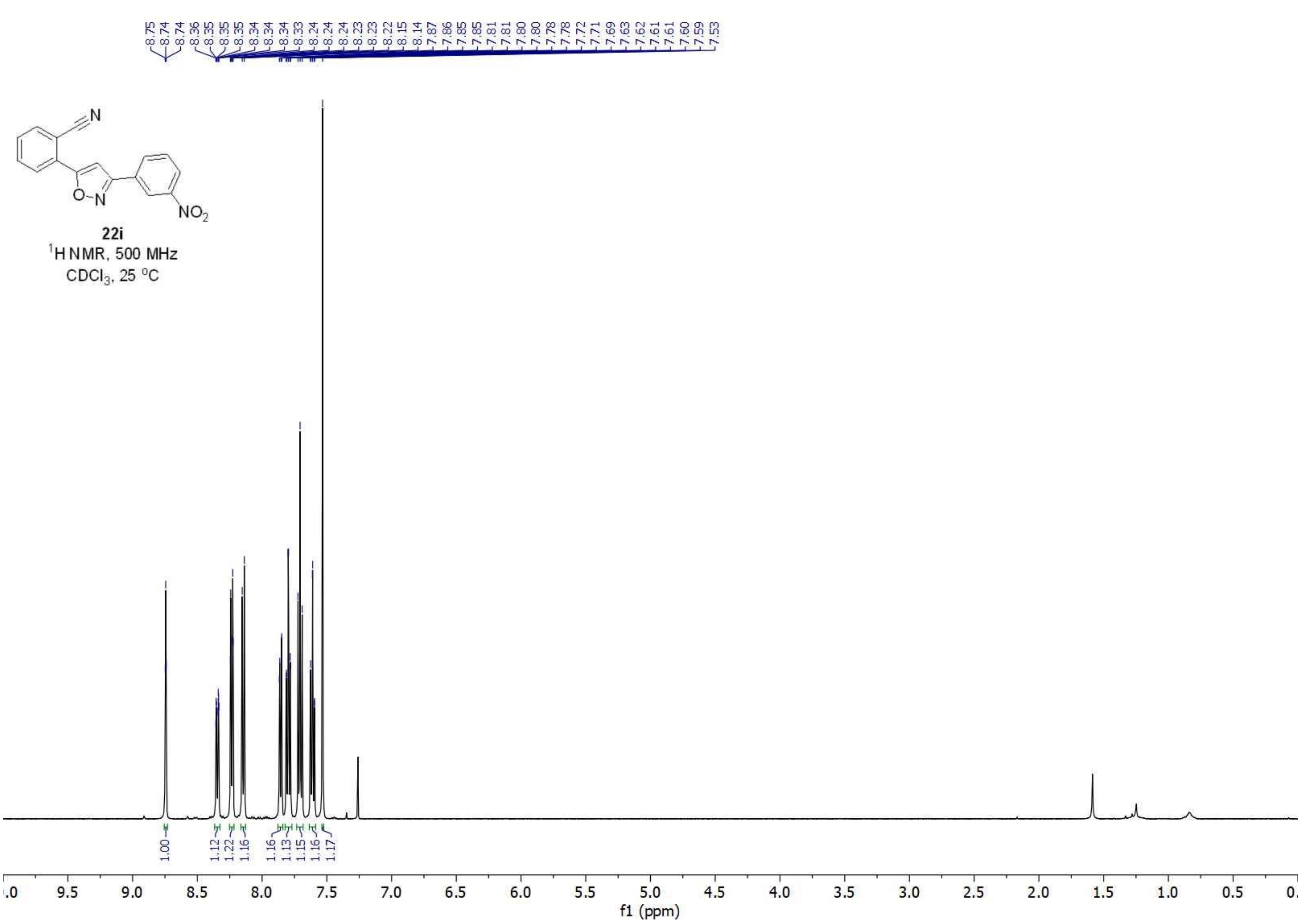
22i
^{1}H NMR, 500 MHz
$CDCl_3$, 25 °C
f1 (ppm)

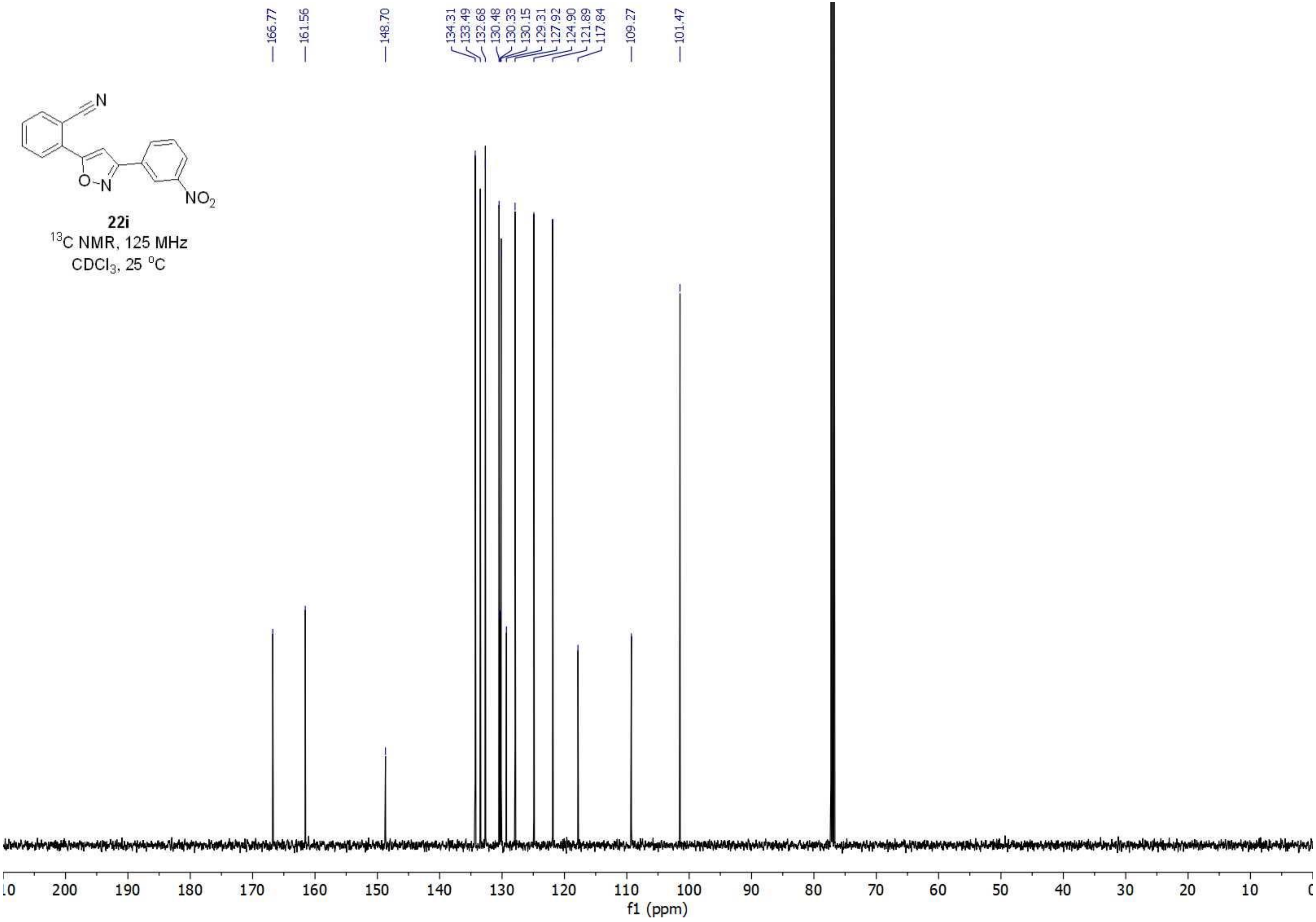
166.77
161.56
148.70
134.31
133.49
132.68
130.48
130.33
130.15
129.31
127.92
124.90
121.89
117.84
109.27
101.47
N
O
N
NO_2
22i
^{13}C NMR, 125 MHz
$CDCl_3$, 25 °C
200
190
180
170
160
150
140
130
120
110
100
90
80
70
60
50
40
30
20
10
f1 (ppm)

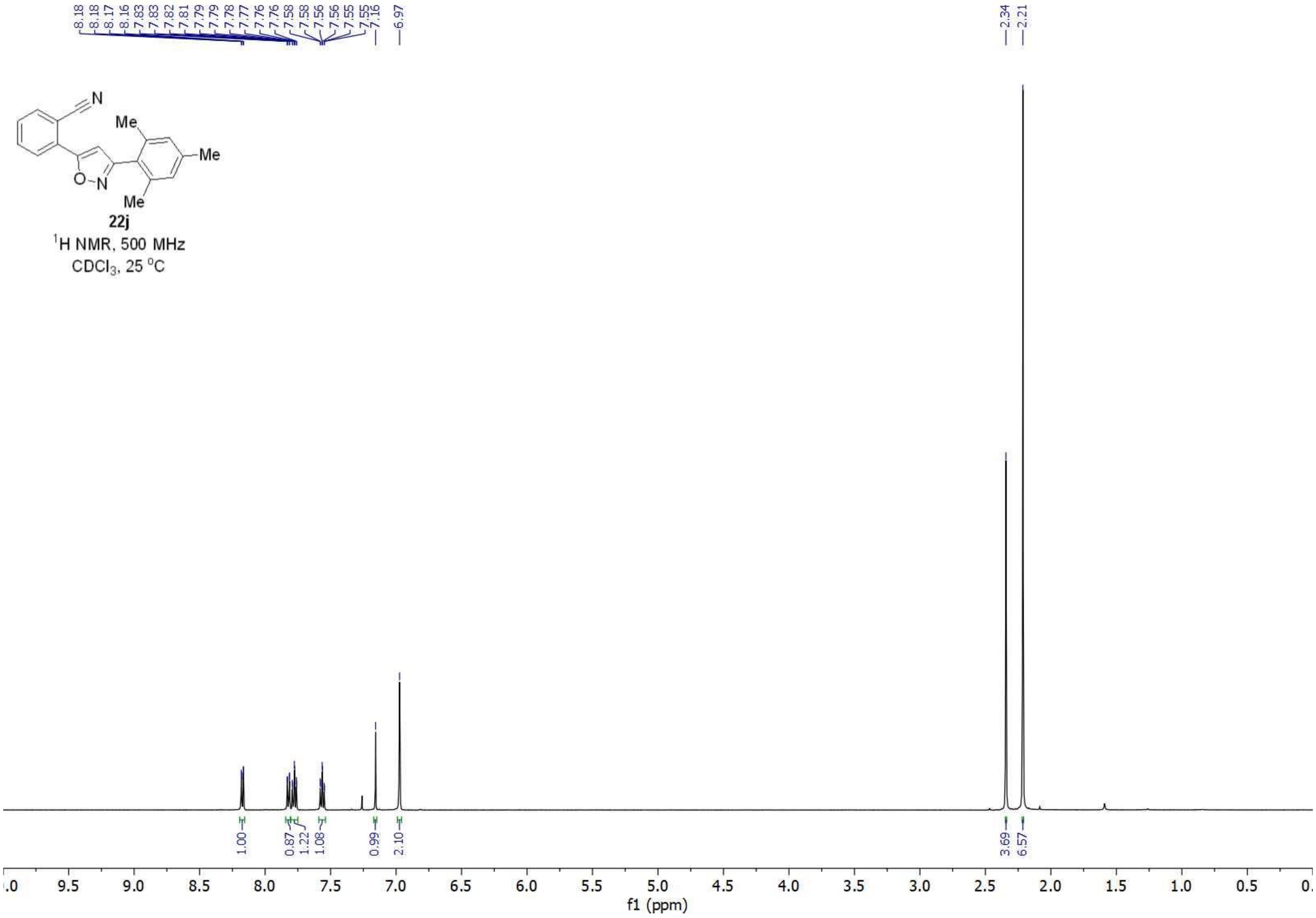

8.18
8.18
8.17
8.16
7.83
7.83
7.82
7.81
7.79
7.79
7.78
7.77
7.76
7.76
7.58
7.58
7.56
7.56
7.55
7.55
7.16
6.97
2.34
2.21
N
Me
Me
O
N
Me
22j
1H NMR, 500 MHz
CDCl3, 25 °C
1.00
0.87
1.22
1.08
0.99
2.10
3.69
6.57
9.5
9.0
8.5
8.0
7.5
7.0
6.5
6.0
5.5
5.0
4.5
4.0
3.5
3.0
2.5
2.0
1.5
1.0
0.5
f1 (ppm)

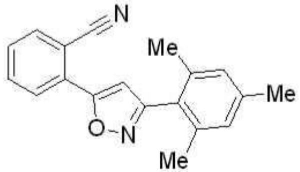

22j

^{13}C NMR, 125 MHz

$CDCl_3$, 25 °C

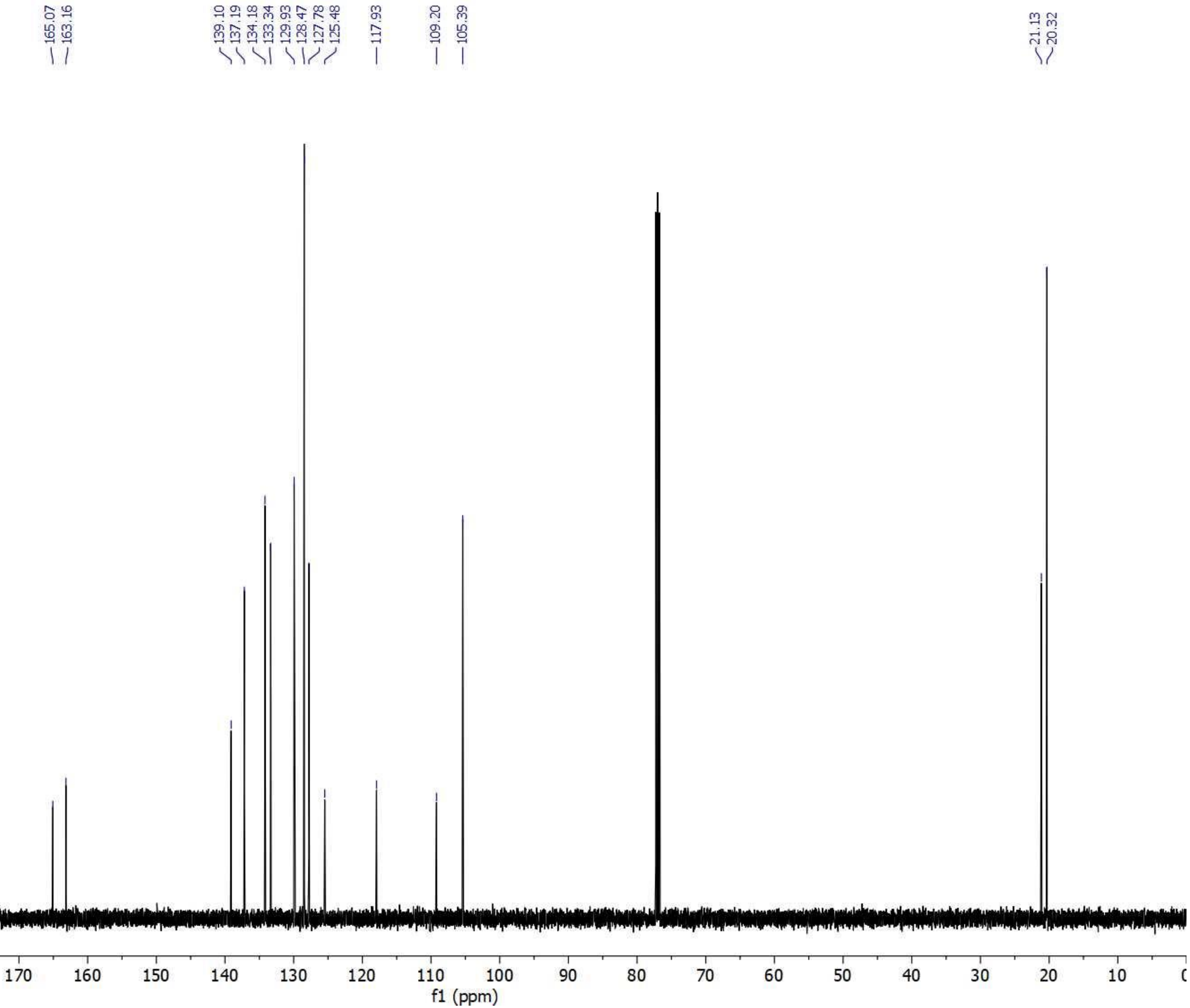

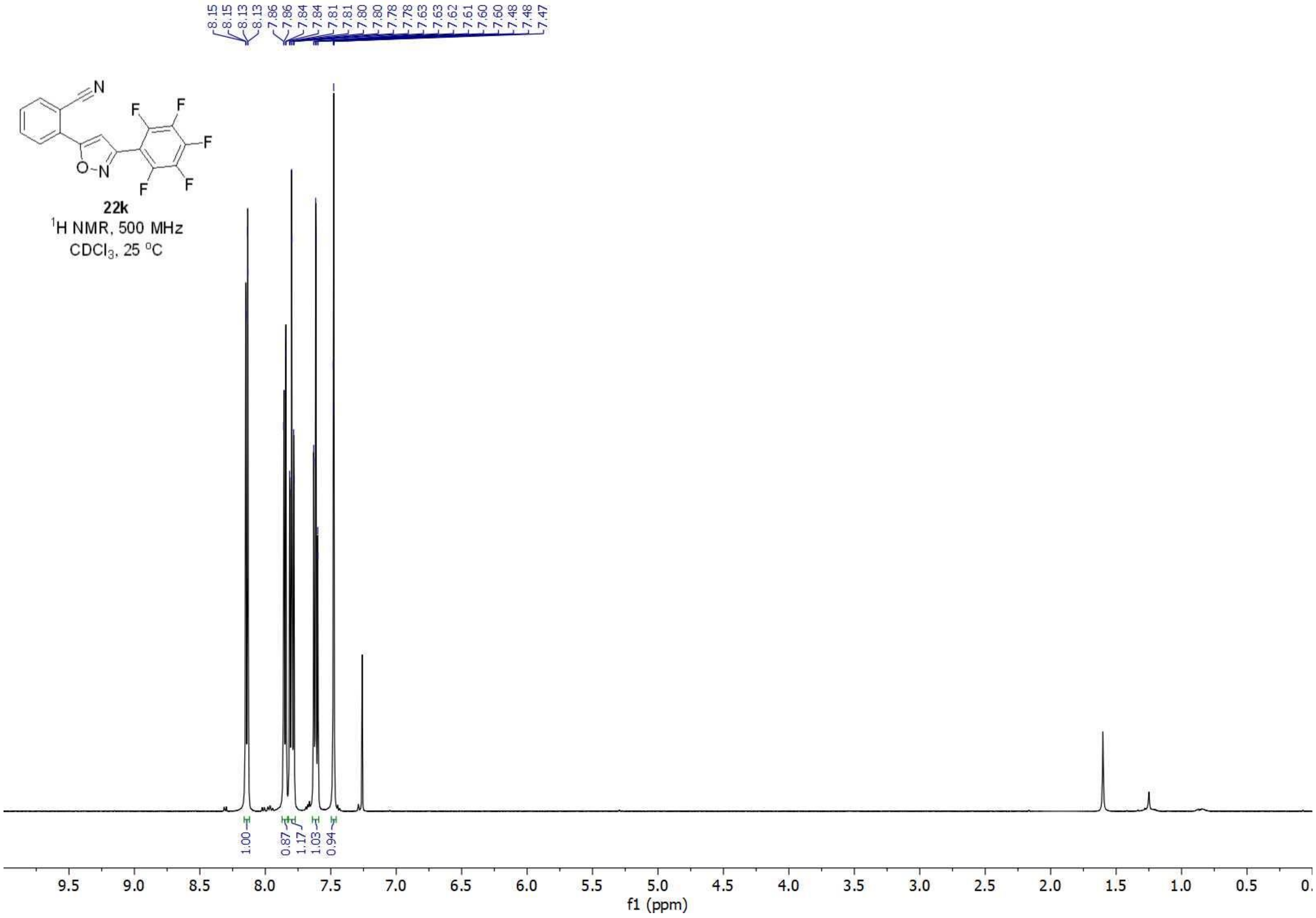
8.15
8.15
8.13
8.13
7.86
7.86
7.84
7.84
7.81
7.80
7.80
7.78
7.78
7.63
7.62
7.61
7.60
7.48
7.48
7.47
N
F
F
F
F
F
O
N
22k
^{1}H NMR, 500 MHz
$CDCl_3$, 25 °C
1.00
0.87
1.17
1.03
0.94
9.5
9.0
8.5
8.0
7.5
7.0
6.5
6.0
5.5
5.0
4.5
4.0
3.5
3.0
2.5
2.0
1.5
1.0
0.5
f1 (ppm)

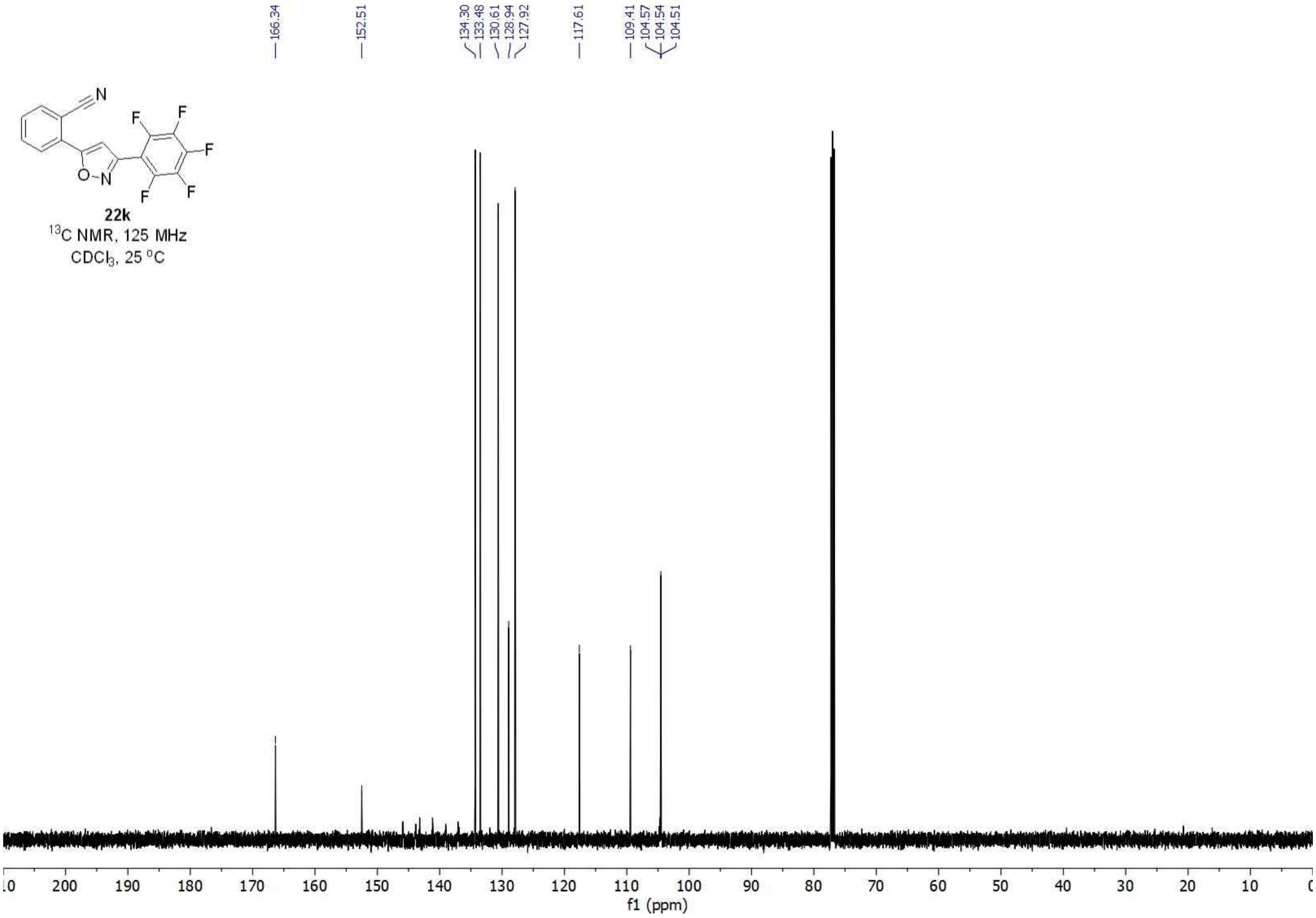
22k
^{13}C NMR, 125 MHz
$CDCl_3$, 25 °C
166.34
152.51
134.30
133.48
130.61
128.94
127.92
117.61
109.41
104.57
104.54
104.51
f1 (ppm)

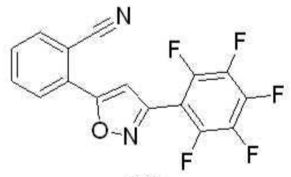

22k
^{19}F NMR, 470 MHz
$CDCl_3$, 25 °C

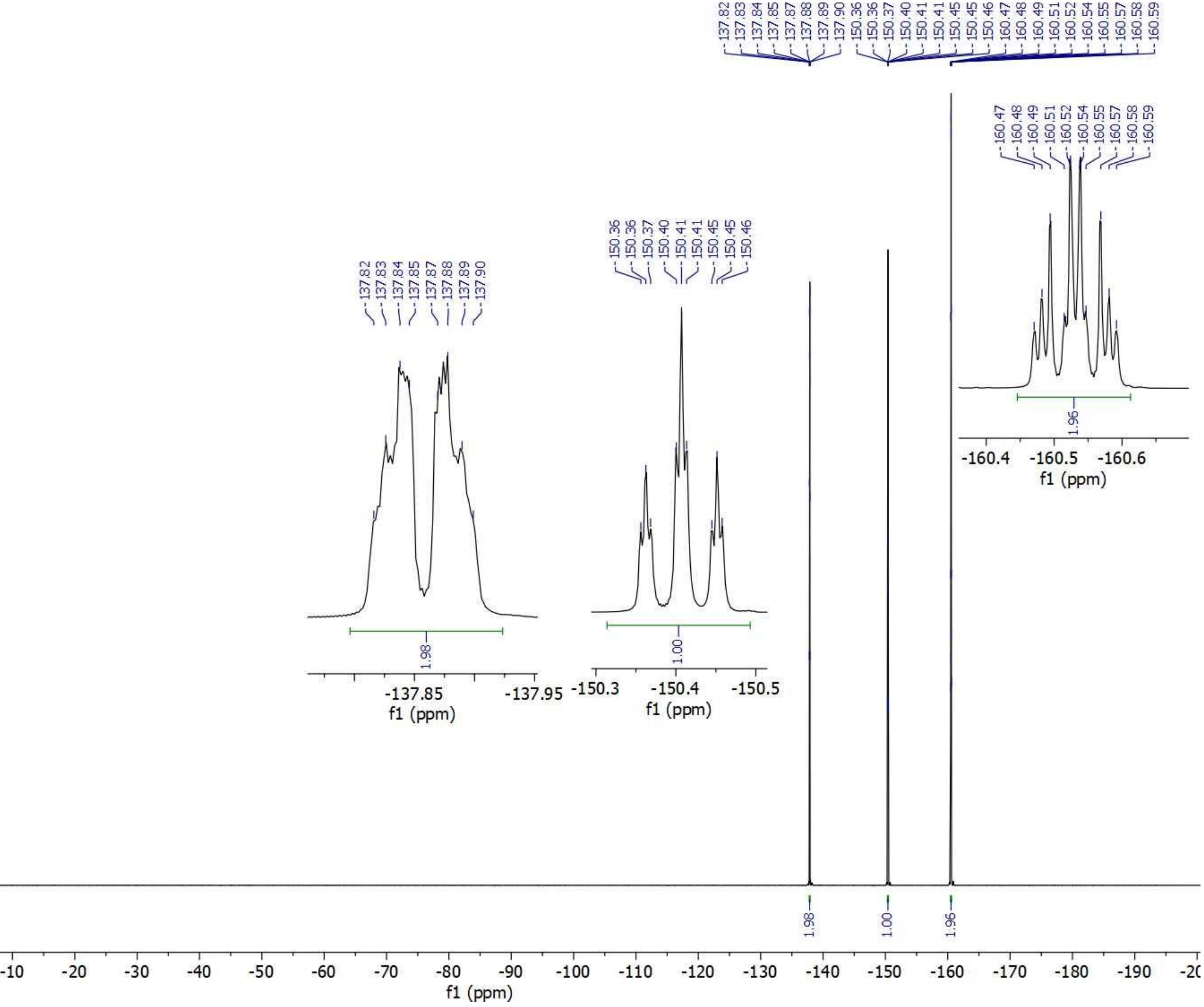

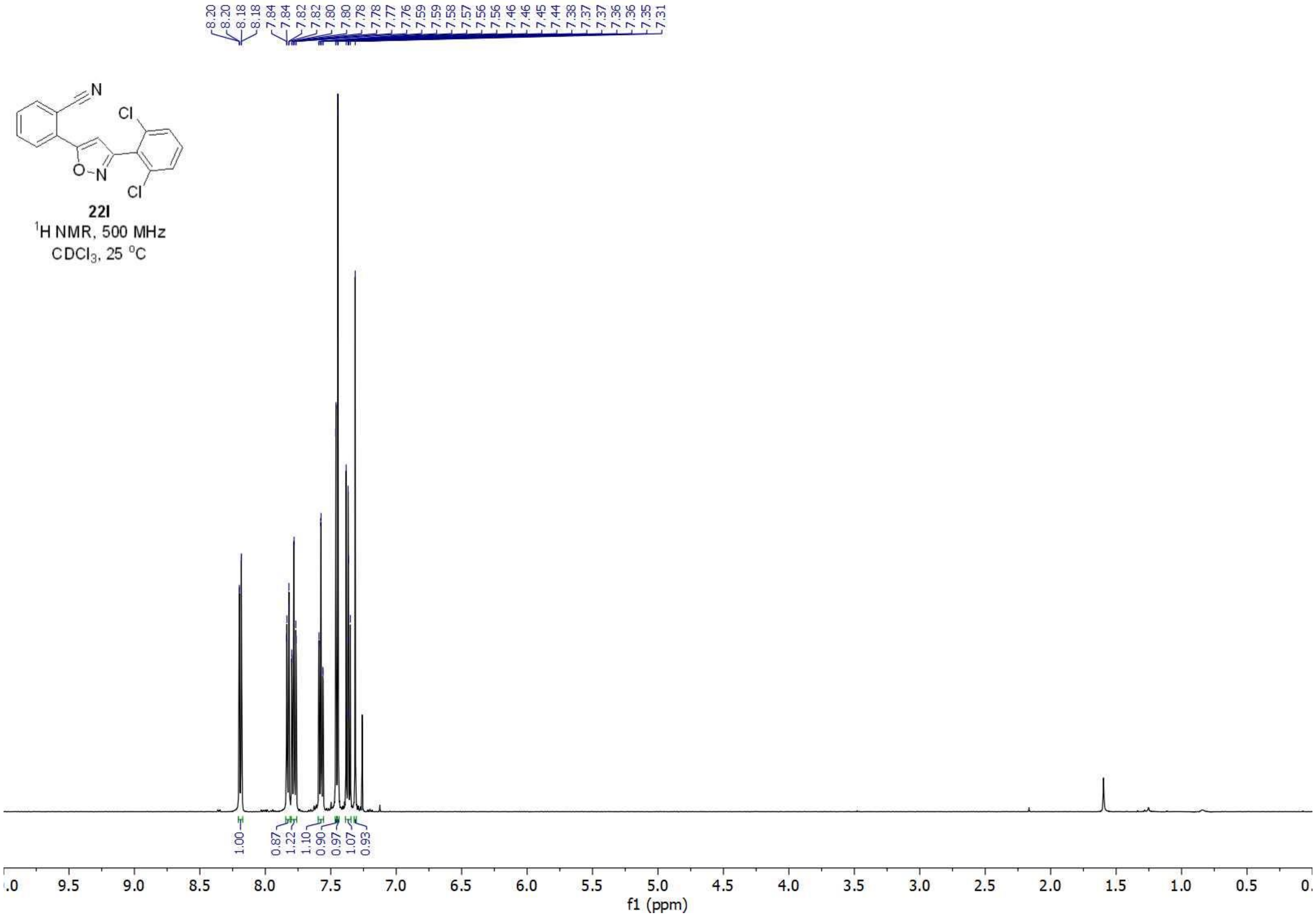
8.20
8.20
8.18
8.18
7.84
7.84
7.82
7.82
7.80
7.80
7.78
7.78
7.77
7.76
7.59
7.59
7.58
7.57
7.56
7.56
7.46
7.46
7.45
7.44
7.38
7.37
7.37
7.36
7.35
7.31
N
Cl
O
N
Cl
22l
^{1}H NMR, 500 MHz
$CDCl_3$, 25 °C
1.00
0.87
1.22
1.10
0.90
0.97
1.07
0.93
9.5
9.0
8.5
8.0
7.5
7.0
6.5
6.0
5.5
5.0
4.5
4.0
3.5
3.0
2.5
2.0
1.5
1.0
0.5
f1 (ppm)

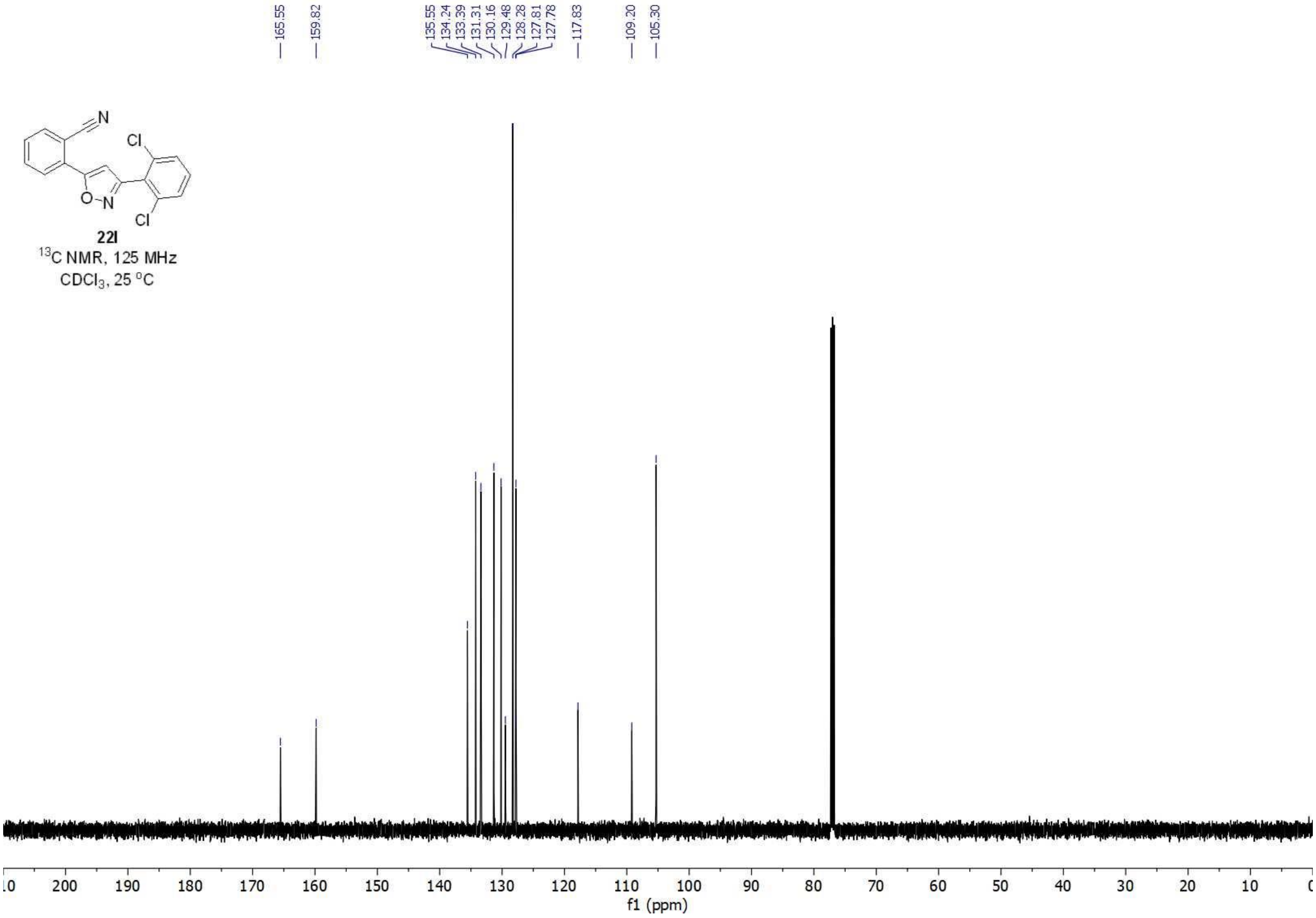
165.55
159.82
135.55
134.24
133.39
131.31
130.16
129.48
128.28
127.81
127.78
117.83
109.20
105.30
22l
13C NMR, 125 MHz
CDCl3, 25 °C
200 190 180 170 160 150 140 130 120 110 100 90 80 70 60 50 40 30 20 10
f1 (ppm)

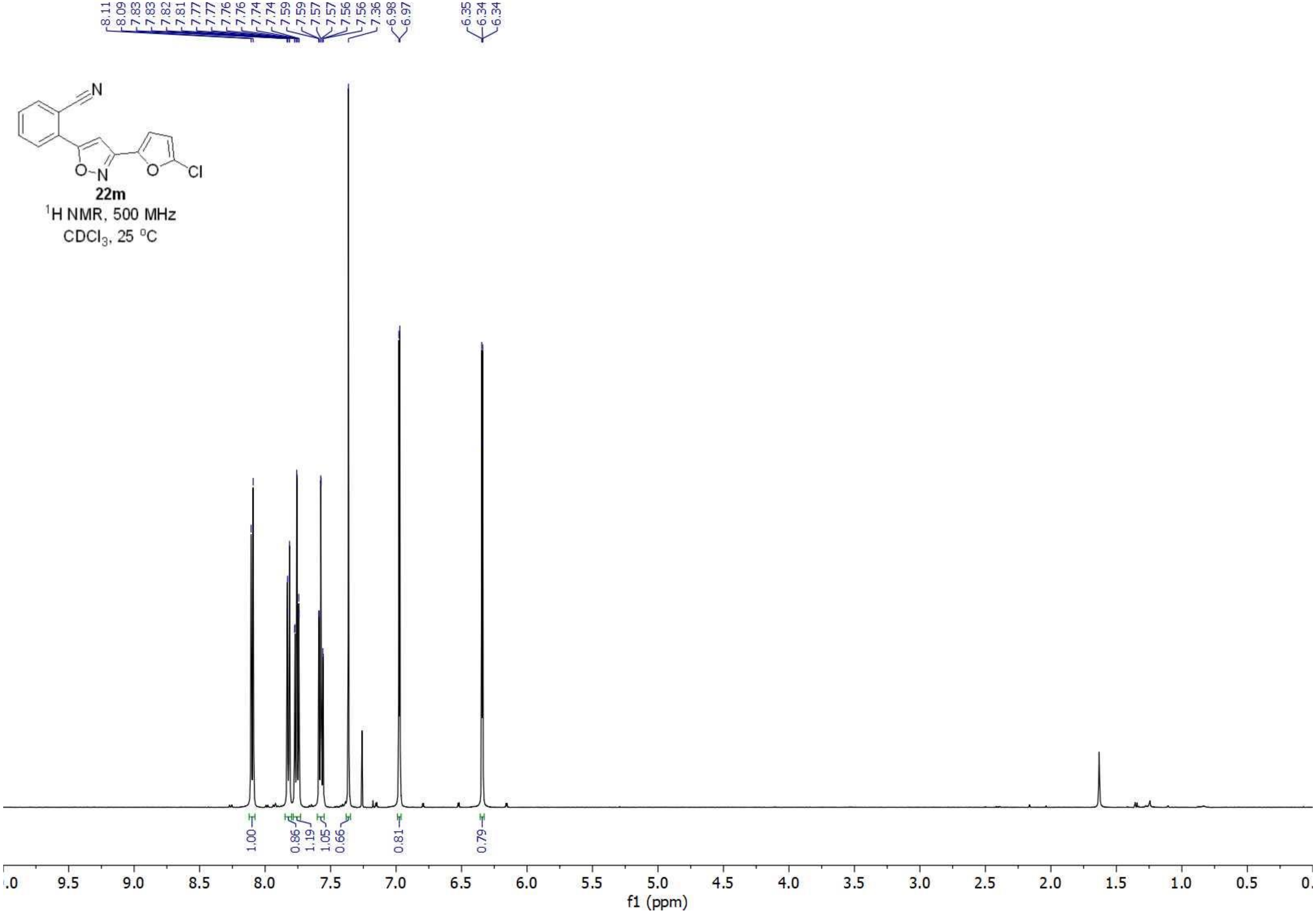
8.11
8.09
7.83
7.83
7.82
7.81
7.77
7.77
7.76
7.76
7.74
7.74
7.59
7.59
7.57
7.57
7.56
7.36
6.98
6.97
6.35
6.34
6.34
N
O
N
O
Cl
22m
1H NMR, 500 MHz
CDCl3, 25 °C
1.00
0.86
1.19
1.05
0.66
0.81
0.79
9.5
9.0
8.5
8.0
7.5
7.0
6.5
6.0
5.5
5.0
4.5
4.0
3.5
3.0
2.5
2.0
1.5
1.0
0.5
f1 (ppm)

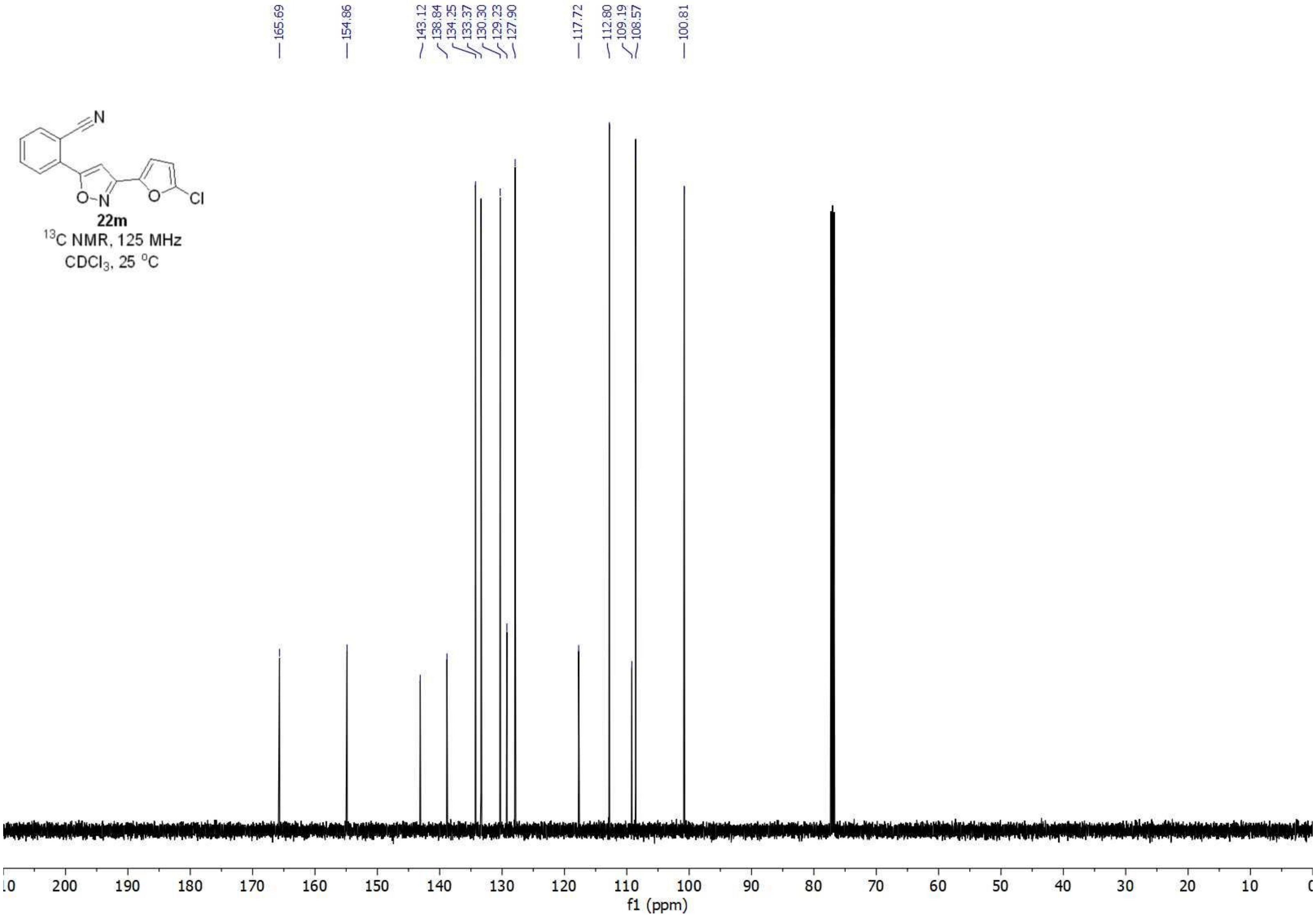
165.69
154.86
143.12
138.84
134.25
133.37
130.30
129.23
127.90
117.72
112.80
109.19
108.57
100.81
N
O
N
O
Cl
22m
^{13}C NMR, 125 MHz
$CDCl_3$, 25 °C
200
190
180
170
160
150
140
130
120
110
100
90
80
70
60
50
40
30
20
10
f1 (ppm)

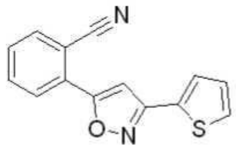

22n

^{1}H NMR, 500 MHz

$CDCl_3$, 25 ^oC

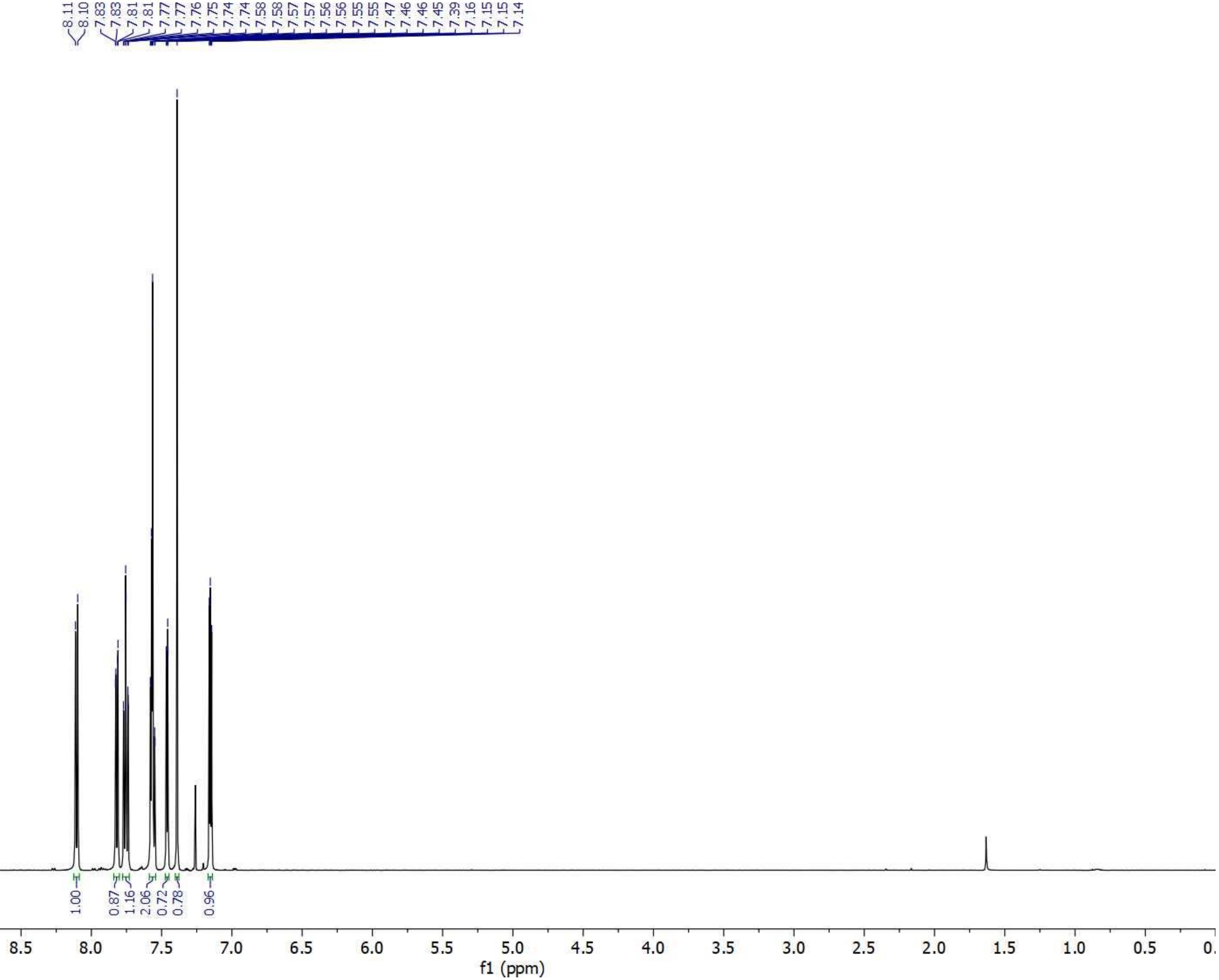

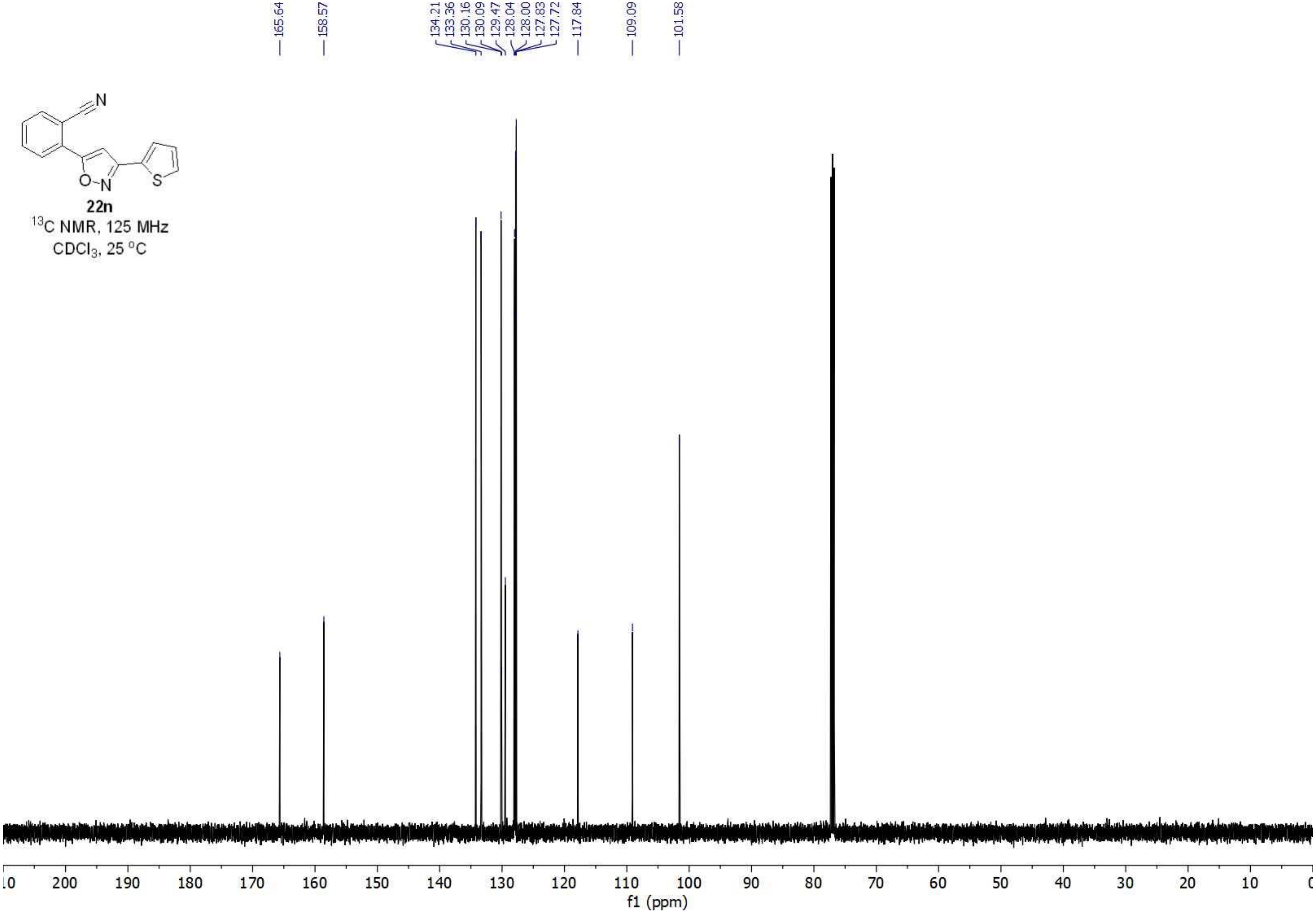
22n
13C NMR, 125 MHz
CDCl3, 25 °C
165.64
158.57
134.21
133.36
130.16
130.09
129.47
128.04
128.00
127.83
127.72
117.84
109.09
101.58
f1 (ppm)
0
200
190
180
170
160
150
140
130
120
110
100
90
80
70
60
50
40
30
20
10
0

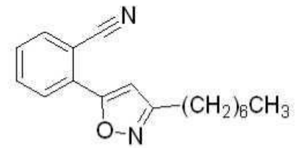

22o

^{1}H NMR, 500 MHz

$CDCl_3$, 25 ^oC

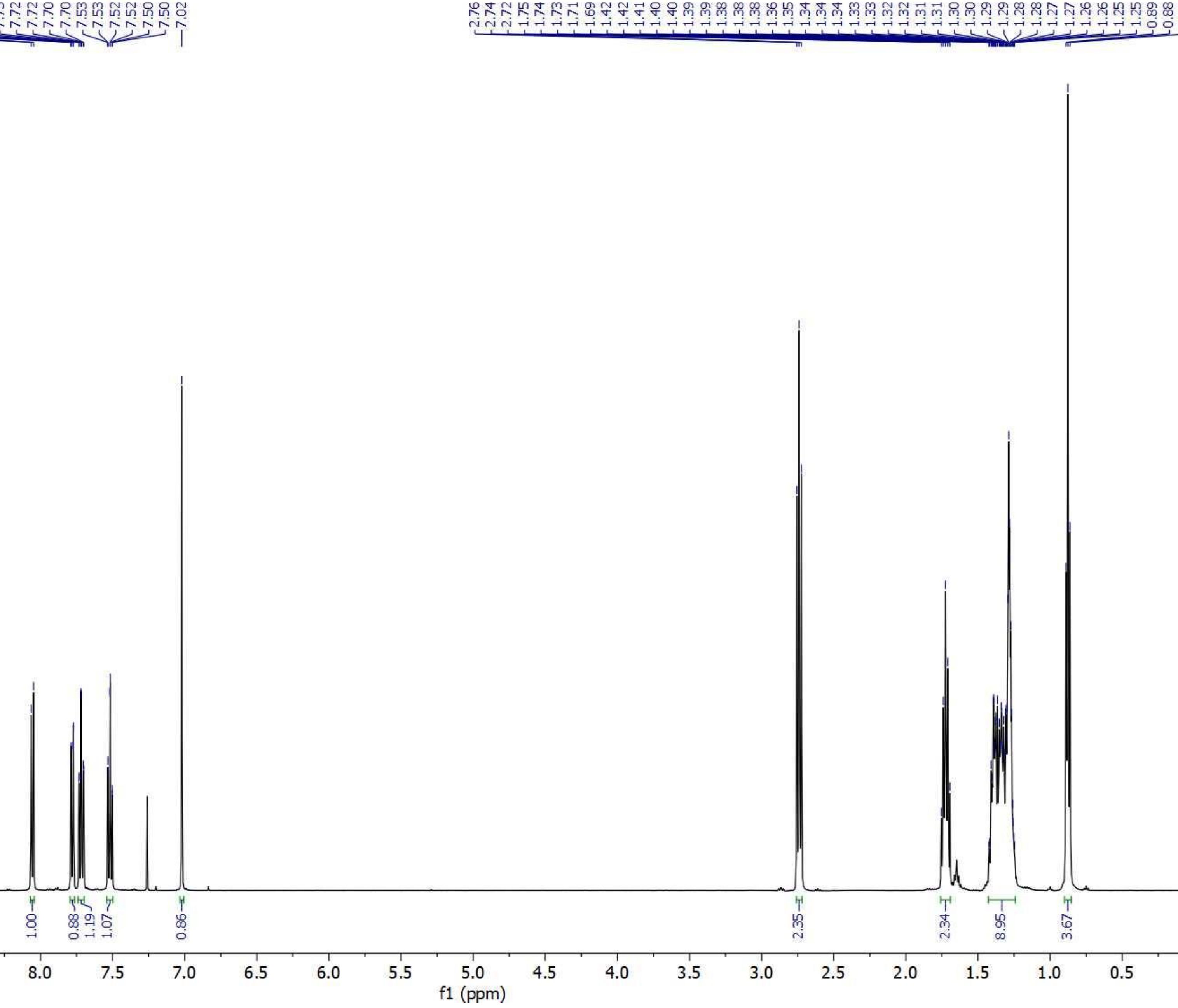

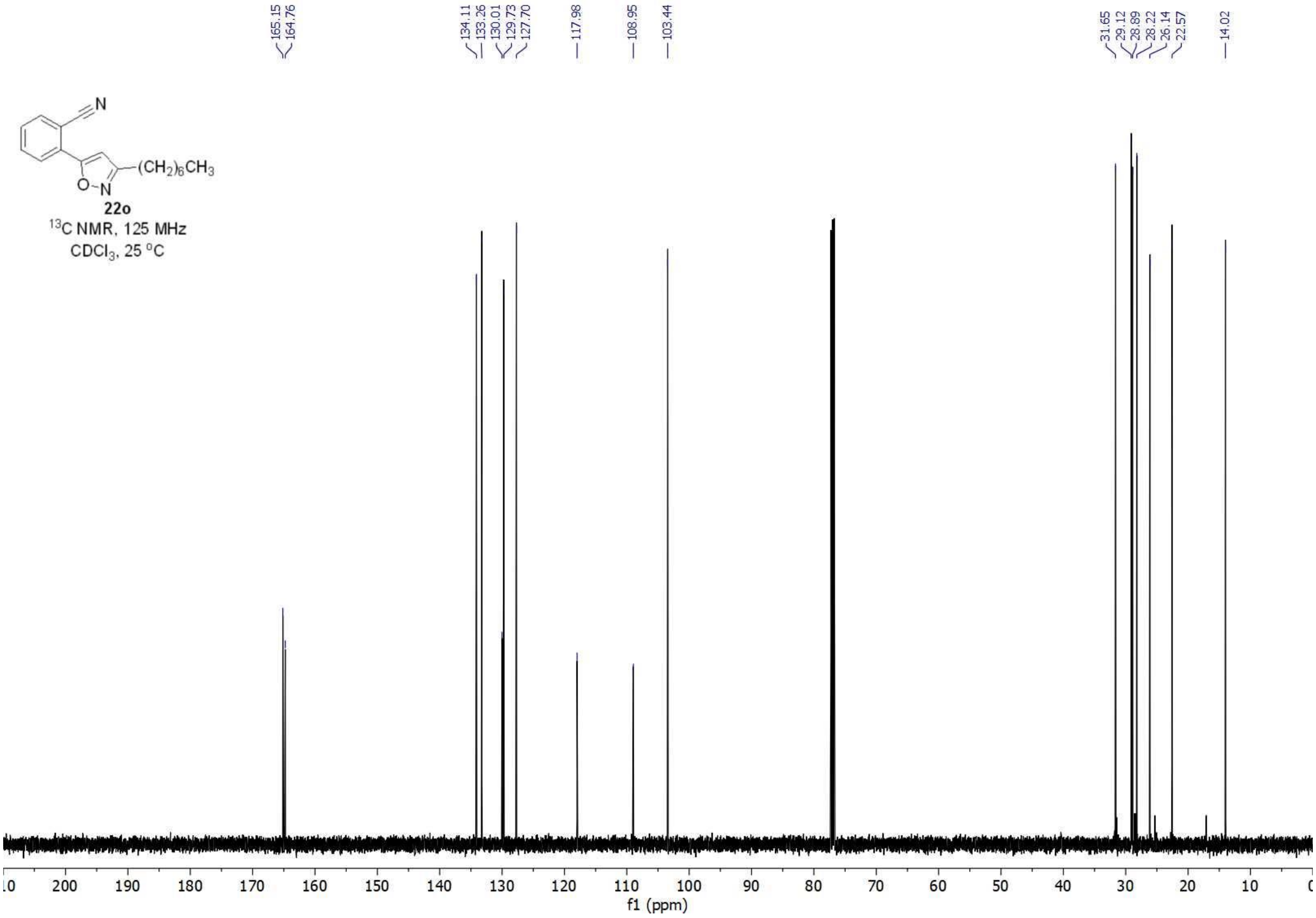
165.15
164.76
134.11
133.26
130.01
129.73
127.70
117.98
108.95
103.44
31.65
29.12
28.89
28.22
26.14
22.57
14.02
N
(CH2)6CH3
O
N
22o
13C NMR, 125 MHz
CDCl3, 25 °C
f1 (ppm)

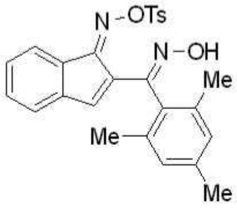

29
^{1}H NMR, 500 MHz
$CDCl_3$, 25 ^{o}C

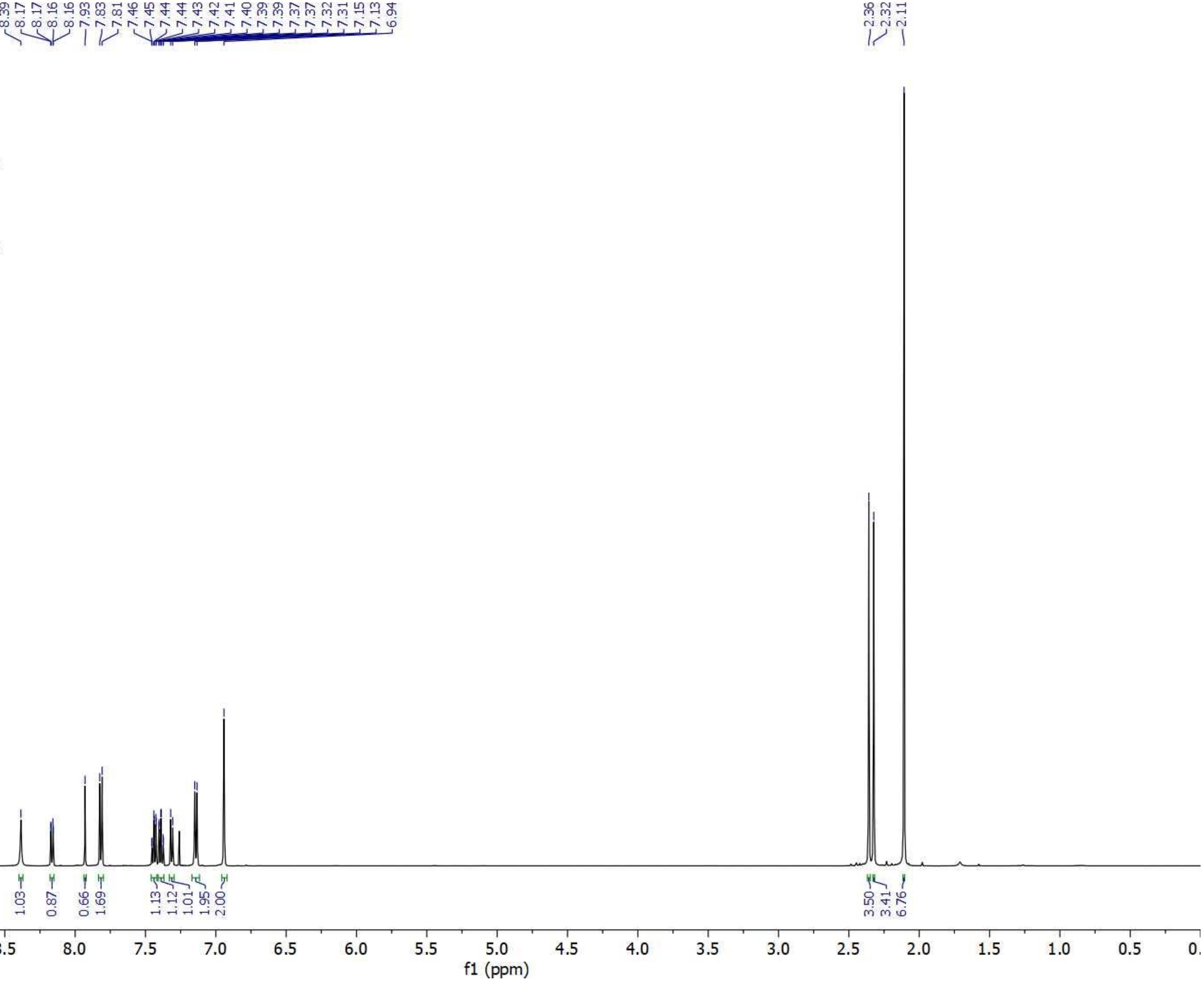

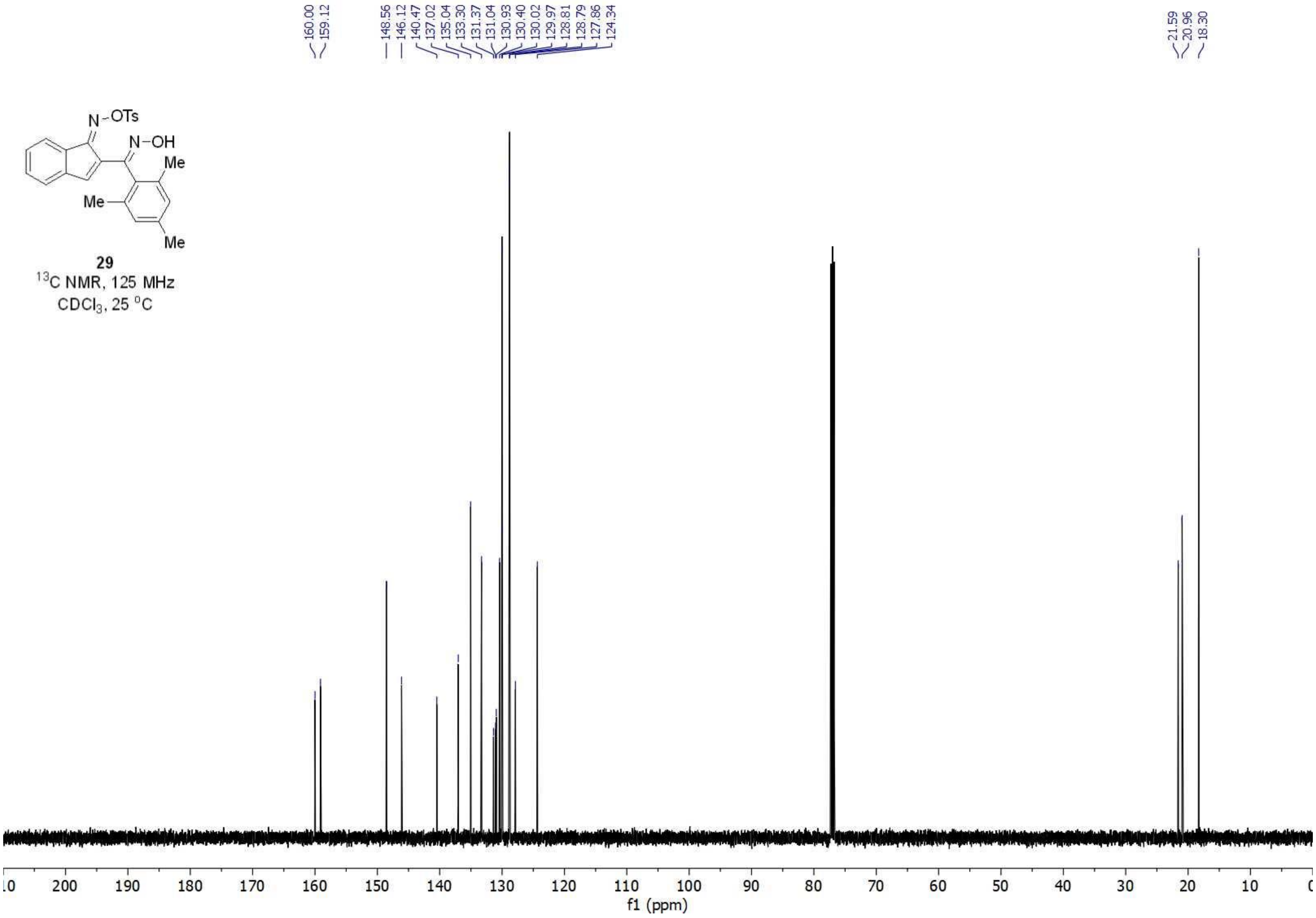
160.00
159.12
148.56
146.12
140.47
137.02
135.04
133.30
131.37
131.04
130.93
130.40
130.02
129.97
128.81
128.79
127.86
124.34
21.59
20.96
18.30
N-OTs
N-OH
Me
Me
Me
29
13C NMR, 125 MHz
CDCl3, 25 °C
f1 (ppm)

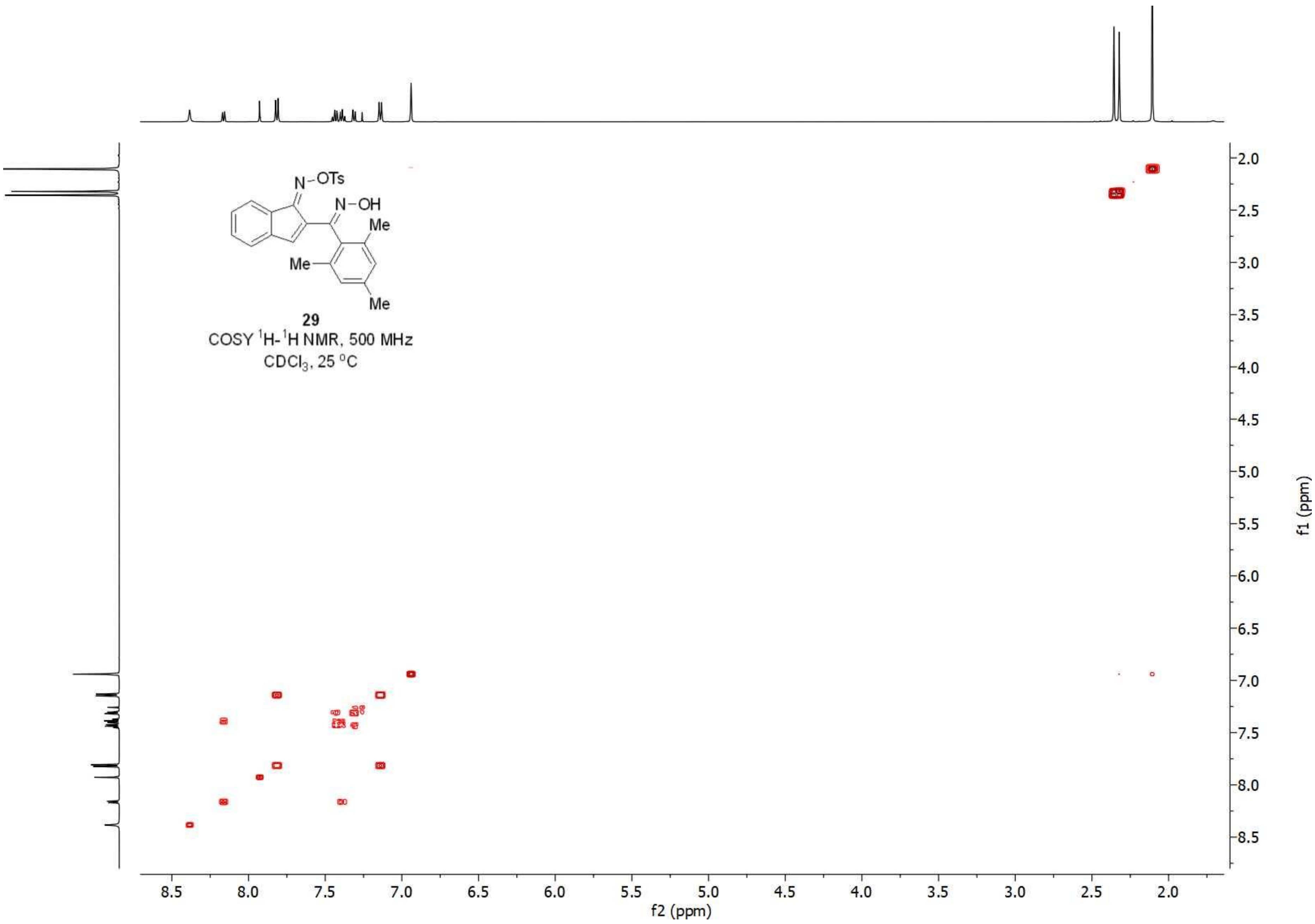

N-OTs
N-OH
Me
Me
Me
29
COSY ¹H-¹H NMR, 500 MHz
CDCl₃, 25 °C
f1 (ppm)
f2 (ppm)

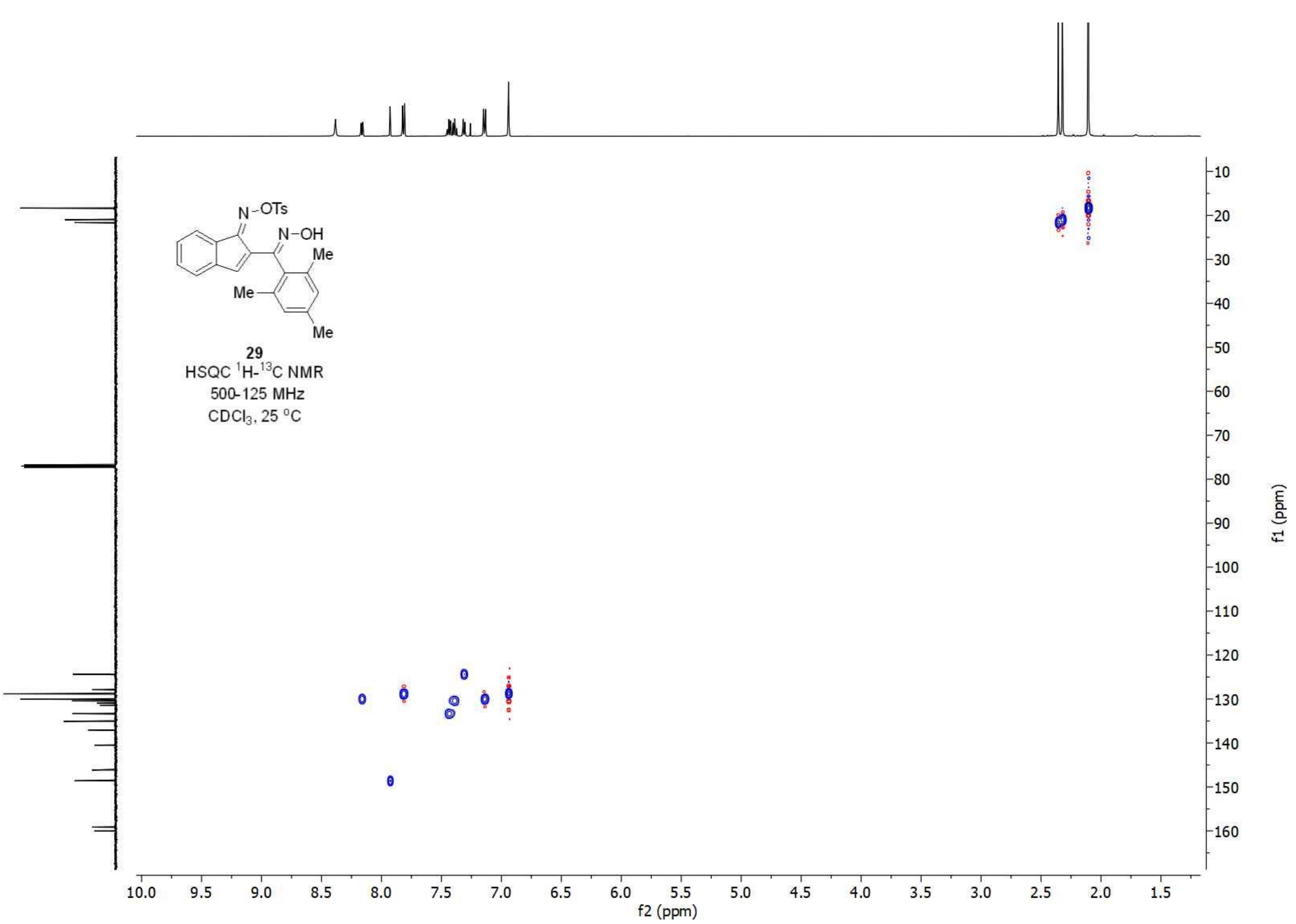
N-OTs
N-OH
Me
Me
Me
29
HSQC ¹H-¹³C NMR
500-125 MHz
CDCl₃, 25 °C
f1 (ppm)
f2 (ppm)

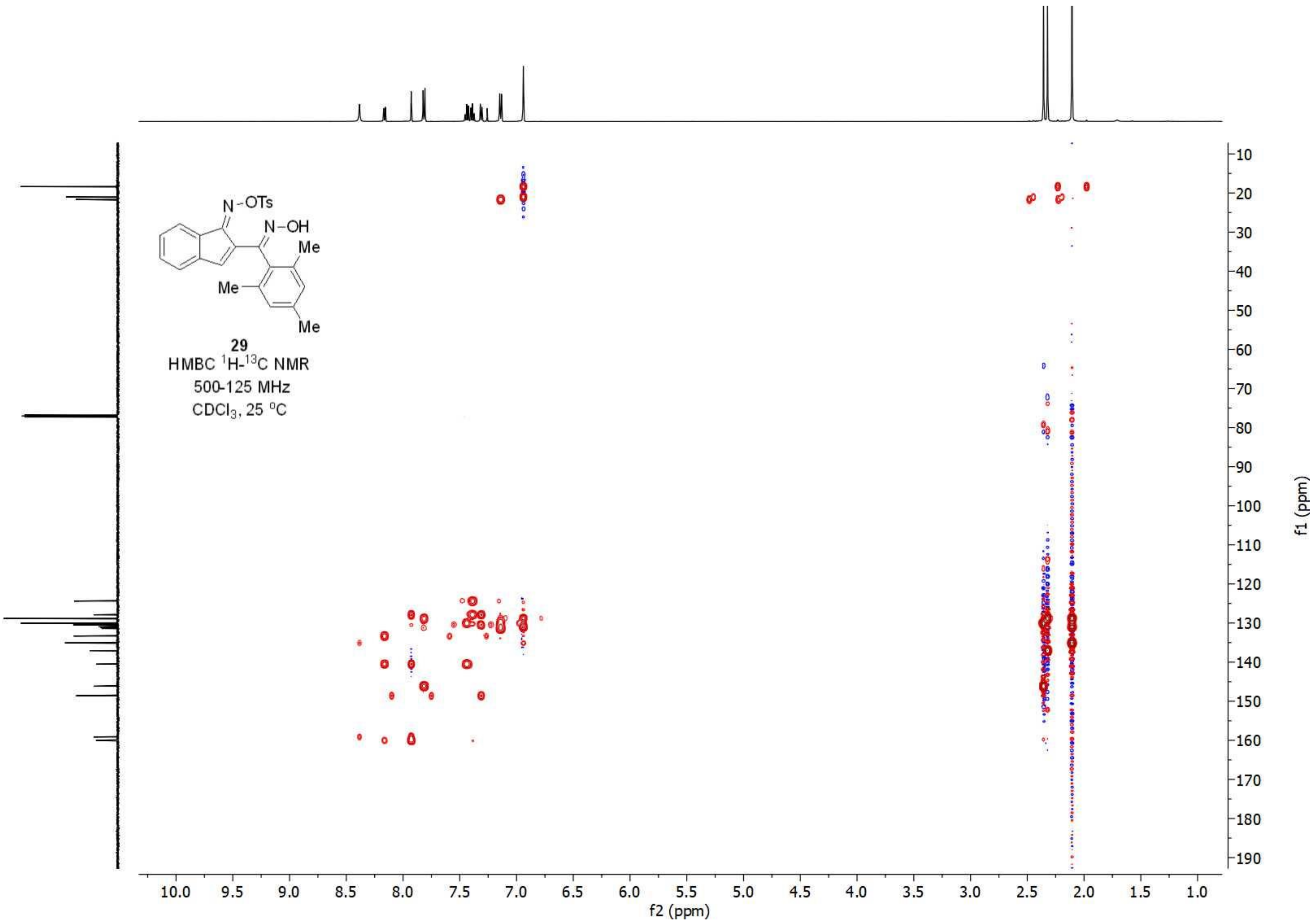
N-OTs
N-OH
Me
Me
Me
29
HMBC ^{1}H-^{13}C NMR
500-125 MHz
$CDCl_3$, 25 °C
f2 (ppm)
f1 (ppm)

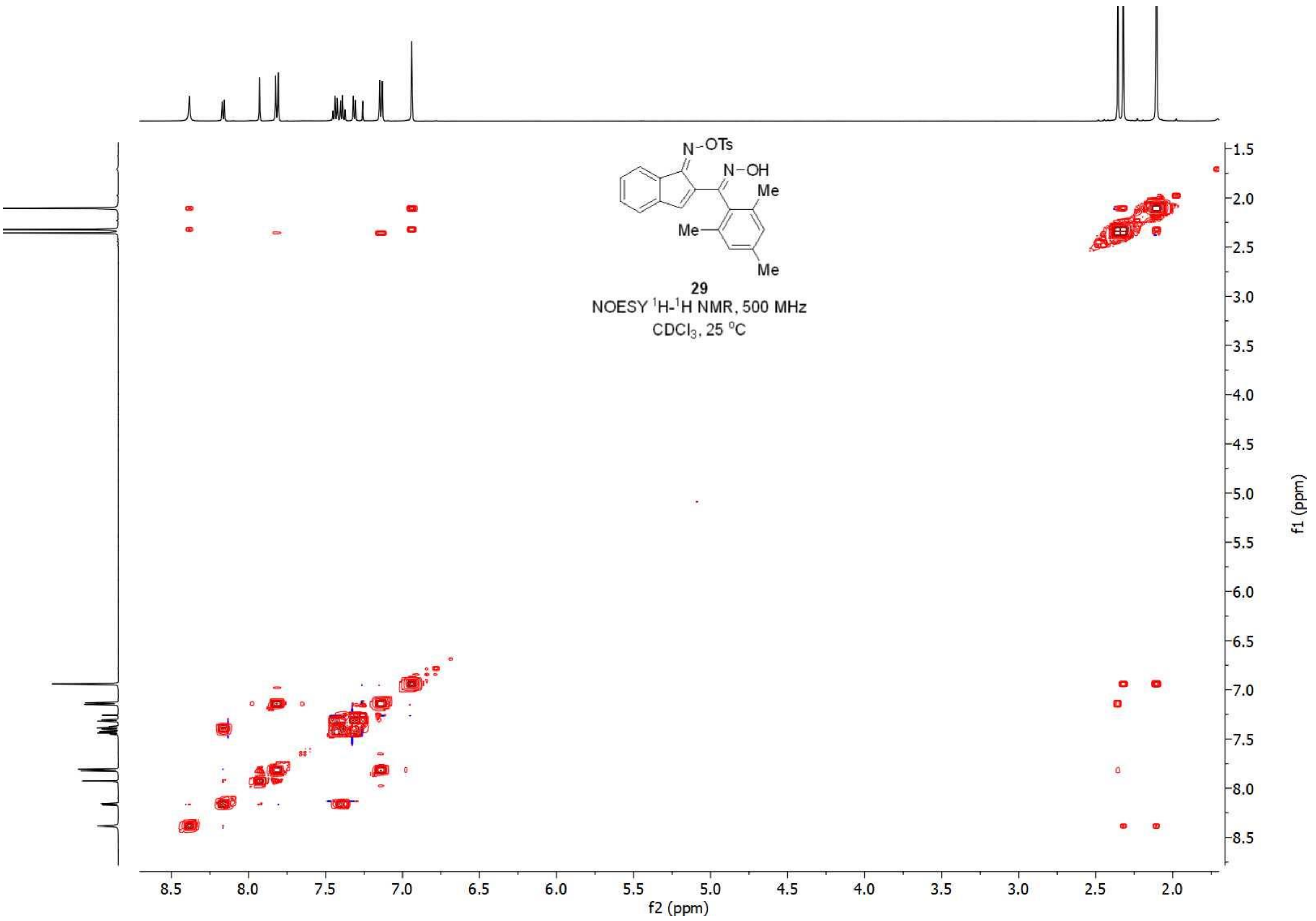
N-OTs
N-OH
Me
Me
Me
29
NOESY ¹H-¹H NMR, 500 MHz
CDCl3, 25 °C
f1 (ppm)
f2 (ppm)

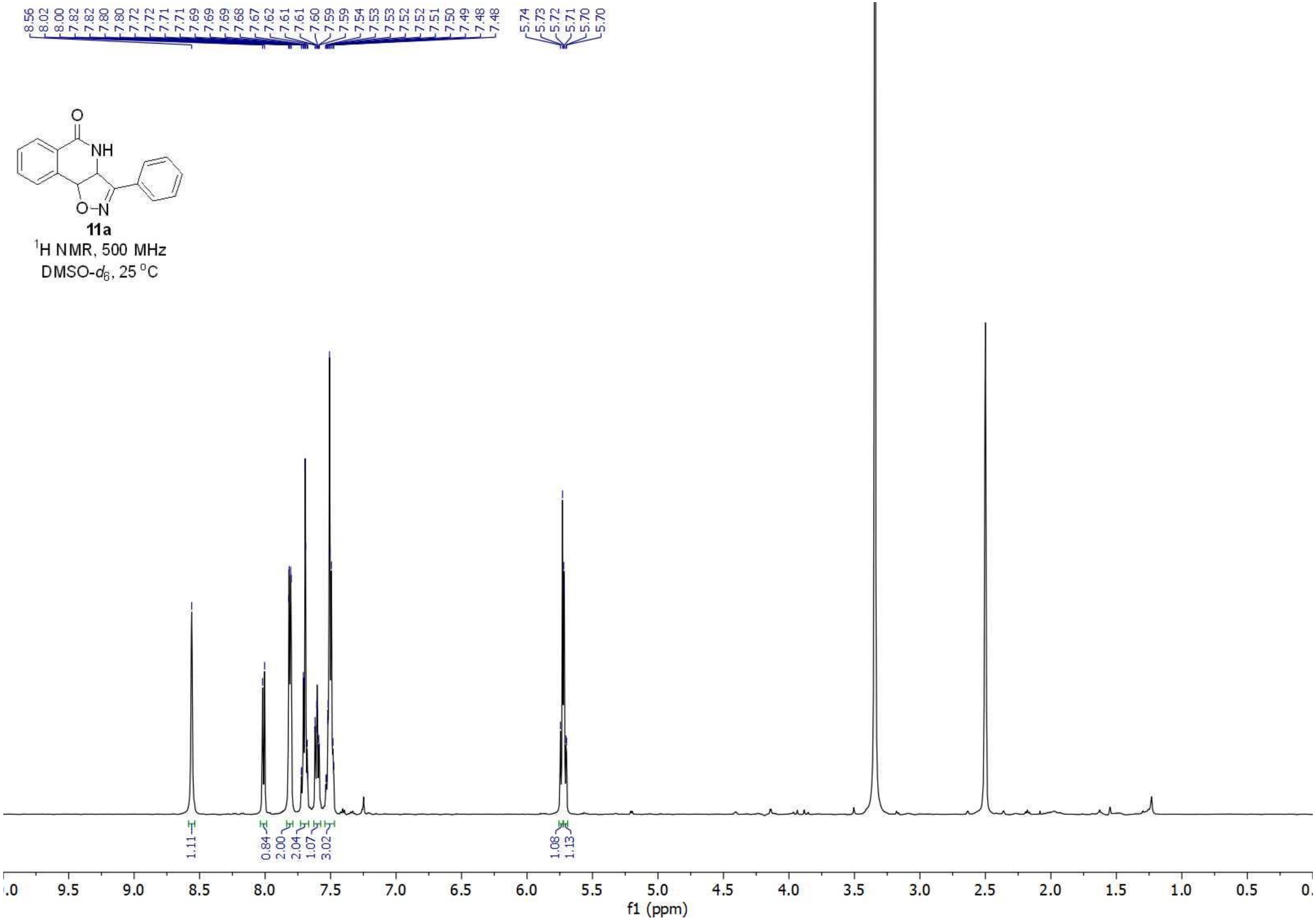
8.56 8.02 8.00 7.82 7.82 7.80 7.80 7.72 7.72 7.71 7.71 7.69 7.69 7.69 7.68 7.67 7.62 7.61 7.61 7.60 7.59 7.59 7.54 7.53 7.53 7.52 7.52 7.51 7.50 7.49 7.48 7.48
5.74 5.73 5.72 5.71 5.70 5.70
O
NH
O–N
11a
^{1}H NMR, 500 MHz
DMSO-d_6, 25 °C
1.11
0.84 2.00 2.04 1.07 3.02
1.08 1.13
f1 (ppm)

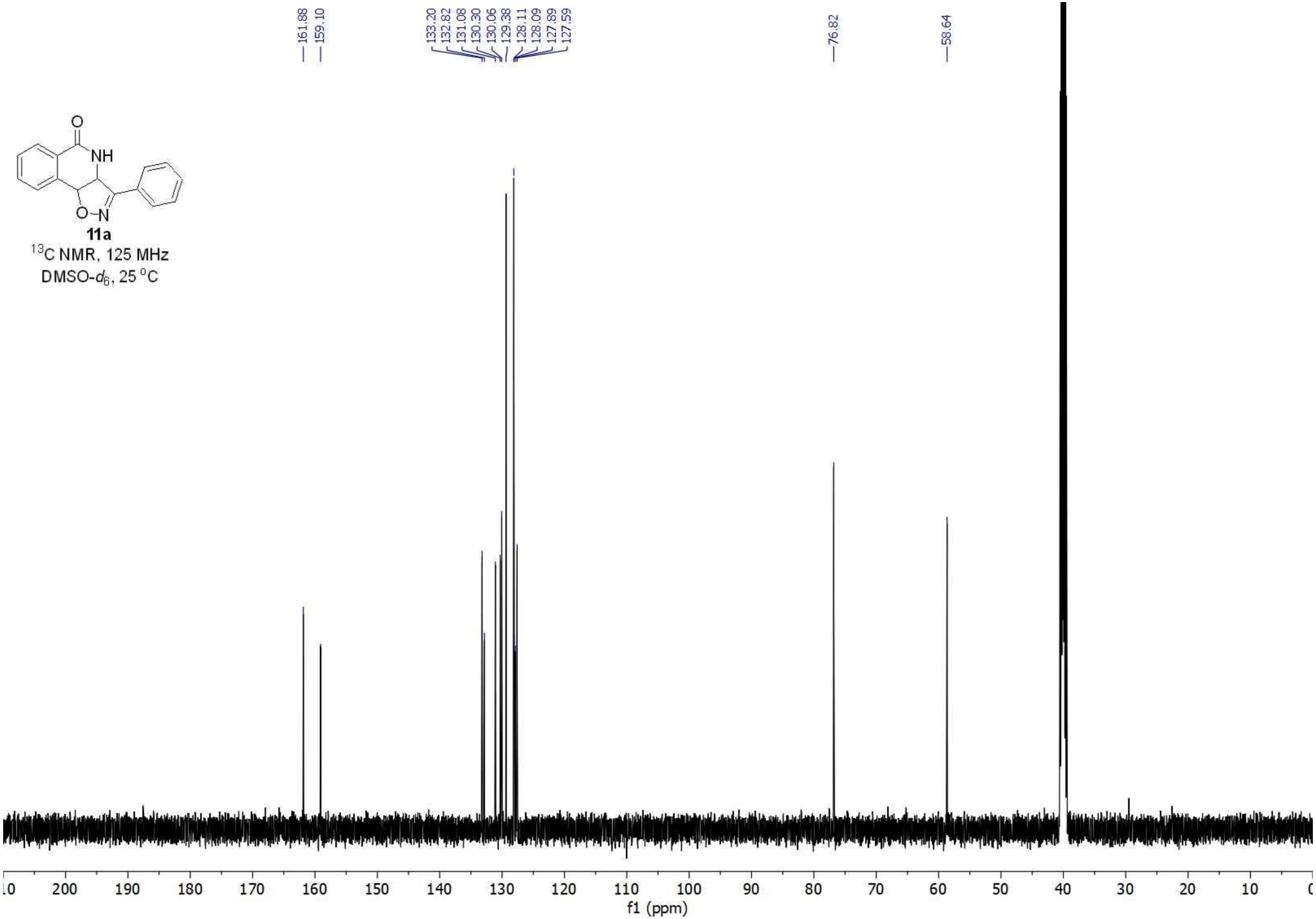
161.88
159.10
133.20
132.82
131.08
130.30
130.06
129.38
128.11
128.09
127.89
127.59
76.82
58.64
O
NH
O-N
11a
13C NMR, 125 MHz
DMSO-d6, 25 °C
200
190
180
170
160
150
140
130
120
110
100
90
80
70
60
50
40
30
20
10
f1 (ppm)

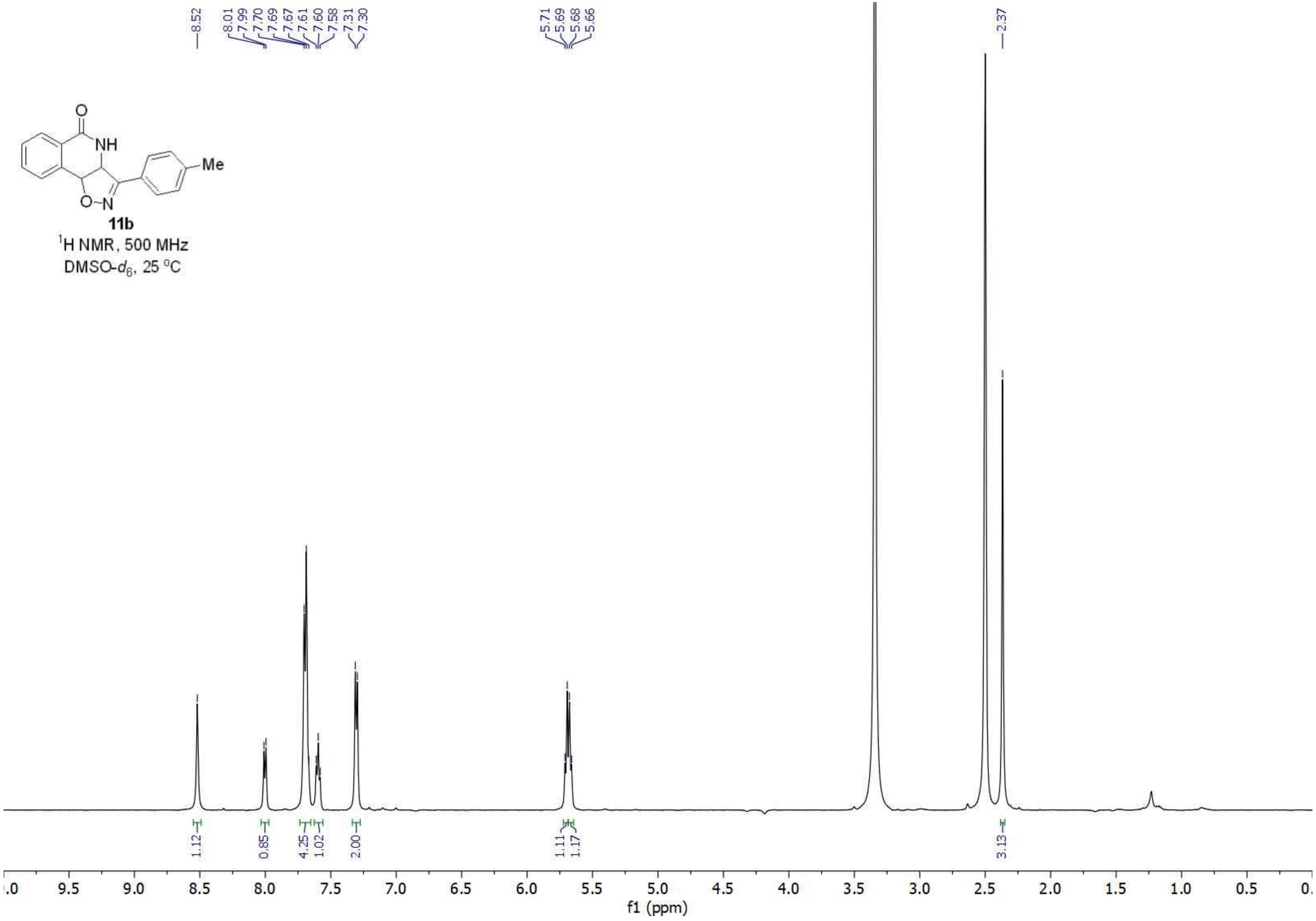

11b
^{1}H NMR, 500 MHz
DMSO-d_6, 25 °C
8.52
8.01 7.99 7.70 7.69 7.67 7.61 7.60 7.58 7.31 7.30
5.71 5.69 5.68 5.66
2.37
1.12 0.85 4.25 1.02 2.00 1.11 1.17 3.13
f1 (ppm)

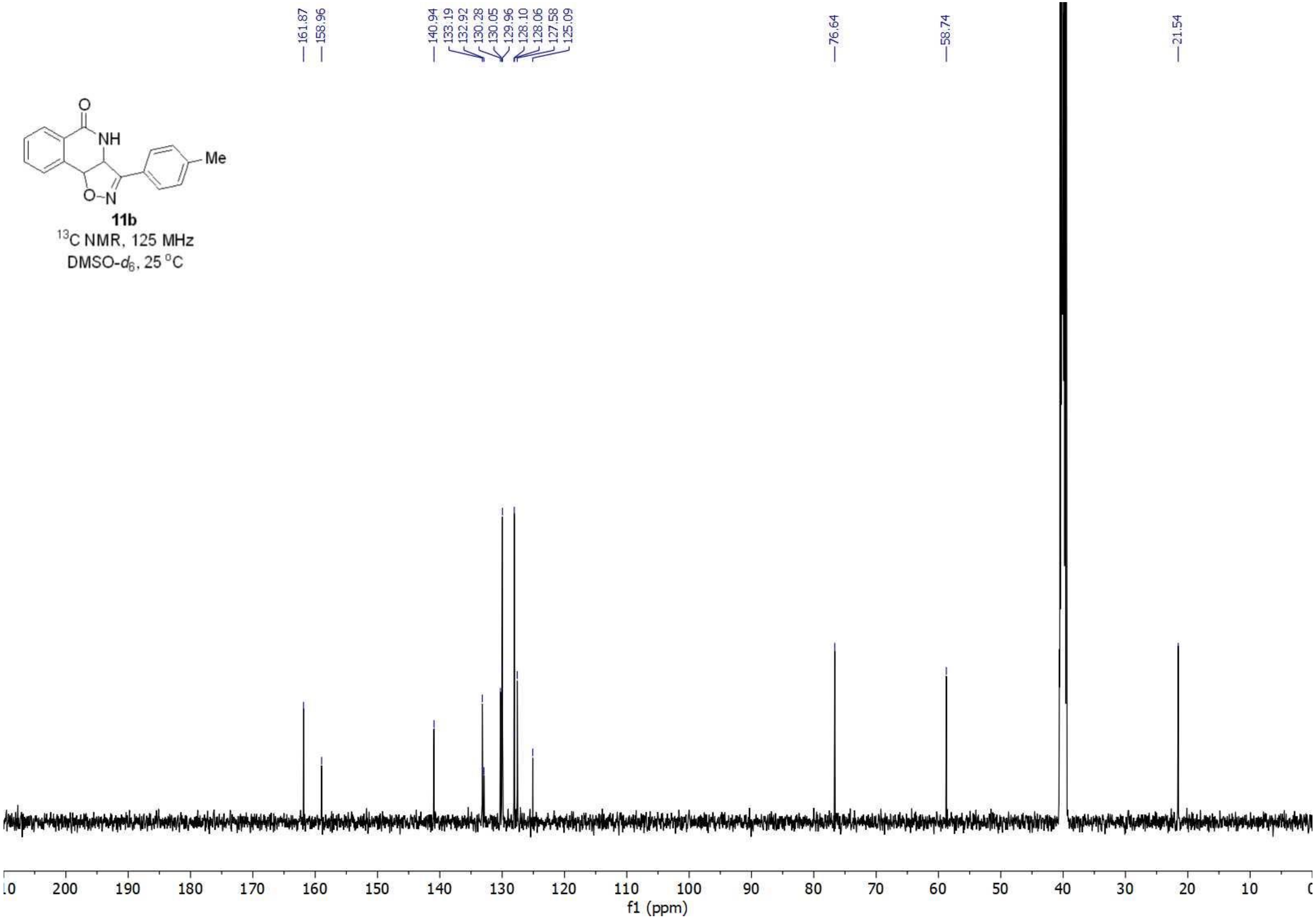
161.87
158.96
140.94
133.19
132.92
130.28
130.05
129.96
128.10
128.06
127.58
125.09
76.64
58.74
21.54
O
NH
Me
O–N
11b
13C NMR, 125 MHz
DMSO-d6, 25 °C
200
190
180
170
160
150
140
130
120
110
100
90
80
70
60
50
40
30
20
10
f1 (ppm)

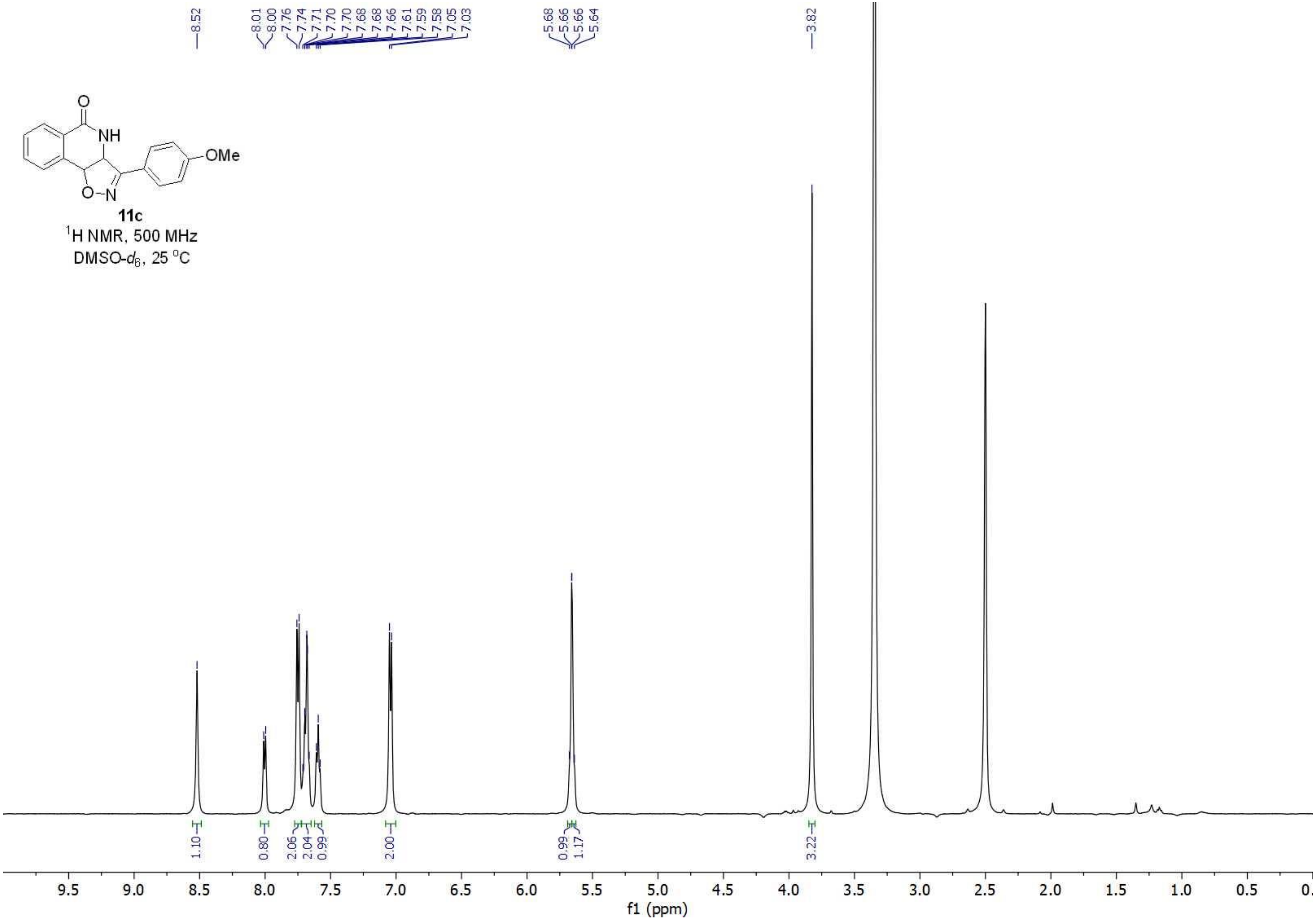
8.52
8.01
8.00
7.76
7.74
7.71
7.70
7.68
7.68
7.66
7.61
7.59
7.58
7.05
7.03
5.68
5.66
5.66
5.64
3.82
O
NH
OMe
O
N
11c
¹H NMR, 500 MHz
DMSO-d6, 25 °C
1.10
0.80
2.06
2.04
0.99
2.00
0.99
1.17
3.22
9.5
9.0
8.5
8.0
7.5
7.0
6.5
6.0
5.5
5.0
4.5
4.0
3.5
3.0
2.5
2.0
1.5
1.0
0.5
f1 (ppm)

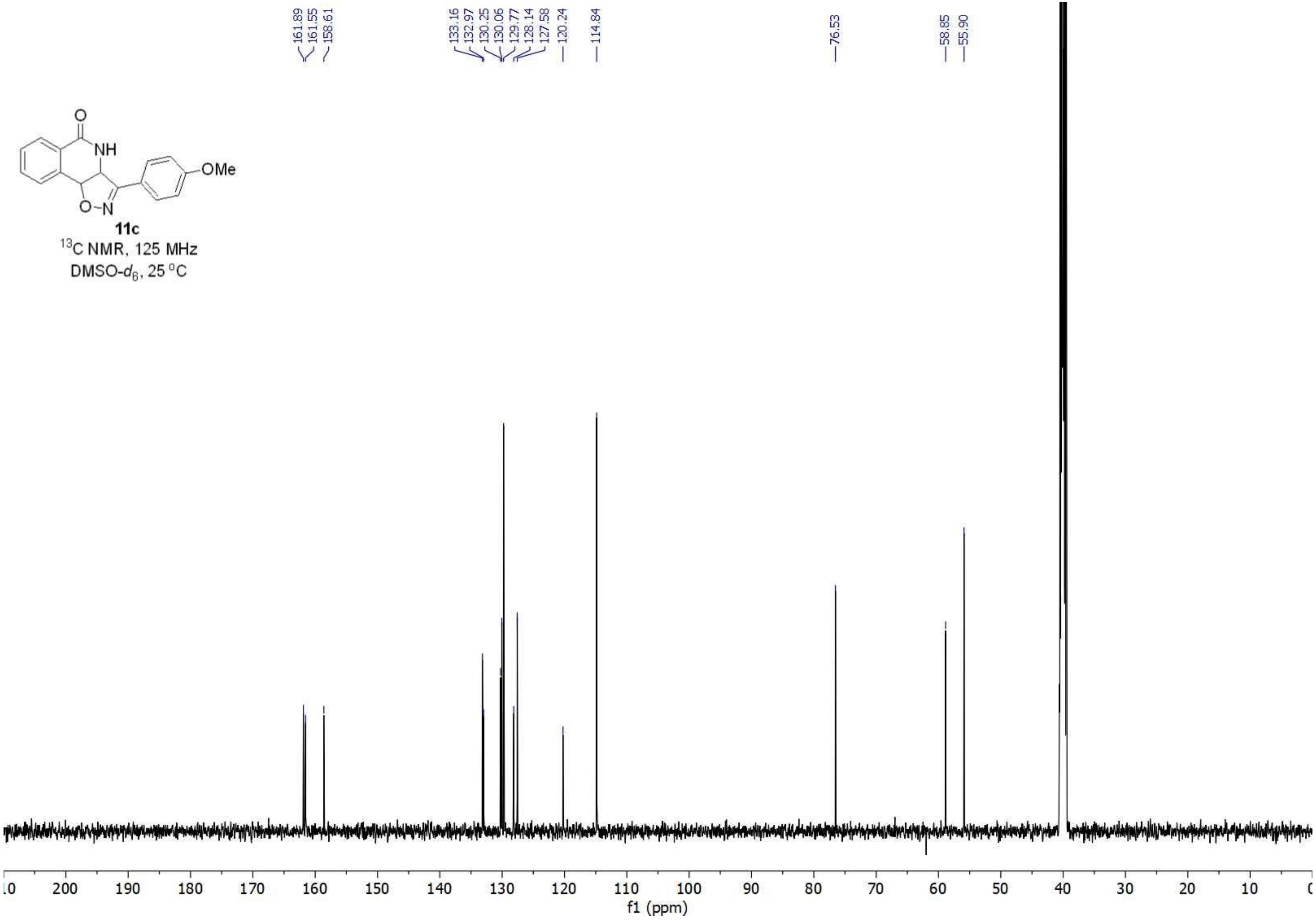

161.89
161.55
158.61
133.16
132.97
130.25
130.06
129.77
128.14
127.58
120.24
114.84
76.53
58.85
55.90
OMe
NH
O
O–N
11c
^{13}C NMR, 125 MHz
DMSO-d_6, 25 °C
f1 (ppm)

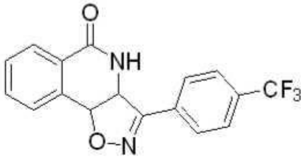

11d
^{1}H NMR, 500 MHz
DMSO-d_6, 25 ^{o}C

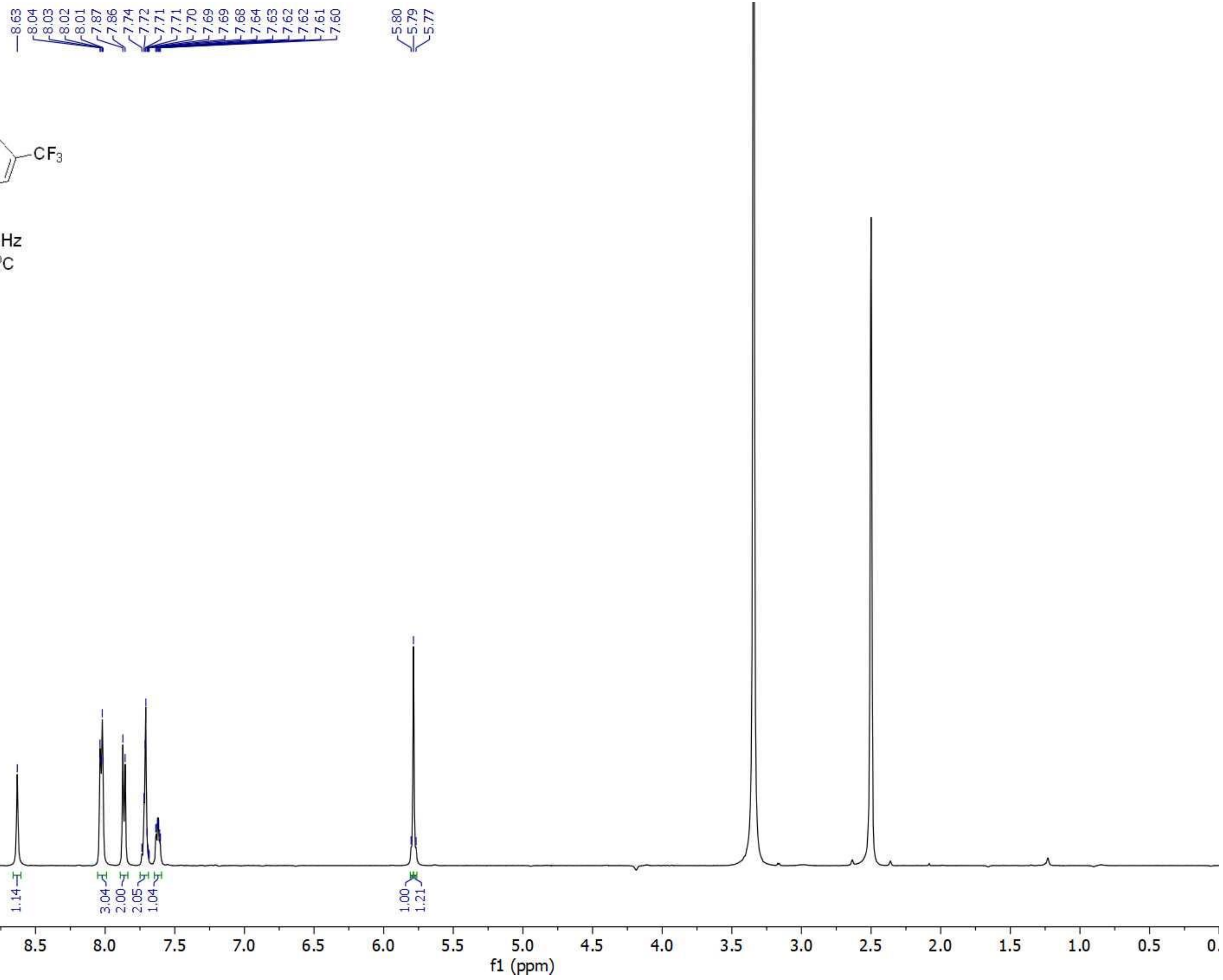

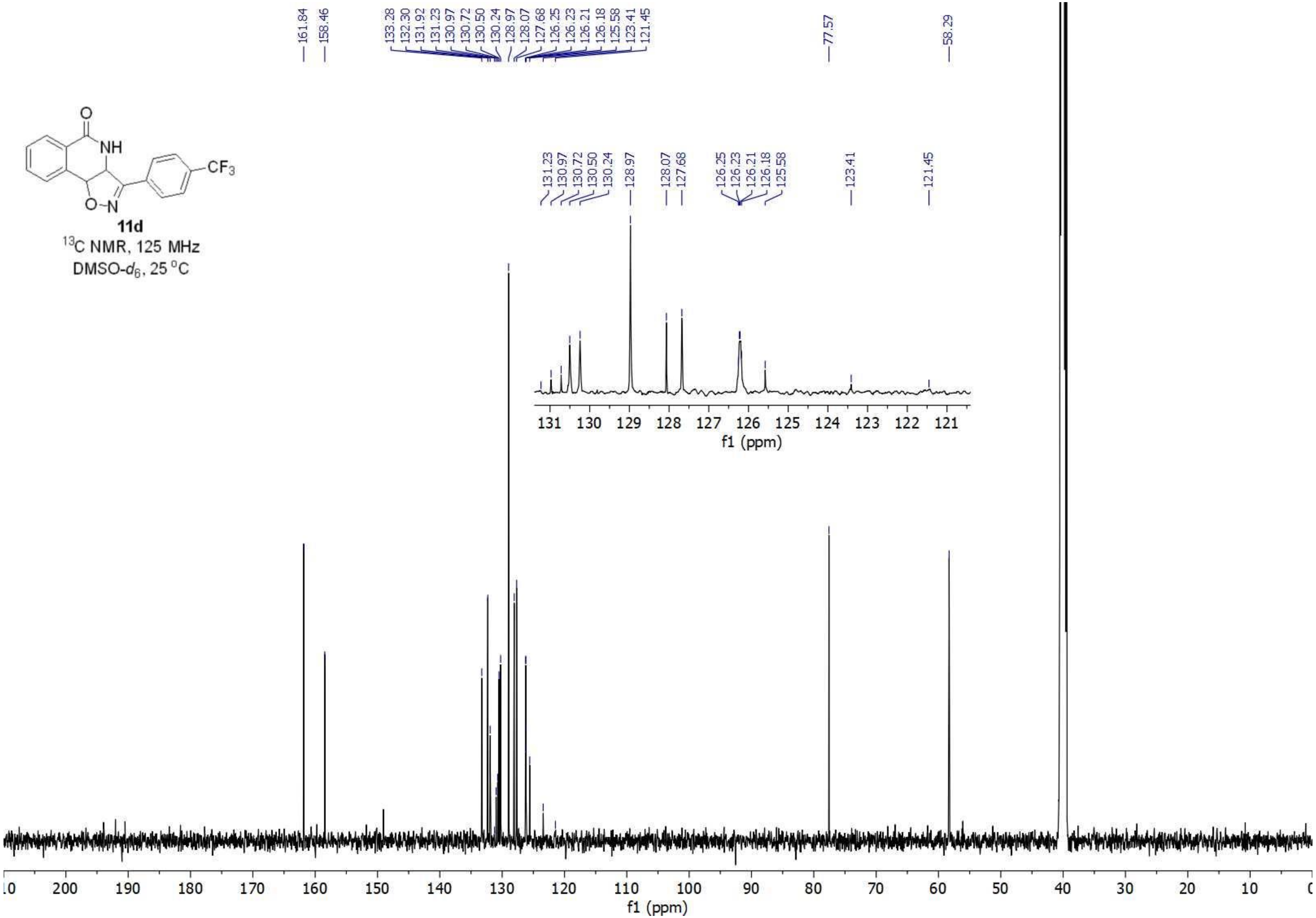
161.84
158.46
133.28
132.30
131.92
131.23
130.97
130.72
130.50
130.24
128.97
128.07
127.68
126.25
126.23
126.21
126.18
125.58
123.41
121.45
77.57
58.29
f1 (ppm)
11d
^{13}C NMR, 125 MHz
DMSO-d_6, 25 °C

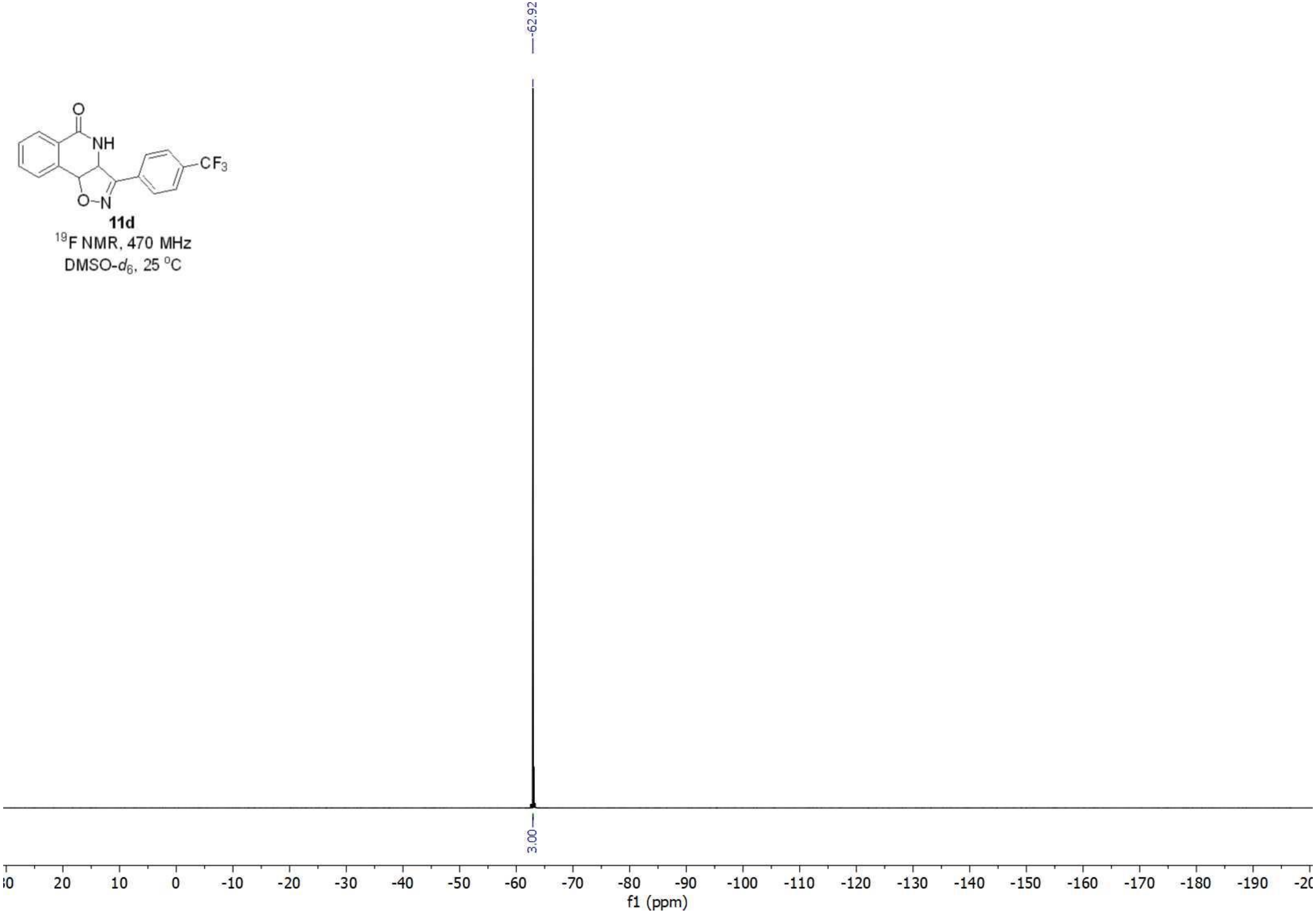

-62.92
11d
^{19}F NMR, 470 MHz
DMSO-d_6, 25 ^{o}C
3.00
20 10 0 -10 -20 -30 -40 -50 -60 -70 -80 -90 -100 -110 -120 -130 -140 -150 -160 -170 -180 -190
f1 (ppm)

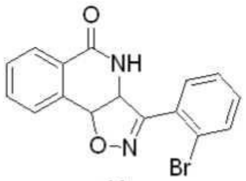

11g
^{1}H NMR, 500 MHz
DMSO-d_6, 25 °C

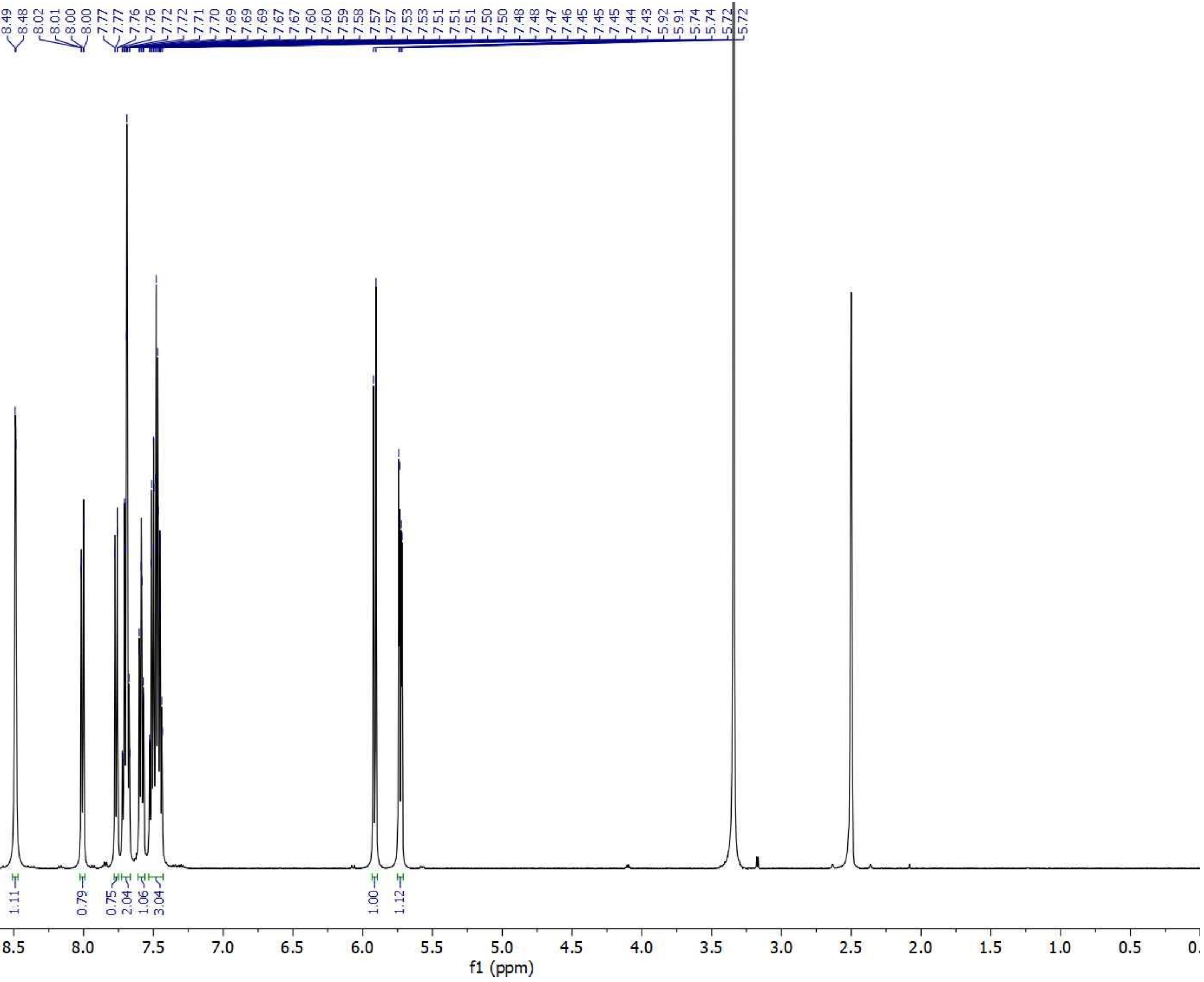

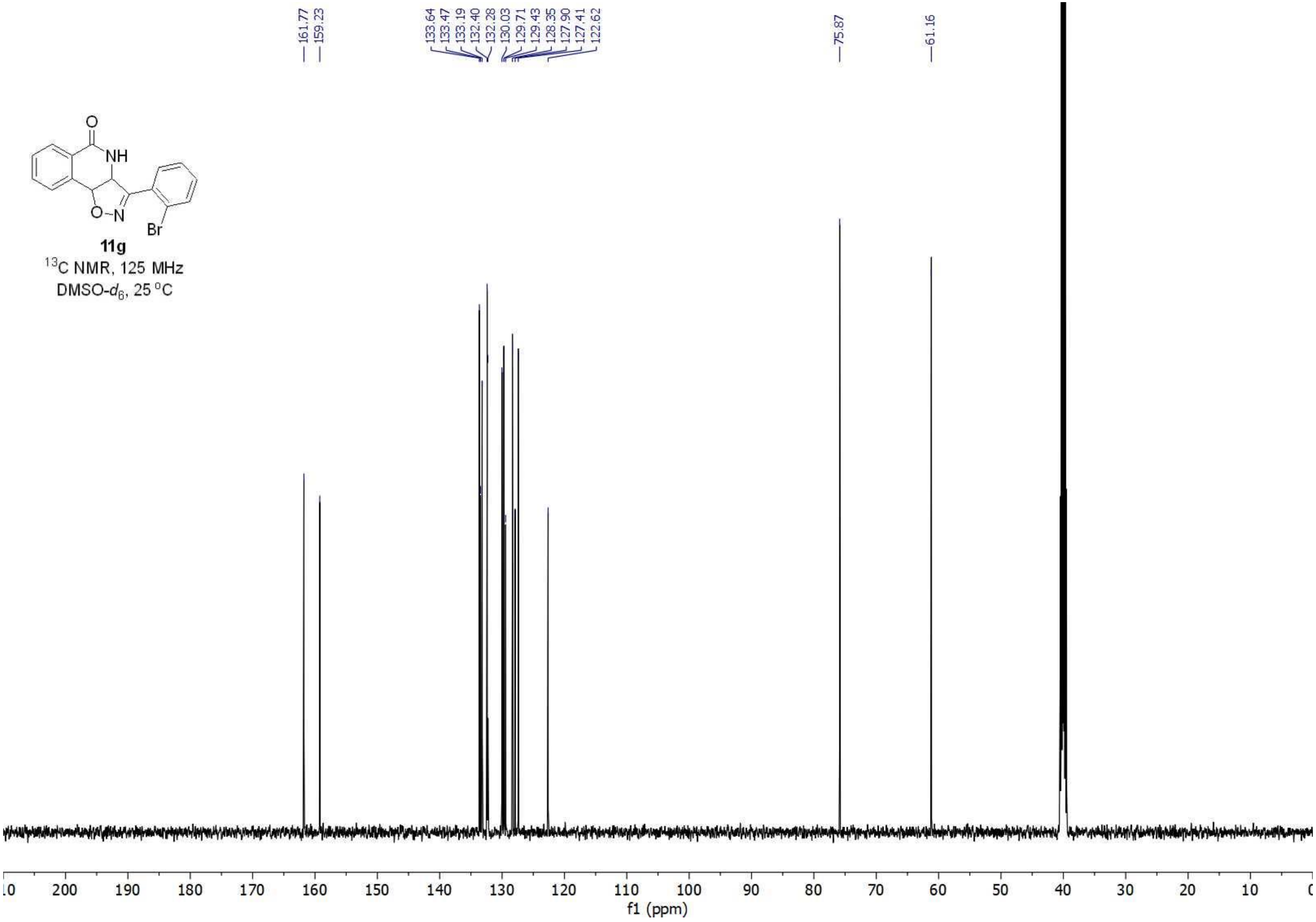

11g
^{13}C NMR, 125 MHz
DMSO-d_6, 25 °C

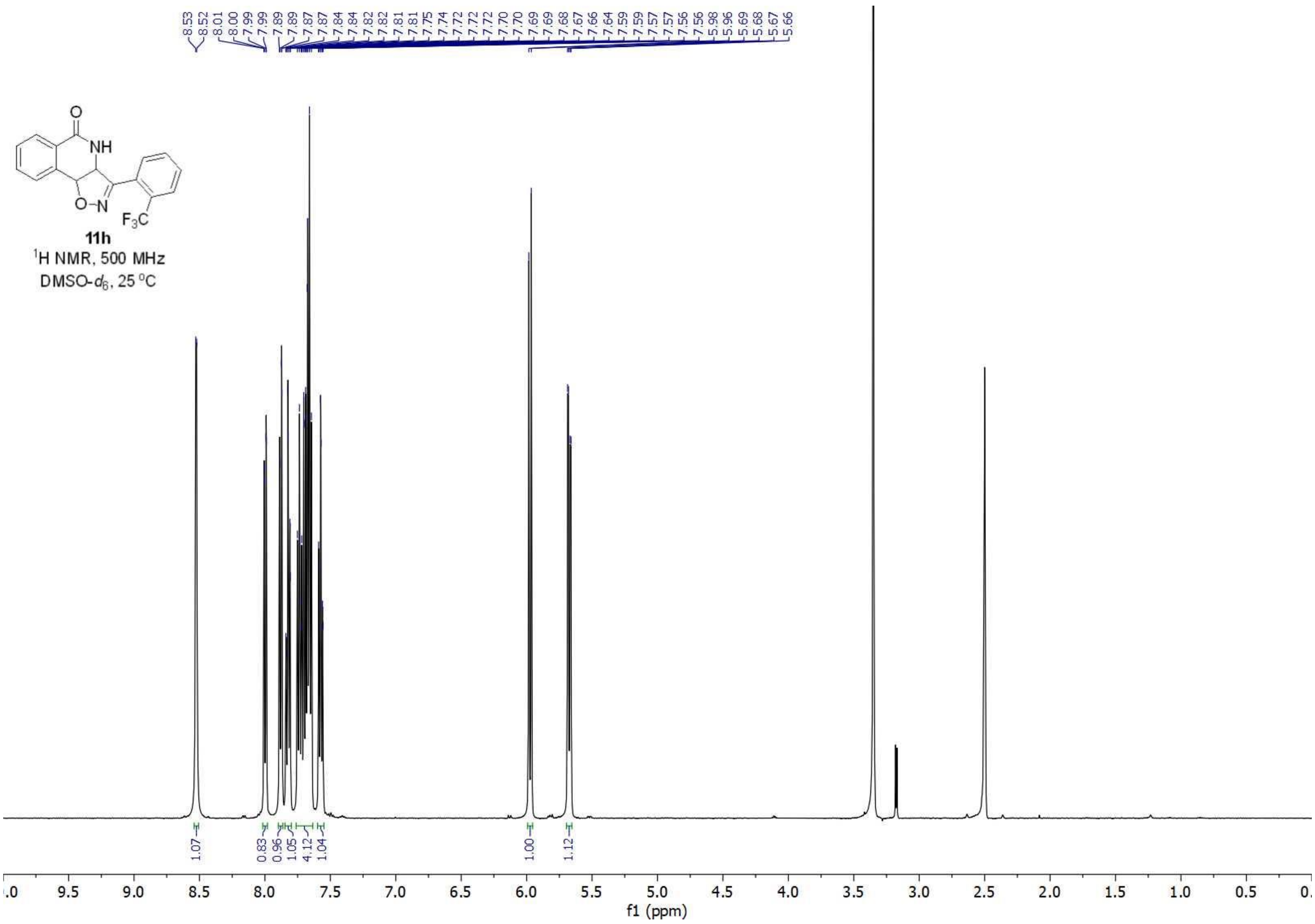
8.53
8.52
8.01
8.00
7.99
7.99
7.89
7.89
7.87
7.87
7.84
7.84
7.82
7.82
7.81
7.81
7.75
7.74
7.72
7.72
7.72
7.70
7.70
7.69
7.69
7.68
7.67
7.66
7.64
7.59
7.59
7.57
7.57
7.56
7.56
5.98
5.96
5.69
5.68
5.67
5.66
O
NH
O–N
F3C
11h
1H NMR, 500 MHz
DMSO-d6, 25 °C
1.07
0.83
0.96
1.05
4.12
1.04
1.00
1.12
9.5
9.0
8.5
8.0
7.5
7.0
6.5
6.0
5.5
5.0
4.5
4.0
3.5
3.0
2.5
2.0
1.5
1.0
0.5
f1 (ppm)

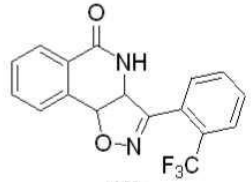

11h
^{13}C NMR, 125 MHz
DMSO-d_6, 25 °C

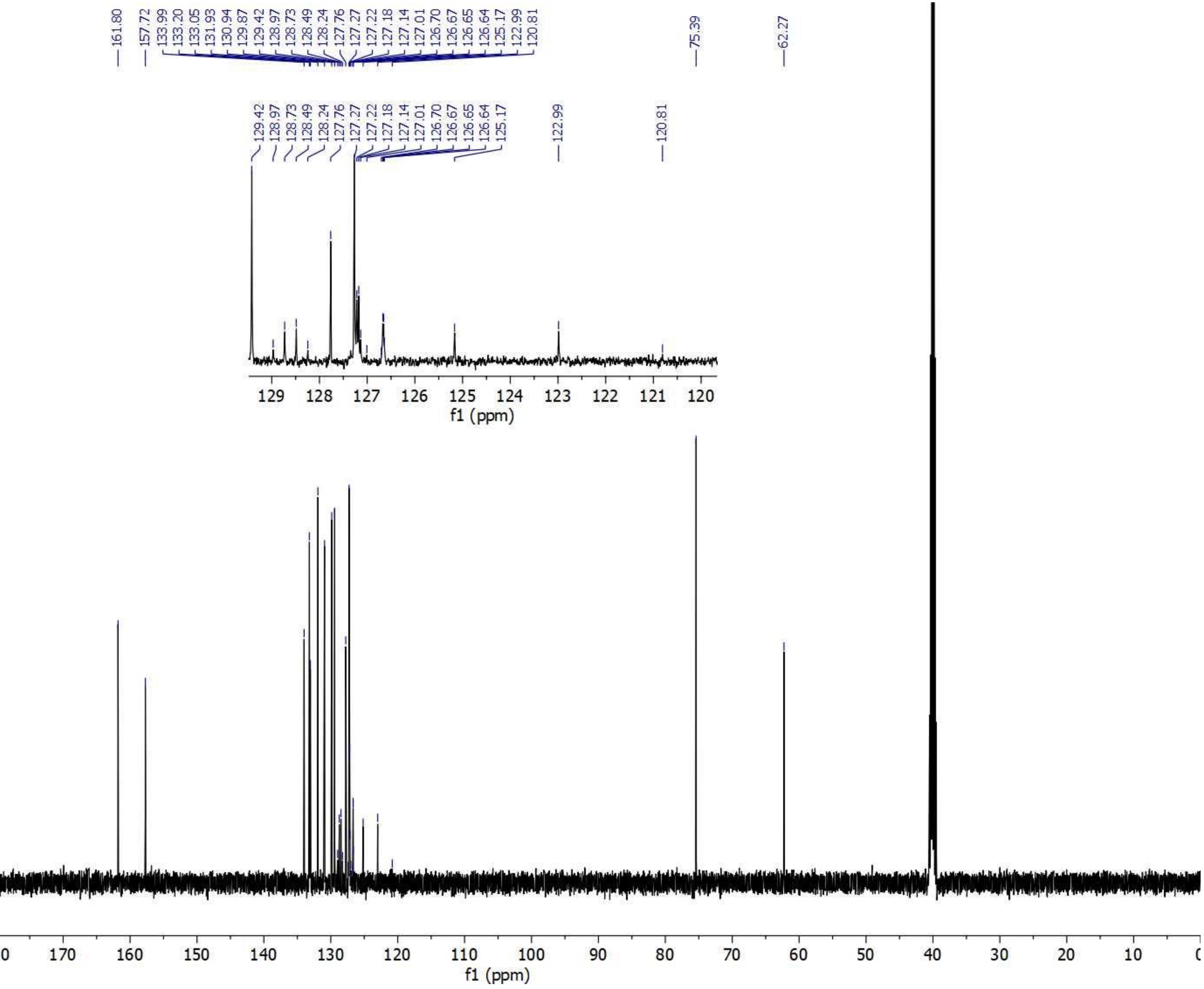

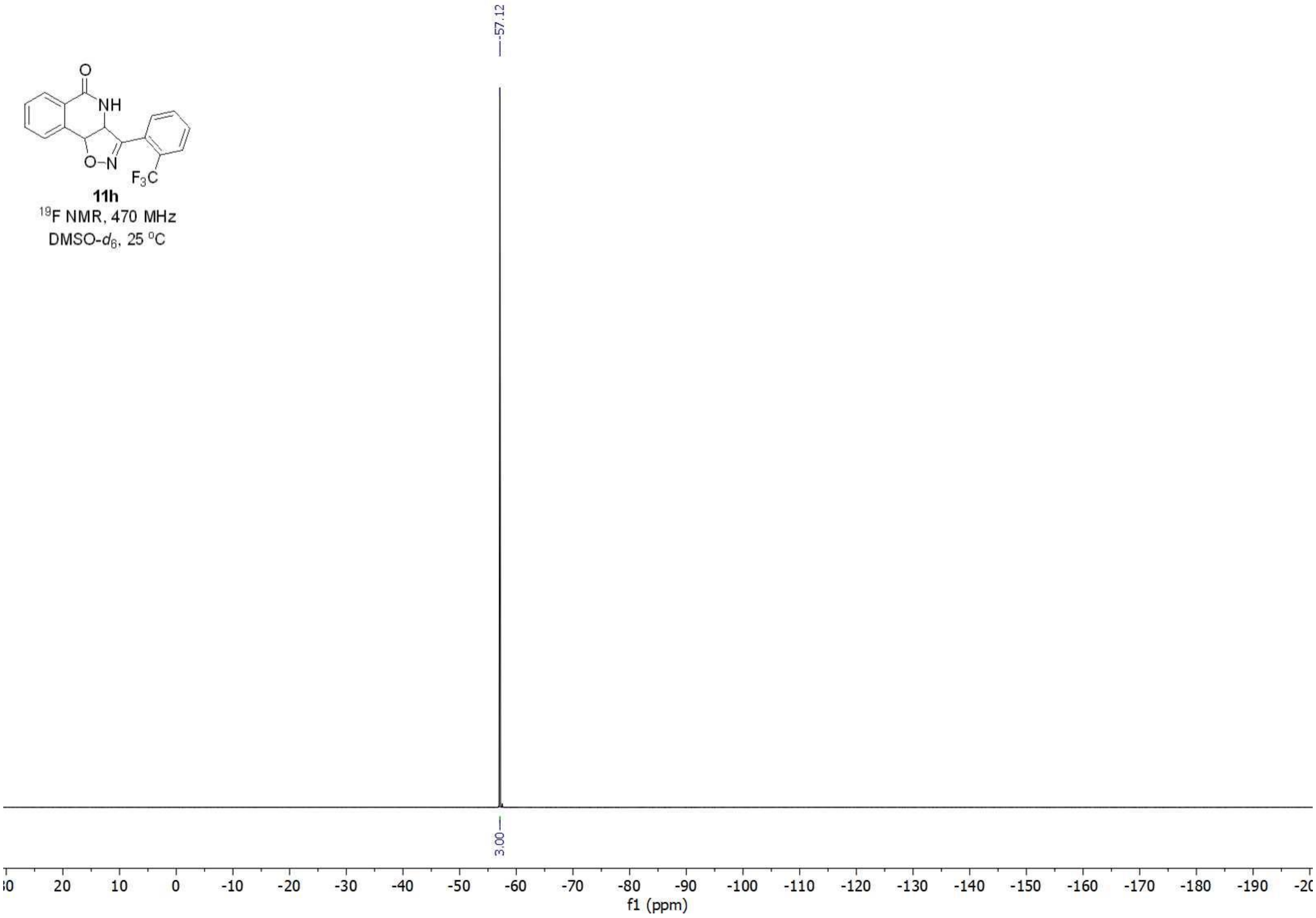

-57.12
11h
19F NMR, 470 MHz
DMSO-d6, 25 °C
3.00
20 10 0 -10 -20 -30 -40 -50 -60 -70 -80 -90 -100 -110 -120 -130 -140 -150 -160 -170 -180 -190
f1 (ppm)

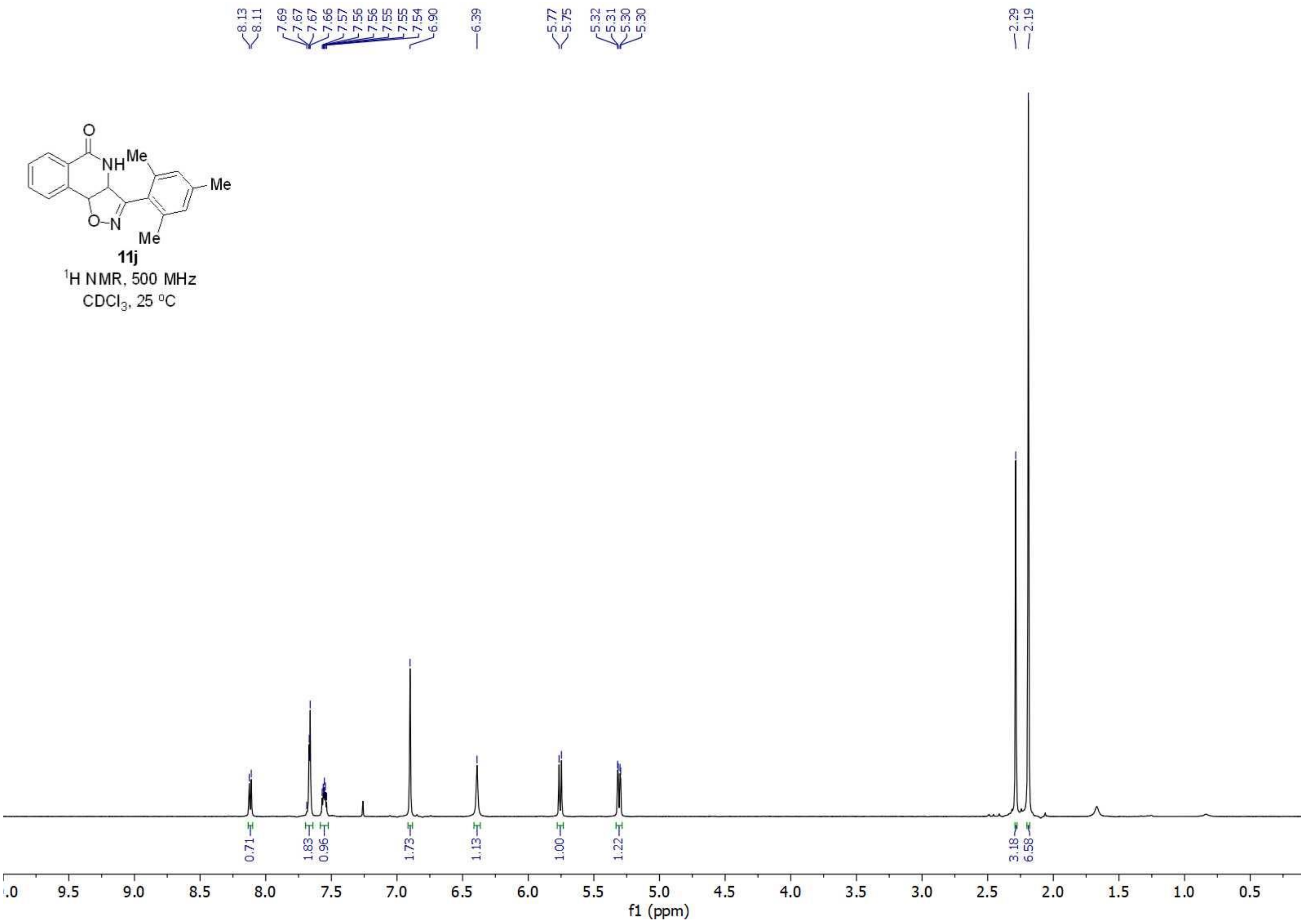

8.13
8.11
7.69
7.67
7.67
7.66
7.57
7.56
7.56
7.55
7.55
7.54
6.90
6.39
5.77
5.75
5.32
5.31
5.30
5.30
2.29
2.19
O
NH
Me
Me
O–N
Me
11j
¹H NMR, 500 MHz
CDCl3, 25 °C
0.71
1.83
0.96
1.73
1.13
1.00
1.22
3.18
6.58
9.5
9.0
8.5
8.0
7.5
7.0
6.5
6.0
5.5
5.0
4.5
4.0
3.5
3.0
2.5
2.0
1.5
1.0
0.5
f1 (ppm)

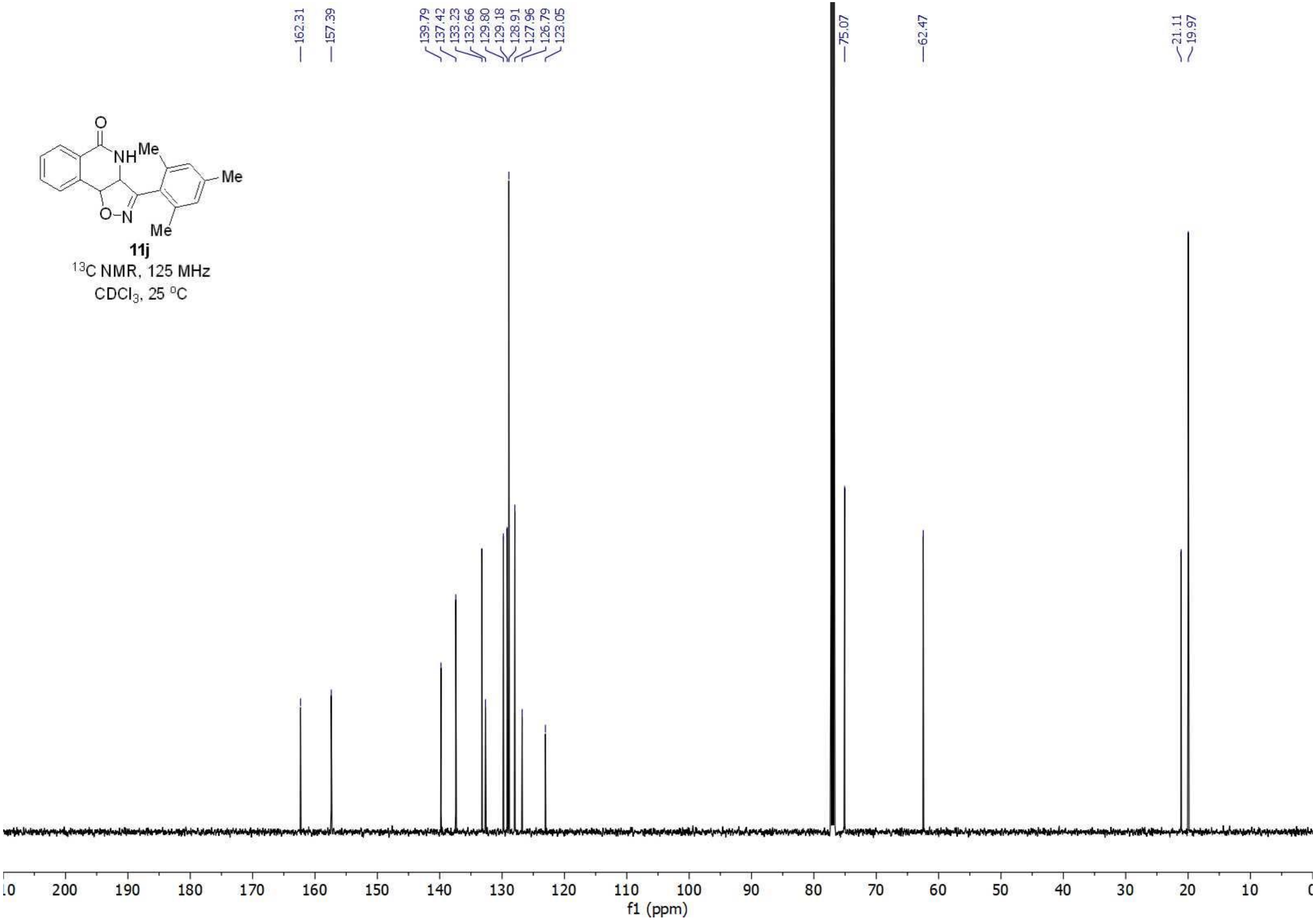
162.31
157.39
139.79
137.42
133.23
132.66
129.80
129.18
128.91
127.96
126.79
123.05
75.07
62.47
21.11
19.97
O
NH
Me
Me
Me
O–N
11j
13C NMR, 125 MHz
CDCl3, 25 °C
210
200
190
180
170
160
150
140
130
120
110
100
90
80
70
60
50
40
30
20
10
0
f1 (ppm)

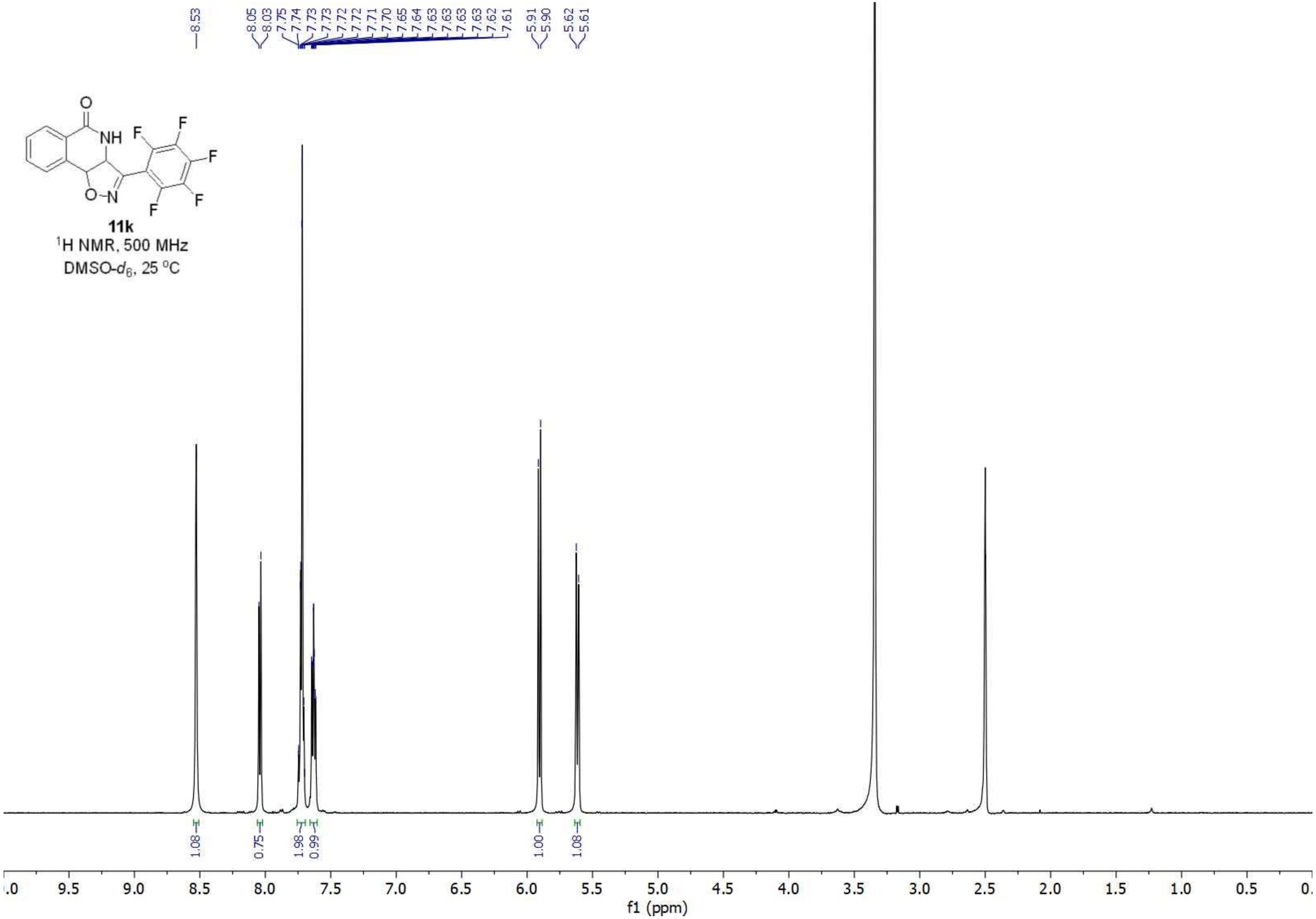
8.53
8.05
8.03
7.75
7.74
7.73
7.73
7.72
7.72
7.71
7.70
7.65
7.64
7.63
7.63
7.63
7.62
7.61
5.91
5.90
5.62
5.61
O
NH
F
F
F
F
F
O-N
11k
¹H NMR, 500 MHz
DMSO-d6, 25 °C
1.08
0.75
1.98
0.99
1.00
1.08
9.5
9.0
8.5
8.0
7.5
7.0
6.5
6.0
5.5
5.0
4.5
4.0
3.5
3.0
2.5
2.0
1.5
1.0
0.5
f1 (ppm)

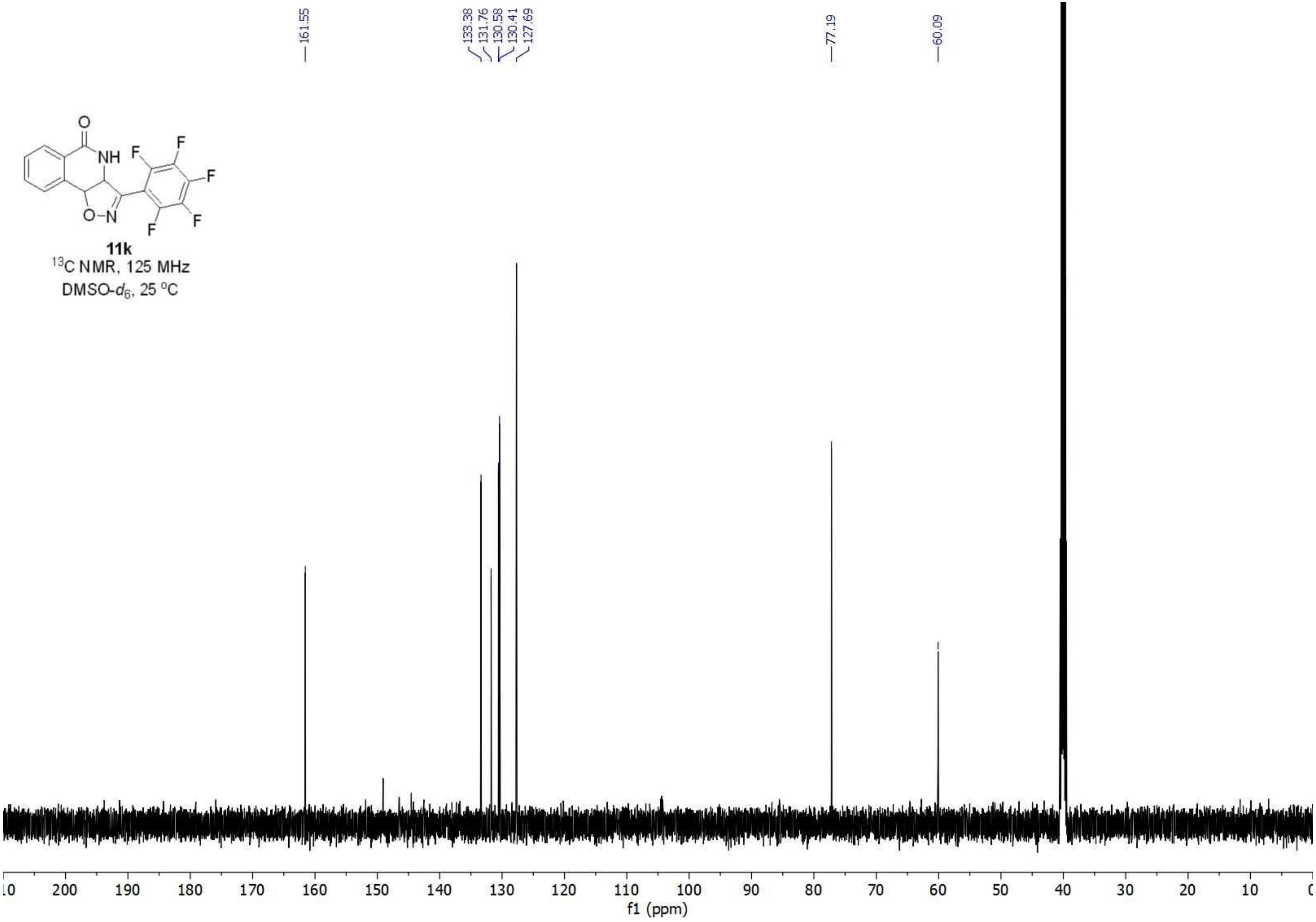

11k
^{13}C NMR, 125 MHz
DMSO-d_6, 25 °C

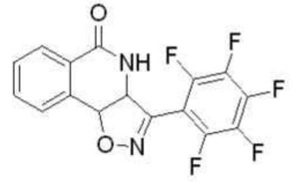

11k
^{19}F NMR, 470 MHz
DMSO-d_6, 25 °C

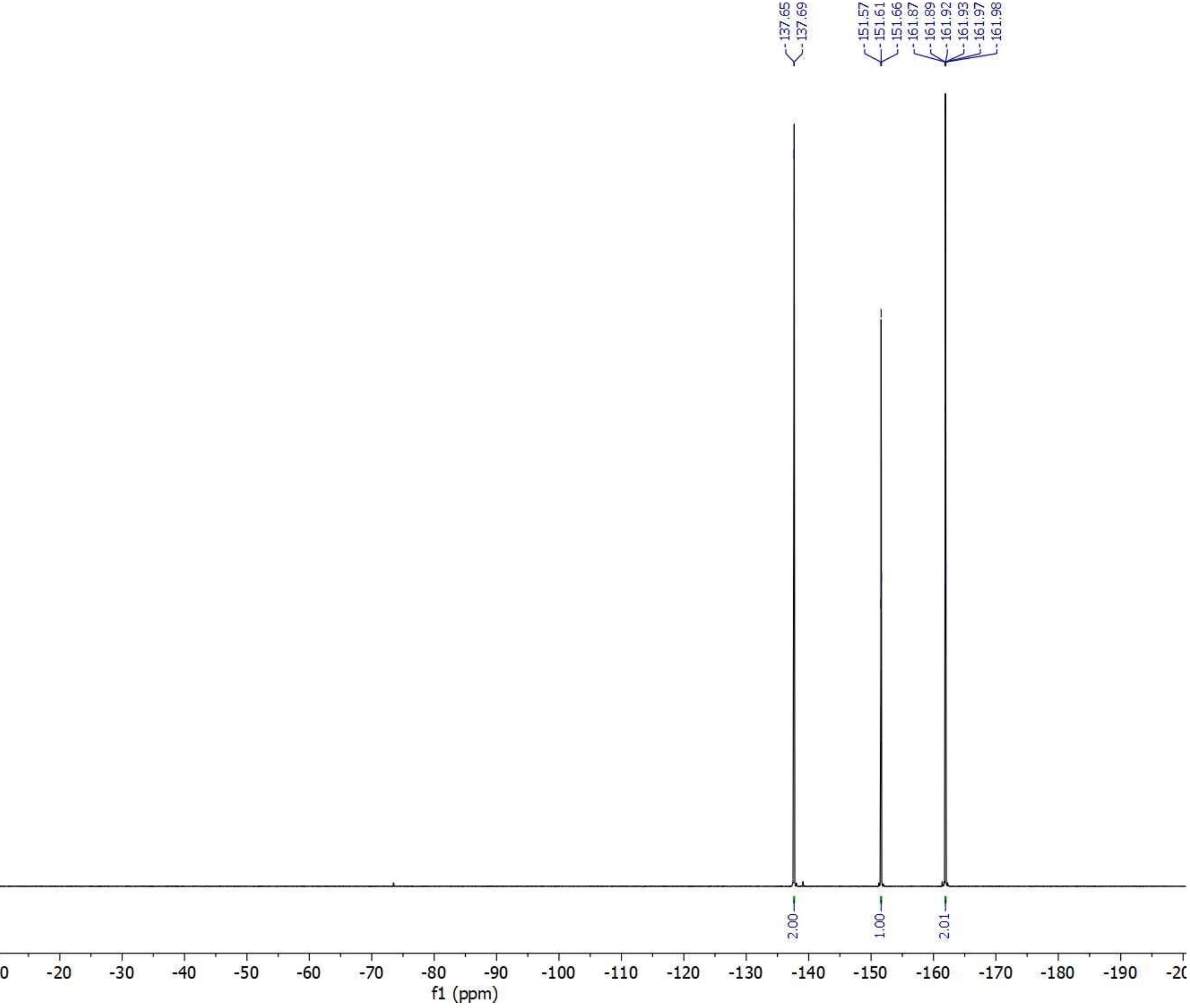

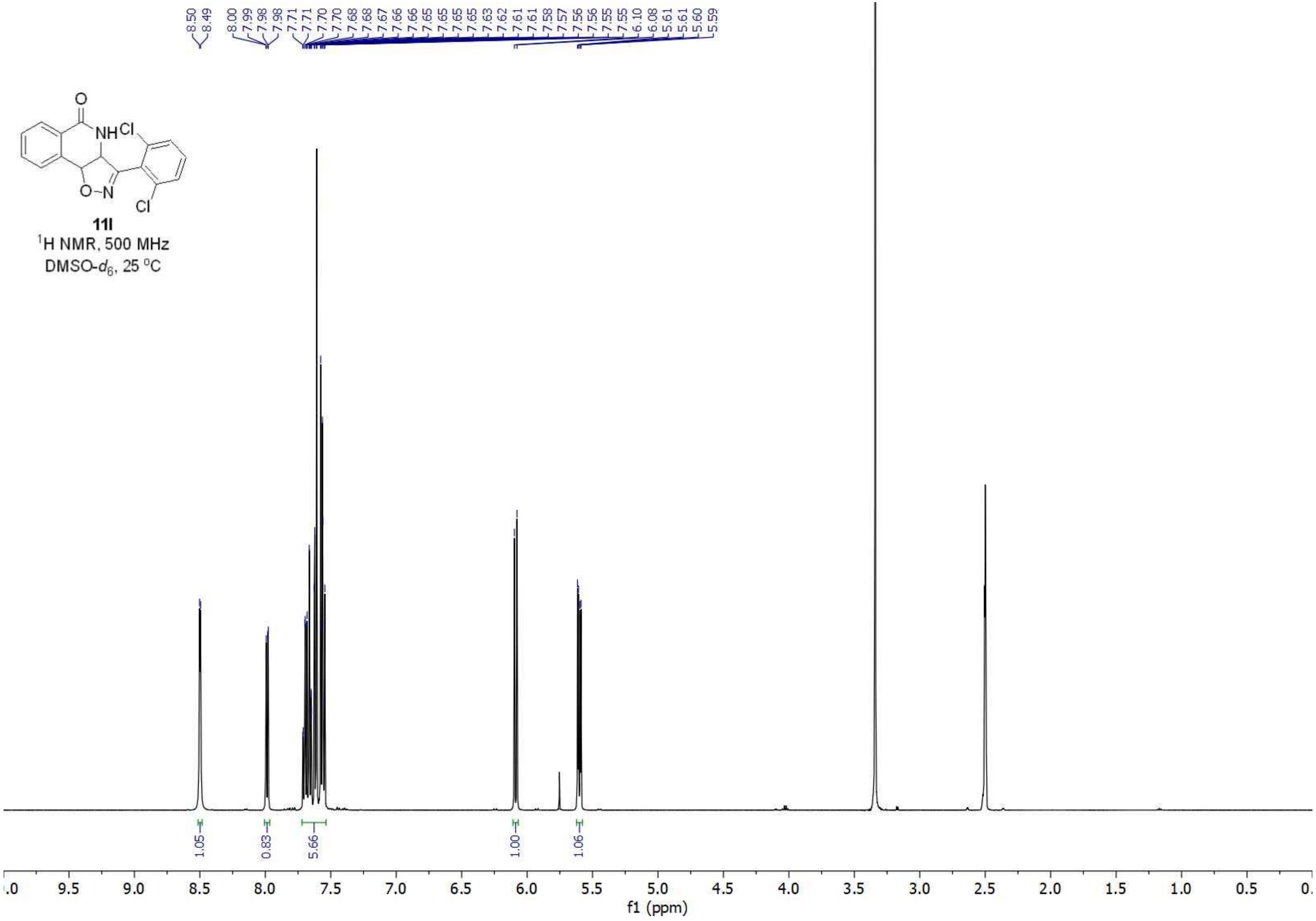

NH
Cl
O
O–N
Cl
11l
^{1}H NMR, 500 MHz
DMSO-d_6, 25 °C
8.50
8.49
8.00
7.99
7.98
7.98
7.71
7.71
7.70
7.70
7.68
7.68
7.67
7.66
7.66
7.65
7.65
7.65
7.63
7.62
7.61
7.61
7.58
7.57
7.56
7.56
7.55
7.55
6.10
6.08
5.61
5.61
5.60
5.59
1.05
0.83
5.66
1.00
1.06
9.5
9.0
8.5
8.0
7.5
7.0
6.5
6.0
5.5
5.0
4.5
4.0
3.5
3.0
2.5
2.0
1.5
1.0
0.5
f1 (ppm)

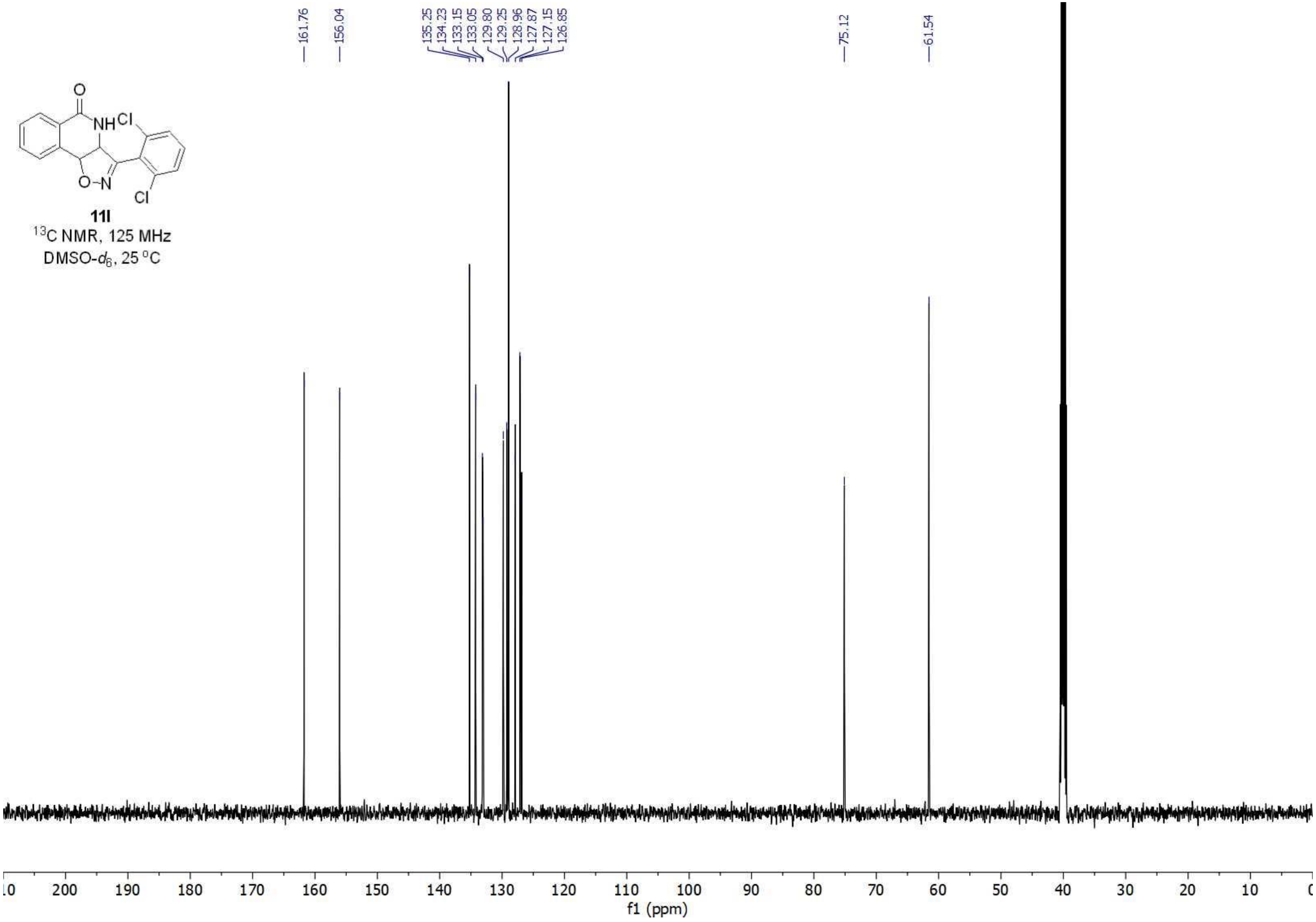
11l
^{13}C NMR, 125 MHz
DMSO-d_6, 25 °C
161.76
156.04
135.25
134.23
133.15
133.05
129.80
129.25
128.96
127.87
127.15
126.85
75.12
61.54
f1 (ppm)
